# Effect of Tension on Human Periodontal Ligament Cells: Systematic Review and Network Analysis

**DOI:** 10.3389/fbioe.2021.695053

**Published:** 2021-08-27

**Authors:** Changyun Sun, Mila Janjic Rankovic, Matthias Folwaczny, Sven Otto, Andrea Wichelhaus, Uwe Baumert

**Affiliations:** ^1^ Department of Orthodontics and Dentofacial Orthopedics, University Hospital, LMU Munich, Munich, Germany; ^2^ Department of Conservative Dentistry and Periodontology, University Hospital, LMU Munich, Munich, Germany; ^3^ Department of Oral and Maxillofacial Plastic Surgery, Martin-Luther-University Halle-Wittenberg, Halle (Saale), Germany

**Keywords:** periodontal ligament fibroblasts, tension, tissue remodelling, orthodontic tooth movement, mechanical stress

## Abstract

Orthodontic tooth movement is based on the remodeling of tooth-surrounding tissues in response to mechanical stimuli. During this process, human periodontal ligament cells (hPDLCs) play a central role in mechanosensing and mechanotransduction. Various *in vitro* models have been introduced to investigate the effect of tension on hPDLCs. They provide a valuable body of knowledge on how tension influences relevant genes, proteins, and metabolites. However, no systematic review summarizing these findings has been conducted so far. Aim of this systematic review was to identify all related *in vitro* studies reporting tension application on hPDLCs and summarize their findings regarding force parameters, including magnitude, frequency and duration. Expression data of genes, proteins, and metabolites was extracted and summarized. Studies’ risk of bias was assessed using tailored risk of bias tools. Signaling pathways were identified by protein-protein interaction (PPI) networks using STRING and GeneAnalytics. According to our results, Flexcell Strain Unit^®^ and other silicone-plate or elastic membrane-based apparatuses were mainly adopted. Frequencies of 0.1 and 0.5 Hz were predominantly applied for dynamic equibiaxial and uniaxial tension, respectively. Magnitudes of 10 and 12% were mostly employed for dynamic tension and 2.5% for static tension. The 10 most commonly investigated genes, proteins and metabolites identified, were mainly involved in osteogenesis, osteoclastogenesis or inflammation. Gene-set enrichment analysis and PPI networks gave deeper insight into the involved signaling pathways. This review represents a brief summary of the massive body of knowledge in this field, and will also provide suggestions for future researches on this topic.

## Introduction

Orthodontic treatment aims to align malpositioned teeth towards a functional optimal position by application of an appropriate force (Wichelhaus, 2017). This force leads to bone resorption in direction of the movement (“compressive side”) and bone formation on the opposite side (“tension side”). Orthodontic tooth movement (OTM) is therefore based on the controlled stimulation of bone remodeling by application of external force (“orthodontic force”). At the cellular level, OTM is based on remodeling processes in the periodontal ligament (PDL) and the alveolar bone (Cho et al., 2010; Chang et al., 2017). Placed between the teeth and the surrounding alveolar bone, the PDL is a heterogeneous connective tissue, that is composed of several different cell populations including but not limited to fibroblasts, macrophages, stem cells and endothelial cells (Marchesan et al., 2011). In the context of *in vitro* experiments, the term “PDL fibroblast” should be used carefully. Cells described as “PDL fibroblast” are commonly isolated from the middle third of the tooth root. For cell isolation either the “explant” or the “digestion” technique is employed (for further details: see discussion). Yet, both techniques will result in a heterogeneous mixture of different cell types (Marchesan et al., 2011).

The PDL is essential for maintaining the homeostasis and integrity of the tooth supporting tissue (Ren et al., 2015; Wu et al., 2019b) and plays a pivotal role in coping with physiological forces that occur during routine activities, i.e. speaking or mastication and non-physiological external forces (Pavasant and Yongchaitrakul, 2011). Involution and atrophy of PDL is induced by lack of recurring mechanical stimuli (Cohn, 1965), while exposure against excessive forces will impair the subtle balance between osteogenesis and osteoclastogenesis, ultimately leading to the disintegration and loss of the osseous tooth support (Nogueira et al., 2014a). Therapeutic mechanical force applied onto teeth is mediated to the alveolar bone via the PDL thereby inducing bone remodeling and OTM (Vansant et al., 2018). Periodontal ligament cells (PDLCs) play a vital role in the transduction of mechanical force to biological signals, achieving the balance between bone formation and resorption (Kook and Lee, 2012; Li et al., 2019). PDLCs can be activated in response to periodontal ligament injury followed by proliferation, migration and synthesis of new matrix components (Krishnan and Davidovitch, 2006). Potentially, PDLCs can differentiate into cementoblasts or osteoblasts and are involved in the repair and the regeneration of the periodontal tissues (Pavasant and Yongchaitrakul, 2011).

**FIGURE 1:**
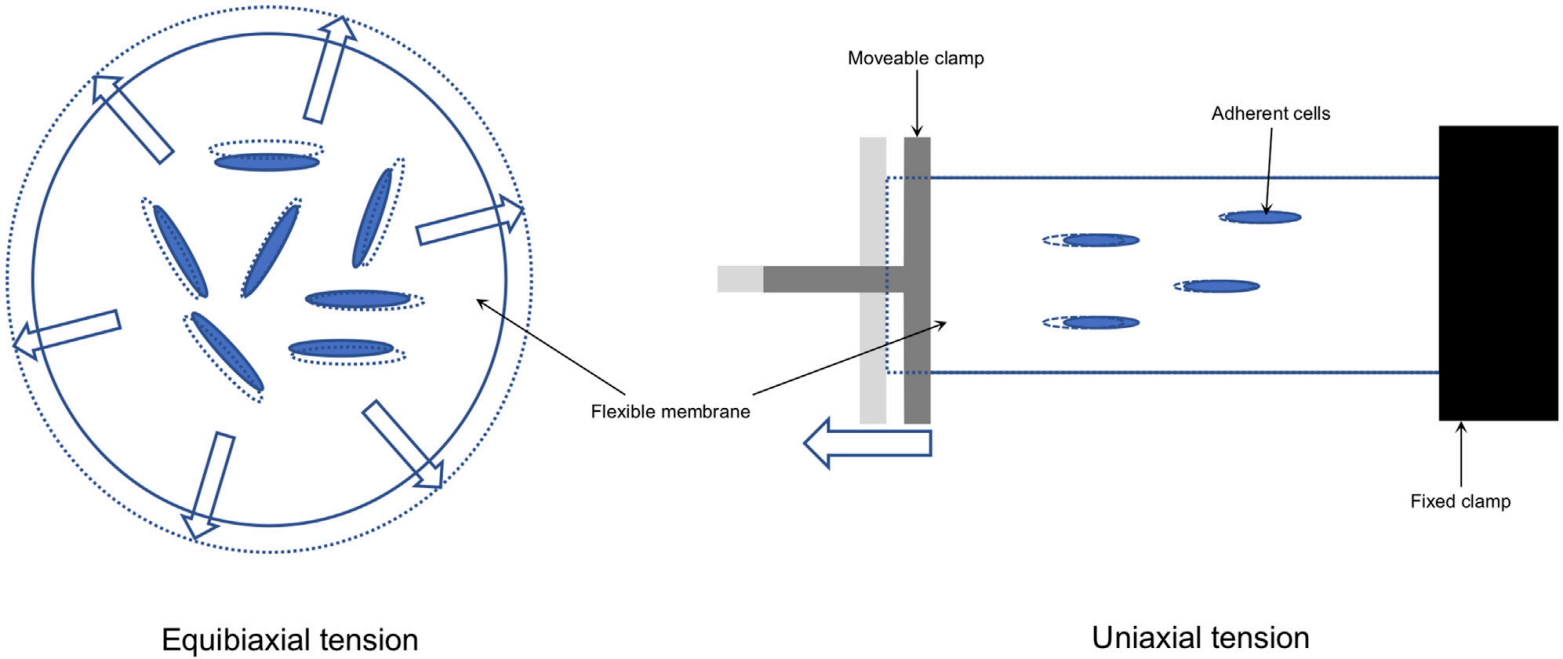
Tension is applied to adherent cells growing on a flexible surface (e.g. a. silicon membrane; solid lines) by elongation (dotted lines) of that surface either with equal forces acting in all directions in the same way (equibiaxial) or equal forces acting in one principle axis (uniaxial).

Due to the complex structure of the periodontium and to evaluate inter and intracellular signaling pathways, *in vitro* models have been established to simulate the two major mechanical stimuli occurring during OTM (Yang et al., 2015; Janjic et al., 2018; Vansant et al., 2018): tension and compression. The main working principles of these setups can be summarized as approaches in which tension is applied via substrate deformation, whereas compression is mainly applied via weight, hydrostatic pressure, or centrifugation (Yang et al., 2015). *In vitro* compression models were recently summarized (Janjic et al., 2018) and the underlying molecular signal transduction has been shown by numerous *in vitro* experiments (Baumert et al., 2004; Shi et al., 2019a; Shi et al., 2019b; Janjic Rankovic et al., 2020). Based on these reports various molecular pathways involved in OTM have been identified, including but not limited to genes and proteins which are related to osteogenesis, osteoclastogenesis, inflammation and apoptosis (Yang et al., 2015; Janjic et al., 2018; Vansant et al., 2018). To study the effect of tension on PDLCs, different *in vitro* models have been designed to apply continuous (“static”) or intermitted (“dynamic”) tension force along one principal axis (“uniaxial”) or along all axes in all directions (“equibiaxial”) of a cell (definition according to Lee and von Recum, 2015)(Figure 1). However, the force parameters in terms of dynamic and static tension used show enormous heterogeneity, depending on the specific objectives of the experiments.

Therefore, this systematic review aimed to summarize the data on different *in vitro* tension models applied to human PDLCs (hPDLCs) as well as the effect of tension on the expression of genes and proteins. Specifically, this systematic review aimed 1) to identify all relevant studies applying tension on hPDLCs e.g. to simulate orthodontic force or other clinically relevant forces; 2) to make an assessment of the methodological and reporting quality of the included studies; 3) to summarize the biological and force parameters, and the commonly adopted methods for detecting biological regulation; and 4) to identify the most frequently investigated genes/proteins and their regulation, as well as the biological processes and pathways that might be affected by tension in hPDLCs.

## Materials and Methods

This systematic review was conducted following the “Preferred Reporting Items for Systematic Reviews and Meta-Analyses” (PRISMA) guidelines (Moher et al., 2009). The protocol of this systematic review was finalized before data collection. A registration in the PROSPERO database was not possible, since only *in vitro* studies were included.

### Eligibility Criteria

Inclusion criteria were defined in accordance with the “P.I.C.O.” framework (Schardt et al., 2007):

• P(atient): human periodontal ligament cells (hPDLCs) or human periodontal ligament derived stem cells (hPDLSCs);
• I(ntervention): *in vitro* static and dynamic tension (e.g. to simulate orthodontic force or other clinically relevant forces);
• C(ontrol): human periodontal ligament cells (hPDLCs) or human periodontal ligament derived stem cells (hPDLSCs) not subjected to mechanical force;
• O(utcome): force parameters (i.e. apparatus, force duration, force magnitude, frequency of force exposure) and regulation of gene, protein and/or metabolite expression in response to tension.

The following exclusion criteria were applied:

• *In vivo* studies
• *In vitro* studies not applying tension on hPDLCs or hPDLSCs
• Reviews
• Studies not reporting quantitative data on gene or protein expression
• Application of force other than tension or the specific type of force is undefined
• 3D model
• Co-culture
• Articles not published in English

### Search Strategy and Study Selection

The search strategy considered keywords concerning the specific objectives of the studies, the force applied and the cells exposed to experimental force and were summarized in Table 1. PubMed search was completed on 31-01-2020 and the results were imported into EndNote^®^ X9.3.1 (Clarivate Analytics, Philadelphia, Pennsylvania, United States).

**TABLE 1:** Final PubMed search strategy applied.

Field		Force		Cells
orthodont* OR	AND	BioFlex culture plates OR	AND	fibroblast* OR
tooth movement OR		biomechanic* OR		PDL OR
periodont*		mechanical force* OR		hPDLCs OR
		load* OR		hPDLFs OR
		stretch* OR		hPDLF OR
		tension OR		progenitor cell* OR
		tensile OR		stem cell* OR
		dynamic structural remodeling OR		human PDL-cells OR
		equi-biaxial strain OR		human PDL-fibroblasts OR
		Flexercell OR		human PDLFs OR
		four-point bending OR		human PDLs OR
		mechanical coupling OR		human periodontal ligament OR
		mechanical deformation OR		ligament fibroblast OR
		mechano-sensitive OR		periodontal tissue OR
		mechanostimulation OR		Periodontium
		mechanotransduction OR		
		petri dish OR		
		flexible bottom OR		
		elastic membrane OR		
		silicon* OR		
		strength OR		
		stress OR		
		substrate strain OR		
		Tensile		

Search phrase: (orthodont* OR tooth movement OR periodont*) AND (BioFlex culture plates OR biomechanic* OR mechanical force* OR load* OR stretch* OR tension OR tensile OR dynamic structural remodeling OR equi-biaxial strain OR Flexercell OR four-point bending OR mechanical coupling OR mechanical deformation OR mechano-sensitive OR mechanostimulation OR mechanotransduction OR petri dish OR flexible bottom OR elastic membrane OR silicon* OR strength OR stress OR substrate strain OR tensile) AND (fibroblast* OR PDL OR hPDLCs OR hPDLFs OR hPDLF OR progenitor cell* OR stem cell* OR human PDL-cells OR human PDL-fibroblasts OR human PDLFs OR human PDLs OR human periodontal ligament OR ligament fibroblast OR periodontal tissue OR periodontium).

First, unrelated studies were excluded after reading titles and abstracts according to the eligibility criteria defined. Then, full texts of the remaining studies were acquired. After full text reading, articles not fulfilling the eligibility criteria were excluded (Supplementary Table S1), and those in accordance with the inclusion criteria were used for data extraction (Supplementary Table S2). Any disagreements or uncertainties during both steps were discussed with two other review authors (U.B. and M.J.R.) until agreement was achieved.

### Risk of Bias Assessment (Definition and Table for Assessment)

Risk of bias of the included *in vitro* studies was assessed using the methods described by Vansant et al. (2018) and Samuel et al. (2016). Methodological risk of bias was evaluated using 15 criteria and reporting risk of bias using 10 criteria (Supplementary Table S3). Each criterion was scored “low risk of bias” (“+”), “high risk of bias” (“−“), “incomplete or unclear risk of bias” (“?”) or “not applicable” (“n.a.”) based on the low risk of bias definitions given in Supplementary Table S3 for both, reporting and methodological quality. Low risk of bias (“LoB”) and if necessary high risk of bias (“HoB”) of the different criteria were defined according to the information provided in the aforementioned publications, citations therein and the following additional sources: “OHAT Risk of Bias Rating Tool for Human and Animal Studies” and “Biophysical Journal’s Guidelines for the Reproducibility in Biophysics Research” (details in Supplementary Table S3). To simplify data entry during the assessment, data sheets for both risk of bias assessments were developed (Supplementary Table S3).

All included articles were scored by two authors (C.S. and M.J.R.). Any disagreement was discussed internally until consensus was achieved. The results of the risk of bias assessment were recorded and summarized in predefined tables (Supplementary Tables S4.1, S4.2).

### Data Extraction

After final selection of relevant studies, the following information on experimental design and outcome were extracted and summarized: reference (author, year, journal); cells used (age/gender of donors, tooth type, isolation method, cell culture passages and cell density used in the experiments); force applied (“dynamic”/”static” and “equibiaxial”/”uniaxial” force application; its duration, frequency of exposure, magnitude, and the device used); genes analyzed (official gene symbol if applicable) with reference to force application and the methods applied to measure their expression. “Gene or analyte investigated”, “Cells used” and the details on the “Force apparatus” were extracted using the original phrases from the studies and recorded in Supplementary Table S2. In addition, gene expression patterns including peak expression and the reported units were recorded; fold changes and ratios were calculated, if applicable.

#### Information Related to Genes and Proteins

Specificity of primers used in PCR reactions was verified with Primer-BLAST (https://www.ncbi.nlm.nih.gov/tools/primer-blast/). All genes were reported using their official gene symbol according to the HUGO Gene Nomenclature Committee (HGNC; URL: https://www.genenames.org). For protein data, antibody or ELISA specificity was verified using information provided within the studies and the information given by the suppliers. If possible, official gene symbols according to HGNC were applied. If antibody specificity was not sufficient, e.g. no discrimination between isoforms or gene variants, antibody targets were recorded according to the manufacturer given in that publication.

**FIGURE 2:**
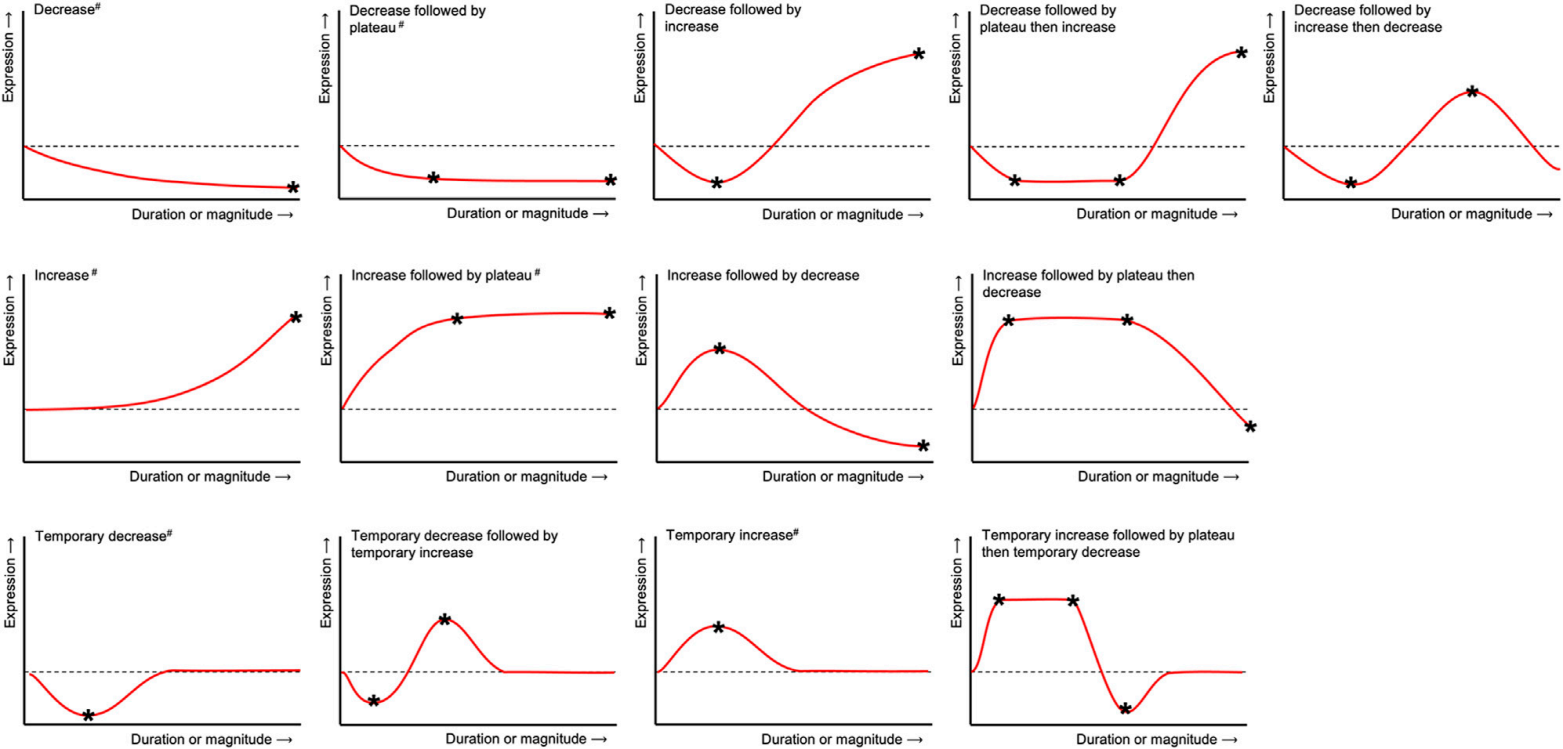
Terms used to describe the general expression patterns in hPDLCs after tension application for each gene, protein or metabolite included herein. Numerical data describing specific maxima and minima (*) was collected and reported in Supplementary Tables S2, S7. Expression patterns labeled with a double cross (#) were mapped on the protein-protein interaction networks constructed from gene lists shown in Figure 5.

The expression patterns of genes and/or proteins were described using specific terms (Figure 2) and numerical data describing specific maxima and minima (stars in Figure 2) was collected. Data was directly acquired from the publications itself or extracted from graphs using Engauge Digitizer Software version 10.12 (URL: https://markummitchell.github.io/engauge-digitizer). To allow comparisons between gene or protein expression data from different sources, ratios (experimental condition vs control) or fold changes were calculated if not given by the authors. Gene expression was reported as “fold change” (FC), if its calculation was done either according to Livak and Schmittgen (2001) or similar sources or was clearly described as “2^−ΔΔCt^” or “ΔΔCt”. Gene expression was designated as “ratio”, if the control was “defined as 1”, otherwise it was described as “relative change” (“rel”). Results reported from more than one donor were listed separately.

#### Information Related to Force

The frequency of force exposure used in all dynamic tension studies was reported as “Hz” if possible. If frequency was reported in other units, conversion was done according to the information given. Any inconsistencies were resolved by discussion between the authors. Information about the force apparatuses was either directly collected from the publications, by searching the manufacturer information or Google^®^ patent search (URL: https://patents.google.com) if applicable. Equibiaxial and uniaxial tension (Figure 1) were distinguished depending on the direction of the force applied in relation to the hPDLCs using information contained in the publications and citation chaining. The information was defined “unclear” or “incomplete”, if insufficient information was given that was even not resolved after communication with the manufacturer.

#### Summary Statistics

A summary of statistics on the force apparatuses was prepared from a unified list based on “apparatus”, followed by sorting according to the force type (i.e. “dynamic”/“static” and “equibiaxial”/“uniaxial”). Afterwards, publications using the same type of apparatus were combined into the same category for further analysis. A summary statistic on the different force parameters was compiled from a unified list of publications based on tension type and tension frequency. From each publication reporting dynamic tension application, maximum duration of force exposure and mainly adopted magnitude were summarized for the same frequency. Studies utilizing static tension were classified first by magnitude followed by maximum force duration. Replicates derived from the same study were removed and all analytes were ranked according to abundance.

#### Gene-List and Protein-Protein Interaction Network Analysis

**FIGURE 3:**
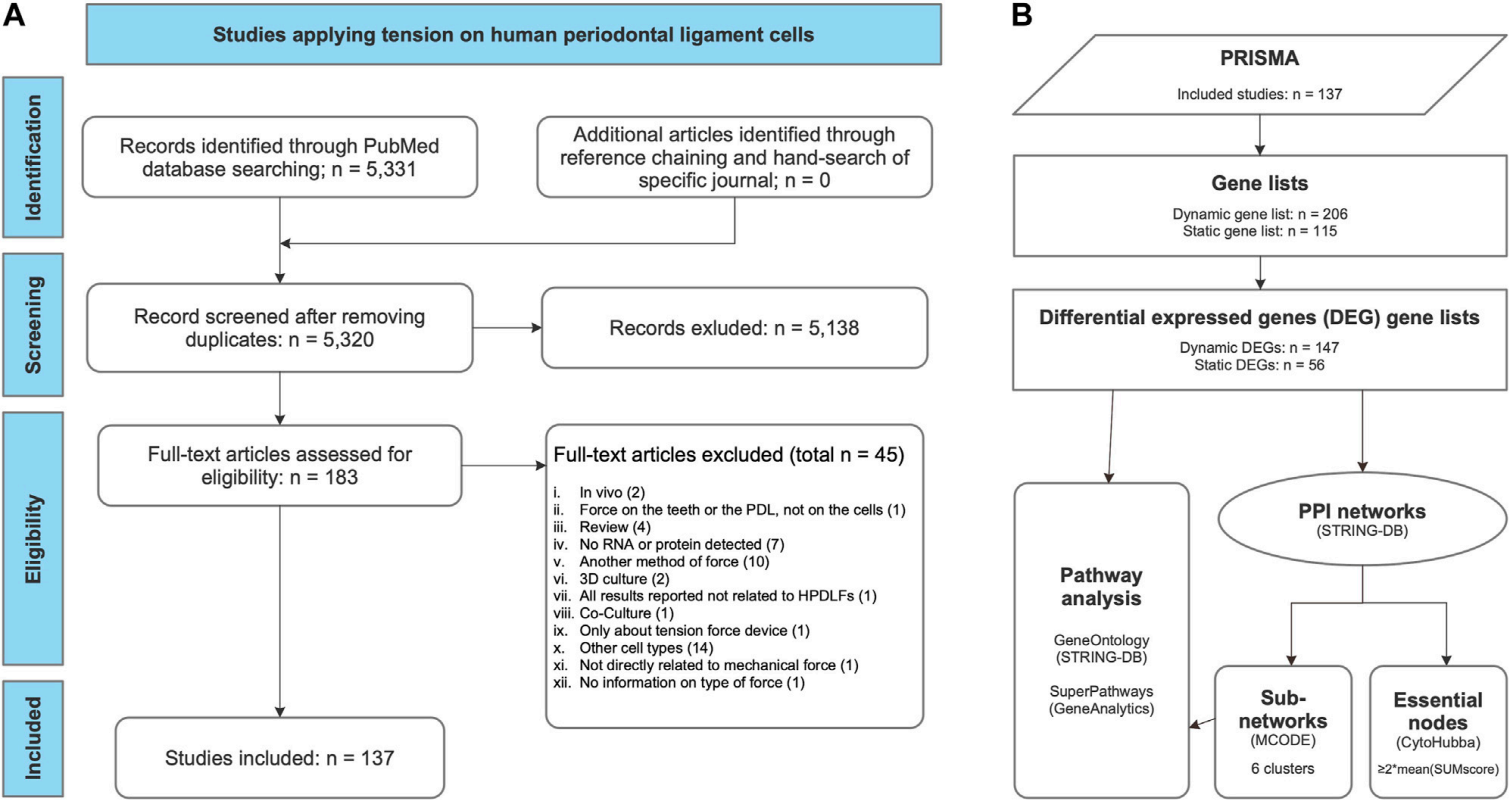
Workflows applied in this systematic review. **(A)** PRISMA flow diagram for the whole process of study selection according to Moher et al. (2009). **(B)** Gene set enrichment and network analysis of gene lists derived from the review process. Gene lists were compiled, listing the examined genes, proteins or metabolites studied either after dynamic or static tension application (Table 3, Supplementary Table S2). From these, differential expressed gene (DEG) lists were generated according to the specified criteria (Table 4). For each of these two DEG lists (dynamic DEG, static DEG) protein-protein interaction (PPI) networks were constructed using STRING-DB (Szklarczyk et al., 2019) (Figures 5A,B) and pathway analysis was conducted quering “GeneOntology/Biologcal Process” (Ashburner et al., 2000) and GeneAnalytics’ “SuperPath” databases (Ben-Ari Fuchs et al., 2016) (Supplementary Table S8). Subnetworks were identified in both PPI networks using MCODE (Bader and Hogue, 2003) (Table 4; Figures 5C,D) and essential nodes, so called “hub genes”, using *cytoHubba* (Chin et al., 2014) (Table 5; Figure 5).

Based on the complete lists of examined genes, differential expressed gene (DEG) lists were compiled according to the following criteria: the gene was identified unequivocally and changes in gene expression due to force application were reported using the terms defined in Figure 2. Depending on force application, the genes were assigned either to the “dynamic” or to the “static” gene list. Both DEG lists were used to generate protein-protein-interaction (PPI) networks and for gene list enrichment (Figure 3B).

To predict potential interactions between the DEGs at the protein level, PPI networks were constructed querying the “Search Tool for the Retrieval of Interacting Genes/Proteins” database (STRING-DB) (v11.0; URL: https://string-db.org) (Szklarczyk et al., 2019) using the *stringApp* plugin version 1.5.1 (Doncheva et al., 2019) with *Cytoscape* version 3.8.0 (Shannon et al., 2003; Su et al., 2014). A minimum required combined score of 0.7 was applied, i.e. only high confidence interactions were included in the predicted networks. Cluster analysis and cluster visualization of both “dynamic” and “static” PPI networks was done applying the “Molecular Complex Detection” (MCODE) algorithm (Bader and Hogue, 2003) as implemented in the *clusterMaker2* app version 1.3.1 (Morris et al., 2011) with default settings and “fluff” activated. Hub genes, i.e. essential genes in a network, were identified with *cytoHubba* version 0.1 (Chin et al., 2014) using default settings. This plugin applies eleven different local and global topological methods to the nodes of a given network. For each node, a total score was calculated based on these eleven measures. A node was considered as a hub node, if its total score was at least twofold higher than the mean total score of all nodes of that particular network. Networks were visualized with *Cytoscape* version 3.8.0.

*StringApp* was also applied for gene list enrichment using the “GeneOntology/Biological Process*”* database (Ashburner et al., 2000). The “SuperPaths” database was analyzed online with “GeneAnalytics” (version 4.14 Build 1; URL: https://ga.genecards.org) (Ben-Ari Fuchs et al., 2016). To increase specificity, results from both databases were filtered according to the proportion of query genes in relation to the number of background genes of the specific database entry and a cut-off of ≥0.05 (i.e. 5 %) was applied. In all cases the ten most significant terms or pathways were reported. Individual gene set enrichment was applied to 1) the two complete networks and 2) each identified cluster, using “GeneAnalytics” and *stringApp*.

## Results

### Study Selection

The whole process of study selection was summarized in the PRISMA flow diagram (Figure 3A) (Moher et al., 2009). The applied search strategy identified 5,331 publications. No additional articles were identified through reference chaining or hand-search of specific journals. After removing 11 duplicates, 5,320 studies were left, of which 5,138 publications were excluded after title and abstract reading according to the defined criteria. Afterwards, 182 publications were assessed by full-text reading, of which 45 were excluded according to the exclusion criteria defined previously (Figure 3A; Supplementary Table S1).

### Risk of Bias

**FIGURE 4:**
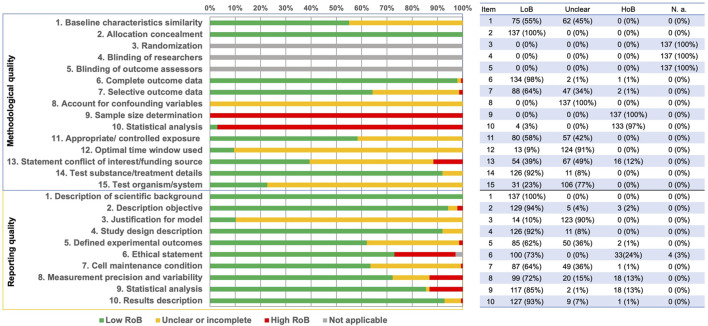
Summary of the risk of bias assessments for methodological (upper panel) and reporting quality risk of bias (lower panel). The tabulated data on the right reported frequency and percentage [n(%)] for each item according to its scoring: “LoB” – low risk of bias, “Unclear” – unclear or incomplete, “HoB” – high risk of bias, “N.a.” – not applicable.

Methodological quality was assessed using 15 criteria (Supplementary Table S3). The criteria “Randomization”, “Blinding of researchers”, and “Blinding of outcome assessors” were not applicable to *in vitro* studies. The results of the other risk of bias criteria showed a large variability. “Sample size determination” and “Statistical analysis” were mostly assessed as “high risk of bias” (“HoB”). “Accounting for confounding variables”, “Optimal time window used” and “Test organism/system” were found to have a high level of “incomplete or unclear risk of bias”. With reference to the remaining criteria, most of the studies were assessed as “low risk of bias” (“LoB”) (Figure 4; details in Supplementary Table S4.1).

The reporting quality of the publications was higher in comparison to the methodological quality, since more studies were classified as “LoB” (Figure 4, Supplementary Table S4.2). Only the criterion “Justification for model” was found to have a large percent of “Incomplete or unclear risk of bias” (Figure 4; details in Supplementary Table S4.2).

### Tension Characteristics

In 30 out of 137 qualified studies (∼22%) static tension was applied, whereas 103 out of 137 included studies (∼75%) focused on the effect of dynamic tension (Supplementary Table S5). Direct comparison between static and dynamic tension was conducted in three studies (∼2%) (Papadopoulou et al., 2017; Wada et al., 2017; Memmert et al., 2020). One study did not clearly define the specific force type applied (Supplementary Table S5).

#### Devices for Tension Application

Regarding the type of apparatus, all included studies were identified as either equibiaxial or uniaxial (Figure 1). Various apparatuses were used in these studies either for equibiaxial or uniaxial tension (Table 2; Tables S5.1 and S5.2 in Supplementary Table S5).

**TABLE 2:** Summary statistics according to tension type and apparatus. Since some publications applied two tension types, the total number given here is larger (*n* = 140) than the number of studies identified (*n* = 137).

*Tension type (n)*	Tension applied with (*n*)	Fraction of Tension type (%)
Dynamic equibiaxial (72)	Flexcell Strain Unit^®^ and its revisions (53)	73.6
	Bioflex^®^-plate based apparatus (9)	12.5
	Other silicone (not Bioflex^®^)-plate based or elastic membrane-based apparatuses (10)	13.9
Dynamic uniaxial (34)	STREX^®^ STB-140 (10)	29.4
	Silicone (not Uniflex^®^)-plate based and other elastic membrane-based apparatuses (10)	29.4
	Flexcell Strain Unit^®^ and its revisions using Uniflex^®^ plates (8)	23.5
	Four-point bending system (6)	17.6
Static equibiaxial (31)	Flexcell Strain Unit^®^ and its revised version (11)	35.5
	Petriperm^®^ dish (10)	32.2
	Bioflex^®^ based apparatus (7)	22.6
	Lumox^®^ dish (2)	6.5
	Tension incubator (1)	3.2
Static uniaxial (2)	Silicone dishes (1)	50.0
	STREX^®^ system (1)	50.0
Not given (1)	Not given (1)	

*Dynamic equibiaxial tension*: Fifty three studies applied dynamic equibiaxial tension using the Flexcell Strain Unit (Flexcell^®^ International Corporation, Burlington, NC, United States) and its revisions (FX-2000, FX-3000, FX-4000, FX-5000) (Banes et al., 1985). This system employs tension to cells seeded on elastic silicone membranes fixed in special 6-well plates (Bioflex^®^ plates; Flexcell^®^ International Corporation) by application of a vacuum below the flexible membrane. In nine studies, Bioflex^®^ plates were used together with individually constructed devices, including the “CESTRA” (Deschner et al., 2007) and the “Cell Extender” devices (Wada et al., 2017) (Table 2; Supplementary Table S5.1 in Supplementary Table S5). Ten studies used different non-Bioflex^®^-plate based silicone or elastic membrane-based apparatuses including the “Cell Strain Unit (CSU)” (Hao et al., 2009), which was used in eight studies.

*Dynamic uniaxial tension*: Application of dynamic uniaxial tension using the “STB-140” system (STREX^®^ Inc., Osaka, Japan) was described in ten publications. The Flexcell Strain Unit^®^ in combination with Uniflex^®^ culture plates (Flexcell^®^ International Corporation) was adopted in eight studies. Non Bioflex^®^-plate based silicone and other elastic membrane-based apparatus were adopted in ten studies. Four-point bending systems were employed in six studies (Table 2; Supplementary Table S5.2 in Supplementary Table S5).

*Static tension*: To apply static equibiaxial tension, the Flexcell Strain Unit and its revisions (FX-3000™, FX-4000™, FX-5000™) were adopted in eleven studies. Apparatuses based on the Petriperm™ dish were used in ten studies, in which the dish was deformed by the weight placed onto a spheroidal template. Bioflex^®^-based devices were used in seven studies, Lumox^®^ culture dishes were adopted in two and the “*tension incubator*” in one study (Table 2; Supplementary Table S5.3 in Supplementary Table S5). Static uniaxial tension was applied in two studies (Table 2; Supplementary Table S5.4 in Supplementary Table S5): one with the STREX system and the other via a “*silicone dishes based in-house designed device*” with a moving clamp (Papadopoulou et al., 2017).

In summary, dynamic tension was more frequently investigated than static tension, and equibiaxial tension was more commonly adopted than uniaxial tension, both with a ratio ∼3:1.

#### Force Magnitude and Duration in Equibiaxial and Uniaxial Tension

*Dynamic equibiaxial tension* was applied using frequencies between 0.005–1 Hz, with 0.1 Hz used in the majority of the studies. The maximum magnitude most frequently adopted varied between 1–24% of which 10 and 12% were more commonly applied. The mainly adopted duration of force exposure varied from 1 h to 6 days, using 48 and 72 h in the majority of studies (Supplementary Table S5.5).

For *dynamic uniaxial tension* frequencies between 0.005 – 1 Hz were used. The most frequently adopted frequency was 0.5 Hz. The magnitude varied between 0.2 and 33%, with magnitudes of 10 and 12% being most frequently applied. Force duration ranged from 1 h to 7 days and 48 h was the most frequent one (Supplementary Table S5.6).

*Static equibiaxial tension* was applied with force magnitudes varying between 0.28 and 35%, mimicking physiological or pathological mechanical force. The most frequent magnitude was 2.5%. Force duration varied between 0.5 h and 15 days, whereby 12 h was the most commonly used one (Supplementary Table S5.7).

For *static uniaxial tension*, magnitudes of 5%, 8 and 10% were applied for a maximum of 12, 3, and 12 h, respectively (Supplementary Table S5.8).

### Genes, Proteins and Metabolites Analyzed

Relevant data on 205 genes, proteins or metabolites in relation to dynamic tension application to hPDLCs was extracted from 104 publications (Table 3). Static tension was applied in 33 publications and expression profiles of 115 different genes, proteins or metabolites related to force application were determined (Table 3). Genes or proteins that were not clearly assigned to a specific gene symbol due to ambiguities in the reported PCR primers or antibodies used in western blot or ELISA procedures were also included (e.g. “COL1A1/COL1A2” or “MAPK3/MAPK1”).

**TABLE 3:** Genes, proteins and metabolites analyzed in the included studies. Differential expressed genes (DEGs) used for the subsequent gene list enrichment analysis are given in bold.

Dynamic tension	Static tension
**ACE**, **ACTA2**, ACTB, ACVR2B, **ACY1**, **ADRB2**, **AGT**, **AGTR1**, AGTR2, AKT1, **ALPP**, **AMDHD2**, ARHGDIA, **ATF1**, **ATF4**, ATP, **BCL2**, **BGLAP**, **BGN**, **BMP2**, **BMP4**, **BMP6**, **BMP7**, **BMPR1A**, BMPR1B, **BMPR2**, CASP1, **CASP3**, CASP3/CASP7, **CASP5**, CASP7, CASP8, CASP9, CCDC88A, **CCL2**, **CCL20**, **CCL3**, **CCL5**, **CCN1**, **CCN2**, CCND1, **CCR5**, **CDC42EP2**, CFL1, **COL12A1**, **COL1A1**, COL1A1/COL1A2, **COL3A1**, **COL4A1**, **COL5A1**, **CREB1**, CSF1, CTNNB1, **CXCL8**, **BHLHE40**, DEFB1, **DEFB103B**, **DEFB4A**, DIAPH1, **DKK1**, **DVL2**, **EGFR**, EIF2AK3, ELN, **FBLN5**, FBN1, FBN2, **FGF2**, FN1, **FOS**, **FST**, **GATA4**, **GDF2**, **GDF5**, **GJA1**, **GLI2**, **GOSR1**, **GRIA3**, **GRIN1**, **GRIN2C**, **GRIN2D**, **GRIN3A**, **GRIN3B**, **GRM2**, **GRM3**, **GRM4**, **GRM5**, **GRM6**, **GSDMD**, Glutamate, **HACD1**, **HIF1A**, **HMOX1**, **HOMER1**, **HSPA5**, **IBSP**, **IER3, IGF1**, **IL10**, **IL11**, **IL12A**, IL18, **IL1B**, **IL1RN**, **IL6**, **IL6R**, **ITGA1**, **ITGA3**, ITGAV, **JUN**, **KLF10**, **LATS1**, LEF1, **LIMD1**, LTBP2, MAPK14, MAPK3/MAPK1, MAPK7, MAPK8, MAPK8/MAPK9/MAPK10, **MCAM**, **MEF2C**, **MGP**, **MMP1**, **MMP14**, **MMP2**, **MMP3**, MMP8, **MSX1**, **MSX2**, **MYH7**, **MYL2**, **MYL7**, **NAMPT**, NFKB1, NFKBIB, **NKX2-5**, **NLRP1**, **NLRP3**, **NOG**, NOS2, NOS3, **NPPA**, **NPPB**, Nitric oxide, **P2RY1**, PARP1, PFN1, PGE_2_, **PLAT**, PLAT/PLAU, PLAU, **POSTN**, PTGER1, **PTGER2**, PTGER3, **PTGER4**, **PTGS1**, **PTGS2**, **PYCARD**, **REN**, **RHOA**, **ROCK1**, ROCK1/ROCK2, **RSPO2**, **RUNX2**, **RXFP1**, RXFP2, **SATB2**, SERPINE1, SERPINF1, **SIRT1**, **SLC17A7**, **SMAD7**, **SP7**, SPARC, **SPP1**, **SPRY2**, **SQSTM1**, STMN1, **TAZ**, **TEAD1**, **TEAD2**, **TGFB1**, TGFBI, **TGFBR1**, **TGFBR2**, **TIMP1**, **TIMP2**, **TLR2**, **TLR4**, **TNF**, **TNFRSF11B**, **TNFSF11**, **TNNT2**, **TP53BP2**, **TPM1**, **UNC50**, **VEGFA**, WASL, WNT3A, **WTIP**, **XBP1**, **YAP1**	**ALPP**, **ATG10**, **ATG4C**, **ATG7**, **BAD**, **BCL2**, **BGLAP**, **BID**, cAMP, CCNA1/CCNA2, **CCND1**, CCNE1, CDK2, CDK4, CDKN1A, CDKN1B, **COL1A1**, COL1A1/COL1A2, **CRADD**, **CTSB**, **CTSL**, **DAPK1**, **EFNB2**, **EPHB4**, **FAS**, **FOS**, **GDF15**, HMGB1, **IGF1**, **IGF1R**, **IGF2**, **IGFBP1**, **IGFBP3**, **IGFBP5**, **IL1B**, IL1B/IL1A, **IL6**, **IRS1**, ITGA1, ITGA2, ITGA3, ITGA4, **ITGA5**, ITGA6, ITGAV, **ITGB1**, ITGB3, ITGB4, **JUN**, MAP1LC3A, MAP4, MAPK14, MAPK3/MAPK1, MAPK8, MAPK8/MAPK9/MAPK10, MKI67, **MMP1**, **MMP12**, MMP14, **MMP2**, MMP8, MMP9, MYO1C, **NFKB1**, **NOS2**, **PCNA**, PGE_2_, PIK3CG, **PLAT**, PLAT/PLAU, PLAU, **PLXNA1**, PLXNB1, **PLXNC1**, **PTGS2**, PTK2, RAB17, RAB3A, RAB3B, RAB6A, RHOA, RPS15, **RUNX2**, SEMA3A, SEMA3C, **SEMA3D**, SEMA3E, SEMA4A, SEMA4C, SEMA4D, SEMA4F, SEMA5A, **SEMA5B**, SEMA6B, SEMA6C, **SEMA7A**, SERPINE1, **SLC2A1**, **SNCA**, **SPP1**, **SQSTM1**, TCEAL1, **TIMP1**, **TIMP2**, TIMP3, TLN1/TLN2, **TNF**, **TNFRSF11B**, **TNFSF11**, **TP53**, TUBA1B, TUBA1C/TUBA3C/TUBA3D/TUBA4A, **UVRAG**, VIM, YAP1

The most commonly investigated 10 genes or metabolites in these studies were (in descending order): runt-related transcription factor 2 (*RUNX2*), alkaline phosphatase (*ALPP;* also known as ALP), bone gamma-carboxy glutamic acid-containing protein (*BGLAP*; also known as osteocalcin), interleukin 1β (*IL1B)*, prostaglandin-endoperoxide synthase 2 (*PTGS2*; also known as *COX2*), tumor necrosis factor-alpha receptor superfamily member 11B (*TNFRSF11B*; also known as osteoprotegerin, OPG), TNF superfamily member 11 (*TNFSF11*; also known as RANKL), collagen Iα1 (*COL1A1)*, prostaglandin E_2_ (PGE_2_) and Osterix (*SP7*; identical with *OSX*) (Supplementary Table S6). Their expression profiles and the corresponding force-related information were summarized in Supplementary Table S7.

### Gene List and Protein-Protein Interaction Network Analysis

Gene list analysis was done as described (Figure 3B). The gene list compiled from studies on dynamic forces contained 206 genes, proteins or analytes, of which 147 (∼71.4%) were identified as differential expressed genes (DEG) as defined above. Of 115 entries from the gene list related to studies applying static forces, 56 (∼48.7%) were identified as DEGs (Table 3).

**FIGURE 5:**
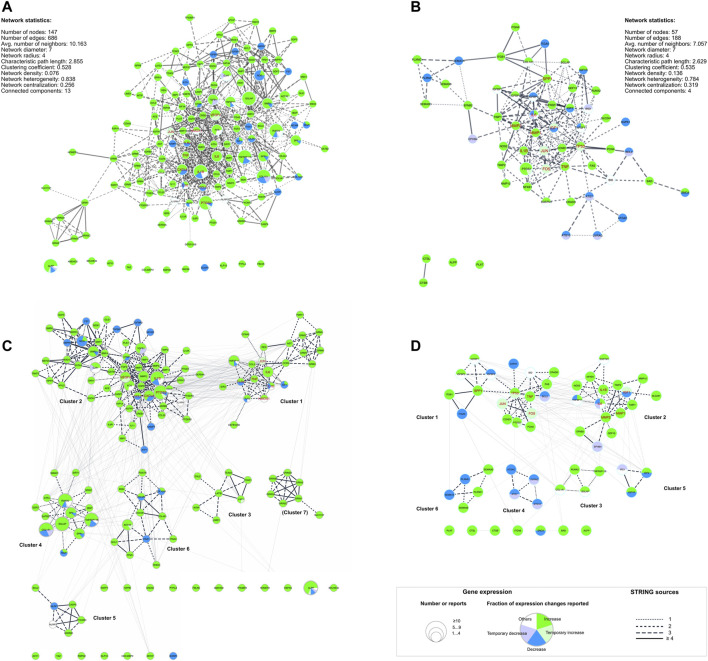
Dynamic **(A, C)** and static **(B, C)** protein-protein-interaction (PPI) networks generated using differential expressed genes (DEG) lists (Table 4). **(A, B)** PPI networks including basic network statistics for each were generated using STRING-DB. **(C, D)** show the same networks as in **(A, B)** but herein, the identified subnetworks are emphasized and labeled (Table 4, Supplementary Table S8). The legend applies to all four networks: The node size corresponds to the number of reports identified for the respective underlying gene. The edges’ line style depicts the number of STRING sources for the given connection. Additionally, hub genes identified with *cytoHubba* (Chin et al., 2014) were colored red (Table 5). For the complete networks **(A, B)** and each of the subnetworks **(C, D)** pathway analysis was applied (Supplementary Table S8).

Protein-protein interaction (PPI) networks were generated using both gene lists with *STRING-DB* as described, and network statistics for both networks were calculated (Figure 5). For each gene node shown in Figure 5 the number of studies (node size) and expression pattern(s) were depicted: most of the genes included in the “dynamic” network showed (Figure 5A) an upregulation in gene expression after dynamic tension application. Some genes were downregulated only (e.g. *ATF1*, *BMPR2*, *TGFBR1* and *TGFBR2*) whereas a few genetic loci were reported to be either up- or down regulated (e.g. *ALPP*, *COL1A1*, *CXCL8*, *IL1B*, or *IL10*). In contrast, gene expression of the majority of genes included in the “static” PPI network was either up- or downregulated depending on the particular study (Figure 5B). Up- and downregulation was reported for three genes (*IL6*, *IGF*, *TNFSF11*) by different studies. Interestingly, 12 genes from the “dynamic” DEG list (Figure 5A) and 4 genes from the “static” DEG list (Figure 5B) were not included in their particular PPI networks (Table 4) including *ALPP*, which is one of the 10 most frequently investigated genes or metabolites here.

**TABLE 4:** Differential expressed genes (DEGs) from the “dynamic” and “static” gene lists and their affiliation to the “dynamic” and “static” protein-protein interaction networks and one of the MCODE clusters. The corresponding networks and clusters were depicted in Figure 5.

Gene list	Genes in network	MCODE clusters		
		Cluster number	Number of genes (n) and genes in cluster	
„Dynamic“	Genes in the “dynamic” PPI network	#1	(22)	*AGT, CCL3, CCNA2, CCR5, DEFB103B, GRM2, GRM3, GRM4, GRM5, GRM6, HMOX1, IER3, IGF1, IL10, IL6, JUN, P2RY1, REN, TLR2, TLR4, TNF, TNFSF11*
	*ACE, ACTA2, ADRB2, AGT, AGTR1, ATF1, ATF4, BCL2, BGLAP, BGN, BMP2, BMP4, BMP6, BMP7, BMPR1A, BMPR2, CASP3, CASP5, CCL2, CCL20, CCL3, CCL5, CCNA2, CCR5, COL12A1, COL1A1, COL3A1, COL4A1, COL5A1, CREB1, CTGF, CXCL8, DEFB103B, DEFB4A, DKK1, DVL2, EGFR, FGF2, FOS, FST, GATA4, GDF2, GDF5, GJA1, GLI2, GRIA3, GRIN1, GRIN2C, GRIN2D, GRIN3A, GRIN3B, GRM2, GRM3, GRM4, GRM5, GRM6, GSDMD, HIF1A, HMOX1, HOMER1, HSPA5, IBSP, IER3, IGF1, IL10, IL11, IL12A, IL1B, IL1RN, IL6, IL6R, ITGA1, ITGA3, JUN, LATS1, LIMD1, MCAM, MEF2C, MGP, MMP1, MMP14, MMP2, MMP3, MSX1, MSX2, MYH7, MYL2, MYL7, NAMPT, NKX2-5, NLRP1, NLRP3, NOG, NPPA, NPPB, P2RY1, PLAT, POSTN, PTGER2, PTGER4, PTGS1, PTGS2, PYCARD, REN, RHOA, ROCK1, RUNX2, RXFP1, SATB2, SIRT1, SLC17A7, SMAD7, SP7, SPP1, SPRY2, SQSTM1, TEAD1, TEAD2, TGFB1, TGFBR1, TGFBR2, TIMP1, TIMP2, TLR2, TLR4, TNF, TNFRSF11B, TNFSF11, TNNT2, TP53BP2, TPM1, VEGFA, WTIP, XBP1, YAP1*	#2	(61)	*ACE, ADRB2, AGTR1, ATF1, BMP2, BMP4, BMP6, BMP7, BMPR1A, BMPR2, CASP3, CCL2, CCL20, CCL5, CREB1, CTGF, CXCL8, DEFB4A, DKK1, EGFR, FGF2, FOS, FST, GATA4, GDF5, GJA1, GLI2, HIF1A, IL11, IL12A, IL1B, IL1RN, IL6R, MCAM, MEF2C, MMP1, MMP14, MMP2, MMP3, MSX1, MYL2, NAMPT, NKX2-5, NOG, NPPA, PLAT, PTGER2, PTGER4, PTGS1, PTGS2, ROCK1, SPRY2, SQSTM1, TGFB1, TGFBR1, TGFBR2, TIMP1, TIMP2, TNNT2, VEGFA, XBP1*
	Genes outside the “dynamic” PPI network	#3	(7)	*DVL2, LATS1, LIMD1, TEAD1, TEAD2, WTIP, YAP1*
	*ACY1, ALPP, AMDHD2, BHLHE40, CDC42EPS, FBLN5, GOSR1, KLF10, PTPLA, RSPO2, TAZ, UNC50*	#4	(14)	*ATF4, BGLAP, COL1A1, GDF2, IBSP, MGP, MSX2, RUNX2, SATB2, SIRT1, SMAD7, SP7, SPP1, TNFRSF11B*
		#5	(6)	*BCL2, CASP5, GSDMD, NLRP1, NLRP3, PYCARD*
		#6	(12)	*ACTA2, BGN, COL12A1, COL3A1, COL4A1, COL5A1, ITGA1, ITGA3, MYL7, POSTN, RHOA, TPM1*
		#7	(7)	*GRIA3, GRIN1, GRIN2C, GRIN2D, GRIN3A, GRIN3B, SLC17A7*
„Static”	Genes in the “static” PPI network	#1	(18)	*BCL2, BID, CCND1, CRADD, DAPK1, FAS, FOS, IGFBP1, IGFBP3, IGFBP5, ITGA5, ITGB1, JUN, PCNA, PTGS2, SPP1, TNF, TP53*
	*ATG10, ATG4C, ATG7, BAD, BCL2, BGLAP, BID, CCND1, COL1A1, CRADD, DAPK1, EFNB2, EPHB4, FAS, FOS, GDF15, IGF1, IGF1R, IGF2, IGFBP1, IGFBP3, IGFBP5, IL1B, IL6, IRS1, ITGA5, ITGA6, ITGB1, JUN, MMP1, MMP12, MMP2, NFKB1, NOS2, PCNA, PLXNA1, PLXNC1, PTGS2, RUNX2, SEMA3D, SEMA5B, SEMA7A, SLC2A1, SNCA, SPP1, SQSTM1, TIMP1, TIMP2, TNF, TNFRSF11B, TNFSF11, TP53, UVRAG*	#2	(16)	*EFNB2, EPHB4, GDF15, IGF1, IL1B, IL6, MMP1, MMP12, MMP2, NFKB1, NOS2, SLC2A1, SQSTM1, TIMP1, TIMP2, TNFSF11*
		#3	(4)	*BGLAP, COL1A1, RUNX2, TNFRSF11B*
		#4	(4)	*ATG10, ATG4C, ATG7, UVRAG*
	Genes outside the “static” PPI network	#5	(3)	*IGF1R, IGF2, IRS1*
	*ALPP, CTSB, CTSL, PLAT*	#6	(5)	*PLXNA1, PLXNC1, SEMA3D, SEMA5B, SEMA7A*

Biological processes and signaling pathways involved in the regulation of gene expression after dynamic and static tension application on hPDLCs were analyzed querying the “Biological Process” subsection of GeneOntology (GO) and GeneAnalytics’ SuperPathway database. The top 10 enriched terms from the “dynamic” and “static” DEG lists were ranked according to log_10_(FDR) for GO or the SuperPathway’s score, respectively. Only GO terms containing at least 5% of the genes from the particular DEG list (ratio ≥0.05) were considered (Supplementary Table S8).

*Dynamic DEG list.* The most significant GO terms describing biological processes were related to “*responses to endogenous stimuli*” (ratio: 0.05/log_10_(FDR) = 36.16; 0.05/30.14), the action of growth factors (0.09/30.14; 0.09/28.91), ossification (0.15/29.10) or differentiation (0.06/27.36) and motility/movement (0.06/27.80; 0.06/27.77; 0.06/27.54) (Supplementary Table S8.1). SuperPathways analysis revealed high scores of the more general ERK (ratio: 0.05/score: 115.97), Akt (0.06/96.02), and PAK (0.06/95.94) signaling pathways (Supplementary Table S8.2). Lower scores but higher ratios were attributed to “*Interleukin-4 and 13 signaling*” (0.18/70.16) and the “*Hippo signaling pathway*” (0.14/66.29). Nevertheless, “*Lung Fibrosis*” SuperPathway (0.27/70.05) was also part of the “top 10 list” of enriched terms.

*Static DEG list.* The most significant GO terms describing biological processes driven by genes from the “static” DEG list were ossification (0.06/10.08), responses to mechanical stimuli (0.05/8.97; 0.10/8.40), regulation by glucocorticoids (0.06/8.91; 0.07/7.99), positive regulation of small molecule metabolic processes (0.07/8.21), and apoptotic signaling pathways (0.09/7.88; 0.19/7.54) (Supplementary Table S8.3). SuperPathways related to apoptosis (0.15/76.37) and autophagy (0.07/62.14; 0.03/53.00) but also to ERK signaling (0.02/62.14), cell adhesion/ECM remodeling (0.20/61.73) and interleukin-4 and 13 signaling (0.11/56.77) were significantly enriched (Supplementary Table S8.4 in Supplementary Table S8).

To identify highly connected gene clusters within each network, MCODE clustering was performed. Seven different clusters were identified in the dynamic PPI network (Figure 5C; Table 4) and six clusters in the static PPI network (Figure 5D; Table 4). Cluster #7 of the “dynamic” network consisted of seven genes, which were included in this DEG list based on one study only (Fujihara et al., 2010) (Figure 5C). This cluster was not further analyzed. All other clusters were re-analyzed concerning GO/Biological Process terms and SuperPathway enrichment (Supplementary Table S8). The same ordering and ratio cut-off were applied as above.

Generally, the identified clusters showed a higher ratio of included genes than the whole network (Supplementary Table S8) independent of the database used. For example, cluster #1 from the dynamic network was described significantly as “*adenylate cyclase-inhibiting G protein-coupled glutamate receptor signaling pathway*”-related (0.56/8.97). Cluster #3 from the same network was described significantly by the “*ionotropic glutamate receptor signaling pathway*” (0.24/14.10), but also by “*excitatory chemical synaptic transmission*” (0.38/6.97), whereas cluster #4 was dominated by terms related to bone mineralization and remodeling, e.g. “*osteoblast differentiation*” (0.10/12.50) and development (0.22/6.80), or “*regulation of bone resorption*” (0.08/4.09). In general, an increase in specificity (i.e. higher ratio) was also observed for SuperPathways enrichment (Supplementary Tables S8.2,S8.4 in Supplementary Table S8).

Essential nodes (i.e. hub genes) in both networks were identified with *cytoHubba*. Within the network derived from the dynamic DEG list, eleven hub genes were identified (Table 5: *IL6*, *CXCL8*, *IL1B*, *TNF*, *CCL2*, *IL10*, *VEGFA*, *HMOX1*, *JUN*, *PTGS2*, *TLR4*; red labeled nodes in Figures 5A,C). Altogether, ten hub genes were identified in the network generated from the static DEG list (Table 5: *IL6*, *IGF1*, *TP53*, *TNF*, *MMP2*, *JUN*, *FOS*, *IL1B*, *MMP1*, *SPP1*; red labeled nodes in Figures 5B,D). In both networks, the hub genes were either located in cluster #1 or #2 of the particular network.

**TABLE 5:** Top hub genes identified in both STRING networks derived from the “dynamic” (*n* = 147) and “static” (*n* = 57) tension DEG lists. Different score measures were calculated by *cytoHubba* (Chin et al., 2014). The total score cut-off was set to ≥ 2*mean total score of each network being 361511.0 for the “dynamic” network and 1209.0 for the “static” network. In both lists the hub genes were sorted in descending order according to the total score. These hub genes were colored red in Figure 5.

Gene list	Gene	MCODE cluster #	Local-based methods				Global-based methods							Total score (SUMscore)
			MCC	DMNC	MNC	Degree	EPC	BottleNeck	EcCentricity	Closeness	Radiality	Betweenness	Stress	
Dynamic	*IL6*	1	4949248	0.44127	43	43	55.7020	5	0.22959	84.50000	5.67469	2127.04962	18,402	4970014.6
	*CXCL8*	2	4946706	0.54262	33	33	54.6030	1	0.22959	76.00000	5.47594	1435.65143	12,426	4960771.5
	*IL1B*	2	4880486	0.43410	36	36	54.2840	8	0.22959	77.91667	5.48964	1563.68358	12,042	4894310.0
	*TNF*	1	4859699	0.53877	29	30	53.6020	7	0.22959	74.83333	5.44852	757.28180	5478	4866134.9
	*CCL2*	2	4704534	0.61776	25	25	52.0030	5	0.22959	71.41667	5.37313	192.83836	1884	4706795.5
	*IL10*	1	4544888	0.61015	23	23	51.3110	2	0.22959	69.33333	5.31145	221.65300	2056	4547340.4
	*VEGFA*	2	4450715	0.40116	43	44	55.6160	30	0.22959	85.33333	5.69525	2581.50569	18,952	4472512.8
	*HMOX1*	1	4357032	0.72691	16	16	47.4180	1	0.22959	65.16667	5.22921	61.59565	766	4358011.4
	*JUN*	1	4269668	0.63037	25	27	53.6160	3	0.22959	73.58333	5.44167	750.77011	5418	4276025.3
	*PTGS2*	2	4228086	0.63871	21	21	51.0090	5	0.22959	68.91667	5.32516	421.56904	3358	4232038.7
	*TLR4*	1	4198470	0.67581	18	18	48.3580	1	0.22959	67.25000	5.29089	124.59022	1334	4200087.4
Static	*IL6*	2	6008	0.44392	22	22	25.7170	7	0.23246	35.08333	5.88293	263.47537	1206	7595.8
	*IGF1*	2	6088	0.49129	20	20	25.6770	8	0.23246	33.91667	5.82928	174.76358	868	7244.9
	*TP53*	1	2970	0.32783	21	23	25.2950	21	0.23246	36.16667	5.97233	914.98502	2848	6866.0
	*TNF*	1	4527	0.46144	17	18	24.7640	8	0.23246	32.83333	5.79352	304.73126	1120	6058.8
	*MMP2*	2	1598	0.54053	14	16	24.5630	10	0.23246	32.75000	5.86505	581.49581	2310	4593.4
	*JUN*	1	3792	0.61467	12	12	23.5690	1	0.18596	28.90000	5.52530	22.03107	146	4043.8
	*FOS*	1	3720	0.69834	10	10	23.1720	1	0.18596	27.90000	5.48954	6.50124	60	3864.9
	*IL1B*	2	2246	0.48461	16	16	24.8150	1	0.23246	31.41667	5.70412	109.01102	574	3024.7
	*MMP1*	2	2286	0.60003	12	12	23.9330	1	0.23246	29.58333	5.65047	31.57875	252	2654.6
	*SPP1*	1	1134	0.49815	13	15	23.9390	4	0.23246	31.75000	5.79352	296.75685	1106	2631.0

Abbreviations: MCC, Maximal Clique Centrality; DMNC, Density of Maximum Neighborhood Component; MNC, Maximum Neighborhood Component; EPC, Edge Percolated Component.

The expression of five of eleven hub genes (*VEGFA*, *JUN*, *TNF*, *IL6*, *HMOX1*) included into the “dynamic” PPI network was upregulated by dynamic tension forces. For the remaining six hub genes both up- and downregulation of gene expression was reported. For eight out of ten hub genes (*SPP1*, *JUN*, *TP53*, *TNF*, *FOS*, *IL1B*, *MMP1* and *MMP2*) upregulated expression was observed after exposure to static tension forces, and for the remaining two genetic loci (*IL6* and *IGF1*) both up- and downregulated expression was reported.

## Discussion

This systematic review aimed to identify and analyze studies applying tension forces to human periodontal ligament cells (hPDLCs) and to delineate the impact of different force parameters on the expression of relevant genes. Risk of bias assessment was conducted by application of published criteria using clear definitions. The commonly investigated genes, proteins, and metabolites were summarized and analyzed by gene enrichment and pathway analysis.

### Commonly Used Force Apparatuses

To apply tension type of force on hPDLCs *in vitro* three different major groups of apparatuses were identified here: 1) commercially available systems. i.e. the Flexcell® Tension System or the STREX® Cell Stretching System, 2) self-designed apparatuses using commercially available components (e.g. Bioflex® or Uniflex® plates, petriPERM® or Lumox® dishes) as a central part of a stretching device, and 3) solely self-constructed apparatuses. Irrespective of the particular design, cells were grown on a flexible surface undergoing cyclic (or static) equibiaxial or uniaxial stretching in all devices.

According to this review, the Flexcell® Tension System was the most widely adopted apparatus for equibiaxial tension using Bioflex®, and uniaxial tension using Uniflex® cell culture plates. In various reports the mechanical characteristics of these elastic membranes used to apply tension to adherent cells have been studied and limitations of this method have been reported. Specifically, several studies reported a heterogeneous strain distribution within the surface used for cell cultivation and a considerable interference of other types of force (e.g. compression and shear stress) (Gilbert et al., 1994; Vande Geest et al., 2004; Matheson et al., 2006). In the Flexcell® Tension system using Bioflex® plates without a biaxial loading post, equibiaxial strain was mostly focused in the center of the membrane, whereas almost pure uniaxial strain was found at the rigid rim of the well (Gilbert et al., 1994). We consider this setup to be similar to several self-designed apparatuses using Bioflex^®^ plates. As such, we propose, that the heterogeneous strain distribution might also apply to these setups. In contrast, in Flexcell Tension systems using Bioflex® plates with a “*biaxial loading post*” the constant biaxial strain region was located in the membrane area on the post, whereas off-post large radial strain was produced (Vande Geest et al., 2004). Application of uniaxial strain with Flexcell’s Uniflex® culture plates using an uniaxial loading post results in almost uniform strain distribution in the membrane area on-post (Matheson et al., 2006). Irregular strain distribution might occur, anyhow, comparing longitudinal and transverse orientation together with a certain amount of compressive strain (Matheson et al., 2006). As a consequence of the heterogenous strain distribution, only some of the cells receive the desired mechanical stimulation (Matsuda et al., 1998) and thus gene expression reflects the average of all cells in the examined area. Irrespective of the not entirely standardized strain parameters, the tension systems identified herein have proven to be efficient models to mimic the *in vivo* mechanical microenvironment to investigate the related biological reactions on the cellular level. In 2017, the “BioFlex® Cell Seeder” (Flexcell Inc.) was introduced to the market to be used in combination with the BioFlex cell culture plates. Limiting cell growth within a defined area providing uniform strain distribution increases the reproducibility of cell seeding and exposure against mechanical cues. However, none of the included studies reported the application of this device.

The PDL comprises different types of collagen, including type I (about 80%), type III (15%), type V, type VI, and type XII collagen (Berkovitz et al., 2009, pp. 179–180). Most frequently BioFlex plates pre-coated with collagen type I were used, but pronectin coating was also reported (Jacobs et al., 2013). Other silicone-based membranes were used together with coatings of gelatin (Tantilertanant et al., 2019a; b) or collagen type I and/or fibronectin (Konstantonis et al., 2014; Papadopoulou et al., 2019). Further studies did not report any coating, particularly when using PetriPerm or Lumox dishes. Diercke et al. (2011) applied a combination of collagen type-I and fibronectin to coat the PetriPerm dishes. Commonly coatings were applied to increase the biocompatibility of the membrane surface.

Most recently, three-dimensional (3D) cell culture studies have reached increasing significance since they allow for a better simulation of the cells’ extracellular matrix (ECM) and *in vivo* microenvironment. Providing structural support and signal transduction to the cells, ECM is involved in various biological processes, including cell migration, proliferation, differentiation and intercellular communication (Dieterle et al., 2021). For simulation matrices made from collagen, polylactic-co-glycolic acid (PLGA) and hydrophilically modified poly-L-lactide (PLLA) are used in cytomechanics (Yang et al., 2015; Janjic et al., 2018). Though 2D coating resembles the *in vivo* situation more than tissue culture plastic alone, differences in porosity, microarchitecture and local rigidity in comparison to 3D substrates effect cell migration (Doyle et al., 2015) and mechanotransduction (Yang et al., 2015).

Interestingly, only two studies reported on the effects of tension force applied to cells within 3D scaffolds (Von den Hoff, 2003; Ku et al., 2009). The first one focused on the assessment of tension force exerted to the substrate by the cells (Von den Hoff, 2003). With only one qualified study (Ku et al., 2009) a comparison between 2D and 3D cell culture setups seemed not conclusive. Yang et al. (2015) emphasized that 3D cell culture techniques are yet not standardized and suggested to establish tissue specific scaffolds along with the identification of appropriate cell densities. The insufficient knowledge on 3D scaffolds might explain its infrequent use to study the effect of tension force on hPDLCs.

### Rationale for Force Parameters

Tension type of force was applied either statically or dynamically on hPDLCs. As such, the selection of the relevant model parameters (cell type, type of force and its duration, magnitude and frequency) was mostly based on the purpose of the specific study, being either the simulation of a clinical situation (occlusal forces or OTM), or to investigate the force-related expression of specific genes, group of genes or pathways. Due to the general objectives of the studies analyzed herein, the selection of cell type and type of force seems plausible. The remaining parameters were selected according to: 1) *in vivo* evidence from animal models simulating OTM or measurement of bite force in human subjects (e.g. He et al., 2004; Fujihara et al., 2010; Li et al., 2013; Li et al., 2014; Ren et al., 2015; Tantilertanant et al., 2019a); 2) finite element analysis to study the biomechanical behaviour of the PDL (e.g. Howard et al., 1998; Kletsas et al., 2002; Konstantonis et al., 2014; Chen et al., 2015); 3) *in vitro* studies defining an “optimal” window to study the expression of specific genes or pathways (e.g. Basdra et al., 1996; Hao et al., 2009; Huelter-Hassler et al., 2017; Hülter-Hassler et al., 2017).

*Force duration*: Continuous exposure to a stretching force is considered as an appropriate surrogate for *in vivo* forces applied by fixed appliances during OTM (Ziegler et al., 2010; Steinberg et al., 2011). As such, time intervals for application were chosen reflecting different stages in OTM or other clinically relevant conditions (Ziegler et al., 2010; Goto et al., 2011; Steinberg et al., 2011). According to our results, the maximum force duration varied between 0.5 h and 15 days. The most commonly used force duration in dynamic tension experiments were either 48 h or 72 h, and static tension was most commonly applied for 12 h. Under experimental *in vitro* conditions the maximum application time is, in fact, limited by the feeding intervals of the cells and the apparatus used. With static tension, cell proliferation should be considered as an influencing factor – especially during long-term force application. Cells undergoing cell division might transiently loose contact to the stretched surface. After reattachment to the already deformed surface they might not be further subjected to stretching force.

*Force magnitude*: Similar to force duration, force magnitude is a relevant experimental parameter for simulation of both, dynamic and static tension forces. Based on the studies considered herein, force magnitude selection was again mostly related to the objective of the study: 1) expression of a specific gene in response to tension force application allowing to define the dynamic range along with the range of optimum forces (e.g. Long et al., 2002; Agarwal et al., 2003), and 2) to simulate a clinical situation (e.g. Long et al., 2002; Li et al., 2014). In most studies, 10% dynamic tension was applied to mimic the physiologic conditions of occlusal force or OTM (e.g. Fujihara et al., 2010; Li et al., 2014; Ren et al., 2015). This force magnitude was based on *in vivo* studies, which either focused on tissue remodeling and tooth movement after exposure to different levels of orthodontic force (King et al., 1991; Gonzales et al., 2008), or studies on tooth mobility in response to different force levels (Mühlemann, 1954; Mühlemann and Zander, 1954). Several studies (Howard et al., 1998; Kletsas et al., 2002; Konstantonis et al., 2014; Chen et al., 2015) took results from finite element analysis into consideration to select the force magnitude best corresponding with the real clinical situation (Andersen et al., 1991; Natali et al., 2004; Dong-Xu et al., 2011).

*Force frequency*: To apply dynamic tension different frequencies were adopted. Selection of appropriate force frequencies mostly relied on two different rationales: 1) experience from previous *in vitro* studies either using similar setups or defining appropriate frequency ranges to study the expression of specific genes (Yamaguchi et al., 1994; Long et al., 2002; Ma et al., 2015; e.g.; Ren et al., 2015; Memmert et al., 2020), and 2) deduction from the real clinical situation, e.g. tooth contact rates during sleep (He et al., 2004) or an average masticatory cycle (Tantilertanant et al., 2019a). In this context various estimates of contact rates were reported so far, e.g. contacts ranging from 17.2 to 104.3 contacts/h (Yamashita et al., 1993), a mean frequency of masticatory cycle of ∼70 rpm (Pini et al., 2002), or 1.5–2 Hz (Woda et al., 2006).

According to our review, the most commonly used frequency was 0.1 Hz for dynamic equibiaxial tension and 0.5 Hz for dynamic uniaxial tension. Although the real frequency of tension applied on the PDL cells during OTM yet remains unknown (Long et al., 2002; Padial-Molina et al., 2013; Wang Y. et al., 2019), the clinical and technical evidence summarized in this review will provide clues for design of related experiments.

### Most Frequently Investigated Genes, Proteins and Metabolites

To identify the most relevant mechanical responses during OTM, we focused on the top 10 most frequently investigated genes, proteins and metabolites: *RUNX2*, *ALPP, BGLAP*, *IL1B*, *PTG2*, *TNFRSF11B*, *TNFSF11*, *COL1A1*, PGE_2_ and *SP7* (Supplementary Table S6). According to their functional contribution we grouped these genes into three categories: genes related to osteogenesis (*RUNX2*, *SP7, ALPP*, *BGLAP, COL1A1*), osteoclastogenesis (*TNFRSF11B*, *TNFSF11*), and inflammation (*IL1B*, *PTGS2*, PGE_2_).

#### Osteogenesis

*RUNX2* regulation is an integral and central part in the development and remodeling not only of osseous tissue but also of the periodontal ligament (Ziros et al., 2008). PDL cells are capable of differentiation into osteoblasts or cementoblasts in response to mechanical stimulation (Ziros et al., 2002). *RUNX2* upregulation was reported in 19/26 (73%) studies using either RT-qPCR or both RT-qPCR and western blotting. Decrease, temporary changes or other types of regulation were found in the remaining studies (7/16, ∼27%). Although several studies revealed partially contradictory results, it was well-supported that *RUNX2* expression increased within the first 12 h of tension application.

Another essential transcription factor in the osteogenic pathway acting downstream of RUNX2 is SP7 (also known as Osterix), which belongs to the zinc finger-containing transcription factor SP family (Tang et al., 2012; Li et al., 2013; Li et al., 2015). Significant gene and/or protein upregulation of *SP7* in response to tension was reported in 9/10 (90%) of the relevant studies, whereas only one study reported downregulation of gene expression and temporary upregulation of the corresponding protein (Li et al., 2013). The authors concluded, that this difference might be due to complex regulation mechanisms and modifications occurring during its transcription and translation (Li et al., 2013).

Alkaline phosphatase (ALPP) also plays a crucial role in the initiation of osteogenic differentiation and bone remodeling (Chen et al., 2014). In all of the 24 relevant studies, *ALPP* gene expression was determined using either semiquantitative or quantitative PCR. ALPP protein was quantified in cell lysates using western blots, ELISA or enzyme activity assays. Significant upregulation in response to tension <12% was reported in 17/24 (71%) of the studies, mostly during the first 12 h of force application.

As the most abundant non-collagenous bone-matrix protein, bone gamma-carboxy glutamic acid-containing protein (BGLAP; osteocalcin) is a late marker of osteoblast differentiation and mineralization (Chen et al., 2014). Significant upregulation of BGLAP in response to tension was reported in 16/18 (89%) of the studies. In contrast, one study reported a slight decrease in *BGLAP* gene expression and it was assumed that an enhanced cell proliferation of young osteoblasts might be responsible (Jacobs et al., 2013). Another study described a transient upregulation of BGLAP following 3 h of exposure against tension force (Qin and Hua, 2016).

The extracellular matrix (ECM) of the periodontal ligament mainly consists of fibrillar collagens, among which type I collagen accounts for ∼75% (Kaku and Yamauchi, 2014). The latter is composed of alpha-1 (COL1A1) and alpha-2 (COL1A2) type I collagen chains. COL1A1 is confirmed to be essential for bone remodeling and osteoblastic differentiation in response to tension during OTM (Birkedal-Hansen et al., 1977; Jacobs et al., 2013). Significant upregulation of protein expression was found in 8/13 (62%) of related studies after exposure to ≤12% of tension. Contradicting expression patterns have been reported, which might be due to the heterogeneity of the PDL cells used, digestion of COL1A1 by MMP1 (Nemoto et al., 2010), or the inhibitory effect of IL1β and TNFα on *COL1A1* gene expression (Sun et al., 2017). Although inconsistencies were partially found, upregulation of *COL1A1* in response to tension was confirmed in the majority of the studies considered here.

#### Osteoclastogenesis

Bone remodeling is primarily regulated by a closely interrelated system of receptors and mediators including TNFSF11 (receptor activator of nuclear factor kappa ligand; RANKL), its cellular receptor, receptor activator of NF-kappaB (RANK), and TNFRSF11B (osteoprotegerin, OPG) ultimately maintaining the balance between osteogenesis and osteoclastogenesis (Krishnan et al., 2015). As a decoy receptor for RANKL, OPG suppresses the binding between RANKL and RANK and thus inhibits osteoclastogenesis and bone resorption (Liao and Hua, 2013).

Upregulation of OPG gene expression and/or protein synthesis was reported in 11/14 (79%) of the included studies. Due to the heterogeneity of force parameters, the correlation between gene expression, force duration and/or force magnitude can only partly be defined. The regulation of RANKL expression showed large variability in comparison to OPG: only 57% (8/14) of the relevant studies reported an upregulation of both gene and protein expression after force application, whereas the remaining studies showed inconsistent expression patterns.

It is commonly accepted, that an increased RANKL/OPG ratio favours osteoclastogenesis, meaning an upregulation of RANKL in parallel with a downregulation/lower induction of OPG (Boyce and Xing, 2007). The RANKL/OPG ratio was reported in three studies included into this review (Nogueira et al., 2014b; Konstantonis et al., 2014; Jacobs et al., 2015). Two of these studies identified a decreasing RANKL/OPG ratio compatible with a more intensive bone formation after exposure to minor tension forces (≤10%): a temporary decrease of the RANKL/OPG ratio was found by Jacobs et al. (2015) using RT-qPCR, whereas Liao and Hua (2013) reported an increasing OPG/RANKL ratio for both, gene and protein expression. Higher tension forces (20%) were reported to induce an increased RANKL/OPG ratio at both gene and protein levels indicating net bone resorption (Nogueira et al., 2014b).

#### Inflammation

The response of the PDL to mechanical stress has been characterized as an aseptic transitory inflammatory process, which is regulated by various mediators, including cytokines and chemokines (Lee et al., 2012). Interleukin 1β (IL1B) is an upstream cytokine involved in many inflammatory processes (Lee et al., 2012) and in osteoclast formation, differentiation and activation (Long et al., 2001). Upregulation was reported in 75% (12/16) of the relevant studies depending on the particular duration and magnitude of force application. A reduced expression or inconsistent expression patterns during exposure to forces of various magnitudes and durations were reported in the remaining 4/16 (25%) studies. These differences have been mainly attributed to the particular magnitude of tension: a lower magnitude attenuates the inflammatory response, while a higher one elicits inflammation (Long et al., 2001).

Prostaglandin-endoperoxide synthase 2 (PTGS2; also known as COX2) is known as a key regulator enzyme of the eicosanoid biosynthesis pathway and is thus also involved in prostaglandin E_2_ (PGE_2_) synthesis (Nogueira et al., 2014b). Amongst others, the activity of PTGS2 and the synthesis of PGE_2_ is particularly amplified by pro-inflammatory stimuli including IL1B (Nogueira et al., 2014b). PGE_2_ mediates bone resorption under physiological and pathological conditions, and is centrally involved in both, the response of periodontal tissue to mechanical stress and the pathogenesis of periodontitis (Shimizu et al., 1998). Herein, *PTGS2* gene expression and PGE_2_ synthesis were reported in 14 and 12 of the included studies, respectively. Of these studies, 9 focused on *PTGS2* and PGE_2_, among which 8/9 studies (∼89%) found an increasing transcription of the *PTGS2* gene and/or PGE_2_ concentration after mechanical stimulation correlating with force duration and force magnitude. The data of the remaining studies revealed mixed expression patterns of *PTGS2* and PGE_2_ activity after force exposure, which might be attributable to anti-inflammatory effects of lower tension forces (Long et al., 2002). Taken together *PTGS2* expression and PGE_2_ production is induced by the exposure of cell cultures to tensions forces and it is positively correlated to force duration and force magnitude.

#### Reasons for Heterogeneity of Genes/Proteins/Metabolites Regulation

Considering the list of the most commonly considered genes, inconsistencies between gene and protein expression were found in several reports. The observed non-proportional relationship between gene expression and protein activity can be attributed to the time lag between transcription and translation. This time lag might be prolonged by post-transcriptional processing and degradation of the transcripts, as well as post-translational modifications like phosphorylations (e.g. Li et al., 2013; Ren et al., 2015) or proteolytic cleavage (e.g. Wang et al., 2013; Zhuang et al., 2019). The experimental heterogeneity identified among different studies reporting force-related expression of the same genes can be attributed to: 1) donor-related issues (e.g. age of donor), 2) hPDLC isolation-related issues, 3) cell culture of hPDLCs (e.g. cell culture medium, passage number), 4) reference gene selection in (s)qPCR experiments, and 5) heterogeneity of force parameters. In addition, the pooling of cells from different donors (Stoddart et al., 2012), the seeding density of cells and thus the amount of confluency and the different cell culture media used might have caused heterogeneity of results.

All studies included herein isolated hPDLCs from teeth that have been removed due to orthodontic reasons. When considering all studies the age of donors ranged from 8 to 40 years, but within each study the donors had the same age. This is even more important, since the phenotype of hPDLCs, specifically the proliferation rate, osteogenic potential or *in vitro* life span clearly depends on the age of the donor (Marchesan et al., 2011). Moreover, force-related gene expression has been reported to be significantly dependent on the age of the donor (Mayahara et al., 2007). Accordingly, many studies reported considerable functional inconsistencies between different cell samples most likely caused by the biological heterogeneity among different donors (Monnouchi et al., 2011; Yuda et al., 2015; Papadopoulou et al., 2017; Arima et al., 2019). For experimental simulation of the age, several studies focusing on cellular senescence simply used different passage numbers, supposing a correlation between the passage number and the age. Thus, they designated passage numbers ranging from 3 to 7 as “*young*” and those ranging from 18 to 24 as “*old*” or “*senescent*” (Shimizu et al., 1997; Abiko et al., 1998; Ohzeki et al., 1999; Miura et al., 2000; Konstantonis et al., 2014). Generally, passage numbers of hPDLCs used in the included studies ranged between 2 and 15 (Supplementary Table S2). A maximum passage number of 20 was reported in a study using limited dilution cloning (Long et al., 2001). Differences in morphology and biological activity between early and late passages were reported, with the early passages resembling fibroblasts characteristic of original tissue more closely (Marchesan et al., 2011). Therefore, the use of early passage (passage ≤7) of primary culture is recommended to maintain most of the original cell phenotype (Marchesan et al., 2011).

Exclusively all studies analyzed herein applied tension force to human periodontal ligament cells (hPDLCs). These cells were commonly isolated from the middle third of the roots from teeth removed due to mostly orthodontic reasons using two different cell isolation techniques, i.e. the “explant” (Brunette et al., 1976; Somerman et al., 1988) or the “digestion” technique (Brunette et al., 1976; Seo et al., 2004). Different terms and abbreviations were used to identify these cells, including “hPDLF”, hPDL-fibroblasts”, “hPDL fibroblasts”, “hPDLC”, “hPDLCs”, “hPDL cells”, “hPDLSCs” and “hPDLS cells”. In order to identify and include all relevant studies on this topic into this review, the search strategy considered all terms and abbreviations identified. In all cases, PDL tissue derived from premolars or third molars. Both isolation techniques unequivocally result in a heterogeneous mixture of different cell types (Yamaguchi et al., 2002; Marchesan et al., 2011), though the “digestion” technique was shown to result in a cell population enriched with mesenchymal stem cells (Seo et al., 2004). Therefore, hPDLCs should be regarded as a heterogeneous cell population consisting of cells originating from different lineages. Most recently, *in-vitro* cell type verification was increasingly used including several studies on hPDLCs (Marchesan et al., 2011), that were also reported in some of the included studies herein, i.e. flow cytometric analysis of cell surface markers (Wang H. et al., 2019; Wu et al., 2019a), osteogenic potential as reflected by ALP staining and/or Ca^2+^ deposition (Jacobs et al., 2018; Wang H. et al., 2019), cell type specific gene expression pattern (Memmert et al., 2019), and immunohistochemical staining of vimentin and cytokeratin (Sun et al., 2017; Yu et al., 2018; Wang Y. et al., 2019).

Careful reference gene selection is essential to overcome variations in RT-qPCR experiments and to enhance comparability between various studies. In order to reduce the risk of bias in qPCR experiments, the “*Minimum Information for Publication of Quantitative Real-Time PCR Experiments* (MIQE)” guidelines were established, with reference gene selection being one of the most crucial steps in RT-qPCR establishment (Bustin et al., 2009). As such, the MIQE guidelines cover some of the reporting and methodology-related risk of bias criteria considered in the present review, directly or indirectly related to RT-qPCR. According to the present systematic review, *GAPDH* or *ACTB* were the most frequently adopted reference genes for qPCR experiments. The rationale for reference gene selection was only rarely stated. Only recently, MIQE reporting gained more attention (Janjic Rankovic et al., 2020). Several studies evaluated reference gene selection specifically focusing on hPDLCs in different areas of dentistry (Kirschneck et al., 2017; Setiawan et al., 2019; Nazet et al., 2020).

The heterogeneity of force parameters and the limitations of several experimental set-ups as discussed previously might also have considerable impact on the consistency and comparability between studies as included herein. Additionally, optimal force duration, magnitude and frequency mainly depend on the experimental design and the specific objectives of the study.

### In Silico Analysis of Gene Lists

To gain additional insights into the biological processes and pathways regulated by dynamic or static tension forces, gene-set enrichment analysis and protein-protein interaction (PPI) network construction were applied. Moreover, the most influential genes and sub-networks were identified in both PPI networks. The gene lists were created from the studies identified in this systematic review. To increase specificity, only those genes showing clear force-dependent expression were included.

*Gene-set enrichment analysis*: In general, gene-set enrichment analysis is applied to expression data of individual genes obtained by techniques like microarrays, next-generation sequencing or proteomics (Hutchins, 2014; Mooney and Wilmot, 2015). As such, the gene list contains ranking data (e.g. confidence scores, fold changes or similar quantitative information) or is unranked (Hung, 2013; Haw et al., 2020). Both types are then used for over-representation and/or gene-set enrichment analysis to identify relevant signaling and/or regulation pathways. To increase specificity of the gene lists, gene expression data was restricted to the criteria specified in Materials and Method. Additionally, protein expression data was excluded due to the heterogeneity of the methods used for quantification (quantification via western blotting or ELISA vs enzyme activities) and the specificity of some of the antibodies used in the immunoassays. The results of gene enrichment and the pathway analysis showed a close relationship with osteogenesis, osteoclastogenesis and apoptosis. These findings were consistent with the reporting of the relevant studies identified.

The search strategy used herein identified 18 reports applying tension to hPDLCs with subsequent microarray or RNA-seq analysis: 15 studies applied dynamic tension and 3 static tension. All studies reported the most significantly up- and down-regulated genes applying different cut-offs. A re-analysis was not possible, since full (raw) data was publicly not available. Nevertheless, data from these studies was included if qualified reanalysis using (s)qPCR or protein expression was reported additionally.

#### Protein-Protein Interaction Networks

Complementary to pathway enrichment analysis, protein-protein interaction networks were generated for dynamic and static tension gene sets using the “*Search Tool for the Retrieval of Interacting Genes/Proteins*” database (STRING-DB), which incorporates regularly updated data from different biological pathway and scientific literature databases (Szklarczyk et al., 2019). As such, STRING-DB not only contains data on experimental derived PPI, but also functional annotation from literature and computational predictions. For each individual PPI pair a confidence score is given (range: 0–1), with higher scores are “*meant to express an approximate confidence,* […], *of the association being true, given all the available evidence*” (Szklarczyk et al., 2019; p. D608). Both networks were analyzed regarding subclusters and most-influential nodes (i.e. hub genes) and 7 (dynamic) and 6 (static) subclusters were identified. Subsequent pathway enrichment analysis showed a more specific enrichment of GO/Biological Process terms and GeneAnalytics SuperPathways.

We applied a confidence score cut-off ≥ 0.700, thus only high confidence interactions were included. Interestingly, in both networks several differentially expressed genes were not integrated, including alkaline phosphatase (ALPP), being one of the most frequently analyzed genes/proteins identified herein. With the application of a confidence score cut-off of ≥0.400, thus including medium confidence interactions, ALPP was integrated into dynamic network due to co-mentioning in PubMed abstracts with bone sialoprotein 2 (IBSP; score: 0.407), osteopontin (SPP1; 0.438), bone morphogenetic protein 2 (BMP2; 0.434), RUNX2 (0.495), and osteocalcin (BGLAP; 0.627). GOSR1, CDC42EP2, UNC50, ACY1, and AMDHD2 were still not integrated. Application of the same threshold to the static network would integrate all nodes including ALPP (ALPP – RUNX2: 0.495; ALPP – BGLAP: 0.627; ALPP – SPP1: 0.438).

Interestingly, cluster #7 of the dynamic network consisted of nodes, that were contributed to the gene list based on one study (Fujihara et al., 2010). This finding demonstrates the limits of the approach applied: both lists of differentially expressed genes were compiled based on publications identified in our search strategy. The more specific an individual study deals with a specific aspect, the more specific it will be described by gene-list enrichment analysis and network cluster analysis. As such, measures were taken, to reduce this impact: 1) the genes included in our gene lists were those only with reports on changes in gene expression due to mechanical stimulation. 2) Additionally, cut-offs were applied to GO, pathway enrichment and PPI network construction, to exclude incorporation of data too general, that means very general biological processes with thousands of genes involved.

#### Meta-Analysis

Initially, a meta-analysis of the ten most frequently analyzed genes or metabolites was intended to supplement the findings. Unfortunately, due to heterogeneity of the experimental conditions, including force parameters, cell culture, reference gene selection in RT-qPCR experiments and incomplete reporting especially concerning the statistical unit, this was not further considered.

Several identified studies showed that not only gene and protein expression is regulated by tension application, but also post-translational modifications like proteolytic cleavage, activation by GTP-binding, phosphorylations and protein translocation between nucleus and cytoplasm, or cytoplasm and extracellular space. Regulation of second messengers like cAMP (Ngan et al., 1990) and metabolites like glutamate (Fujihara et al., 2010), NOx (Pelaez et al., 2017) and ATP (Tantilertanant et al., 2019a) are also effected by tension application, as well as microRNA and long non-coding RNAs (Chen et al., 2016). Epigenetic effects on gene expression also should be taken into account, since several genes discussed herein like *COL1A1* (Kaku and Yamauchi, 2014) and *RUNX2* (Montecino et al., 2015) are known to be under epigenetic control (Francis et al., 2019).

### Summary

In this systematic review we summarized relevant information about tension application on hPDLCs *in vitro* and assessed potential reporting and methodology-associated risk of bias related to this issue. Due to the enormous variety of apparatus in both, dynamic and static tension experiments, it is not possible to universally define optimum force parameters including force magnitude, duration and frequency. However, clinically relevant parameters were identified, that can be used as a reference for *in vitro* studies. Taken together, quantitative and qualitative information on mechanical stimulated gene and protein regulation and a comprehensive network analysis have provided more clear insights into the mechanisms involved in the OTM.

Future studies should focus on the comparison of dynamic and static tension. There is also a need to elucidate the differences between the application of equibiaxial and uniaxial tension in more detail, to develop an optimal *in vitro* model for the simulation of orthodontic force, and to provide more reliable evidence for clinical treatment.

## Data Availability

The original contributions presented in the study are included in the article/Supplementary Material. Further inquiries can be directed to the corresponding author.

## Glossary

ALP: Alkaline phosphatase
BGLAP: Bone gamma-carboxy glutamic acid-containing protein
BMPs: Bone morphogenetic proteins
COL I: Collagen type I
DEG: Differential expressed gene
ECM: Extracellular matrix
FC: Fold change
FGFs: Fibroblast growth factors
GO: GeneOntology
HoB: High risk of bias
hPDLFs: Human periodontal ligament fibroblasts
IL6: Interleukin 6
IL8: Interleukin 8
KEGG: Kyoto Encyclopedia of Genes and Genomes
LoB: Low risk of bias
MAPK: Mitogen-activated protein kinase
MCODE: Molecular Complex Detection
MIQE: Minimum Information for Publication of Quantitative Real-Time PCR Experiments
n. a.: Not applicable
OPG: Osteoprotegerin
OTM: Orthodontic tooth movement
PCR: Polymerase chain reaction
PDL: Periodontal ligament
PDLFs: Periodontal ligament fibroblasts
PDLSCs: Periodontal ligament stem cells
PGE_2_: Prostaglandin E_2_
PPI: Protein-protein interaction
PRISMA: Preferred Reporting Items for Systematic Reviews and Meta-Analyses
qPCR: Quantitative polymerase chain reaction
RANK: Receptor activator of NF-kappa B
RANKL: Receptor activator of the nuclear factor kappa ligand
RT-qPCR: Reverse transcriptase quantitative polymerase chain reaction
RT-sqPCR: Reverse transcriptase semi-quantitative polymerase chain reaction
RUNX2: Runt-related transcription factor 2 (identical with CBFA1)
STRING-DB: “Search Tool for the Retrieval of Interacting Genes/Proteins” database
TNFRSF11B: Tumor necrosis factor-alpha receptor superfamily member 11B
TNFSF11: TNF superfamily member 11
WB: Western blotting
