# Supplementary material for: Effect of Tension on Human Periodontal Ligament Cells: Systematic Review and Network Analysis
Source: Front Bioeng Biotechnol. 2021 Aug 27;9:695053. doi: 10.3389/fbioe.2021.695053 (PMC8429507; doi:10.3389/fbioe.2021.695053)
Supplement: Supplementary file 1 [file DataSheet7.pdf]

Supplement 7 The top ten most frequently genes/proteins/metabolites investigated in experiments applying tension on hPDLCs.

For each gene, the data was first sorted by type of force application ("dynamic"/"static") and then by frequency. For each gene, protein or metabolite, force parameters and expression pattern (increase, decrease and other changes) are given. The maximum/minimum of expression and corresponding force parameters are given. Cells reporting either "increase" (or "increase with plateau"), "decrease" (or "decrease with plateau") and "other" changes in expression (definition in Figure 5) were labelled green, red or purple, respectively.

Offical gene symbol	Frequency (Hz)	Reference	Force magnitude	Force duration	Force type	Gene expression: Increase, decrease, other changes (method w/ reference gene)	Gene expression: When it reaches peak and peak's magnitude (fold change; times or ratio; unclear = ?)	Protein expression: Increase, decrease, other changes (method w/ reference)	Protein expression: When it reaches peak and peak's magnitude (times or ratio; unclear = ?)
RUNX2 (dynamic)	0.005	Li et al. (2015)	5%	0.5h, 1h, 2h, 4h, 8h, 12h, 24h	uniaxial	increase (qPCR, GAPDH)	24h: 12.0 (ratio)*	increase followed by plateau (WB, GAPDH)	12h…24h: 2 (rel)* / 1.4 (ratio-calc)
		Yang et al. (2010)	12%	0.5h,1h, 2h, 4h, 6h, 12h, 24h	uniaxial	increase followed by decrease (sqPCR, β-actin)	highest @ 1h: 2.0 (optical density)* / 3.3 (ratio-calc relative to t_0)<br>lowest @ 12h: 0.3 (optical density)* / 0.5 (ratio-calc relative to t_0)	n.g.	n.g.
	0.05	Nokhbehsaim et al. (2011)	3%	1d, 6d	equibiaxial	decrease (qPCR, GAPDH)	1d: 0.2 (FC)*	n.g.	n.g.
		Nokhbehsaim et al. (2010)	3%, 20%	1d, 6d	equibiaxial	3%: decrease (qPCR, GAPDH)<br>20%: decrease (qPCR, GAPDH)	3% @ 1d: 0.24 (FC)<br>20% @ 1d: 0.27 (FC)	n.g.	n.g.
	0.1	Jiang and Hua (2016)	5%	6h, 12h, 24h, 48h	equibiaxial	increase (sqPCR, GAPDH)	24h: 2.9 (FC)*	increase (WB, GAPDH)	24h: no quantitative information given
		Yang et al. (2018)	10%	24h	equibiaxial	increase (qPCR, GAPDH)	1.8 (FC)*	n.g.	n.g.
		Wu et al. (2019)	10%	24h	equibiaxial	increase (qPCR, GAPDH)	1.6 (FC)†	increase (WB, GAPDH)	no quantitative information given
		Lee et al. (2015)	12%	48h	equibiaxial	increase (qPCR, β-actin)	3.8 (ratio)*	n.g.	n.g.
		Wang et al. (2019b)	12%	qPCR for 12h, 24h, 48h; WB for 24h, 48h	equibiaxial	increase (qPCR, β-actin)	48h: 1.3 (FC)†	increase (WB, GAPDH)	48h: no quantitative information given
		Chang et al. (2017)	12%	24h, 48h, 72h	equibiaxial	increase (qPCR, GAPDH)	72h: 3 (FC)*	n.g.	n.g.
		Shen et al. (2014)	12%	6h, 12h, 24h	equibiaxial	increase (qPCR, β-actin)	24h: 13.5 (rel)* / 3.4 (ratio-calc)	increase (WB, GAPDH)	24h: 415.1 (rel)* / 1.3 (ratio-calc)

Offical gene symbol	Frequency (Hz)	Reference	Force magnitude	Force duration	Force type	Gene expression: Increase, decrease, other changes (method w/ reference gene)	Gene expression: When it reaches peak and peak's magnitude (fold change; times or ratio; unclear = ?)	Protein expression: Increase, decrease, other changes (method w/ reference)	Protein expression: When it reaches peak and peak's magnitude (times or ratio; unclear = ?)
		Liu et al. (2017)	6%, 8%, 10%, 12%, 14%	12h	equibiaxial	HPDLSCs: increase (qPCR, β-actin)	HPDLSCs @ 12%: 3.7 (ratio)*	n.g.	n.g.
	0.2	Cho et al. (2010)	12%	0h, 3h, 6h, 12h, 24h, 48h	uniaxial	decrease followed by plateau then increase (sqPCR, GAPDH)	3h…6h: 0.5 (ratio)†<br>48h: 3 (ratio)†	n.g.	n.g.
		Cho et al. (2010)	3%, 6%, 12%, 15%	48h	uniaxial	decrease followed by increase (sqPCR, GAPDH)	12%: 0.8 (ratio)†<br>15%: 1.5 (ratio)†	n.g.	n.g.
	0.5	He et al. (2019)	n. g.	3h	uniaxial	increase (qPCR, β-actin)	10.1 (rel)† / 10.6 (ratio-calc)	n.g.	n.g.
		Tang et al. (2012)	0.3%	3h, 6h, 12h, 24h	uniaxial	increase (qPCR, GAPDH)	24h: 9.6 (rel)* / 7.4 (ratio-calc)	increase followed by plateau (WB, GAPDH)?	12h…24h: 1.1 (rel)* / 1.4 (ratio-calc)
		Fujihara et al. (2010)	10%	48h	uniaxial	increase (qPCR, HPRT)	0.7 (rel)* / 1.6 (ratio-calc)	n.g.	n.g.
		Li et al. (2013)	10%	12h, 24h, 48h	equibiaxial	increase followed by decrease (qPCR, GAPDH)	highest @ 24h: 3.2 (FC)*<br>lowest @ 48h: 0.2 (FC)*	increase followed by decrease (WB, GAPDH)	hightest @ 24h: 0.8 (rel)* / 2.0 (ratio-calc)<br>lowest @ 48h: 0.1 (rel)* / 0.4 (ratio-calc)
		Li et al. (2014)	10%	24h	equibiaxial	increase (qPCR, GAPDH)	3.3 (rel)* / 6.6 (ratio-calc)*	increase (WB, GAPDH)	0.1 (rel)* / 2.7 (ratio-calc)
		Ren et al. (2015)	10%	1h, 3h, 6h, 12h, 18h, 24h	equibiaxial	RUNX2: increase (qPCR, GAPDH)	RUNX2 @ 3h: 2.9 (FC)*	RUNX2: temporary increase followed by plateau then temporary decrease (WB, GAPDH)<br>p-RUNX2: increase followed by plateau (WB, GAPDH)	RUNX2: highest @ 3h…6h: 0.7 (rel)† / 1.9 (ratio-calc)<br>RUNX2: lowest @12h: 0.2 (rel)† / 0.6 (ratio-calc)<br>p-RUNX2 @ 3h…6h: 0.4 (rel)* / 9.5 (ratio-calc)
		Yu et al. (2018)	12%	24h, 48h, 72h	equibiaxial	increase (qPCR, ACTB)	48h: 3.4 (FC)*	increase (WB, β-actin)	no quantitative information given
		Sun et al. (2017)	12%	12h, 24h, 48h	uniaxial	temporary decrease (qPCR, GAPDH)	12h: 0.5 (ratio)	temporary decrease (WB,GAPDH)	12h: 0.8 (ratio)*
		Sun et al. (2016)	12%	1d, 5d	uniaxial	increase followed by decrease (qPCR, GAPDH)	highest @ 1d: 2.0 (ratio)*<br>lowest @ 5d: 0.9 (ratio)*	increase followed by decrease (WB, GAPDH)	highest @ 1d: 1.5 (ratio)*<br>lowest @ 5d: 0.8 (ratio)*
	1.0	Wang et al. (2019a)	10%	12h	equibiaxial	increase (qPCR, GAPDH)	2.5 (FC)*	n.g.	n.g.
		Wei et al. (2014)	10%	12h	equibiaxial	increase (qPCR, GAPDH)	1.8 (ratio)*	increase (WB, β-actin)	no quantitative information given
		Wei et al. (2015)	10%	6h, 12h, 24h, 48h	equibiaxial	increase (qPCR, GAPDH)	48h: 1.8 (FC)*	n.g.	n.g.

Offical gene symbol	Frequency (Hz)	Reference	Force magnitude	Force duration	Force type	Gene expression: Increase, decrease, other changes (method w/ reference gene)	Gene expression: When it reaches peak and peak's magnitude (fold change; times or ratio; unclear = ?)	Protein expression: Increase, decrease, other changes (method w/ reference)	Protein expression: When it reaches peak and peak's magnitude (times or ratio; unclear = ?)
RUNX2 (static)	n. a.	Wada et al. (2017)	15%	6h, 12h, 24h	equibiaxial	increase (qPCR, GAPDH)	24h: 1.7 (FC)*	n.g.	n.g.
ALPP (dynamic)	0.005	Yang et al. (2010)	12%	0.5h,1h, 2h, 4h, 6h, 12h, 24h	uniaxial	temporary increase (sqPCR, β-actin)	4h: 0.8 (rel to t0 with t0 = 0)*	n.g.	n.g.
	0.05	Yang et al. (2006)	310-320 grams force	2h, 4h, 6h, 12h, 24h	uniaxial	n.g.	n.g.	increase (biochemistry test)	4h: 3 (unit/10^4 cellls)* / 4.3 (ratio-calc)
		Nokhbehsaim et al. (2010)	3%, 20%	1d, 6d	equibiaxial	3%: increase followed by decrease (qPCR, GAPDH)<br>20%: decrease (qPCR, GAPDH)	3% highest @1d: 1.13 (FC)<br>3% lowest @ 6d: 0.59 (FC)<br>20% @ 6d: 0.34 (FC)	n.g.	n.g.
	0.1	Jiang and Hua (2016)	5%	6h, 12h, 24h, 48h	equibiaxial	increase (sqPCR, GAPDH)	24h: 2.7 (FC)*	increase (WB, GAPDH)	24h: no quantitative information given
		Yang et al. (2018)	10%	24h	equibiaxial	increase (qPCR, GAPDH)	1.8 (FC)*	n.g.	n.g.
		Lee et al. (2015)	12%	48h	equibiaxial	increase (qPCR, β-actin)	3.8 (ratio)*	n.g.	n.g.
		Chang et al. (2015)	12%	6h, 12h, 24h, 48h, 72h	equibiaxial	increase (qPCR, GAPDH)	72h: 5.3 (ratio)*	n.g.	n.g.
		Chang et al. (2017)	12%	24h, 48h, 72h	equibiaxial	n.g.	n.g.	increase (PNPP)	72h: 0.8 (U/mg)* / 2.7 (ratio-calc)
		Shen et al. (2014)	12%	6h, 12h, 24h	equibiaxial	increase (qPCR, β-actin)	24h: 13.8 (rel)* / 2.5 (ratio-calc)	increase (WB, GAPDH)	24h: 347.2 (rel)* / 1.5 (ratio-calc)
		Yamaguchi and Shimizu (1994)	24%	3d	equibiaxial	n.g.	n.g.	donor 1: decrease (ALP activity)<br>donor 2: decrease (ALP activity)<br>donor 3: decrease (ALP activity)	donor 1: 10.3 (mU/10^5 cells) / 0.6 (ratio-calc)<br>donor 2: 10.0 (mU/10^5 cells) / 0.6 (ratio-calc)<br>donor 3: 10.6 (mU/10^5 cells) / 0.6 (ratio-calc)
		Yamaguchi et al. (1996)	24%	1d, 3d, 5d	equibiaxial	n.g.	n.g.	decrease followed by plateau (ALP activity)	3d…5d: 10.7 (mU/10^5 cells)* / 0.6 (ratio-calc)
		Matsuda et al. (1998)	9%, 18%	2d, 4d, 6d	equibiaxial	n.g.	n.g.	9%: increase (ALP activity)<br>18%: increase (ALP activity)	9% @ 6d: 257.7 (U/mg protein)* / 1.4 (ratio-calc)<br>18% @ 4…6d: 230.8 (U/mg protein)* / 1.3 (ratio-calc)
		Liu et al. (2017)	6%, 8%, 10%, 12%, 14%	12h	equibiaxial	HPDLSCs: increase (qPCR, β-actin)	HPDLSCs @ 12%: 1.6 (ratio)*	n.g.	n.g.

Offical gene symbol	Frequency (Hz)	Reference	Force magnitude	Force duration	Force type	Gene expression: Increase, decrease, other changes (method w/ reference gene)	Gene expression: When it reaches peak and peak's magnitude (fold change; times or ratio; unclear = ?)	Protein expression: Increase, decrease, other changes (method w/ reference)	Protein expression: When it reaches peak and peak's magnitude (times or ratio; unclear = ?)
		Yamaguchi et al. (1996)	ALP activity for 9%, 12%, 15%, 18%, 21%, 24%; sqPCR for 12%, 24%	ALP activity for 5d; sqPCR for 3d	equibiaxial	decrease (Northern blot, β-actin)	quantitative information not given	decrease (ALP activity)	24%: 9.4 (mU/10^5 cells)* / 0.5 (ratio-calc)
	0.5	Qin and Hua (2016)	5%	1h, 3h, 6h	n.g.	not reported with reference to force (qPCR, GAPDH)		temporary decrease (WB, GAPDH)	1h: 0.03 (rel)* / 0.2 (ratio-calc)
		Fujihara et al. (2010)	10%	48h	uniaxial	increase (qPCR, HPRT)	0.12 (rel)* / 3 (ratio-calc)	n.g.	n.g.
		Yamaguchi et al. (2002)	15%	30min, 90min, 6h	equibiaxial	temporary decrease (sqPCR, GAPDH)	90min: 0.8 (ratio)*	n.g.	n.g.
		Chiba and Mitani (2004)	15%	2d, 5d	equibiaxial	n.g.	n.g.	decrease (ALP activity, colorimetric assay)	5d: 0.8 (ratio)*
	1.0	Monnouchi et al. (2011)	8%	1h	uniaxial	increase (qPCR, β-actin)	HPLF-2E: 2.3 (FC)*<br>HPLF-2D: 2.1 (FC)*<br>HPLF-3M: 2.9 (FC)*	n.g.	n.g.
		Konstantonis et al. (2014)	8%	12h	uniaxial	"young cells" (P3-6): increase (qPCR, GAPDH)<br>"senescent cells" (P20-24): increase (qPCR, GAPDH)	"young cells": 150% (rel)* / 1.5 (ratio-calc)<br>"senescent cells": 50% (rel)* / 1.5 (ratio-calc)	"young cells": ALP activity increase (colorimetric assay)<br>"senescent cells": ALP activity increase (colorimetric assay)	"young cells":150% (rel)* / 1.5 (ratio-calc)<br>"senescent cells": 95% (rel)* / 1.6 (ratio-calc)
		Papadopoulou et al. (2019)	8%	18h	uniaxial	increase (qPCR, GAPDH)	2.3 (FC)†	n.g.	n.g.
		Wei et al. (2015)	10%	6h, 12h, 24h, 48h	equibiaxial	n.g.	n.g.	increase (ALP activity)	48h: 0.6 [Sigma unit/(min * mg protein)]* / 11.0 (ratio-calc)
ALPP (static)	n. a.	Chen et al. (2014)	-100 kPa	sqPCR for 1d, 3d, 7d, 15d<br>ELISA for 3d, 7d, 15d	equibiaxial	increase (?, actin)	7d: 0.5 (?)* / 2.1 (ratio-calc)	increase (ELISA)	15d: 0.2 (μM/μg DNA)* / 1.2 (ratio-calc)
		Jacobs et al. (2013)	1%, 5%, 10%	12h	equibiaxial	increase (qPCR, actin + GAPDH)	5%: 2.7 (FC)*	n.g.	n.g.
		Nazet et al. (2020)	35%	24h, 48h, 72h	equibiaxial	n.g.	n.g.	increase followed by platform (ELISA)	48h...72h: 1.4 (ratio)†

Offical gene symbol	Frequency (Hz)	Reference	Force magnitude	Force duration	Force type	Gene expression: Increase, decrease, other changes (method w/ reference gene)	Gene expression: When it reaches peak and peak's magnitude (fold change; times or ratio; unclear = ?)	Protein expression: Increase, decrease, other changes (method w/ reference)	Protein expression: When it reaches peak and peak's magnitude (times or ratio; unclear = ?)
		Nazet et al. (2020)	7%, 10%, 16%. 35%	48h	equibiaxial	n.g.	n.g.	increase (ELISA)	16%: 1.5 (ratio)†
BGLAP (dynamic)	0.05	Yang et al. (2006)	310-320 grams	2h, 4h, 6h, 12h, 24h	uniaxial	n.g.	n.g.	increase (RIA)	12h: 1.6 (ng/10^4 cellls)* / 8 (ratio-calc)
	0.1	Jiang and Hua (2016)	5%	6h, 12h, 24h, 48h	equibiaxial	increase (sqPCR, GAPDH)	24h: 3 (FC)*	increase (WB, GAPDH)	24h: no quantitative information given
		Kim et al. (2007)	9%	6d	equibiaxial	increase (sqPCR, GAPDH)	75 (rel)* / 1.7 (ratio-calc)	n.g.	n.g.
		Wu et al. (2019)	10%	24h	equibiaxial	increase (qPCR, GAPDH)	2.1 (FC)†	n.g.	n.g.
		Yang et al. (2018)	10%	qPCR for 24 h; WB for 72h	equibiaxial	increase (qPCR, GAPDH)	2.0 (FC)*	increase (WB, GAPDH)	1.8 (ratio)*
		Lee et al. (2015)	12%	48h	equibiaxial	increase (qPCR, β-actin)	4.2 (FC?)† / 4.7 (ratio-calc)	n.g.	n.g.
		Shen et al. (2014)	12%	6h, 12h, 24h	equibiaxial	increase (qPCR, β-actin)	24h: 10.5 (rel)* / 2.6 (ratio-calc)	increase (WB, GAPDH)	24h: 279.2 (rel) * / 1.5 (ratio-calc)
		Chang et al. (2015)	12%	6h, 12h, 24h, 48h, 72h	equibiaxial	increase (qPCR, GAPDH)	72h: 2 (ratio)*	n.g.	n.g.
		Chang et al. (2017)	12%	72h	equibiaxial	n.g.	n.g.	increase (WB, β-actin)	72h: 1.8 (ratio)*
		Wang et al. (2019b)	12%	qPCR for 12h, 24h, 48h; WB for 24h, 48h	equibiaxial	increase (qPCR, β-actin)	48h: 1.3 (FC)†	increase (WB, GAPDH)	24h: no quantitative information given
	0.5	Qin and Hua (2016)	5%	1h, 3h, 6h	n.g.	not reported with reference to force (qPCR, GAPDH)		temporary decrease followed by temporary increase (WB, GAPDH)	lowest @ 1h: 0.09 (rel)* / 0.7 (ratio-calc) highest @ 3h: 0.2 (rel)* / 1.5 (ratio-calc)
		Ren et al. (2015)	10%	1h, 3h, 6h, 12h, 18h, 24h	equibiaxial	increase followed by plateau (qPCR, GAPDH)	18…24h: 1.5 (FC)*	n.g.	n.g.
		Yang et al. (2016)	10%	1h, 3h, 6h, 12h, 24h	equibiaxial	increase (qPCR,GAPDH)	24h: 3.2 (FC)*	n.g.	n.g.
	1.0	Wang et al. (2019a)	10%	12h	equibiaxial	increase (qPCR, GAPDH)	5.8 (FC)*	n.g.	n.g.
		Wei et al. (2014)	10%	12h	equibiaxial	increase (qPCR, GAPDH)	2.1 (ratio)*	increase (WB, β-actin)	no quantitative information given

Offical gene symbol	Frequency (Hz)	Reference	Force magnitude	Force duration	Force type	Gene expression: Increase, decrease, other changes (method w/ reference gene)	Gene expression: When it reaches peak and peak's magnitude (fold change; times or ratio; unclear = ?)	Protein expression: Increase, decrease, other changes (method w/ reference)	Protein expression: When it reaches peak and peak's magnitude (times or ratio; unclear = ?)
		Wei et al. (2015)	10%	6h, 12h, 24h, 48h	equibiaxial	increase (qPCR, GAPDH)	24h: 3.5 (FC)*	n.g.	n.g.
BGLAP (static)	n. a.	Chen et al. (2014)	-100 kPa	sqPCR for 1d, 3d, 7d, 15d<br>ELISA for 7d, 15d	equibiaxial	increase (?, actin)	15d: 0.3 (?)* / 2.8 (ratio-calc)	increase (ELISA)	15d: 31.4 (pg/ml/cell)* / 1.3 (ratio-calc)
		Jacobs et al. (2013)	1%, 5%, 10%	12h	equibiaxial	increase followed by decrease (qPCR, actin + GAPDH)	highest @ 1%: 1.3 (FC)*<br>lowest @ 5%: 0.6 (FC)*	n.g.	n.g.
IL1B (dynamic)	0.005	Long et al. (2001)	6%	sqPCR for 4h, 24h, 48h; ELISA for 24h, 48h	equibiaxial	no expression (sqPCR, GAPDH)	no quantitative information is given	no expression (ELISA)	no quantitative information given
		Long et al. (2001)	sqPCR: 3%, 6%, 10%, 15%; ELISA: 6%, 10%, 15%	24h	equibiaxial	decrease followed by plateau then increase (sqPCR, GAPDH)	lowest @ 6%…10%: 11.4 (rel) / 0.1 (ratio-calc)<br>highest @15%: 152.3 (rel) / 1.5 (ratio-calc)	increase (ELISA)	15%: 85.7 (pg/ml)* / control n.g
	0.05	Nokhbehsaim et al. (2012)	3%, 20%	1d, 6d	equibiaxial	3%: decrease (qPCR, GAPDH)<br>20%: decrease (qPCR, GAPDH)	3% @ 6d: 0.08 (FC)*<br>20% @ 6d: 0.5 (FC)*	n.g.	n.g.
		Nokhbehsaim et al. (2010)	3%, 20%	1d, 6d	equibiaxial	3%: increase (qPCR, GAPDH)<br>20%: increase (qPCR, GAPDH)	3% @ 1d: 12.41 (FC)<br>20% @ 6d: 36.16 (FC)	n.g.	n.g.
	0.1	Shimizu et al. (1997)	18%	RIA for 1d, 3d, 5d<br>sqPCR for 3d	equibiaxial	"young cells" (P5-6): increase (sqPCR, GAPDH)<br>"old cells" (P18-20): increase (sqPCR, GAPDH)	no quantitative information is given	"young cells": increase followed by plateau (RIA)<br>"old cells": increase (RIA)	"young cells" @ 3d…5d: 40 (fmol/10^5 cells)* / 2.1 (ratio-calc)<br>"old cells" @ 5d: 60 (fmol/10^5 cells)* / 3 (ratio-calc)
		Shimizu et al. (1995)	18%	1d, 3d, 5d	equibiaxial	n.g.	n.g.	increase followed by plateau (radioactivity)	3d…5d: 54.1 (fmol/10^5 cells)* / 2.1 (ratio-calc)
		Abiko et al. (1998)	18%	1d, 3d, 5d	equibiaxial	n.g.	n.g.	"young cells" (P5-6): increase followed by plateau (ELISA)<br>"old cells" (P18-20): increase (ELISA)	"young cells" @ 3d…5d: 40.15 (ng/10^6 cellls) / 2.1 (ratio-calc)<br>"old cells" @ 5d: 58.10 (ng/10^6 cellls) / 2.8 (ratio-calc)

Offical gene symbol	Frequency (Hz)	Reference	Force magnitude	Force duration	Force type	Gene expression: Increase, decrease, other changes (method w/ reference gene)	Gene expression: When it reaches peak and peak's magnitude (fold change; times or ratio; unclear = ?)	Protein expression: Increase, decrease, other changes (method w/ reference)	Protein expression: When it reaches peak and peak's magnitude (times or ratio; unclear = ?)
		Zhuang et al. (2019)	20%	WB for 6h, 24h; ELISA for 1h, 2h, 4h, 6h, 12h, 24h	equibiaxial	n.g.	n.g.	Pro-IL-1β (31 kDa): increase followed by decrease (WB, GAPDH)<br>Mature-IL-1β (17 kDa): decrease (WB, GAPDH)<br>IL-1β in culture medium: increase (ELISA)	Pro-IL-1β highest @ 6h: 0.8 (rel)* / 1.1 (ratio-calc)<br>Pro-IL-1β lowest @24h: 0.6 (rel)* / 0.9 (ratio-calc)<br>Mature-IL-1β @ 24h: 0.3 (rel)* / 0.6 (ratio-calc)<br>IL-1β in culture medium 6h: 10.4 ((ratio)*
		Zhao et al. (2016)	20%	qPCR and WB for 0h, 6h, 24h; ELISA for 1h, 2h, 4h, 6h, 12h, 24h	equibiaxial	increase followed by decrease (qPCR, GAPDH)	highest @ 6h: 1.4 (ratio)*<br>lowest @ 24h: 0.6 (ratio)*	Pro-IL-1β (31 kDa): increase (WB, GAPDH)<br>IL-1β (17 kDa): increase (WB, GAPDH)<br>IL-1β in the culture medium: temporary increase (ELISA)	Pro-IL-1β @ 6h: 1.7 (ratio)*<br>IL-1β @6h: 1.8 (ratio)*<br>IL-1β in the culture medium @ 6h: 5.5 (ratio)*
		Liu et al. (2017)	6%, 8%, 10%, 12%, 14%	12h	equibiaxial	n.g.	n.g.	HPDLSCs: increase followed by plateau (ELISA)	HPDLSCs @ 6%...14%: 3.2 (pg/10^6 cells)* / 1.5 (ratio)*
		Shimizu et al. (1997)	9%, 18%	5d	equibiaxial	n.g.	n.g.	"young cells" (P5-6): increase (RIA)<br>"old cells" (P18-20): increase (RIA)	"young cells" @ 18%: 41.6 (fmol/10^5 cells)* / 2.1 (ratio-calc)<br>"old cells" @ 18%: 61.3 (fmol/10^5 cells)* / 3.1 (ratio-calc)
		Shimizu et al. (1994)	9%, 18%	1d, 3d, 5d	equibiaxial	n.g.	n.g.	9%: increase followed by plateau (RIA)<br>18%: increase followed by plateau (RIA)	9% @ 3d…5d: 27.9 (fmol/10^5 cells)* / 1.2 (ratio-calc)<br>18% @ 3d…5d: 42.3 (fmol/10^5 cells)* / 1.8 (ratio-calc)
		Shimizu et al. (1997)	n.g.	5d	equibiaxial	n.g.	n.g.	Donor 1, "young cells" (P5-6): increase (RIA)<br>Donor 1, "old cells" (P18-20): increase (RIA)<br>Donor 2, "young cells" (P5-6): increase (RIA)<br>Donor 2, "old cells" (P18-20): increase (RIA)<br>Donor 3, "young cells" (P5-6): increase (RIA)<br>Donor 3, "old cells" (P18-20): increase (RIA)	Donor 1, "young cells": 39.8 (fmol/10^5 cells)† / 2.1 (ratio-calc)<br>Donor 1, "old cells": 60.2 (fmol/10^5 cells)† / 3.0 (ratio-calc)<br>Donor 2, "young cells": 35.0 (fmol/10^5 cells)† / 2.4 (ratio-calc)<br>Donor 2, "old cells": 51.7 (fmol/10^5 cells)† / 3.3 (ratio-calc)<br>Donor 3, "young cells": 39.3 (fmol/10^5 cells)† / 2.2 (ratio-calc)<br>Donor 3, "old cells": 55.3 (fmol/10^5 cells)† / 3.2 (ratio-calc)
	0.2	Lee et al. (2012)	12%	3h, 6h, 12h, 24h, 48h	uniaxial	increase (sqPCR, GAPDH)	48h: 3 (ratio)†	n.g.	n.g.

Offical gene symbol	Frequency (Hz)	Reference	Force magnitude	Force duration	Force type	Gene expression: Increase, decrease, other changes (method w/ reference gene)	Gene expression: When it reaches peak and peak's magnitude (fold change; times or ratio; unclear = ?)	Protein expression: Increase, decrease, other changes (method w/ reference)	Protein expression: When it reaches peak and peak's magnitude (times or ratio; unclear = ?)
		Lee et al. (2012)	3%, 6%, 12%, 15%	24h	uniaxial	increase (sqPCR, GAPDH)	15%: 3.3 (ratio)†	n.g.	n.g.
	0.5	Kaku et al. (2019)	12%	48h	equibiaxial	n.g.	n.g.	increase (ELISA)	12.1 (pg/ml)†/ 1.6 (ratio-calc)
		Sun et al. (2017)	12%	12h, 24h, 48h	uniaxial	n.g.	n.g.	increase (ELISA)	48h: 89.76 (pg/ml) / 89.8 (ratio-calc)
		Sun et al. (2016)	12%	1d, 5d	uniaxial	n.g.	n.g.	increase (ELISA)	5d: 53 (pg/ml)* / control not detectable
IL1B (static)	n. a.	Wada et al. (2017)	15%	6h, 12h, 24h	equibiaxial	increase (qPCR, GAPDH)	24h: 5.2 (FC)*	n.g.	n.g.
		Ritter et al. (2007)	2.5%	6h	equibiaxial	increase (qPCR, β-actin)	5.8 (FC)	n.g.	n.g.
PTGS2 (dynamic)	0.005	Long et al. (2002)	6%	4h, 24h, 48h	equibiaxial	decrease (sqPCR, GAPDH)	quantitative information not given	n.g.	n.g.
		Agarwal et al. (2003)	15%	4h, 24h, 48h	equibiaxial	increase (sqPCR; GAPDH)	48h: 116.8 (rel)† / 31.8 (ratio-calc)	n.g.	n.g.
		Agarwal et al. (2003)	3%, 6%, 8%	4h	equibiaxial	increase (sqPCR; GAPDH)	6%: 7.6 (rel)†/ 2.2 (ratio-calc)	n.g.	n.g.
	0.017	Suzuki et al. (2014)	5%	6h	uniaxial;	increase (qPCR, GAPDH)	6h: 66.3 (rel)* / 25.2 (ratio-calc)	n.g.	n.g.
	0.05	Nogueira et al. (2014)	3%, 20%	36h	equibiaxial	n.g.	n.g.	increase (ELISA)	20%: 210 (pg/mL)* / 1.8 (ratio-calc)
		Nokhbehsaim et al. (2010)	3%, 20%	1d, 6d	equibiaxial	3% temporary increase (qPCR, GAPDH)<br>20% increase (qPCR, GAPDH)	3% @ 1d: 1.29 (FC)<br>20% @ 6d: 3.32 (FC)	n.g.	n.g.
		Nokhbehsaim et al. (2012)	3%, 20%	1d, 6d	equibiaxial	3%: no change (qPCR, GAPDH)<br>20%: increase (qPCR, GAPDH)	20% @ 6d: 1.5 (FC)*	n.g.	n.g.
	0.1	Ohzeki et al. (1999)	unclear	unclear	equibiaxial	increase (sqPCR, GAPDH)	no quantitative information is given	n.g.	n.g.
		Abiko et al. (1998)	18%	1d, 3d, 5d	equibiaxial	increase (sqPCR, GAPDH)	only day 3 reported (no quantitative information is given)	n.g.	n.g.

Offical gene symbol	Frequency (Hz)	Reference	Force magnitude	Force duration	Force type	Gene expression: Increase, decrease, other changes (method w/ reference gene)	Gene expression: When it reaches peak and peak's magnitude (fold change; times or ratio; unclear = ?)	Protein expression: Increase, decrease, other changes (method w/ reference)	Protein expression: When it reaches peak and peak's magnitude (times or ratio; unclear = ?)
		Shimizu et al. (1998)	18%	6h, 24h, 3d, 5d	equibiaxial	increase (sqPCR, GAPDH)	no quantitative information is given	n.g.	n.g.
	0.5	Wang et al. (2011)	0.5%	2h	uniaxial	increase (qPCR, GAPDH)	6.4 (FC)*	n.g.	n.g.
PTGS2 (static)	n. a.	(2018)	3%	12h	equibiaxial	increase (qPCR; actin + GAPDH)	3.8 (FC)†	n.g.	n.g.
		Wada et al. (2017)	15%	6h, 12h, 24h	equibiaxial	increase (qPCR, GAPDH)	12h: 3.8 (FC)*	n.g.	n.g.
		Jacobs et al. (2014)	1%, 5%, 10%	12h	equibiaxial	increase (qPCR, actin + GAPDH)	10%: 31.4 (FC)	n.g.	n.g.
		Nazet et al. (2020)	35%	24h, 48h, 72h	equibiaxial	temporary increase (qPCR, TBP/PPIB)	48h: 2.2 (FC)†	n.g.	n.g.
		Nazet et al. (2020)	7%, 10%, 16%. 35%	48h	equibiaxial	increase (qPCR, TBP/PPIB)	35%: 2.5 (FC)†	n.g.	n.g.
TNFRSF11B (dynamic)	0.005	Li et al. (2015)	5%	0.5h, 1h, 2h, 4h, 8h, 12h, 24h	uniaxial	increase (qPCR, GAPDH)	12h: 4.0 (ratio)*	increase (WB, GAPDH)	8h: 0.9 (rel)* / 4.4 (ratio-calc)
		Yang et al. (2010)	12%	0.5h,1h, 2h, 4h, 6h, 12h, 24h	uniaxial	increase followed by decrease (sqPCR, β-actin)	highest @ 2h: 1.5 (rel)* / 1.4 (ratio-calc relative to t_0)<br>lowest @ 6h: 0.3 (rel)* / 0.3 (ratio-calc relative to t_0)	n.g.	n.g.
	0.05	Yang et al. (2006)	310-320 grams	2h, 4h, 6h, 12h, 24h	uniaxial	decrease (in-situ hybridization staining)	4h: 0.3 (optical density)* / 0.5 (ratio-calc)	decrease (ELISA)	24h: 38.9 (10^{-15} mol)* / 0.9 (ratio-calc)
		Nogueira et al. (2014)	20%	1d, 3d	equibiaxial	OPG: decrease followed by plateau (qPCR, GAPDH)<br>*RANKL/OPG: increase*	OPG @ 1d…3d: 0.2 (FC)*<br>*RANKL/OPG @ 1d: 4.5 (ratio)**	OPG: decrease (ELISA)<br>*RANKL/OPG: increase*	OPG @ 1d: 0.6 (ratio)*<br>*RANKL/OPG @ 3d: 1.4 (ratio)**
	0.1	Lee et al. (2015)	12%	48h	equibiaxial	increase (qPCR, β-actin)	7.7 (ratio)*	n.g.	n.g.
		Liu et al. (2017)	6%, 8%, 10%, 12%, 14%	12h	equibiaxial	HPDLSCs: increase (qPCR, β-actin)	HPDLSCs @ 12%: 1.6 (ratio)*	n.g.	n.g.
	0.17	Tsuji et al. (2004)	20%	sqPCR for 12h, 24h, 48h, 72h, 120h; qPCR for 48h	equibiaxial	increase (sqPCR, GAPDH)<br>increase (qPCR, β-actin)	sqPCR @ 48h: 2.2 (ratio)*<br>qPCR @ 48h: 34.8 (rel)* / 6.4 (ratio-calc)	n.g.	n.g.

Offical gene symbol	Frequency (Hz)	Reference	Force magnitude	Force duration	Force type	Gene expression: Increase, decrease, other changes (method w/ reference gene)	Gene expression: When it reaches peak and peak's magnitude (fold change; times or ratio; unclear = ?)	Protein expression: Increase, decrease, other changes (method w/ reference)	Protein expression: When it reaches peak and peak's magnitude (times or ratio; unclear = ?)
		Tsuji et al. (2004)	sqPCR for 5%, 20%, 25%; ELISA for 20%	48h	equibiaxial	increase (sqPCR, GAPDH)	20%: 1.5 (rel)*/ 2.2 (ratio-calc)	increase (ELISA)	20%: 339 (pM) / 3.0 (ratio-calc)
	0.5	Kanzaki et al. (2019)	15%	24h	equibiaxial	increase (qPCR, RPS18)	2.2 (FC)*	increase (WB, n.g.)<br>increase (ELISA)	WB: 1.8 (ratio)*<br>ELISA: 16.3 (ng/ml)* / 1.3 (ratio-calc)
		Kanzaki et al. (2006)	15%	sqPCR for 48h; ELISA for 72h	equibiaxial	increase (sqPCR, β-actin)	1.7 (ratio)*	increase (ELISA)	277.1 (pmol/L)* / 1.3 (ratio-calc)
	1	Monnouchi et al. (2011)	8%, 12%	1h	uniaxial	increase (sqPCR, GAPDH)	HPDLF-2E: 12%: 2.2 (FC)*	n.g.	n.g.
TNFRSF11B (static)	n. a.	Liao and Hua (2013)	1.5%	60min	equibiaxial	*OPG:* increase (qPCR, GAPDH)<br>*OPG/RANKL*: increase (qPCR, GAPDH)	1.4 (ratio)*<br>1.2 (ratio)*	OPG: increase (ELISA)<br>OPG/RANKL: increase (ELISA)	2127.7 (ng/mL)* / 1.2 (ratio-calc)<br>1.2 (rel)* / 1.7 (ratio-calc)
		Spencer and Lallier (2009)	10%	12h	equibiaxial	increase (sqPCR, S15rRNA)	2.3 (ratio)*	n.g.	n.g.
		Jacobs et al. (2015)	5%, 10%	12h	equibiaxial	OPG: increase (qPCR, actin + GAPDH)<br>*RANKL/OPG: temporary decrease*	OPG @ 5%: 2.9 (FC)*<br>*RANKL/OPG @ 5%:* 0.2 (ratio)*	increase (ELISA)	5%: 13.7 (ng/ml)* / 3.1 (ratio-calc)
		Jacobs et al. (2013)	1%, 5%, 10%	12h	equibiaxial	increase (qPCR, actin + GAPDH)	5%: 2.7 (FC)*	increase (ELISA)	10%: 44.6 (ng/10^5 cells)* / control no OPG detectable
TNFSF11 (dynamic)	0.005	Li et al. (2015)	5%	0.5h, 1h, 2h, 4h, 8h, 12h, 24h	uniaxial	increase (qPCR, GAPDH)	1h: 3.9 (ratio)*	increase (WB, GAPDH)	4h: 1.3 (rel)* / 3.3 (ratio-calc)
		Yang et al. (2010)	12%	0.5h,1h, 2h, 4h, 6h, 12h, 24h	uniaxial	increase (sqPCR, β-actin)	24h: 0.7 (optical density)†/ 14.0 (ratio-calc relative to t_0)	n.g.	n.g.
	0.05	Nogueira et al. (2014)	20%	1d, 3d	equibiaxial	RANKL: decrease (qPCR, GAPDH)<br>*RANKL/OPG: increase*	RANKL @ 3d: 0.3 (FC)*<br>*RANKL/OPG @ 1d: 4.5 (ratio)**	RANKL: decrease (ELISA)<br>*RANKL/OPG: increase*	RANKL @ 1d: 0.6 (ratio)*<br>*RANKL/OPG @ 3d: 1.4 (ratio)**
	0.1	Lee et al. (2015)	12%	48h	equibiaxial	increase (qPCR, β-actin)	1.9 (rel)* / 1.2 (ratio-calc)	n.g.	n.g.
		Liu et al. (2017)	6%, 8%, 10%, 12%, 14%	12h	equibiaxial	HPDLSCs: increase (qPCR, β-actin)	HPDLSCs @ 14%: 1.8 (ratio)*	n.g.	n.g.

Offical gene symbol	Frequency (Hz)	Reference	Force magnitude	Force duration	Force type	Gene expression: Increase, decrease, other changes (method w/ reference gene)	Gene expression: When it reaches peak and peak's magnitude (fold change; times or ratio; unclear = ?)	Protein expression: Increase, decrease, other changes (method w/ reference)	Protein expression: When it reaches peak and peak's magnitude (times or ratio; unclear = ?)
	0.17	Tsuji et al. (2004)	20%	48h	equibiaxial	no change (sqPCR, GAPDH)		n.g.	n.g.
	0.5	Kaku et al. (2019)	12%	48h	equibiaxial	n.g.	n.g.	increase (ELISA)	27.8 (pg/ml)†/ 3.3 (ratio-calc)
		Kanzaki et al. (2019)	15%	24h	equibiaxial	increase (qPCR, RPS18)	9.9 (FC)*	n.g.	n.g.
		Kanzaki et al. (2006)	15%	48h	equibiaxial	increase (sqPCR, β-actin)	12.9 (ratio)*	n.g.	n.g.
	1	Monnouchi et al. (2011)	8%, 12%	1h	uniaxial	decrease followed by increase (sqPCR, GAPDH)	HPDLF-2E: lowest @ 8%: 0.5 (FC)*<br>HPDLF-2E: highest @ 12%: 1.3 (FC)*	n.g.	n.g.
TNFSF11 (static)	n. a.	Liao and Hua (2013)	1.5%	60min	equibiaxial	*RANKL:* increase (qPCR, GAPDH)<br>*OPG/RANKL*: increase (qPCR, GAPDH)	1.2 (ratio)*<br>1.2 (ratio)*	RANKL: decrease (ELISA)<br>OPG/RANKL: increase (ELISA)	1787.2 (ng/mL)* / 0.7 (ratio-calc)<br>1.2 (rel)* / 1.7 (ratio-calc)
		Spencer and Lallier (2009)	10%	12h	equibiaxial	decrease (sqPCR, S15rRNA)	0.2 (ratio)*	n.g.	n.g.
		Jacobs et al. (2015)	5%, 10%	12h	equibiaxial	*RANKL:* decrease followed by increase (qPCR, actin + GAPDH)<br>*RANKL/OPG: temporary decrease*	*RANKL* lowest @ 5%: 0.6 (FC)*<br>*RANKL* highest @ 10%: 2.4 (FC)*<br>*RANKL/OPG @ 5%:* 0.2 (ratio)*	n.g.	n.g.
		Jacobs et al. (2013)	1%, 5%, 10%	12h	equibiaxial	increase followed by decrease (qPCR, actin + GAPDH)	highest @1%: 1.6 (FC)*<br>lowest @ 5%: 0.7 (FC)*	n.g.	n.g.
COL1A1 (dynamic)	0.017	Nemoto et al. (2010)	5%	1h, 3h, 12h, 24h, 48h, 1d, 3d, 5d, 7d	uniaxial	short time: decrease (qPCR, GAPDH)<br>long time: decrease (qPCR, GAPDH)	short time @ 24h: 0.3 (rel)* / 0.3 (ratio-calc)<br>long time @ 3d: 0.2 (rel)* / 0.3 (ratio-calc)	n.g.	n.g.
	0.1	Yang et al. (2018)	10%	24h	equibiaxial	increase (qPCR, GAPDH)	1.7 (FC)*	n.g.	n.g.
		Chen et al. (2015)	12%	24h	uniaxial	increase (qPCR, β actin)	1.2 (FC)*	n.g.	n.g.
		Lee et al. (2015)	12%	48h	equibiaxial	increase (qPCR, β-actin)	4.1 (ratio)*	n.g.	n.g.
		Wang et al. (2019b)	12%	qPCR for 12h, 24h, 48h; WB for 24h, 48h	equibiaxial	increase (qPCR, β-actin)	48h: 1.6 (FC)†	increase (WB, GAPDH)	48h: no quantitative information given

Offical gene symbol	Frequency (Hz)	Reference	Force magnitude	Force duration	Force type	Gene expression: Increase, decrease, other changes (method w/ reference gene)	Gene expression: When it reaches peak and peak's magnitude (fold change; times or ratio; unclear = ?)	Protein expression: Increase, decrease, other changes (method w/ reference)	Protein expression: When it reaches peak and peak's magnitude (times or ratio; unclear = ?)
	0.5	Qin and Hua (2016)	5%	1h, 3h, 6h	n.g.	not reported with reference to force (qPCR, GAPDH)		increase (WB, GAPDH)	3h: 0.16 (rel)* / 3.2 (ratio-calc)
		He et al. (2004)	10%	24h	equibiaxial	increase (qPCR, GAPDH)	5.695 (rel) / 4.4 (ratio-calc)	increase (ELISA)	4.1 (pg/cell)* / 3.5 (ratio-calc)
		Sun et al. (2016)	12%	1d, 5d	uniaxial	temporary decrease (qPCR, GAPDH)	1d: 0.7 (ratio)*	decrease (WB, GAPDH)	1d: 0.6 (ratio)*
		Sun et al. (2017)	12%	12h, 24h, 48h	uniaxial	decrease (qPCR, GAPDH)	24h: 0.5 (ratio)*	decrease (WB,GAPDH)	24h: 0.4 (ratio)*
		Yu et al. (2018)	12%	24h, 48h, 72h	equibiaxial	increase (qPCR, ACTB)	48h: 1.3 (FC)*	increase (WB, β-actin)	no quantitative information given
		Yamaguchi et al. (2002)	15%	30min, 90min, 6h	equibiaxial	temporary increase (sqPCR, GAPDH)	30min: 1.2 (ratio)*	n.g.	n.g.
COL1A1 (static)	n. a.	Takano et al. (2009)	5%, 10%	12h	uniaxial	increase (qPCR, β-actin)	10%: 1.9 (ratio)*	increase (ELISA)	10%: 2.4 (ug/ml)* / 1.7 (ratio-calc)
		Jacobs et al. (2013)	1%, 5%, 10%	12h	equibiaxial	decrease followed by increase then decrease (qPCR, actin + GAPDH)	1%: 0.8 (FC)†<br>5%: 1.1 (FC)†<br>10%: 0.7 (FC)†	n.g.	n.g.
PGE2 (dynamic)	0.005	Agarwal et al. (2003)	15%	12h, 24h, 48h	equibiaxial	n.a.	n.a.	increase followed by plateau (RIA)	24h…48h: 75.1† (?) / 15.6 (ratio-calc)
		Long et al. (2002)	1.8%, 3%, 6%, 10%, 12.5%	24h	equibiaxial	n.a.	n.a.	1.8%, 3%, 6%: no expression (RIA)<br>10%, 12.5%: increase (RIA)	12.5%: 102.8 (ng /10^6 cells) / control: no PGE_2 detectable
		Agarwal et al. (2003)	1%, 2%, 3%, 4%, 5%, 6%, 7%, 8%, 10%, 12.5%, 15%, 18%	24h	equibiaxial	n.a.	n.a.	1%, 2%, 3%, 4%, 5%, 6%, 7%, 8%: no expression (RIA)<br>10%, 12.5%, 15%, 18%: increase (RIA)	18%: 135 (ng/10^6 cells)* / control n.g.
	0.017	Suzuki et al. (2014)	5%	6h	uniaxial	n.a.	n.a.	increase followed by plateau (ELISA)	1h…6h: 229.9 (pg/ml)* / 2.9 (ratio-calc)
	0.05	Nogueira et al. (2014)	3%, 20%	1d, 3d	equibiaxial	n.a.	n.a.	1d: increase (ELISA)<br>3d: increase followed by plateau (ELISA)	20% @ 1d: 28.3 (ratio)†<br>3…20% @ 3d: 4.3 (ratio)†

Offical gene symbol	Frequency (Hz)	Reference	Force magnitude	Force duration	Force type	Gene expression: Increase, decrease, other changes (method w/ reference gene)	Gene expression: When it reaches peak and peak's magnitude (fold change; times or ratio; unclear = ?)	Protein expression: Increase, decrease, other changes (method w/ reference)	Protein expression: When it reaches peak and peak's magnitude (times or ratio; unclear = ?)
	0.1	Ohzeki et al. (1999)	18%	5d	equibiaxial	n.a.	n.a.	"young cells" (P5-7): increase (RIA)<br>"aged cells" (P19-11): increase (RIA)	"young cells" (donor 1): 8.3 (ng/10^6 cells) / 13.2 (ratio-calc); 2.5 (ug/mg protein) / 8.1 (ratio-calc)<br>"aged cells" (donor 1): 12.0 (ng/10^6 cells) / 24 (ratio-calc); 2.9 (ug/mg protein) / 11.6 (ratio-calc)<br>"young cells" (donor 2): 9.3 (ng/10^6 cells) / 18.6 (ratio-calc); 2.6 (ug/mg protein) / 8.4 (ratio-calc)<br>"aged cells" (donor 2): 14.0 (ng/10^6 cells) / 18.7 (ratio-calc); 3.2 (ug/mg protein) / 12.8 (ratio-calc)<br>"young cells" (donor 3): 9.3 (ng/10^6 cells) / 14.8 (ratio-calc); 2.5 (ug/mg protein) / 8.2 (ratio-calc)<br>"aged cells" (donor 3): 12.0 (ng/10^6 cells) / 19.0 (ratio-calc); 3.0 (ug/mg protein) / 10.7 (ratio-calc)
		Abiko et al. (1998)	18%	1d, 3d, 5d	equibiaxial	n.a.	n.a.	"young cells" (P5-6): increase (ELISA)<br>"old cells" (P18-20): increase (ELISA)	"young cells" @ 5d: 8.20 (ng/10^6 cellls) / 14.9 (ratio-calc)<br>"old cells" @ 5d: 12.25 (ng/10^6 cellls) / 18 (ratio-calc)
		Ohzeki et al. (1999)	18%	1d, 3d, 5d	equibiaxial	n.a.	n.a.	"young cells" (P5-7): increase (RIA)<br>"aged cells" (P19-22): increase (RIA)	"young cells" @ 5d: 5.9 (ng/10^6 cells) / 11.2 (ratio-calc)<br>"aged cells" @ 5d: 12.1 (ng/10^6 cells) / 17.3 (ratio-calc)
		Shimizu et al. (1995)	18%	1d, 3d, 5d	equibiaxial	n.a.	n.a.	increase (RIA)	5d: 7.9 (ng/10^6 cells)* / 19.8 (ratio-calc)
		Yamaguchi et al. (1994)	18%	1d, 3d, 5d	equibiaxial	n.a.	n.a.	increase (RIA)	5d: 8.9 (ng/10^6 cells)* / 17.8 (ratio-calc)
		Shimizu et al. (1998)	18%	1d, 2d, 3d, 4d, 5d	equibiaxial	n.a.	n.a.	increase (RIA)	5d: 9.1(ng/10^6 cells) / 10.1 (ratio-calc)
		Ohzeki et al. (1999)	9%, 18%	5d	equibiaxial	n.a.	n.a.	"young cells" (P5-7): increase (RIA)<br>"aged cells" (P19-22): increase (RIA)	"young cells" @ 18%: 5.9 (ng/10^6 cells) / 5.6 (ratio-calc)<br>"aged cells" @ 18%: 11.6 (ng/10^6 cells) / 9.4 (ratio-calc)
		Yamaguchi et al. (1994)	9%, 12%, 15%, 18%, 21%, 24%	5d	equibiaxial	n.a.	n.a.	increase (RIA)	24%: 14 (ng/10^6 cells)* / 28 (ratio-calc)

Offical gene symbol	Frequency (Hz)	Reference	Force magnitude	Force duration	Force type	Gene expression: Increase, decrease, other changes (method w/ reference gene)	Gene expression: When it reaches peak and peak's magnitude (fold change; times or ratio; unclear = ?)	Protein expression: Increase, decrease, other changes (method w/ reference)	Protein expression: When it reaches peak and peak's magnitude (times or ratio; unclear = ?)
PGE2 (static)	n. a.	Jacobs et al. (2014)	1%, 5%, 10%	12h	equibiaxial	n.a.	n.a.	increase (ELISA)	10%: 47.9 (pg/ml), ration can not be calculated
		Jacobs et al. (2018)	3%	12h	equibiaxial	n.a.	n.a.	no change (ELISA)	
		Ngan et al. (1990)	0.28%, 0.95%, 1.09%, 1.72%	5min, 15min, 30min, 60min	equibiaxial	n.a.	n.a.	0.28%: increase (RIA)<br>0.95%: temporary decrease (RIA)<br>1.09%: increase (RIA)<br>1.72%: increase (RIA)	0.28% @ 120min: 109 (pg/10^4 PDL cells)* / 1.1 (ratio-calc)<br>0.95% @ 15min: 98 (pg/10^4 PDL cells)* / 0.9 (ratio-calc)<br>1.09% @ 120min: 111 (pg/10^4 PDL cells)* / 1.1 (ratio-calc)<br>1.72% @ 30min: 101 (pg/10^4 PDL cells)* / 1.7 (ratio-calc)
SP7 *(dynamic)*	0.005	Li et al. (2015)	5%	0.5h, 1h, 2h, 4h, 8h, 12h, 24h	uniaxial	increase (qPCR, GAPDH)	24h: 14.0 (ratio)*	increase followed by plateau (WB, GAPDH)	12h…24h: 1.6 (rel)* / 4.2 (ratio-calc)
	0.1	Yang et al. (2018)	10%	24h	equibiaxial	increase (qPCR, GAPDH)	1.7 (FC)*	n.g.	n.g.
		Chang et al. (2017)	12%	72h	equibiaxial	n.g.	n.g.	increase (WB, β-actin)	72h: 26 (ratio)*
		Wang et al. (2019b)	12%	qPCR for 12h, 24h, 48h; WB for 24h, 48h	equibiaxial	increase followed by plateau (qPCR, β-actin)	24h...48h: 1.5 (FC)†	increase (WB, GAPDH)	48h: no quantitative information given
	0.5	Tang et al. (2012)	0.3%	3h, 6h, 12h, 24h	uniaxial	increase (qPCR, GAPDH)	24h: 15.4 (rel)* / 8.6 (ratio-calc)	increase (WB, GAPDH)	24h: 1.2 (rel)* / 1.4 (ratio-calc)
		Li et al. (2013)	10%	12h, 24h, 48h	equibiaxial	decrease (qPCR, GAPDH)	48h: 0.1 (FC)*	temporary increase (WB, GAPDH)	24h: 0.5 (rel)* / 1.3 (ratio-calc)
		Li et al. (2014)	10%	24h	equibiaxial	increase (qPCR, GAPDH)	2.7 (rel)* / 5.7 (ratio)*	increase (WB, GAPDH)	0.05 (rel)* / 1.3 (ratio-calc)
		Ren et al. (2015)	10%	1h, 3h, 6h, 12h, 18h, 24h	equibiaxial	increase (qPCR, GAPDH)	12h: 4.3 (FC)*	n.g.	n.g.
		Yu et al. (2018)	12%	24h, 48h, 72h	equibiaxial	increase (qPCR, ACTB)	72h: 3.3 (FC)*	increase (WB, β-actin)	no quantitative information given
	1.0	Wang et al. (2019a)	10%	12h	equibiaxial	increase (qPCR, GAPDH)	4.5 (FC)*	n.g.	n.g.

Reference

Abiko, Y., Shimizu, N., Yamaguchi, M., Suzuki, H., and Takiguchi, H. (1998). Effect of aging on functional changes of periodontal tissue cells. *Ann. Periodontol.* 3(1)**,** 350-369. doi: 10.1902/annals.1998.3.1.350.

Agarwal, S., Long, P., Seyedain, A., Piesco, N., Shree, A., and Gassner, R. (2003). A central role for the nuclear factor-κB pathway in anti-inflammatory and proinflammatory actions of mechanical strain. *FASEB J.* 17(8)**,** 899-901. doi: 10.1096/fj.02-0901fje.

Chang, M., Lin, H., Fu, H., Wang, B., Han, G., and Fan, M. (2017). MicroRNA-195-5p regulates osteogenic differentiation of periodontal ligament cells under mechanical loading. *J. Cell. Physiol.* 232(12)**,** 3762-3774. doi: 10.1002/jcp.25856.

Chang, M., Lin, H., Luo, M., Wang, J., and Han, G. (2015). Integrated miRNA and mRNA expression profiling of tension force-induced bone formation in periodontal ligament cells. *In Vitro Cell. Dev. Biol. Anim.* 51(8)**,** 797-807. doi: 10.1007/s11626-015-9892-0.

Chen, Y., Mohammed, A., Oubaidin, M., Evans, C.A., Zhou, X., Luan, X., et al. (2015). Cyclic stretch and compression forces alter microRNA-29 expression of human periodontal ligament cells. *Gene* 566(1)**,** 13-17. doi: 10.1016/j.gene.2015.03.055.

Chen, Y.J., Shie, M.Y., Hung, C.J., Wu, B.C., Liu, S.L., Huang, T.H., et al. (2014). Activation of focal adhesion kinase induces extracellular signal-regulated kinase-mediated osteogenesis in tensile force-subjected periodontal ligament fibroblasts but not in osteoblasts. *J. Bone Miner. Metab.* 32(6)**,** 671-682. doi: 10.1007/s00774-013-0549-3.

Chiba, M., and Mitani, H. (2004). Cytoskeletal changes and the system of regulation of alkaline phosphatase activity in human periodontal ligament cells induced by mechanical stress. *Cell Biochem. Funct.* 22(4)**,** 249-256. doi: 10.1002/cbf.1097.

Cho, J.H., Lee, S.K., Lee, J.W., and Kim, E.C. (2010). The role of heme oxygenase-1 in mechanical stress- and lipopolysaccharide-induced osteogenic differentiation in human periodontal ligament cells. *Angle Orthod.* 80(4)**,** 552-559. doi: 10.2319/091509-520.1.

Fujihara, C., Yamada, S., Ozaki, N., Takeshita, N., Kawaki, H., Takano-Yamamoto, T., et al. (2010). Role of mechanical stress-induced glutamate signaling-associated molecules in cytodifferentiation of periodontal ligament cells. *J. Biol. Chem.* 285(36)**,** 28286-28297. doi: 10.1074/jbc.M109.097303.

He, Y., Macarak, E.J., Korostoff, J.M., and Howard, P.S. (2004). Compression and tension: differential effects on matrix accumulation by periodontal ligament fibroblasts in vitro. *Connect Tissue Res.* 45(1)**,** 28-39. doi: 10.1080/03008200490278124.

He, Y., Xu, H., Xiang, Z., Yu, H., Xu, L., Guo, Y., et al. (2019). YAP regulates periodontal ligament cell differentiation into myofibroblast interacted with RhoA/ROCK pathway. *J. Cell. Physiol.* 234(4)**,** 5086-5096. doi: 10.1002/jcp.27312.

Jacobs, C., Grimm, S., Ziebart, T., Walter, C., and Wehrbein, H. (2013). Osteogenic differentiation of periodontal fibroblasts is dependent on the strength of mechanical strain. *Arch. Oral Biol.* 58(7)**,** 896-904. doi: 10.1016/j.archoralbio.2013.01.009.

Jacobs, C., Schramm, S., Dirks, I., Walter, C., Pabst, A., Meila, D., et al. (2018). Mechanical loading increases pro-inflammatory effects of nitrogen-containing bisphosphonate in human periodontal fibroblasts. *Clin. Oral Investig.* 22(2)**,** 901-907. doi: 10.1007/s00784-017-2168-1.

Jacobs, C., Walter, C., Ziebart, T., Dirks, I., Schramm, S., Grimm, S., et al. (2015). Mechanical loading influences the effects of bisphosphonates on human periodontal ligament fibroblasts. *Clin. Oral Investig.* 19(3)**,** 699-708. doi: 10.1007/s00784-014-1284-4.

Jacobs, C., Walter, C., Ziebart, T., Grimm, S., Meila, D., Krieger, E., et al. (2014). Induction of IL-6 and MMP-8 in human periodontal fibroblasts by static tensile strain. *Clin. Oral Investig.* 18(3)**,** 901-908. doi: 10.1007/s00784-013-1032-1.

Jiang, Z., and Hua, Y. (2016). Hydrogen sulfide promotes osteogenic differentiation of human periodontal ligament cells via p38-MAPK signaling pathway under proper tension stimulation. *Arch. Oral Biol.* 72**,** 8-13. doi: 10.1016/j.archoralbio.2016.08.008.

Kaku, M., Yamamoto, T., Yashima, Y., Izumino, J., Kagawa, H., Ikeda, K., et al. (2019). Acetaminophen reduces apical root resorption during orthodontic tooth movement in rats. *Arch. Oral Biol.* 102**,** 83-92. doi: 10.1016/j.archoralbio.2019.04.002.

Kanzaki, H., Chiba, M., Sato, A., Miyagawa, A., Arai, K., Nukatsuka, S., et al. (2006). Cyclical tensile force on periodontal ligament cells inhibits osteoclastogenesis through OPG induction. *J. Dent. Res.* 85(5)**,** 457-462. doi: 10.1177/154405910608500512.

Kanzaki, H., Wada, S., Yamaguchi, Y., Katsumata, Y., Itohiya, K., Fukaya, S., et al. (2019). Compression and tension variably alter Osteoprotegerin expression via miR-3198 in periodontal ligament cells. *BMC Mol. Cell Biol.* 20(1)**,** 6. doi: 10.1186/s12860-019-0187-2.

Kim, H.J., Choi, Y.S., Jeong, M.J., Kim, B.O., Lim, S.H., Kim, D.K., et al. (2007). Expression of UNCL during development of periodontal tissue and response of periodontal ligament fibroblasts to mechanical stress in vivo and in vitro. *Cell Tissue Res.* 327(1)**,** 25-31. doi: 10.1007/s00441-006-0304-3.

Konstantonis, D., Papadopoulou, A., Makou, M., Eliades, T., Basdra, E., and Kletsas, D. (2014). The role of cellular senescence on the cyclic stretching-mediated activation of MAPK and ALP expression and activity in human periodontal ligament fibroblasts. *Exp. Gerontol.* 57**,** 175-180. doi: 10.1016/j.exger.2014.05.010.

Lee, S.I., Park, K.H., Kim, S.J., Kang, Y.G., Lee, Y.M., and Kim, E.C. (2012). Mechanical stress-activated immune response genes via Sirtuin 1 expression in human periodontal ligament cells. *Clin. Exp. Immunol.* 168(1)**,** 113-124. doi: 10.1111/j.1365-2249.2011.04549.x.

Lee, S.Y., Yoo, H.I., and Kim, S.H. (2015). CCR5-CCL Axis in PDL during Orthodontic Biophysical Force Application. *J. Dent. Res.* 94(12)**,** 1715-1723. doi: 10.1177/0022034515603926.

Li, L., Han, M., Li, S., Wang, L., and Xu, Y. (2013). Cyclic tensile stress during physiological occlusal force enhances osteogenic differentiation of human periodontal ligament cells via ERK1/2-Elk1 MAPK pathway. *DNA Cell Biol.* 32(9)**,** 488-497. doi: 10.1089/dna.2013.2070.

Li, L., Han, M.X., Li, S., Xu, Y., and Wang, L. (2014). Hypoxia regulates the proliferation and osteogenic differentiation of human periodontal ligament cells under cyclic tensile stress via mitogen-activated protein kinase pathways. *J. Periodontol.* 85(3)**,** 498-508. doi: 10.1902/jop.2013.130048.

Li, S., Zhang, H., Li, S., Yang, Y., Huo, B., and Zhang, D. (2015). Connexin 43 and ERK regulate tension-induced signal transduction in human periodontal ligament fibroblasts. *J. Orthop. Res.* 33(7)**,** 1008-1014. doi: 10.1002/jor.22830.

Liao, C., and Hua, Y. (2013). Effect of hydrogen sulphide on the expression of osteoprotegerin and receptor activator of NF-kappaB ligand in human periodontal ligament cells induced by tension-force stimulation. *Arch. Oral Biol.* 58(12)**,** 1784-1790. doi: 10.1016/j.archoralbio.2013.08.004.

Liu, J., Li, Q., Liu, S., Gao, J., Qin, W., Song, Y., et al. (2017). Periodontal Ligament Stem Cells in the Periodontitis Microenvironment Are Sensitive to Static Mechanical Strain. *Stem Cells Int.* 2017**,** 1380851. doi: 10.1155/2017/1380851.

Long, P., Hu, J., Piesco, N., Buckley, M., and Agarwal, S. (2001). Low magnitude of tensile strain inhibits IL-1beta-dependent induction of pro-inflammatory cytokines and induces synthesis of IL-10 in human periodontal ligament cells in vitro. *J. Dent. Res.* 80(5)**,** 1416-

1420. doi: 10.1177/00220345010800050601.
Long, P., Liu, F., Piesco, N.P., Kapur, R., and Agarwal, S. (2002). Signaling by mechanical strain involves transcriptional regulation of proinflammatory genes in human periodontal ligament cells in vitro. *Bone* 30(4), 547-552. doi: 10.1016/s8756-3282(02)00673-7.
Matsuda, N., Yokoyama, K., Takeshita, S., and Watanabe, M. (1998). Role of epidermal growth factor and its receptor in mechanical stress-induced differentiation of human periodontal ligament cells in vitro. *Arch. Oral Biol.* 43(12), 987-997. doi: 10.1016/s0003-9969(98)00079-x.
Monnouchi, S., Maeda, H., Fujii, S., Tomokiyo, A., Kono, K., and Akamine, A. (2011). The roles of angiotensin II in stretched periodontal ligament cells. *J. Dent. Res.* 90(2), 181-185. doi: 10.1177/0022034510382118.
Nazet, U., Schröder, A., Spanier, G., Wolf, M., Proff, P., and Kirschneck, C. (2020). Simplified method for applying static isotropic tensile strain in cell culture experiments with identification of valid RT-qPCR reference genes for PDL fibroblasts. *Eur. J. Orthod.* 42(4), 359-370. doi: 10.1093/ejo/cjz052.
Nemoto, T., Kajiya, H., Tsuzuki, T., Takahashi, Y., and Okabe, K. (2010). Differential induction of collagens by mechanical stress in human periodontal ligament cells. *Arch. Oral Biol.* 55(12), 981-987. doi: 10.1016/j.archoralbio.2010.08.004.
Ngan, P., Saito, S., Saito, M., Lanese, R., Shanfeld, J., and Davidovitch, Z. (1990). The interactive effects of mechanical stress and interleukin-1 beta on prostaglandin E and cyclic AMP production in human periodontal ligament fibroblasts in vitro: comparison with cloned osteoblastic cells of mouse (MC3T3-E1). *Arch. Oral Biol.* 35(9), 717-725. doi: 10.1016/0003-9969(90)90094-Q.
Nogueira, A.V., Nokhbehsaim, M., Eick, S., Bourauel, C., Jäger, A., Jepsen, S., et al. (2014). Biomechanical loading modulates proinflammatory and bone resorptive mediators in bacterial-stimulated PDL cells. *Mediators Inflamm.* 2014, 425421. doi: 10.1155/2014/425421.
Nokhbehsaim, M., Deschner, B., Bourauel, C., Reimann, S., Winter, J., Rath, B., et al. (2011). Interactions of enamel matrix derivative and biomechanical loading in periodontal regenerative healing. *J. Periodontol.* 82(12), 1725-1734. doi: 10.1902/jop.2011.100678.
Nokhbehsaim, M., Deschner, B., Winter, J., Bourauel, C., Jäger, A., Jepsen, S., et al. (2012). Anti-inflammatory effects of EMD in the presence of biomechanical loading and interleukin-1β in vitro. *Clin. Oral Investig.* 16(1), 275-283. doi: 10.1007/s00784-010-0505-8.
Nokhbehsaim, M., Deschner, B., Winter, J., Reimann, S., Bourauel, C., Jepsen, S., et al. (2010). Contribution of orthodontic load to inflammation-mediated periodontal destruction. *J. Orofac. Orthop.* 71(6), 390-402. doi: 10.1007/s00056-010-1031-7.
Ohzeki, K., Yamaguchi, M., Shimizu, N., and Abiko, Y. (1999). Effect of cellular aging on the induction of cyclooxygenase-2 by mechanical stress in human periodontal ligament cells. *Mech. Ageing Dev.* 108(2), 151-163. doi: 10.1016/s0047-6374(99)00006-8.
Papadopoulou, A., Todaro, A., Eliades, T., and Kletsas, D. (2019). Effect of hyperglycaemic conditions on the response of human periodontal ligament fibroblasts to mechanical stretching. *Eur. J. Orthod.* doi: 10.1093/ejo/cjz051.
Qin, J., and Hua, Y. (2016). Effects of hydrogen sulfide on the expression of alkaline phosphatase, osteocalcin and collagen type I in human periodontal ligament cells induced by tension force stimulation. *Mol. Med. Rep.* 14(4), 3871-3877. doi: 10.3892/mmr.2016.5680.
Ren, D., Wei, F., Hu, L., Yang, S., Wang, C., and Yuan, X. (2015). Phosphorylation of Runx2, induced by cyclic mechanical tension via ERK1/2 pathway, contributes to osteodifferentiation of human periodontal ligament fibroblasts. *J. Cell. Physiol.* 230(10), 2426-2436. doi: 10.1002/jcp.24972.
Ritter, N., Mussig, E., Steinberg, T., Kohl, A., Komposch, G., and Tomakidi, P. (2007). Elevated expression of genes assigned to NF-kappaB and apoptotic pathways in human periodontal ligament fibroblasts following mechanical stretch. *Cell Tissue Res.* 328(3), 537-548. doi: 10.1007/s00441-007-0382-x.
Shen, T., Qiu, L., Chang, H., Yang, Y., Jian, C., Xiong, J., et al. (2014). Cyclic tension promotes osteogenic differentiation in human periodontal ligament stem cells. *Int. J. Clin. Exp. Pathol.* 7(11), 7872-7880.
Shimizu, N., Goseki, T., Yamaguchi, M., Iwasawa, T., Takiguchi, H., and Abiko, Y. (1997). In vitro cellular aging stimulates interleukin-1 beta production in stretched human periodontal-ligament-derived cells. *J. Dent. Res.* 76(7), 1367-1375. doi: 10.1177/00220345970760070601.
Shimizu, N., Ozawa, Y., Yamaguchi, M., Goseki, T., Ohzeki, K., and Abiko, Y. (1998). Induction of COX-2 expression by mechanical tension force in human periodontal ligament cells. *J. Periodontol.* 69(6), 670-677. doi: 10.1902/jop.1998.69.6.670.
Shimizu, N., Yamaguchi, M., Goseki, T., Ozawa, Y., Saito, K., Takiguchi, H., et al. (1994). Cyclic-tension force stimulates interleukin-1 beta production by human periodontal ligament cells. *J. Periodontal Res.* 29(5), 328-333. doi: 10.1111/j.1600-0765.1994.tb01230.x.
Shimizu, N., Yamaguchi, M., Goseki, T., Shibata, Y., Takiguchi, H., Iwasawa, T., et al. (1995). Inhibition of prostaglandin E2 and interleukin 1-beta production by low-power laser irradiation in stretched human periodontal ligament cells. *J. Dent. Res.* 74(7), 1382-1388. doi: 10.1177/00220345950740071001.
Spencer, A.Y., and Lallier, T.E. (2009). Mechanical tension alters semaphorin expression in the periodontium. *J. Periodontol.* 80(10), 1665-1673. doi: 10.1902/jop.2009.090212.
Sun, C., Chen, L., Shi, X., Cao, Z., Hu, B., Yu, W., et al. (2016). Combined effects of proinflammatory cytokines and intermittent cyclic mechanical strain in inhibiting osteogenicity in human periodontal ligament cells. *Cell Biol. Int.* 40(9), 999-1007. doi: 10.1002/cbin.10641.
Sun, C., Liu, F., Cen, S., Chen, L., Wang, Y., Sun, H., et al. (2017). Tensile strength suppresses the osteogenesis of periodontal ligament cells in inflammatory microenvironments. *Mol. Med. Rep.* 16(1), 666-672. doi: 10.3892/mmr.2017.6644.
Suzuki, R., Nemoto, E., and Shimauchi, H. (2014). Cyclic tensile force up-regulates BMP-2 expression through MAP kinase and COX-2/PGE2 signaling pathways in human periodontal ligament cells. *Exp. Cell Res.* 323(1), 232-241. doi: 10.1016/j.yexcr.2014.02.013.
Takano, M., Yamaguchi, M., Nakajima, R., Fujita, S., Kojima, T., and Kasai, K. (2009). Effects of relaxin on collagen type I released by stretched human periodontal ligament cells. *Orthod. Craniofac. Res.* 12(4), 282-288. doi: 10.1111/j.1601-6343.2009.01463.x.
Tang, N., Zhao, Z., Zhang, L., Yu, Q., Li, J., Xu, Z., et al. (2012). Up-regulated osteogenic transcription factors during early response of human periodontal ligament stem cells to cyclic tensile strain. *Arch. Med. Sci.* 8(3), 422-430. doi: 10.5114/aoms.2012.28810.
Tsuji, K., Uno, K., Zhang, G.X., and Tamura, M. (2004). Periodontal ligament cells under intermittent tensile stress regulate mRNA expression of osteoprotegerin and tissue inhibitor of matrix metalloprotease-1 and -2. *J. Bone Miner. Metab.* 22(2), 94-103. doi: 10.1007/s00774-003-0456-0.
Wada, S., Kanzaki, H., Narimiya, T., and Nakamura, Y. (2017). Novel device for application of continuous mechanical tensile strain to mammalian cells. *Biol. Open* 6(4), 518-524. doi: 10.1242/bio.023671.
Wang, H., Feng, C., Jin, Y., Tan, W., and Wei, F. (2019a). Identification and characterization of circular RNAs involved in mechanical force-induced periodontal ligament stem cells. *J. Cell. Physiol.* 234(7), 10166-10177. doi: 10.1002/jcp.27686.

Wang, Y., Hu, B., Hu, R., Tong, X., Zhang, M., Xu, C., et al. (2019b). TAZ contributes to osteogenic differentiation of periodontal ligament cells under tensile stress. *J. Periodontal Res.* doi: 10.1111/jre.12698.

Wang, Y., Li, Y., Fan, X., Zhang, Y., Wu, J., and Zhao, Z. (2011). Early proliferation alteration and differential gene expression in human periodontal ligament cells subjected to cyclic tensile stress. *Arch. Oral Biol.* 56(2)**,** 177-186. doi: 10.1016/j.archoralbio.2010.09.009.

Wei, F., Liu, D., Feng, C., Zhang, F., Yang, S., Hu, Y., et al. (2015). microRNA-21 mediates stretch-induced osteogenic differentiation in human periodontal ligament stem cells. *Stem Cells Dev.* 24(3)**,** 312-319. doi: 10.1089/scd.2014.0191.

Wei, F.L., Wang, J.H., Ding, G., Yang, S.Y., Li, Y., Hu, Y.J., et al. (2014). Mechanical force-induced specific MicroRNA expression in human periodontal ligament stem cells. *Cells Tissues Organs* 199(5-6)**,** 353-363. doi: 10.1159/000369613.

Wu, Y., Ou, Y., Liao, C., Liang, S., and Wang, Y. (2019). High-throughput sequencing analysis of the expression profile of microRNAs and target genes in mechanical force-induced osteoblastic/cementoblastic differentiation of human periodontal ligament cells. *Am. J. Transl. Res.* 11(6)**,** 3398-3411.

Yamaguchi, M., and Shimizu, N. (1994). Identification of factors mediating the decrease of alkaline phosphatase activity caused by tension-force in periodontal ligament cells. *Gen. Pharmacol.* 25(6)**,** 1229-1235. doi: 10.1016/0306-3623(94)90142-2.

Yamaguchi, M., Shimizu, N., Goseki, T., Shibata, Y., Takiguchi, H., Iwasawa, T., et al. (1994). Effect of different magnitudes of tension force on prostaglandin E2 production by human periodontal ligament cells. *Arch. Oral Biol.* 39(10)**,** 877-884. doi: 10.1016/0003-9969(94)90019-1.

Yamaguchi, M., Shimizu, N., Shibata, Y., and Abiko, Y. (1996). Effects of different magnitudes of tension-force on alkaline phosphatase activity in periodontal ligament cells. *J. Dent. Res.* 75(3)**,** 889-894. doi: 10.1177/00220345960750030501.

Yamaguchi, N., Chiba, M., and Mitani, H. (2002). The induction of c-fos mRNA expression by mechanical stress in human periodontal ligament cells. *Arch. Oral Biol.* 47(6)**,** 465-471. doi: 10.1016/s0003-9969(02)00022-5.

Yang, S.Y., Wei, F.L., Hu, L.H., and Wang, C.L. (2016). PERK-eIF2alpha-ATF4 pathway mediated by endoplasmic reticulum stress response is involved in osteodifferentiation of human periodontal ligament cells under cyclic mechanical force. *Cell. Signal.* 28(8)**,** 880-886. doi: 10.1016/j.cellsig.2016.04.003.

Yang, Y., Wang, B.K., Chang, M.L., Wan, Z.Q., and Han, G.L. (2018). Cyclic Stretch Enhances Osteogenic Differentiation of Human Periodontal Ligament Cells via YAP Activation. *Biomed Res. Int.* 2018**,** 2174824. doi: 10.1155/2018/2174824.

Yang, Y., Yang, Y., Li, X., Cui, L., Fu, M., Rabie, A.B., et al. (2010). Functional analysis of core binding factor a1 and its relationship with related genes expressed by human periodontal ligament cells exposed to mechanical stress. *Eur. J. Orthod.* 32(6)**,** 698-705. doi: 10.1093/ejo/cjq010.

Yang, Y.Q., Li, X.T., Rabie, A.B., Fu, M.K., and Zhang, D. (2006). Human periodontal ligament cells express osteoblastic phenotypes under intermittent force loading in vitro. *Front. Biosci.* 11**,** 776-781. doi: 10.2741/1835.

Yu, W., Hu, B., Shi, X., Cao, Z., Ren, M., He, Z., et al. (2018). Nicotine inhibits osteogenic differentiation of human periodontal ligament cells under cyclic tensile stress through canonical Wnt pathway and alpha7 nicotinic acetylcholine receptor. *J. Periodontal Res.* 53(4)**,** 555-564. doi: 10.1111/jre.12545.

Zhao, D., Wu, Y., Zhuang, J., Xu, C., and Zhang, F. (2016). Activation of NLRP1 and NLRP3 inflammasomes contributed to cyclic stretch-induced pyroptosis and release of IL-1beta in human periodontal ligament cells. *Oncotarget* 7(42)**,** 68292-68302. doi: 10.18632/oncotarget.11944.

Zhuang, J., Wang, Y., Qu, F., Wu, Y., Zhao, D., and Xu, C. (2019). Gasdermin-d Played a Critical Role in the Cyclic Stretch-Induced Inflammatory Reaction in Human Periodontal Ligament Cells. *Inflammation* 42(2)**,** 548-558. doi: 10.1007/s10753-018-0912-6.
