# Supplementary material for: Effect of Tension on Human Periodontal Ligament Cells: Systematic Review and Network Analysis
Source: Front Bioeng Biotechnol. 2021 Aug 27;9:695053. doi: 10.3389/fbioe.2021.695053 (PMC8429507; doi:10.3389/fbioe.2021.695053)
Supplement: Supplementary file 2 [file DataSheet2.PDF]

Supplement 2: Extracted data from included studies

Tabulated were the study citation and extracted information on the cells use (age/gender of donors, tooth type, isolation method, passages and cell density used in the experiments), force-related information ("dynamic"/"static" and "equibiaxial"/"uniaxial"; its duration, frequency of exposure, magnitude, and the device used), and the genes, proteins and/or metaoblites analyzed includng the official gene symbol, methods applied to measure their expression and data on the expression pattern (Figure 2) and the peak expression data.

Reference	Gene/ Analyte[a]	Official gene symbol / abbreviation[b]	Cell (age/gender of donors, tooth type, isolation method, passages used, cell density)[a,c]	Force type (stat./ dyn.)[a]	Force duration and frequency[d]	Force magnitude[a]	Force apparatus[a]	Force type: equibiaxial or uniaxial[e]	Gene expression: Increase, decrease, no change (method w/ reference gene); Methods: qPCR, sqPCR, Northern blot[f]	Gene expression: When it reaches peak and peak's magnitude (fold change; times or ratio; unclear = ?)[f]	Protein expression: Increase, decrease, no change (method w/ reference); Methods: ELISA, WB, RIA, EMSA, IF[f]	Protein expression: When it reaches peak and peak's magnitude (times or ratio; unclear = ?)[f]
Abiko et al. (1998)	COX1	*PTGS1*	HPDLF (18/n.g., 19/n.g., 23/n.g., PM, exp, P5-6, P18-20, 5×10^3)	dynamic	**0.1Hz** (6cyc/min) for 1d, 3d, 5d	18%	Flexercell Strain Unit + Flexcell Corp. plate + vacuum (Yamaguchi et al, 1997)	equibiaxial	no change (sqPCR, GAPDH)	only day 3 reported (no quantitative information is given)	n.g.	n.g.
Abiko et al. (1998)	COX2	*PTGS2*	HPDLF (18/n.g., 19/n.g., 23/n.g., PM, exp, P5-6, P18-20, 5×10^3)	dynamic	**0.1Hz** (6cyc/min) for 1d, 3d, 5d	18%	Flexercell Strain Unit + Flexcell Corp. plate + vacuum (Yamaguchi et al, 1997)	equibiaxial	increase (sqPCR, GAPDH)	only day 3 reported (no quantitative information is given)	n.g.	n.g.
Abiko et al. (1998)	ICE	*CASP1*	HPDLF (18/n.g., 19/n.g., 23/n.g., PM, exp, P5-6, P18-20, 5×10^3)	dynamic	**0.1Hz** (6cyc/min) for 1d, 3d, 5d	18%	Flexercell Strain Unit + Flexcell Corp. plate + vacuum (Yamaguchi et al, 1997)	equibiaxial	no change (sqPCR, GAPDH)	only day 3 reported (no quantitative information is given)	n.g.	n.g.
Abiko et al. (1998)	IL-1β	*IL1B*	HPDLF (18/n.g., 19/n.g., 23/n.g., PM, exp, P5-6, P18-20, 5×10^3)	dynamic	**0.1Hz** (6cyc/min) for 1d, 3d, 5d	18%	Flexercell Strain Unit + Flexcell Corp. plate + vacuum (Yamaguchi et al, 1997)	equibiaxial	n.g.	n.g.	"young cells" (P5-6): increase followed by plateau (ELISA)<br>"old cells" (P18-20): increase (ELISA)	"young cells" @ 3d…5d: 40.15 (ng/10^6 cellls) / 2.1 (ratio-calc)<br>"old cells" @ 5d: 58.10 (ng/10^6 cellls) / 2.8 (ratio-calc)
Abiko et al. (1998)	PA	*PLAT; PLAU*	HPDLF (18/n.g., 19/n.g., 23/n.g., PM, exp, P5-6, P18-20, 5×10^3)	dynamic	**0.1Hz** (6cyc/min) for 1d, 3d, 5d	18%	Flexercell Strain Unit + Flexcell Corp. plate + vacuum (Yamaguchi et al, 1997)	equibiaxial	n.g.	n.g.	"young cells" (P5-6): increase (ELISA)<br>"old cells" (P18-20): increase (ELISA)	"young cells" @ 5d: 7.90 (ng/10^6 cellls) / 2.1 (ratio-calc)<br>"old cells" @ 5d: 11.75 (ng/10^6 cellls) / 3.1 (ratio-calc)
Abiko et al. (1998)	PAI-1	*SERPINE1*	HPDLF (18/n.g., 19/n.g., 23/n.g., PM, exp, P5-6, P18-20, 5×10^3)	dynamic	**0.1Hz** (6cyc/min) for 1d, 3d, 5d	18%	Flexercell Strain Unit + Flexcell Corp. plate + vacuum (Yamaguchi et al, 1997)	equibiaxial	no change (sqPCR, GAPDH)	only day 3 reported (no quantitative information is given)	n.g.	n.g.
Abiko et al. (1998)	PGE_2	PGE_2	HPDLF (18/n.g., 19/n.g., 23/n.g., PM, exp, P5-6, P18-20, 5×10^3)	dynamic	**0.1Hz** (6cyc/min) for 1d, 3d, 5d	18%	Flexercell Strain Unit + Flexcell Corp. plate + vacuum (Yamaguchi et al, 1997)	equibiaxial	n.a.	n.a.	"young cells" (P5-6): increase (ELISA)<br>"old cells" (P18-20): increase (ELISA)	"young cells" @ 5d: 8.20 (ng/10^6 cellls) / 14.9 (ratio-calc)<br>"old cells" @ 5d: 12.25 (ng/10^6 cellls) / 18 (ratio-calc)
Abiko et al. (1998)	tPA	*PLAT*	HPDLF (18/n.g., 19/n.g., 23/n.g., PM, exp, P5-6, P18-20, 5×10^3)	dynamic	**0.1Hz** (6cyc/min) for 1d, 3d, 5d	18%	Flexercell Strain Unit + Flexcell Corp. plate + vacuum (Yamaguchi et al, 1997)	equibiaxial	increase (sqPCR, GAPDH)	only day 3 reported (no quantitative information is given)	n.g.	n.g.
Abiko et al. (1998)	uPA	*PLAU*	HPDLF (18/n.g., 19/n.g., 23/n.g., PM, exp, P5-6, P18-20, 5×10^3)	dynamic	**0.1Hz** (6cyc/min) for 1d, 3d, 5d	18%	Flexercell Strain Unit + Flexcell Corp. plate + vacuum (Yamaguchi et al, 1997)	equibiaxial	no change (sqPCR, GAPDH)	only day 3 reported (no quantitative information is given)	n.g.	n.g.
Agarwal et al. (2003)	COX2	*PTGS2*	HPDLF (18/F, 18/F, 22/M, M, exp, P6-12, 80% confluence)	dynamic	0.005Hz for 4h	3%, 6%, 8%	Flexercell Strain Unit + collagen type I-coated Bioflex II, six-well plates + vacuum	equibiaxial	increase (sqPCR; GAPDH)	6%: 7.6 (rel)†/ 2.2 (ratio-calc)	n.g.	n.g.
Agarwal et al. (2003)	COX2	*PTGS2*	HPDLF (18/F, 18/F, 22/M, M, exp, P6-12, 80% confluence)	dynamic	0.005Hz for 4h, 24h, 48h	15%	Flexercell Strain Unit + collagen type I-coated Bioflex II, six-well plates + vacuum	equibiaxial	increase (sqPCR; GAPDH)	48h: 116.8 (rel)† / 31.8 (ratio-calc)	n.g.	n.g.
Agarwal et al. (2003)	I-κBβ	*NFKBIB*	HPDLF (18/F, 18/F, 22/M, M, exp, P6-12, 80% confluence)	dynamic	0.005Hz for 30min	6%	Flexercell Strain Unit + collagen type I-coated Bioflex II, six-well plates + vacuum	equibiaxial	n.g.	n.g.	n.g.	n.g.
Agarwal et al. (2003)	I-κBβ	*NFKBIB*	HPDLF (18/F, 18/F, 22/M, M, exp, P6-12, 80% confluence)	dynamic	0.005Hz for 30min	15%	Flexercell Strain Unit + collagen type I-coated Bioflex II, six-well plates + vacuum	equibiaxial	n.g.	n.g.	n.g.	n.g.
Agarwal et al. (2003)	NF-kB	*NFKB1*	HPDLF (18/F, 18/F, 22/M, M, exp, P6-12, 80% confluence)	dynamic	0.005Hz for 30min, 60min, 90min, 120min	6%	Flexercell Strain Unit + collagen type I-coated Bioflex II, six-well plates + vacuum	equibiaxial	n.g.	n.g.	n.g.	n.g.
Agarwal et al. (2003)	NF-kB	*NFKB1*	HPDLF (18/F, 18/F, 22/M, M, exp, P6-12, 80% confluence)	dynamic	0.005Hz for 30min, 60min, 120min	15%	Flexercell Strain Unit + collagen type I-coated Bioflex II, six-well plates + vacuum	equibiaxial	n.g.	n.g.	n.g.	n.g.
Agarwal et al. (2003)	PGE_2	PGE_2	HPDLF (18/F, 18/F, 22/M, M, exp, P6-12, 80% confluence)	dynamic	0.005Hz for 24h	1%, 2%, 3%, 4%, 5%, 6%, 7%, 8%, 10%, 12.5%, 15%, 18%	Flexercell Strain Unit + collagen type I-coated Bioflex II, six-well plates + vacuum	equibiaxial	n.a.	n.a.	1%, 2%, 3%, 4%, 5%, 6%, 7%, 8%: no expression (RIA)<br>10%, 12.5%, 15%, 18%: increase (RIA)	18%: 135 (ng/10^6 cells)* / control n.g.
Agarwal et al. (2003)	PGE_2	PGE_2	HPDLF (18/F, 18/F, 22/M, M, exp, P6-12, 80% confluence)	dynamic	0.005Hz for 12h, 24h, 48h	15%	Flexercell Strain Unit + collagen type I-coated Bioflex II, six-well plates + vacuum	equibiaxial	n.a.	n.a.	increase followed by plateau (RIA)	24h…48h: 75.1† (?) / 15.6 (ratio-calc)
Arima et al. (2019)	DKK1	*DKK1*	HPDLC (3 donors: 22/F, 23/M, 25/F, M, exp, P4-P6, Subconfluence)	dynamic	0.5Hz for 24h	10%	STREX STB-140 + culture chambers (STREX Co., Osaka, Japan) coated with type-I collagen (Cell matrix I-P, Nitta Gelatin Inc., Osaka, Japan) + motor	uniaxial	donor 3D: increase (qPCR, β-actin)<br>donor 3S: increase (qPCR, β-actin)<br>donor 3U: increase (qPCR, β-actin)	donor 3D: 11.7 (FC)*<br>donor 3S: 1.7 (FC)*<br>donor 3U: 1.6 (FC)*	n.g.	n.g.
Arima et al. (2019)	RSPO2	*RSPO2*	HPDLC (3 donors: 22/F, 23/M, 25/F, M, exp, P4-P6, Subconfluence)	dynamic	0.5Hz for 24h	10%	STREX STB-140 + culture chambers (STREX Co., Osaka, Japan) coated with type-I collagen (Cell matrix I-P, Nitta Gelatin Inc., Osaka, Japan) + motor	uniaxial	donor 3D: increase (qPCR, β-actin)<br>donor 3S: increase (qPCR, β-actin)<br>donor 3U: increase (qPCR, β-actin)	donor 3D: 5.2 (FC)*<br>donor 3S: 2 (FC)*<br>donor 3U: 1.6 (FC)*	n.g.	n.g.

[a] Entry given as reported in the study.
[b] All official gene symbols come from the HUGO Gene Nomenclature Committee (HGNC; URL: https://www.genenames.org) after checking specificity of primers with Primer-BLAST.
[c] Gender/Sex of donors: "M" – male, "F" – female; Tooth type: "PM" – premolar, "M" – molar; Cell density: given in cells/well if not otherwise mentioned.
[d] Frequencies labeled bold orange were converted to hertz (Hz) according to its definition using the information reported in the study (in brackets)
[e] Force type deduced from the description of the force apparatus given by the authors.
[f] Gene and protein expression: 1. conclusion of change (increase, decrease…) was given according to the defined criteria in Figure 2; 2. different markers to describe the amount of change; † Information derived from figures using Engauge Digitizer; *Folds calculated by measuring the graphs, without using the Engauge Digitizer; No makers: Information derived from figures by description in the articles

Reference	Gene/ Analyte[a]	Official gene symbol / abbreviation[b]	Cell (age/gender of donors, tooth type, isolation method, passages used, cell density)[a,c]	Force type (stat./ dyn.)[a]	Force duration and frequency[d]	Force magnitude[a]	Force apparatus[a]	Force type: equibiaxial or uniaxial[e]	Gene expression: Increase, decrease, no change (method w/ reference gene); Methods: qPCR, sqPCR, Northern blot[f]	Gene expression: When it reaches peak and peak's magnitude (fold change; times or ratio; unclear = ?)[f]	Protein expression: Increase, decrease, no change (method w/ reference); Methods: ELISA, WB, RIA, EMSA, IF[f]	Protein expression: When it reaches peak and peak's magnitude (times or ratio; unclear = ?)[f]
Basdra et al. (1995)	Rab17	*RAB17*	HPDL-fibroblasts outgrowth (n.g./n.g., M, exp, P n.g., 4×10^5)	static	1h	2.5%	Petriperm dish + brass spheroidal convex template + brass weight	equibiaxial	n.g.	n.g.	increase (WB)	no quantitative information is given
Basdra et al. (1995)	rab3a	*RAB3A*	HPDL-fibroblasts outgrowth (n.g./n.g., M, exp, P n.g., 4×10^5)	static	1h	2.5%	Petriperm dish + brass spheroidal convex template + brass weight	equibiaxial	n.g.	n.g.	increase (WB)	no quantitative information is given
Basdra et al. (1995)	rab3b	*RAB3B*	HPDL-fibroblasts outgrowth (n.g./n.g., M, exp, P n.g., 4×10^5)	static	1h	2.5%	Petriperm dish + brass spheroidal convex template + brass weight	equibiaxial	n.g.	n.g.	increase (WB)	no quantitative information is given
Basdra et al. (1995)	rab6	*RAB6A*	HPDL-fibroblasts outgrowth (n.g./n.g., M, exp, P n.g., 4×10^5)	static	1h	2.5%	Petriperm dish + brass spheroidal convex template + brass weight	equibiaxial	n.g.	n.g.	increase (WB)	no quantitative information is given
Basdra et al. (1995)	rhoA	*RHOA*	HPDL-fibroblasts outgrowth (n.g./n.g., M, exp, P n.g., 4×10^5)	static	1h	2.5%	Petriperm dish + brass spheroidal convex template + brass weight	equibiaxial	n.g.	n.g.	decrease (WB)	no quantitative information is given
Basdra et al. (1996)	Vimentin	*VIM*	HPDL-fibroblasts outgrowth (n.g./n.g., M, exp, P n.g., 3×10^5)	static	12h	2.5%	Petriperm dish + spheroidal copper template + weight	equibiaxial	n.g.	n.g.	no change (WB)	no quantitative information is given
Basdra et al. (1996)	α-tubulin	*TUBA1C; TUBA3C; TUBA3D; TUBA4A*	HPDL-fibroblasts outgrowth (n.g./n.g., M, exp, P n.g., 3×10^5)	static	12h	2.5%	Petriperm dish + spheroidal copper template + weight	equibiaxial	n.g.	n.g.	no change (WB)	no quantitative information is given
Basdra et al. (1996)	β-tubulin	*TUBA1B*	HPDL-fibroblasts outgrowth (n.g./n.g., M, exp, P n.g., 3×10^5)	static	12h	2.5%	Petriperm dish + spheroidal copper template + weight	equibiaxial	n.g.	n.g.	no change (WB)	no quantitative information is given
Bolcato-Bellemin et al. (2000)	integrin α1	*ITGA1*	HPDL fibroblasts (n.g./F, M, exp, P4, 4×10^5)	static	12h	20kPa	Flexercell Strain Unit + Bioflex Culture Plate + vacuum (Carvalho et al 1996)	equibiaxial	no change (sqPCR, β-actin)	no change	n.g.	n.g.
Bolcato-Bellemin et al. (2000)	integrin α2	*ITGA2*	HPDL fibroblasts (n.g./F, M, exp, P4, 4×10^5)	static	12h	20kPa	Flexercell Strain Unit + Bioflex Culture Plate + vacuum (Carvalho et al 1996)	equibiaxial	no change (sqPCR, β-actin)	no change	n.g.	n.g.
Bolcato-Bellemin et al. (2000)	Integrin α3	*ITGA3*	HPDL fibroblasts (n.g./F, M, exp, P4, 4×10^5)	static	12h	20kPa	Flexercell Strain Unit + Bioflex Culture Plate + vacuum (Carvalho et al 1996)	equibiaxial	no change (sqPCR, β-actin)	no change	n.g.	n.g.
Bolcato-Bellemin et al. (2000)	Integrin α4	*ITGA4*	HPDL fibroblasts (n.g./F, M, exp, P4, 4×10^5)	static	12h	20kPa	Flexercell Strain Unit + Bioflex Culture Plate + vacuum (Carvalho et al 1996)	equibiaxial	no change (sqPCR, β-actin)	no change	n.g.	n.g.
Bolcato-Bellemin et al. (2000)	Integrin α5	*ITGA5*	HPDL fibroblasts (n.g./F, M, exp, P4, 4×10^5)	static	12h	20kPa	Flexercell Strain Unit + Bioflex Culture Plate + vacuum (Carvalho et al 1996)	equibiaxial	decrease (sqPCR, β-actin)	0.283 (rel) / 0.5 (ratio-calc)	n.g.	n.g.
Bolcato-Bellemin et al. (2000)	Integrin α6	*ITGA6*	HPDL fibroblasts (n.g./F, M, exp, P4, 4×10^5)	static	12h	20kPa	Flexercell Strain Unit + Bioflex Culture Plate + vacuum (Carvalho et al 1996)	equibiaxial	increase (sqPCR, β-actin)	0.247 (rel) / 2.2 (ratio-calc)	n.g.	n.g.
Bolcato-Bellemin et al. (2000)	Integrin αv	*ITGAV*	HPDL fibroblasts (n.g./F, M, exp, P4, 4×10^5)	static	12h	20kPa	Flexercell Strain Unit + Bioflex Culture Plate + vacuum (Carvalho et al 1996)	equibiaxial	no change (sqPCR, β-actin)	no change	n.g.	n.g.
Bolcato-Bellemin et al. (2000)	Integrin β1	*ITGB1*	HPDL fibroblasts (n.g./F, M, exp, P4, 4×10^5)	static	12h	20kPa	Flexercell Strain Unit + Bioflex Culture Plate + vacuum (Carvalho et al 1996)	equibiaxial	increase (sqPCR, β-actin)	0.360 (rel) / 3.3 (ratio-calc)	n.g.	n.g.
Bolcato-Bellemin et al. (2000)	Integrin β3	*ITGB3*	HPDL fibroblasts (n.g./F, M, exp, P4, 4×10^5)	static	12h	20kPa	Flexercell Strain Unit + Bioflex Culture Plate + vacuum (Carvalho et al 1996)	equibiaxial	no change (sqPCR, β-actin)	no change	n.g.	n.g.
Bolcato-Bellemin et al. (2000)	Integrin β4	*ITGB4*	HPDL fibroblasts (n.g./F, M, exp, P4, 4×10^5)	static	12h	20kPa	Flexercell Strain Unit + Bioflex Culture Plate + vacuum (Carvalho et al 1996)	equibiaxial	no change (sqPCR, β-actin)	no change	n.g.	n.g.
Bolcato-Bellemin et al. (2000)	MMP-1	*MMP1*	HPDL fibroblasts (n.g./F, M, exp, P4, 4×10^5)	static	12h	20kPa	Flexercell Strain Unit + Bioflex Culture Plate + vacuum (Carvalho et al 1996)	equibiaxial	increase (sqPCR, β-actin)	0.250 (rel) / 5 (ratio-calc)	n.g.	n.g.
Bolcato-Bellemin et al. (2000)	MMP-2	*MMP2*	HPDL fibroblasts (n.g./F, M, exp, P4, 4×10^5)	static	12h	20kPa	Flexercell Strain Unit + Bioflex Culture Plate + vacuum (Carvalho et al 1996)	equibiaxial	increase (sqPCR, β-actin)	0.210 (rel) / 2.1 (ratio-calc)	n.g.	n.g.
Bolcato-Bellemin et al. (2000)	MMP-9	*MMP9*	HPDL fibroblasts (n.g./F, M, exp, P4, 4×10^5)	static	12h	20kPa	Flexercell Strain Unit + Bioflex Culture Plate + vacuum (Carvalho et al 1996)	equibiaxial	no expression (sqPCR, β-actin)	no expression	n.g.	n.g.
Bolcato-Bellemin et al. (2000)	MT1-MMP	*MMP14*	HPDL fibroblasts (n.g./F, M, exp, P4, 4×10^5)	static	12h	20kPa	Flexercell Strain Unit + Bioflex Culture Plate + vacuum (Carvalho et al 1996)	equibiaxial	no change (sqPCR, β-actin)	no change	n.g.	n.g.
Bolcato-Bellemin et al. (2000)	TIMP-1	*TIMP1*	HPDL fibroblasts (n.g./F, M, exp, P4, 4×10^5)	static	12h	20kPa	Flexercell Strain Unit + Bioflex Culture Plate + vacuum (Carvalho et al 1996)	equibiaxial	increase (sqPCR, β-actin)	0.075 (rel) / 1.5 (ratio-calc)	n.g.	n.g.
Bolcato-Bellemin et al. (2000)	TIMP-2	*TIMP2*	HPDL fibroblasts (n.g./F, M, exp, P4, 4×10^5)	static	12h	20kPa	Flexercell Strain Unit + Bioflex Culture Plate + vacuum (Carvalho et al 1996)	equibiaxial	increase (sqPCR, β-actin)	0.291 (rel) / 5 (ratio-calc)	n.g.	n.g.
Bolcato-Bellemin et al. (2000)	TIMP-3	*TIMP3*	HPDL fibroblasts (n.g./F, M, exp, P4, 4×10^5)	static	12h	20kPa	Flexercell Strain Unit + Bioflex Culture Plate + vacuum (Carvalho et al 1996)	equibiaxial	no change (sqPCR, β-actin)	no change	n.g.	n.g.
Chang et al. (2015)	ALP	*ALPP*	HPDLCs (n.g./n.g., PM, exp, P3, 1.0×10^6)	dynamic	**0.1Hz** (6cyc/min: 5s on and 5s off) for 6h, 12h, 24h, 48h, 72h	12%	Flexercell FX-4000 Strain Unit + 6-well BioFlex plates coated with type I collagen + vacuum	equibiaxial	increase (qPCR, GAPDH)	72h: 5.3 (ratio)*	n.g.	n.g.
Chang et al. (2015)	ARRAY	ARRAY	HPDLCs (n.g./n.g., PM, exp, P3, 1.0×10^6)	dynamic	**0.1Hz** (6cyc/min: 5s on and 5s off) for 6h, 12h, 24h, 48h, 72h	12%	Flexercell FX-4000 Strain Unit + 6-well BioFlex plates coated with type I collagen + vacuum	equibiaxial	mRNA: Agilent Whole Genome Oligo Microarrays (Agilent, Santa Clara, CA) miRNA: Exiqon miRNA Array (Exiqon, Vedbaek, Denmark)		n.g.	n.g.
Chang et al. (2015)	CREB1	*CREB1*	HPDLCs (n.g./n.g., PM, exp, P3, 1.0×10^6)	dynamic	**0.1Hz** (6cyc/min: 5s on and 5 s off) for 72h	12%	Flexercell FX-4000 Strain Unit + 6-well BioFlex plates coated with type I collagen + vacuum	equibiaxial	increase (qPCR, GAPDH)	72h: 1.8 (ratio)*	n.g.	n.g.
Chang et al. (2015)	FGF2	*FGF2*	HPDLCs (n.g./n.g., PM, exp, P3, 1.0×10^6)	dynamic	**0.1Hz** (6cyc/min: 5s on and 5s off) for 72h	12%	Flexercell FX-4000 Strain Unit + 6-well BioFlex plates coated with type I collagen + vacuum	equibiaxial	increase (qPCR, GAPDH)	72h: 2.2 (ratio)*	n.g.	n.g.

[a] Entry given as reported in the study.
[b] All official gene symbols come from the HUGO Gene Nomenclature Committee (HGNC; URL: https://www.genenames.org) after checking specificity of primers with Primer-BLAST.
[c] Gender/Sex of donors: "M" – male, "F" – female; Tooth type: "PM" – premolar, "M" – molar; Cell density: given in cells/well if not otherwise mentioned.
[d] Frequencies labeled bold orange were converted to hertz (Hz) according to its definition using the information reported in the study (in brackets)
[e] Force type deduced from the description of the force apparatus given by the authors.
[f] Gene and protein expression: 1. conclusion of change (increase, decrease…) was given according to the defined criteria in Figure 2; 2. different markers to describe the amount of change; † Information derived from figures using Engauge Digitizer; *Folds calculated by measuring the graphs, without using the Engauge Digitizer; No makers: Information derived from figures by description in the articles

Reference	Gene/ Analyte[a]	Official gene symbol / abbreviation[b]	Cell (age/gender of donors, tooth type, isolation method, passages used, cell density)[a,c]	Force type (stat./ dyn.)[a]	Force duration and frequency[d]	Force magnitude[a]	Force apparatus[a]	Force type: equibiaxial or uniaxial[e]	Gene expression: Increase, decrease, no change (method w/ reference gene); Methods: qPCR, sqPCR, Northern blot[f]	Gene expression: When it reaches peak and peak's magnitude (fold change; times or ratio; unclear = ?)[f]	Protein expression: Increase, decrease, no change (method w/ reference); Methods: ELISA, WB, RIA, EMSA, IF[f]	Protein expression: When it reaches peak and peak's magnitude (times or ratio; unclear = ?)[f]
Chang et al. (2015)	miR-424-5p<br>miR-1297<br>miR-3607-5p<br>miR-145-5p<br>miR-4328<br>miR-224-5p<br>miR-195-5p	*MIR424*<br>*MIR1297*<br>*miR-3607-5p*<br>*MIR145*<br>*MIR4328*<br>*MIR224*<br>*MIR195*	HPDLCs (n.g./n.g., PM, exp, P3, $1.0×10^6$)	dynamic	**0.1Hz** (6cyc/min: 5s on and 5s off) for 72h	12%	Flexercell FX-4000 Strain Unit + 6-well BioFlex plates coated with type I collagen + vacuum	equibiaxial	decrease (qPCR, U6 SnRNA)<br>decrease (qPCR, U6 SnRNA)<br>decrease (qPCR, U6 SnRNA)<br>decrease (qPCR, U6 SnRNA)<br>decrease (qPCR, U6 SnRNA)<br>decrease (qPCR, U6 SnRNA)<br>decrease (qPCR, U6 SnRNA)	miR-424-5p: 0.4 (ratio)*<br>miR-1297: 0.6 (ratio)*<br>miR-3607-5p: 0.2 (ratio)*<br>miR-145-5p: 0.3 (ratio)*<br>miR-4328: 0.8 (ratio)*<br>miR-224-5p: 0.4 (ratio)*<br>miR-195-5p: 0.6 (ratio)*	n.g.	n.g.
Chang et al. (2015)	OCN	*BGLAP*	HPDLCs (n.g./n.g., PM, exp, P3, $1.0×10^6$)	dynamic	**0.1Hz** (6cyc/min: 5s on and 5s off) for 6h, 12h, 24h, 48h, 72h	12%	Flexercell FX-4000 Strain Unit + 6-well BioFlex plates coated with type I collagen + vacuum	equibiaxial	increase (qPCR, GAPDH)	72h: 2 (ratio)*	n.g.	n.g.
Chang et al. (2017)	ALP	*ALPP*	HPDLCs (14-20/n.g., PM, dig, P<5, 80% confluence)	dynamic	0.1Hz for 24h, 48h, 72h	12%	Custom-made strain device Tension Plus System (Chang et al 2015; Wescott et al 2007) + flexible-bottomed culture plates (Flexcell)	equibiaxial	n.g.	n.g.	increase (PNPP)	72h: 0.8 (U/mg)* / 2.7 (ratio-calc)
Chang et al. (2017)	BMPR1A	*BMPR1A*	HPDLCs (14-20/n.g., PM, dig, P<5, 80% confluence)	dynamic	0.1Hz for 72h	12%	Custom-made strain device Tension Plus System (Chang et al 2015; Wescott et al 2007) + flexible-bottomed culture plates (Flexcell)	equibiaxial	n.g.	n.g.	increase (WB, β-actin)	72h: 3.3 (ratio)*
Chang et al. (2017)	FGF2	*FGF2*	HPDLCs (14-20/n.g., PM, dig, P<5, 80% confluence)	dynamic	0.1Hz for 72h	12%	Custom-made strain device Tension Plus System (Chang et al 2015; Wescott et al 2007) + flexible-bottomed culture plates (Flexcell)	equibiaxial	n.g.	n.g.	increase (WB, β-actin)	72h: 3.1 (ratio)*
Chang et al. (2017)	miR-195-5p	*MIR195*	HPDLCs (14-20/n.g., PM, dig, P<5, 80% confluence)	dynamic	0.1Hz for 24h, 48h, 72h	12%	Custom-made strain device Tension Plus System (Chang et al 2015; Wescott et al 2007) + flexible-bottomed culture plates (Flexcell)	equibiaxial	decrease (qPCR, U6 snRNA)	24h: 0.5 (FC)*	n.g.	n.g.
Chang et al. (2017)	OCN	*BGLAP*	HPDLCs (14-20/n.g., PM, dig, P<5, 80% confluence)	dynamic	0.1Hz for 72h	12%	Custom-made strain device Tension Plus System (Chang et al 2015; Wescott et al 2007) + flexible-bottomed culture plates (Flexcell)	equibiaxial	n.g.	n.g.	increase (WB, β-actin)	72h: 1.8 (ratio)*
Chang et al. (2017)	OPN	*SPP1*	HPDLCs (14-20/n.g., PM, dig, P<5, 80% confluence)	dynamic	0.1Hz for 72h	12%	Custom-made strain device Tension Plus System (Chang et al 2015; Wescott et al 2007) + flexible-bottomed culture plates (Flexcell)	equibiaxial	n.g.	n.g.	increase (WB, β-actin)	72h: 2.4 (ratio)*
Chang et al. (2017)	OSX	*SP7*	HPDLCs (14-20/n.g., PM, dig, P<5, 80% confluence)	dynamic	0.1Hz for 72h	12%	Custom-made strain device Tension Plus System (Chang et al 2015; Wescott et al 2007) + flexible-bottomed culture plates (Flexcell)	equibiaxial	n.g.	n.g.	increase (WB, β-actin)	72h: 26 (ratio)*
Chang et al. (2017)	Runx2	*RUNX2*	HPDLCs (14-20/n.g., PM, dig, P<5, 80% confluence)	dynamic	0.1Hz for 24h, 48h, 72h	12%	Custom-made strain device Tension Plus System (Chang et al 2015; Wescott et al 2007) + flexible-bottomed culture plates (Flexcell)	equibiaxial	increase (qPCR, GAPDH)	72h: 3 (FC)*	n.g.	n.g.
Chang et al. (2017)	WNT3A	*WNT3A*	HPDLCs (14-20/n.g., PM, dig, P<5, 80% confluence)	dynamic	0.1Hz for 72h	12%	Custom-made strain device Tension Plus System (Chang et al 2015; Wescott et al 2007) + flexible-bottomed culture plates (Flexcell)	equibiaxial	n.g.	n.g.	increase (WB, β-actin)	72h: 51 (ratio)*
Chen et al. (2014)	ALP	*ALPP*	HPDLs (n.g./n.g., M, dig, P3-9, 10^4 cells/mL)	static	sqPCR for 1d, 3d, 7d, 15d ELISA for 3d, 7d, 15d	-100 kPa (1Pa=1/100,000kg/cm², equal to a negative force of 101g/mm²)	"a tension incubator" (TI, Model 3618P; LabLine Instrument, Thermolyne Co., IL, USA) + 24-well plate + vacuum	equibiaxial	increase (?, actin)	7d: 0.5 (?)* / 2.1 (ratio-calc)	increase (ELISA)	15d: 0.2 (μM/μg DNA)* / 1.2 (ratio-calc)
Chen et al. (2014)	Collagen-1	*COL1A1; COL1A2*	HPDLs (n.g./n.g., M, dig, P3-9, 10^4 cells/mL)	static	1d, 3d, 7d, 15d	-100 kPa (1Pa=1/100,000kg/cm², equal to a negative force of 101g/mm²)	"a tension incubator" (TI, Model 3618P; LabLine Instrument, Thermolyne Co., IL, USA) + 24-well plate + vacuum	equibiaxial	no change (?, actin)		n.g.	n.g.
Chen et al. (2014)	ERK/p-ERK	MAPK3; MAPK1	HPDLs (n.g./n.g., M, dig, P3-9, 10^4 cells/mL)	static	3h, 6h, 12h, 24h	-100 kPa (1Pa=1/100,000kg/cm², equal to a negative force of 101g/mm²)	"a tension incubator" (TI, Model 3618P; LabLine Instrument, Thermolyne Co., IL, USA) + 24-well plate + vacuum	equibiaxial	n.g.	n.g.	p-ERK/ERK: increase (WB)	p-ERK/ERK: 24h: 0.6 (ratio)* / 2 (ratio-calc)
Chen et al. (2014)	FAK/p-FAK	PTK2	HPDLs (n.g./n.g., M, dig, P3-9, 10^4 cells/mL)	static	3h, 6h, 12h, 24h	-100 kPa (1Pa=1/100,000kg/cm², equal to a negative force of 101g/mm²)	"a tension incubator" (TI, Model 3618P; LabLine Instrument, Thermolyne Co., IL, USA) + 24-well plate + vacuum	equibiaxial	n.g.	n.g.	p-FAK/FAK: increase (WB)	p-FAK/FAK: 24h: 0.7 (ratio)* / 1.3 (ratio-calc)
Chen et al. (2014)	IL-1	*IL1B; IL1A*	HPDLs (n.g./n.g., M, dig, P3-9, 10^4 cells/mL)	static	1d, 3d, 7d, 15d	-100 kPa (1Pa=1/100,000kg/cm², equal to a negative force of 101g/mm²)	"a tension incubator" (TI, Model 3618P; LabLine Instrument, Thermolyne Co., IL, USA) + 24-well plate + vacuum	equibiaxial	increase (?, actin)	7d: 1.2 (?)* / 2.8 (ratio-calc)	n.g.	n.g.
Chen et al. (2014)	iNOS	*NOS2*	HPDLs (n.g./n.g., M, dig, P3-9, 10^4 cells/mL)	static	1d, 3d, 7d, 15d	-100 kPa (1Pa=1/100,000kg/cm², equal to a negative force of 101g/mm²)	"a tension incubator" (TI, Model 3618P; LabLine Instrument, Thermolyne Co., IL, USA) + 24-well plate + vacuum	equibiaxial	increase (?, actin)	7d: 1.2 (?)* / 2.4 (ratio-calc)	n.g.	n.g.
Chen et al. (2014)	OC	BGLAP	HPDLs (n.g./n.g., M, dig, P3-9, 10^4 cells/mL)	static	sqPCR for 1d, 3d, 7d, 15d ELISA for 7d, 15d	-100 kPa (1Pa=1/100,000kg/cm², equal to a negative force of 101g/mm²)	"a tension incubator" (TI, Model 3618P; LabLine Instrument, Thermolyne Co., IL, USA) + 24-well plate + vacuum	equibiaxial	increase (?, actin)	15d: 0.3 (?)* / 2.8 (ratio-calc)	increase (ELISA)	15d: 31.4 (pg/ml/cell)* / 1.3 (ratio-calc)

[a] Entry given as reported in the study.
[b] All official gene symbols come from the HUGO Gene Nomenclature Committee (HGNC; URL: https://www.genenames.org) after checking specificity of primers with Primer-BLAST.
[c] Gender/Sex of donors: "M" – male, "F" – female; Tooth type: "PM" – premolar, "M" – molar; Cell density: given in cells/well if not otherwise mentioned.
[d] Frequencies labeled bold orange were converted to hertz (Hz) according to its definition using the information reported in the study (in brackets)
[e] Force type deduced from the description of the force apparatus given by the authors.
[f] Gene and protein expression: 1. conclusion of change (increase, decrease…) was given according to the defined criteria in Figure 2; 2. different markers to describe the amount of change; † Information derived from figures using Engauge Digitizer; *Folds calculated by measuring the graphs, without using the Engauge Digitizer; No makers: Information derived from figures by description in the articles

Reference	Gene/ Analyte[a]	Official gene symbol / abbreviation[b]	Cell (age/gender of donors, tooth type, isolation method, passages used, cell density)[a,c]	Force type (stat./ dyn.)[a]	Force duration and frequency[d]	Force magnitude[a]	Force apparatus[a]	Force type: equibiaxial or uniaxial[e]	Gene expression: Increase, decrease, no change (method w/ reference gene); Methods: qPCR, sqPCR, Northern blot[f]	Gene expression: When it reaches peak and peak's magnitude (fold change; times or ratio; unclear = ?)[f]	Protein expression: Increase, decrease, no change (method w/ reference); Methods: ELISA, WB, RIA, EMSA, IF[f]	Protein expression: When it reaches peak and peak's magnitude (times or ratio; unclear = ?)[f]
Chen et al. (2014)	TNFα	*TNF*	HPDLs (n.g./n.g., M, dig, P3-9, 10^4 cells/mL)	static	1d, 3d, 7d, 15d	-100 kPa (1Pa=1/100,000kg/cm^2, equal to a negative force of 101g/mm^2)	"a tension incubator" (TI, Model 3618P; LabLine Instrument, Thermolyne Co., IL, USA) + 24-well plate + vacuum	equibiaxial	increase (?, actin)	7d: 0.6 (?)* / 2.6 (ratio-calc)	n.g.	n.g.
Chen et al. (2015)	ARRAY	ARRAY	HPDLCs (18-25/n.g., M, exp, P3-5, 2×10^5)	dynamic	**0.1Hz** (6cyc/min) for 24h	12%	Flexcell FX-4000 strain unit + six-well flexible-bottomed uniflex-plates + vacuum	uniaxial	miRCURY LNA microRNA Array 7th generation (Exiqon A/S, Vedbaek, Denmark)		n.g.	n.g.
Chen et al. (2015)	COL1A1	*COL1A1*	HPDLCs (18-25/n.g., M, exp, P3-5, 2×10^5)	dynamic	**0.1Hz** (6cyc/min) for 24h	12%	Flexcell FX-4000 strain unit + six-well flexible-bottomed uniflex-plates + vacuum	uniaxial	increase (qPCR, β actin)	1.2 (FC)*	n.g.	n.g.
Chen et al. (2015)	COL3A1	*COL3A1*	HPDLCs (18-25/n.g., M, exp, P3-5, 2×10^5)	dynamic	**0.1Hz** (6cyc/min) for 24h	12%	Flexcell FX-4000 strain unit + six-well flexible-bottomed uniflex-plates + vacuum	uniaxial	increase (qPCR, β actin)	1.4 (FC)*	n.g.	n.g.
Chen et al. (2015)	COL5A1	*COL5A1*	HPDLCs (18-25/n.g., M, exp, P3-5, 2×10^5)	dynamic	**0.1Hz** (6cyc/min) for 24h	12%	Flexcell FX-4000 strain unit + six-well flexible-bottomed uniflex-plates + vacuum	uniaxial	increase (qPCR, β actin)	1.5 (FC)*	n.g.	n.g.
Chen et al. (2015)	miR-29a<br>miR-29b<br>miR-29c	*MIR29A*<br>*MIR29B1*<br>*MIR29C*	HPDLCs (18-25/n.g., M, exp, P3-5, 2×10^5)	dynamic	**0.1Hz** (6cyc/min) for 24h	12%	Flexcell FX-4000 strain unit + six-well flexible-bottomed uniflex-plates + vacuum	uniaxial	miR-29a: decrease (qPCR, U6snNA)<br>miR-29b: decrease (qPCR, U6snNA)<br>miR-29c: decrease (qPCR, U6snNA)	miR-29a: 0.6 (FC)*<br>miR-29b: 0.5 (FC)*<br>miR-29c: 0.6 (FC)*	n.g.	n.g.
Chen et al. (2015)	POSTN	*POSTN*	HPDLCs (18-25/n.g., M, exp, P3-5, 2×10^5)	dynamic	**0.1Hz** (6cyc/min) for 24h	12%	Flexcell FX-4000 strain unit + six-well flexible-bottomed uniflex-plates + vacuum	uniaxial	no change (qPCR, β actin)		n.g.	n.g.
Chiba and Mitani (2004)	ALP	*ALPP*	Human PDL cells (n.g./n.g., n.g., exp, P4-6, n.g.)	dynamic	**0.5Hz** (30cyc/min: 1s stretch and 1s relaxation) for 2d, 5d	15%	Flexercell strain unit + Bioflex plates + vacuum	equibiaxial	n.g.	n.g.	decrease (ALP activity, colorimetric assay)	5d: 0.8 (ratio)*
Cho et al. (2010)	BMP-2	*BMP2*	Human immortalized PDLCs (n.g./n.g., n.g., gene transfection, n.g., 3×10^5)	dynamic	**0.2Hz** (12cyc/min: stretch for 2.5s, 2.5s of relaxation) for 3h, 6h, 12h, 24h, 48h	12%	Flexercell FX-4000 Strain Unit + six-well, 35-mm flexible-bottomed Uniflex culture plates with a centrally located rectangular portion (15.25mm × 24.18mm) coated with type I collagen + vacuum	uniaxial	increase (sqPCR, GAPDH)	48h: 6.2 (ratio)†	n.g.	n.g.
Cho et al. (2010)	BMP-2	*BMP2*	Human immortalized PDLCs (n.g./n.g., n.g., gene transfection, n.g., 3×10^5)	dynamic	**0.2Hz** (12cyc/min: stretch for 2.5s, 2.5s of relaxation) for 48h	3%, 6%, 12%, 15%	Flexercell FX-4000 Strain Unit + six-well, 35-mm flexible-bottomed Uniflex culture plates with a centrally located rectangular portion (15.25mm × 24.18mm) coated with type I collagen + vacuum	uniaxial	increase (sqPCR, GAPDH)	15%: 2 (ratio)*	n.g.	n.g.
Cho et al. (2010)	BMP-7	*BMP7*	Human immortalized PDLCs (n.g./n.g., n.g., gene transfection, n.g., 3×10^5)	dynamic	**0.2Hz** (12cyc/min: stretch for 2.5s, 2.5s of relaxation) for 3h, 6h, 12h, 24h, 48h	12%	Flexercell FX-4000 Strain Unit + six-well, 35-mm flexible-bottomed Uniflex culture plates with a centrally located rectangular portion (15.25mm × 24.18mm) coated with type I collagen + vacuum	uniaxial	increase (sqPCR, GAPDH)	48h: 3.5 (ratio)†	n.g.	n.g.
Cho et al. (2010)	BMP-7	*BMP7*	Human immortalized PDLCs (n.g./n.g., n.g., gene transfection, n.g., 3×10^5)	dynamic	**0.2Hz** (12cyc/min: stretch for 2.5s, 2.5s of relaxation) for 48h	3%, 6%, 12%, 15%	Flexercell FX-4000 Strain Unit + six-well, 35-mm flexible-bottomed Uniflex culture plates with a centrally located rectangular portion (15.25mm × 24.18mm) coated with type I collagen + vacuum	uniaxial	increase (sqPCR, GAPDH)	15%: 7.1 (ratio)†	n.g.	n.g.
Cho et al. (2010)	HO-1	*HMOX1*	Human immortalized PDLCs (n.g./n.g., n.g., gene transfection, n.g., 3×10^5)	dynamic	**0.2Hz** (12cyc/min: stretch for 2.5s, 2.5s of relaxation) for 3h, 6h, 12h, 24h, 48h	12%	Flexercell FX-4000 Strain Unit + six-well, 35-mm flexible-bottomed Uniflex culture plates with a centrally located rectangular portion (15.25mm × 24.18mm) coated with type I collagen + vacuum	uniaxial	increase (sqPCR, GAPDH)	24h: 2.4 (ratio)†	increase (WB, β-actin)	48h: 2.3 (ratio)†
Cho et al. (2010)	HO-1	*HMOX1*	Human immortalized PDLCs (n.g./n.g., n.g., gene transfection, n.g., 3×10^5)	dynamic	**0.2Hz** (12cyc/min: stretch for 2.5s, 2.5s of relaxation) for 48h	3%, 6%, 12%, 15%	Flexercell FX-4000 Strain Unit + six-well, 35-mm flexible-bottomed Uniflex culture plates with a centrally located rectangular portion (15.25mm × 24.18mm) coated with type I collagen + vacuum	uniaxial	increase followed by decrease (sqPCR, GAPDH)	12%: 3.7 (ratio)†<br>15%: 0.6 (ratio)†	increase (WB, β-actin)	12%: 4.6 (ratio)†
Cho et al. (2010)	Noggin	*NOG*	Human immortalized PDLCs (n.g./n.g., n.g., gene transfection, n.g., 3×10^5)	dynamic	**0.2Hz** (12cyc/min: stretch for 2.5s, 2.5s of relaxation) for 48h	3%, 6%, 12%, 15%	Flexercell FX-4000 Strain Unit + six-well, 35-mm flexible-bottomed Uniflex culture plates with a centrally located rectangular portion (15.25mm × 24.18mm) coated with type I collagen + vacuum	uniaxial	decrease followed by plateau (sqPCR, GAPDH)	12h…24h: 0.3 (ratio)†	n.g.	n.g.

[a] Entry given as reported in the study.
[b] All official gene symbols come from the HUGO Gene Nomenclature Committee (HGNC; URL: https://www.genenames.org) after checking specificity of primers with Primer-BLAST.
[c] Gender/Sex of donors: "M" – male, "F" – female; Tooth type: "PM" – premolar, "M" – molar; Cell density: given in cells/well if not otherwise mentioned.
[d] Frequencies labeled bold orange were converted to hertz (Hz) according to its definition using the information reported in the study (in brackets)
[e] Force type deduced from the description of the force apparatus given by the authors.
[f] Gene and protein expression: 1. conclusion of change (increase, decrease…) was given according to the defined criteria in Figure 2; 2. different markers to describe the amount of change; † Information derived from figures using Engauge Digitizer; *Folds calculated by measuring the graphs, without using the Engauge Digitizer; No makers: Information derived from figures by description in the articles

Reference	Gene/ Analyte[a]	Official gene symbol / abbreviation[b]	Cell (age/gender of donors, tooth type, isolation method, passages used, cell density)[a,c]	Force type (stat./ dyn.)[a]	Force duration and frequency[d]	Force magnitude[a]	Force apparatus[a]	Force type: equibiaxial or uniaxial[e]	Gene expression: Increase, decrease, no change (method w/ reference gene); Methods: qPCR, sqPCR, Northern blot[f]	Gene expression: When it reaches peak and peak's magnitude (fold change; times or ratio; unclear = ?)[f]	Protein expression: Increase, decrease, no change (method w/ reference); Methods: ELISA, WB, RIA, EMSA, IF[f]	Protein expression: When it reaches peak and peak's magnitude (times or ratio; unclear = ?)[f]
Cho et al. (2010)	Noggin	*NOG*	Human immortalized PDLCs (n.g./n.g., n.g., gene transfection, n.g., 3×10^5)	dynamic	**0.2Hz** (12cyc/min: stretch for 2.5s, 2.5s of relaxation) for 3h, 6h, 12h, 24h, 48h	12%	Flexercell FX-4000 Strain Unit + six-well, 35-mm flexible-bottomed Uniflex culture plates with a centrally located rectangular portion (15.25mm × 24.18mm) coated with type I collagen + vacuum	uniaxial	decrease (sqPCR, GAPDH)	15%: 0.2 (ratio)†	n.g.	n.g.
Cho et al. (2010)	Runx2	*RUNX2*	Human immortalized PDLCs (n.g./n.g., n.g., gene transfection, n.g., 3×10^5)	dynamic	**0.2Hz** (12cyc/min: stretch for 2.5s, 2.5s of relaxation) for 0h, 3h, 6h, 12h, 24h, 48h	12%	Flexercell FX-4000 Strain Unit + six-well, 35-mm flexible-bottomed Uniflex culture plates with a centrally located rectangular portion (15.25mm × 24.18mm) coated with type I collagen + vacuum	uniaxial	decrease followed by plateau then increase (sqPCR, GAPDH)	3h…6h: 0.5 (ratio)†<br>48h: 3 (ratio)†	n.g.	n.g.
Cho et al. (2010)	Runx2	*RUNX2*	Human immortalized PDLCs (n.g./n.g., n.g., gene transfection, n.g., 3×10^5)	dynamic	**0.2Hz** (12cyc/min: stretch for 2.5s, 2.5s of relaxation) for 48h	3%, 6%, 12%, 15%	Flexercell FX-4000 Strain Unit + six-well, 35-mm flexible-bottomed Uniflex culture plates with a centrally located rectangular portion (15.25mm × 24.18mm) coated with type I collagen + vacuum	uniaxial	decrease followed by increase (sqPCR, GAPDH)	12%: 0.8 (ratio)†<br>15%: 1.5 (ratio)†	n.g.	n.g.
Deschner et al. (2012)	ARRAY	ARRAY	hPDL cells (n.g./n.g., n.g., exp, P4, 5×10^4)	dynamic	0.05Hz for 24h	3%	CESTRA cell strain device + BioFlex plates (silicone membranes) coated with collagen type I + stepping motor (Nokhbehsaim, 2010)	equibiaxial	PCR array (RT^2 PCR array)		n.g.	n.g.
Diercke et al. (2011)	Eph-B4	*EPHB4*	hPDL cells (12-20/n.g., PM, exp, P3-6, 80% confluence)	static	1h, 4h, 24h, 48h, 72h	2.5%	flexible bottomed dishes (Greiner Bio-One) coated with collagen type-I and fibronectin (Hasegawa,1985) Petriperm dish placed + template with a convex surface + weight	equibiaxial	temporary decrease (qPCR, GAPDH)	4h: 0.6 (FC)*<br>48h: 0.7 (FC)*	n.g.	n.g.
Diercke et al. (2011)	Ephrin-B2	*EFNB2*	hPDL cells (12-20/n.g., PM, exp, P3-6, 80% confluence)	static	1h, 4h, 24h, 48h, 72h	2.5%	flexible bottomed dishes (Greiner Bio-One) coated with collagen type-I and fibronectin (Hasegawa,1985) Petriperm dish placed + template with a convex surface + weight	equibiaxial	increase (qPCR, GAPDH)	24h: 1.9 (FC)*	n.g.	n.g.
Diercke et al. (2011)	FAK / p-FAK(Tyr^{576})	*PTK2*	hPDL cells (12-20/n.g., PM, exp, P3-6, 80% confluence)	static	5min, 15min, 30min, 60min, 4h, 24h, 48h, 72h	2.5%	flexible bottomed dishes (Greiner Bio-One) coated with collagen type-I and fibronectin (Hasegawa,1985) Petriperm dish placed + template with a convex surface + weight	equibiaxial	n.g.	n.g.	t-FAK: no change (WB, β-actin)<br>p-FAK: increase (WB,β-actin)	t-FAK: no quantitative information is given<br>p-FAK @ 72h: 1.6 (ratio)*
Doi et al. (2003)	RGD-CAP	*TGFBI*	hPDL cells (n.g./n.g., PM, exp, P4-5, 5×10^4)	dynamic	**0.5Hz** (30cyc/min) for 24h, 48h	7.2 kPa, 15.4 kPa	Flexercell strain unit + Flexercell plate dish + vacuum	equibiaxial	7.2 kPa: no change (qPCR, GAPDH)<br>15.4 kPa: increase (qPCR, GAPDH)	15.4 kPa @ 48h:1.7 (ratio)*	n.g.	n.g.
Fujihara et al. (2010)	ALP	*ALPP*	hPDL cells (n.g./n.g., PM, exp, P n.g., 1.5×10^4)	dynamic	0.5Hz (30cyc/min) for 48h	10% (110%)	Scholertec NS-350 (Scholertec) + $10cm^2$ silicon membrane chambers (Scholertec, Osaka, Japan)	uniaxial	increase (qPCR, HPRT)	0.12 (rel)* / 3 (ratio-calc)	n.g.	n.g.
Fujihara et al. (2010)	ARRAY	ARRAY	hPDL cells (n.g./n.g., PM, exp, P n.g., 1.5×10^4)	dynamic	0.5Hz (30cyc/min) for 48h	10% (110%)	Scholertec NS-350 (Scholertec) + $10cm^2$ silicon membrane chambers (Scholertec, Osaka, Japan)	uniaxial	Oligo-DNA Chip Analysis: AceGene Human Oligo Chip 30K (Hitachi Software Eng. Co., Ibaraki, Japan)			
Fujihara et al. (2010)	c-FOS	*FOS*	hPDL cells (n.g./n.g., PM, exp, P n.g., 1.5×10^4)	dynamic	0.5Hz (30cyc/min) for 48h	10% (110%)	Scholertec NS-350 (Scholertec) + $10cm^2$ silicon membrane chambers (Scholertec, Osaka, Japan)	uniaxial	increase (qPCR, HPRT)	2.6 (rel)* / 2.9 (ratio-calc)	n.g.	n.g.
Fujihara et al. (2010)	CREB/p-CREB	*CREB1*	hPDL cells (n.g./n.g., PM, exp, P n.g., 1.5×10^4)	dynamic	0.5Hz (30cyc/min) for 24h, 48h	10% (110%)	Scholertec NS-350 (Scholertec) + $10cm^2$ silicon membrane chambers (Scholertec, Osaka, Japan)	uniaxial	n.g.	n.g.	p-CREB/CREB increase (WB, CREB)	48h: 1.5 (p-CREB/CREB)* / 1.5 (ratio stretch/control)*
Fujihara et al. (2010)	Glutamate	Glutamate	hPDL cells (n.g./n.g., PM, exp, P n.g., 1.5×10^4)	dynamic	0.5Hz (30cyc/min) for 12h, 24h, 48h	10% (110%)	Scholertec NS-350 (Scholertec) + $10cm^2$ silicon membrane chambers (Scholertec, Osaka, Japan)	uniaxial	n.a.	n.a.	increase (ELISA)	48h: 114.6 (nmol/µg)† / 3.5 (ratio-calc)
Fujihara et al. (2010)	GRIA3	*GRIA3*	hPDL cells (n.g./n.g., PM, exp, P n.g., 1.5×10^4)	dynamic	0.5Hz (30cyc/min) for 24h	10% (110%)	Scholertec NS-350 (Scholertec) + $10cm^2$ silicon membrane chambers (Scholertec, Osaka, Japan)	uniaxial	increase (sqPCR, HPRT)	0.9 (rel)† / 1.4 (ratio-calc)	n.g.	n.g.
Fujihara et al. (2010)	GRIN1	*GRIN1*	hPDL cells (n.g./n.g., PM, exp, P n.g., 1.5×10^4)	dynamic	0.5Hz (30cyc/min) for 24h	10% (110%)	Scholertec NS-350 (Scholertec) + $10cm^2$ silicon membrane chambers (Scholertec, Osaka, Japan)	uniaxial	increase (sqPCR, HPRT)	0.6 (rel)†/ control n.g.	n.g.	n.g.
Fujihara et al. (2010)	GRIN2C	*GRIN2C*	hPDL cells (n.g./n.g., PM, exp, P n.g., 1.5×10^4)	dynamic	0.5Hz (30cyc/min) for 24h	10% (110%)	Scholertec NS-350 (Scholertec) + $10cm^2$ silicon membrane chambers (Scholertec, Osaka, Japan)	uniaxial	increase (sqPCR, HPRT)	2.1 (rel)† / 2.2 (ratio-calc)	n.g.	n.g.
Fujihara et al. (2010)	GRIN2D	*GRIN2D*	hPDL cells (n.g./n.g., PM, exp, P n.g., 1.5×10^4)	dynamic	0.5Hz (30cyc/min) for 24h	10% (110%)	Scholertec NS-350 (Scholertec) + $10cm^2$ silicon membrane chambers (Scholertec, Osaka, Japan)	uniaxial	increase (sqPCR, HPRT)	7.6 (rel)† / 1.2 (ratio-calc)	n.g.	n.g.
Fujihara et al. (2010)	GRIN3A	*GRIN3A*	hPDL cells (n.g./n.g., PM, exp, P n.g., 1.5×10^4)	dynamic	0.5Hz (30cyc/min) for 24h, 48h	10% (110%)	Scholertec NS-350 (Scholertec) + $10cm^2$ silicon membrane chambers (Scholertec, Osaka, Japan)	uniaxial	increase (qPCR, HPRT)	48h: 3 (ratio)*	n.g.	n.g.
Fujihara et al. (2010)	GRIN3A	*GRIN3A*	hPDL cells (n.g./n.g., PM, exp, P n.g., 1.5×10^4)	dynamic	0.5Hz (30cyc/min) for 48h	10% (110%)	Scholertec NS-350 (Scholertec) + $10cm^2$ silicon membrane chambers (Scholertec, Osaka, Japan)	uniaxial	increase (qPCR, HPRT)	0.3 (rel)* / 3.8 (ratio-calc)	n.g.	n.g.

[a] Entry given as reported in the study.
[b] All official gene symbols come from the HUGO Gene Nomenclature Committee (HGNC; URL: https://www.genenames.org) after checking specificity of primers with Primer-BLAST.
[c] Gender/Sex of donors: "M" – male, "F" – female; Tooth type: "PM" – premolar, "M" – molar; Cell density: given in cells/well if not otherwise mentioned.
[d] Frequencies labeled bold orange were converted to hertz (Hz) according to its definition using the information reported in the study (in brackets)
[e] Force type deduced from the description of the force apparatus given by the authors.
[f] Gene and protein expression: 1. conclusion of change (increase, decrease…) was given according to the defined criteria in Figure 2; 2. different markers to describe the amount of change; † Information derived from figures using Engauge Digitizer; *Folds calculated by measuring the graphs, without using the Engauge Digitizer; No makers: Information derived from figures by description in the articles

Reference	Gene/ Analyte[a]	Official gene symbol / abbreviation[b]	Cell (age/gender of donors, tooth type, isolation method, passages used, cell density)[a,c]	Force type (stat./ dyn.)[a]	Force duration and frequency[d]	Force magnitude[a]	Force apparatus[a]	Force type: equibiaxial or uniaxial[e]	Gene expression: Increase, decrease, no change (method w/ reference gene); Methods: qPCR, sqPCR, Northern blot[f]	Gene expression: When it reaches peak and peak's magnitude (fold change; times or ratio; unclear = ?)[f]	Protein expression: Increase, decrease, no change (method w/ reference); Methods: ELISA, WB, RIA, EMSA, IF[f]	Protein expression: When it reaches peak and peak's magnitude (times or ratio; unclear = ?)[f]
Fujihara et al. (2010)	GRIN3B	*GRIN3B*	hPDL cells (n.g./n.g., PM, exp, P n.g., 1.5×10^4)	dynamic	0.5Hz (30cyc/min) for 24h	10% (110%)	Scholertec NS-350 (Scholertec) + $10cm^2$ silicon membrane chambers (Scholertec, Osaka, Japan)	uniaxial	increase (sqPCR, HPRT)	0.6 (rel)† / control n.g.	n.g.	n.g.
Fujihara et al. (2010)	HOMER1	*HOMER1*	hPDL cells (n.g./n.g., PM, exp, P n.g., 1.5×10^4)	dynamic	0.5Hz (30cyc/min) for 24h, 48h	10% (110%)	Scholertec NS-350 (Scholertec) + $10cm^2$ silicon membrane chambers (Scholertec, Osaka, Japan)	uniaxial	increase (qPCR, HPRT)	48h: 3.3 (ratio)*	n.g.	n.g.
Fujihara et al. (2010)	HOMER1	*HOMER1*	hPDL cells (n.g./n.g., PM, exp, P n.g., 1.5×10^4)	dynamic	0.5Hz (30cyc/min) for 48h	10% (110%)	Scholertec NS-350 (Scholertec) + $10cm^2$ silicon membrane chambers (Scholertec, Osaka, Japan)	uniaxial	increase (qPCR, HPRT)	0.8 (rel)* / 2 (ratio-calc)	n.g.	n.g.
Fujihara et al. (2010)	mGluR2	*GRM2*	hPDL cells (n.g./n.g., PM, exp, P n.g., 1.5×10^4)	dynamic	0.5Hz (30cyc/min) for 24h	10% (110%)	Scholertec NS-350 (Scholertec) + $10cm^2$ silicon membrane chambers (Scholertec, Osaka, Japan)	uniaxial	increase (sqPCR, HPRT)	2.3 (rel)† / control n.g.	n.g.	n.g.
Fujihara et al. (2010)	mGluR3	*GRM3*	hPDL cells (n.g./n.g., PM, exp, P n.g., 1.5×10^4)	dynamic	0.5Hz (30cyc/min) for 24h	10% (110%)	Scholertec NS-350 (Scholertec) + $10cm^2$ silicon membrane chambers (Scholertec, Osaka, Japan)	uniaxial	increase (sqPCR, HPRT)	4.4 (rel)† / control n.g.	n.g.	n.g.
Fujihara et al. (2010)	mGluR4	*GRM4*	hPDL cells (n.g./n.g., PM, exp, P n.g., 1.5×10^4)	dynamic	0.5Hz (30cyc/min) for 24h	10% (110%)	Scholertec NS-350 (Scholertec) + $10cm^2$ silicon membrane chambers (Scholertec, Osaka, Japan)	uniaxial	increase (sqPCR, HPRT)	4.3 (rel)†/ 2.5 (ratio-calc)	n.g.	n.g.
Fujihara et al. (2010)	mGluR5	*GRM5*	hPDL cells (n.g./n.g., PM, exp, P n.g., 1.5×10^4)	dynamic	0.5Hz (30cyc/min) for 24h	10% (110%)	Scholertec NS-350 (Scholertec) + $10cm^2$ silicon membrane chambers (Scholertec, Osaka, Japan)	uniaxial	increase (sqPCR, HPRT)	1.3 (rel)† / control n.g.	n.g.	n.g.
Fujihara et al. (2010)	mGluR6	*GRM6*	hPDL cells (n.g./n.g., PM, exp, P n.g., 1.5×10^4)	dynamic	0.5Hz (30cyc/min) for 24h	10% (110%)	Scholertec NS-350 (Scholertec) + $10cm^2$ silicon membrane chambers (Scholertec, Osaka, Japan)	uniaxial	increase (sqPCR, HPRT)	2.8 (rel)† / control n.g.	n.g.	n.g.
Fujihara et al. (2010)	RUNX2	*RUNX2*	hPDL cells (n.g./n.g., PM, exp, P n.g., 1.5×10^4)	dynamic	0.5Hz (30cyc/min) for 48h	10% (110%)	Scholertec NS-350 (Scholertec) + $10cm^2$ silicon membrane chambers (Scholertec, Osaka, Japan)	uniaxial	increase (qPCR, HPRT)	0.7 (rel)* / 1.6 (ratio-calc)	n.g.	n.g.
Fujihara et al. (2010)	VGLUT1	*SLC17A7*	hPDL cells (n.g./n.g., PM, exp, P n.g., 1.5×10^4)	dynamic	0.5Hz (30cyc/min) for 24h	10% (110%)	Scholertec NS-350 (Scholertec) + $10cm^2$ silicon membrane chambers (Scholertec, Osaka, Japan)	uniaxial	increase (sqPCR, HPRT)	2.1 (rel)† / control n.g.	n.g.	n.g.
Goto et al. (2011)	ARRAY	ARRAY	hPDLCs (n.g./n.g., M., dig, P4-8, 5 x 10^5 cells/cm^2)	dynamic	**0.017Hz (1/60Hz)** (conditions: 60s/returns; resting time: 29s) for 48h	5% (105%)	STB-140 (Strex Co) + silicon chamber	uniaxial	GeneChip Human Genome U133 plus 2.0 arrays (Agilent Technologies, Santa Clara, CA, USA)	too many	n.a.	n.a.
Goto et al. (2011)	CCL2	*CCL2*	hPDLCs (n.g./n.g., M., dig, P4-8, 5 x 10^5 cells/cm^2)	dynamic	**0.017Hz (1/60Hz)** (conditions: 60s/returns; resting time: 29s) sqPCR for 1d, 2d, 3d, 5d, 7d; qPCR for 1d,3d,5d, 7d; ELISA for 1d, 2d, 3d, 5d; WB for 1d, 2d, 3d, 5d, 7d	5% (105%)	STB-140 (Strex Co) + silicon chamber	uniaxial	increase (sqPCR, β-actin)<br>decrease followed by increase (qPCR, β-actin)	sqPCR: no quantitative information is given<br>qPCR lowest @ 1d: 0.3 (ratio)†<br>qPCR highest @ 7d: 27.3 (ratio)†	increase followed by plateau (ELISA)	3d...5d: 3.3 (ng/ml)* / ratio can not be calculated
Goto et al. (2011)	CCL3	*CCL3*	hPDLCs (n.g./n.g., M., dig, P4-8, 5 x 10^5 cells/cm^2)	dynamic	**0.017Hz (1/60Hz)** (conditions: 60s/returns; resting time: 29s) sqPCR for 1d, 2d, 3d, 5d, 7d; qPCR for 1d, 3d, 5d, 7d; WB for 1d, 2d, 3d, 5d, 7d	5% (105%)	STB-140 (Strex Co) + silicon chamber	uniaxial	temporary increase (sqPCR, β-actin)<br>increase (qPCR, β-actin)	sqPCR: no quantitative information is given<br>qPCR: 7d: 16.2 (ratio)†	increase (WB, β-actin)	no quantitative information is given
Goto et al. (2011)	CCL5	*CCL5*	hPDLCs (n.g./n.g., M., dig, P4-8, 5 x 10^5 cells/cm^2)	dynamic	**0.017Hz (1/60Hz)** (conditions: 60s/returns; resting time: 29s) sqPCR for 1d, 2d, 3d, 5d, 7d; qPCR for 1d, 3d, 5d, 7d; WB for 1d, 2d, 3d, 5d, 7d	5% (105%)	STB-140 (Strex Co) + silicon chamber	uniaxial	temporary increase (sqPCR, β-actin)<br>decrease followed by increase (qPCR, β-actin)	sqPCR: no quantitative information is given<br>qPCR lowest @ 3d: 0.8 (ratio)†<br>qPCR highest @ 7d: 7.0 (ratio)†	increase (WB, β-actin)	no quantitative information is given

[a] Entry given as reported in the study.
[b] All official gene symbols come from the HUGO Gene Nomenclature Committee (HGNC; URL: https://www.genenames.org) after checking specificity of primers with Primer-BLAST.
[c] Gender/Sex of donors: "M" – male, "F" – female; Tooth type: "PM" – premolar, "M" – molar; Cell density: given in cells/well if not otherwise mentioned.
[d] Frequencies labeled bold orange were converted to hertz (Hz) according to its definition using the information reported in the study (in brackets)
[e] Force type deduced from the description of the force apparatus given by the authors.
[f] Gene and protein expression: 1. conclusion of change (increase, decrease…) was given according to the defined criteria in Figure 2; 2. different markers to describe the amount of change; † Information derived from figures using Engauge Digitizer; *Folds calculated by measuring the graphs, without using the Engauge Digitizer; No makers: Information derived from figures by description in the articles

Reference	Gene/ Analyte[a]	Official gene symbol / abbreviation[b]	Cell (age/gender of donors, tooth type, isolation method, passages used, cell density)[a,c]	Force type (stat./ dyn.)[a]	Force duration and frequency[d]	Force magnitude[a]	Force apparatus[a]	Force type: equibiaxial or uniaxial[e]	Gene expression: Increase, decrease, no change (method w/ reference gene); Methods: qPCR, sqPCR, Northern blot[f]	Gene expression: When it reaches peak and peak's magnitude (fold change; times or ratio; unclear = ?)[f]	Protein expression: Increase, decrease, no change (method w/ reference); Methods: ELISA, WB, RIA, EMSA, IF[f]	Protein expression: When it reaches peak and peak's magnitude (times or ratio; unclear = ?)[f]
Hao et al. (2009)	Caspase-3	*CASP3*	hPDL cells (13/F, 15/M, PM, exp, P 3-5, n.g.)	dynamic	**0.1Hz** (6cyc/min: Spherical cap ascended from the initial point to the highest point for 1s, kept at the highest point for 4s, descended to the initial point for 1s, and then kept at the initial point for 4s) for 6h, 12h, 24h, 48h	1%, 10%, 20%	"Cell Strain Unit (CSU)" + elastic silicon rubber membrane + spherical cap (step motor)	equibiaxial	n.g.	n.g.	1%: increase (Caspase-3 colorimetric assay)<br>10%: increase (Caspase-3 colorimetric assay)<br>20%: increase (Caspase-3 colorimetric assay)	1% @ 24h: 2.6 (ratio)*<br>10% @ 24h: 3 (ratio)*<br>20% @ 24h: 3.8 (ratio)*
He et al. (2004)	COL1A1	*COL1A1*	hPDL cells (n.g./n.g., M, dig, P3-12, 10^5 cells/cm^2)	dynamic	**0.5Hz** (30cyc/min) for 24h	10%	plastic culture cylinder + elastic silicone membrane + movable plate	equibiaxial	increase (qPCR, GAPDH)	5.695 (rel) / 4.4 (ratio-calc)	increase (ELISA)	4.1 (pg/cell)* / 3.5 (ratio-calc)
He et al. (2004)	Fibronectin	FN1	hPDL cells (n.g./n.g., M, dig, P3-12, 10^5 cells/cm^2)	dynamic	**0.5Hz** (30cyc/min) for 24h	10%	plastic culture cylinder + elastic silicone membrane + movable plate	equibiaxial	n.g.	n.g.	increase (ELISA)	10.5 (pg/cell)* / 1.4 (ratio-calc)
He et al. (2004)	MMP2	MMP2	hPDL cells (n.g./n.g., M, dig, P3-12, 10^5 cells/cm^2)	dynamic	**0.5Hz** (30cyc/min) for 24h	10%	plastic culture cylinder + elastic silicone membrane + movable plate	equibiaxial	increase (Northern, GAPDH)	0.241 (rel) / 1.4 (ratio-calc)	decrease (zymography)	7.6 (rel) / 0.9 (ratio-calc)
He et al. (2004)	TIMP2	TIMP2	hPDL cells (n.g./n.g., M, dig, P3-12, 10^5 cells/cm^2)	dynamic	**0.5Hz** (30cyc/min) for 24h	10%	plastic culture cylinder + elastic silicone membrane + movable plate	equibiaxial	increase (Northern, GAPDH)	0.118 (rel) / 1.6 (ratio-calc)	n.g.	n.g.
He et al. (2019)	RhoA	*RHOA*	hPDLCs ($) (n.g./n.g., n.g., n.g., P n.g., 80-90 confluence)	dynamic	0.5Hz for 3h	n. g.	a uniaxial four-point bending system (developed at Sichuan University, patents CN2534576 and CN1425905)	uniaxial	increase (qPCR, β-actin)	40.2 (rel)† / 28.9 (ratio-calc)	n.g.	n.g.
He et al. (2019)	ROCK	*ROCK1*	hPDLCs ($) (n.g./n.g., n.g., n.g., P n.g., 80-90 confluence)	dynamic	0.5Hz for 3h	n. g.	a uniaxial four-point bending system (developed at Sichuan University, patents CN2534576 and CN1425905)	uniaxial	increase (qPCR, β-actin)	10.7 (rel)† / 10.8 (ratio-calc)	n.g.	n.g.
He et al. (2019)	RUNX2	*RUNX2*	hPDLCs ($) (n.g./n.g., n.g., n.g., P n.g., 80-90 confluence)	dynamic	0.5Hz for 3h	n. g.	a uniaxial four-point bending system (developed at Sichuan University, patents CN2534576 and CN1425905)	uniaxial	increase (qPCR, β-actin)	10.1 (rel)† / 10.6 (ratio-calc)	n.g.	n.g.
He et al. (2019)	TGF-β1	*TGFB1*	hPDLCs ($) (n.g./n.g., n.g., n.g., P n.g., 80-90 confluence)	dynamic	0.5Hz for 3h	n. g.	a uniaxial four-point bending system (developed at Sichuan University, patents CN2534576 and CN1425905)	uniaxial	increase (qPCR, β-actin)	12.7 (rel)† / 12.8 (ratio-calc)	n.g.	n.g.
He et al. (2019)	YAP	*YAP1*	hPDLCs ($) (n.g./n.g., n.g., n.g., P n.g., 80-90 confluence)	dynamic	0.5Hz for 1h, 3h, 6h, 12h, 18h	**0.2%** (2000μstrain)	a uniaxial four-point bending system (developed at Sichuan University, patents CN2534576 and CN1425905)	uniaxial	temporary increase (qPCR, β-actin)	3h: 10.3 (FC)†	n.g.	n.g.
He et al. (2019)	YAP	*YAP1*	hPDLCs ($) (n.g./n.g., n.g., n.g., P n.g., 80-90 confluence)	dynamic	0.5Hz for 3h	**0.4%** (4000μstrain)	a uniaxial four-point bending system (developed at Sichuan University, patents CN2534576 and CN1425905)	uniaxial	increase (qPCR, β-actin)	16.3 (FC)†	n.g.	n.g.
He et al. (2019)	YAP	*YAP1*	hPDLCs ($) (n.g./n.g., n.g., n.g., P n.g., 80-90 confluence)	dynamic	0.5Hz for 3h	n. g.	a uniaxial four-point bending system (developed at Sichuan University, patents CN2534576 and CN1425905)	uniaxial	increase (qPCR, β-actin)	9.1 (rel)† / 8.8 (ratio-calc)	whole protein: increase (GAPDH)<br>nucleo-protein: increase (GAPDH)	no quantitative information is given
He et al. (2019)	α-SMA	*ACTA2*	hPDLCs ($) (n.g./n.g., n.g., n.g., P n.g., 80-90 confluence)	dynamic	0.5Hz for 1h, 3h, 6h, 12h, 18h	**0.2%** (2000μstrain)	a uniaxial four-point bending system (developed at Sichuan University, patents CN2534576 and CN1425905)	uniaxial	temporary increase (qPCR, β-actin)	6h: 13.4 (FC)†	n.g.	n.g.
He et al. (2019)	α-SMA	*ACTA2*	hPDLCs ($) (n.g./n.g., n.g., n.g., P n.g., 80-90 confluence)	dynamic	0.5Hz for 3h	**0.4%** (4000μstrain)	a uniaxial four-point bending system (developed at Sichuan University, patents CN2534576 and CN1425905)	uniaxial	increase (qPCR, β-actin)	12.6 (FC)†	n.g.	n.g.
He et al. (2019)	α-SMA	*ACTA2*	hPDLCs ($) (n.g./n.g., n.g., n.g., P n.g., 80-90 confluence)	dynamic	0.5Hz for 3h	n. g.	a uniaxial four-point bending system (developed at Sichuan University, patents CN2534576 and CN1425905)	uniaxial	increase (qPCR, β-actin)	21.5 (rel)† / 21.8 (ratio-calc)	whole protein: increase (GAPDH)	no quantitative information is given
Howard et al. (1998)	Fibronectin	FN1	hPDL cells (n.g./n.g., PM, dig, P3-8, Confluent)	dynamic	**0.5Hz** (30times/min) for 24h	5%, 10%	circularly clamped compliant membrane + spherical cap + vacuum	equibiaxial	n.g.	n.g.	increase (ELISA)	5%: 11.6 (pg/cell)* / 2.9 (ratio-calc)<br>10%: 3.7 (pg/cell)* / 5.1 (ratio-calc)
Howard et al. (1998)	Tropoelastin	*ELN*	hPDL cells (n.g./n.g., PM, dig, P3-8, Confluent)	dynamic	**0.5Hz** (30times/min) for 24h	5%, 10%	circularly clamped compliant membrane + spherical cap + vacuum	equibiaxial	n.g.	n.g.	5% (ELISA): decrease<br>10% (ELISA): decrease	5%: 0.02 (pg/cell)* / 0.4 (ratio-calc)<br>10%: 0.006 (pg/cell)* / 0.4 (ratio-calc)
Howard et al. (1998)	Type I Collagen	*COL1A1; COL1A2*	hPDL cells (n.g./n.g., PM, dig, P3-8, Confluent)	dynamic	**0.5Hz** (30times/min) for 24h	5%, 10%	circularly clamped compliant membrane + spherical cap + vacuum	equibiaxial	n.g.	n.g.	5% (ELISA): increase<br>10% (ELISA): no change	5%: 0.3 (pg/cell)* / 1.7 (ratio-calc)
Huelter-Hassler et al. (2017)	ERK1/2 / p-ERK1/2($T^{202,204}$)	MAPK3; MAPK1	hPDL cells (14/M, M, dig, P5-8, 1×10^4 cells/cm^2,)	static	equiaxial strain for 15min, 1h, 6h, 24h	2.5%	Flexercell Strain Unit FX5000-T + silicone bottomed six-well plates coated with collagen type I (Flexcell Int. Corp.) + vacuum	equibiaxial	n.g.	n.g.	ERK1/2 (WB): decrease followed by increase<br>p-ERK1/2 (WB): decrease	ERK1/2: lowest @15min: 0.5 (ratio)*<br>ERK1/2: highest @ 24h: 1.5 (ratio)*<br>p-ERK1/2: 1h: 0.4 (ratio)*

[a] Entry given as reported in the study.
[b] All official gene symbols come from the HUGO Gene Nomenclature Committee (HGNC; URL: https://www.genenames.org) after checking specificity of primers with Primer-BLAST.
[c] Gender/Sex of donors: "M" – male, "F" – female; Tooth type: "PM" – premolar, "M" – molar; Cell density: given in cells/well if not otherwise mentioned.
[d] Frequencies labeled bold orange were converted to hertz (Hz) according to its definition using the information reported in the study (in brackets)
[e] Force type deduced from the description of the force apparatus given by the authors.
[f] Gene and protein expression: 1. conclusion of change (increase, decrease…) was given according to the defined criteria in Figure 2; 2. different markers to describe the amount of change; † Information derived from figures using Engauge Digitizer; *Folds calculated by measuring the graphs, without using the Engauge Digitizer; No makers: Information derived from figures by description in the articles

Reference	Gene/ Analyte[a]	Official gene symbol / abbreviation[b]	Cell (age/gender of donors, tooth type, isolation method, passages used, cell density)[a,c]	Force type (stat./ dyn.)[a]	Force duration and frequency[d]	Force magnitude[a]	Force apparatus[a]	Force type: equibiaxial or uniaxial[e]	Gene expression: Increase, decrease, no change (method w/ reference gene); Methods: qPCR, sqPCR, Northern blot[f]	Gene expression: When it reaches peak and peak's magnitude (fold change; times or ratio; unclear = ?)[f]	Protein expression: Increase, decrease, no change (method w/ reference); Methods: ELISA, WB, RIA, EMSA, IF[f]	Protein expression: When it reaches peak and peak's magnitude (times or ratio; unclear = ?)[f]
Huelter-Hassler et al. (2017)	KI-67	*MKI67*	hPDL cells (14/M, M, dig, P5-8, 1×10^4 cells/cm^2,)	static	equiaxial strain for 15min, 1h, 6h, 24h	2.5%	Flexercell Strain Unit FX5000-T + silicone bottomed six-well plates coated with collagen type I (Flexcell Int. Corp.) + vacuum	equibiaxial	n.g.	n.g.	Increase followed by decrease (WB)	highest @ 15min: 1.8 (ratio)*<br>lowest @ 24h: 0.3 (ratio)*
Huelter-Hassler et al. (2017)	YAP	*YAP1*	hPDL cells (14/M, M, dig, P5-8, 1×10^4 cells/cm^2,)	static	equiaxial strain for 15min, 1h, 6h, 24h	2.5%	Flexercell Strain Unit FX5000-T + silicone bottomed six-well plates coated with collagen type I (Flexcell Int. Corp.) + vacuum	equibiaxial	n.g.	n.g.	cytoplasmic YAP (WB): no change<br>nuclear YAP (WB): increase followed by decrease	nuclear YAP: highest @ 1h: 1.3 (ratio)*<br>nucelar YAP: lowest @ 24h: 0.6 (ratio)*
Hülter-Hassler et al. (2017)	ERK1/2 / p-ERK1/2($T^{202,204}$)	MAPK3; MAPK1	hPDL cells (14/M, M, dig, P5-8, 1×10^4 cells/cm^2,)	static	equiaxial mechanical strain for 15min, 1h, 6h, 24h	2.5%	Flexercell Strain Unit FX5000-T + silicone bottomed six-well plates coated with collagen type I (Flexcell Int. Corp.) + vacuum	equibiaxial	n.g.	n.g.	ERK1/2 (WB): decrease followed by increase<br>p-ERK1/2 (WB): decrease	ERK1/2: lowest @ 15min: 0.6 (ratio)*<br>ERK1/2: highest @ 24h: 1.5 (ratio)*<br>p-ERK1/2 (WB): 1h: 0.4 (ratio)*
Hülter-Hassler et al. (2017)	FAK / p-FAK(Y^{397})	*PTK2*	hPDL cells (14/M, M, dig, P5-8, 1×10^4 cells/cm^2,)	static	equiaxial mechanical strain for 15min, 1h, 6h, 24h	2.5%	Flexercell Strain Unit FX5000-T + silicone bottomed six-well plates coated with collagen type I (Flexcell Int. Corp.) + vacuum	equibiaxial	n.g.	n.g.	FAK increase followed by decrease (WB)<br>p-FAK increase followed by decrease (WB):	FAK: highest @ 1h: 1.7 (ratio)*<br>FAK: lowest @ 24h: 0.8 (ratio)*<br>p-FAK: highest @ 1h: 1.4 (ratio)*<br>p-FAK: lowest @ 24h: 0.7 (ratio)*
Hülter-Hassler et al. (2017)	KI-67	*MKI67*	hPDL cells (14/M, M, dig, P5-8, 1×10^4 cells/cm^2,)	static	equiaxial mechanical strain for 15min, 1h, 6h, 24h	2.5%	Flexercell Strain Unit FX5000-T + silicone bottomed six-well plates coated with collagen type I (Flexcell Int. Corp.) + vacuum	equibiaxial	n.g.	n.g.	increase followed by decrease (WB)	highest @ 15min: 1.8 (ratio)*<br>lowest @ 24h: 0.3 (ratio)*
Hülter-Hassler et al. (2017)	YAP	*YAP1*	hPDL cells (14/M, M, dig, P5-8, 1×10^4 cells/cm^2,)	static	equiaxial mechanical strain for 15min, 1h, 6h, 24h	2.5%	Flexercell Strain Unit FX5000-T + silicone bottomed six-well plates coated with collagen type I (Flexcell Int. Corp.) + vacuum	equibiaxial	n.g.	n.g.	cytoplasmic extract (WB): no change<br>nuclear extract (WB): increase followed by decrease	YAP (nuclear extract): highest @ 1h: 1.3 ratio)*<br>YAP (nuclear extract): lowest @ 24h: 0.6 (ratio)*
Jacobs et al. (2013)	ALP	*ALPP*	hPDL cells ($) (n.g./n.g., n.g, method n.g., P4-6, subconfluency)	static	12h	1% (0.7cN/mm^2), 5% (3cN/mm^2), 10% (5.2cN/mm^2)	Flexercell Strain Unit FX 3000 + BioFlex® Plates coated with pronectin + vacuum	equibiaxial	increase (qPCR, actin + GAPDH)	5%: 2.7 (FC)*	n.g.	n.g.
Jacobs et al. (2013)	Collagen type-I (COL-1)	*COL1A1*	hPDL cells ($) (n.g./n.g., n.g, method n.g., P4-6, subconfluency)	static	12h	1% (0.7cN/mm^2), 5% (3cN/mm^2), 10% (5.2cN/mm^2)	Flexercell Strain Unit FX 3000 + BioFlex® Plates coated with pronectin + vacuum	equibiaxial	decrease followed by increase then decrease (qPCR, actin + GAPDH)	1%: 0.8 (FC)†<br>5%: 1.1 (FC)†<br>10%: 0.7 (FC)†	n.g.	n.g.
Jacobs et al. (2013)	Cyclin D1	*CCND1*	hPDL cells ($) (n.g./n.g., n.g, method n.g., P4-6, subconfluency)	static	12h	1% (0.7cN/mm^2), 5% (3cN/mm^2), 10% (5.2cN/mm^2)	Flexercell Strain Unit FX 3000 + BioFlex® Plates coated with pronectin + vacuum	equibiaxial	increase (qPCR, actin + GAPDH)	10%: 8.3 (FC)*	n.g.	n.g.
Jacobs et al. (2013)	OCN	*BGLAP*	hPDL cells ($) (n.g./n.g., n.g, method n.g., P4-6, subconfluency)	static	12h	1% (0.7cN/mm^2), 5% (3cN/mm^2), 10% (5.2cN/mm^2)	Flexercell Strain Unit FX 3000 + BioFlex® Plates coated with pronectin + vacuum	equibiaxial	increase followed by decrease (qPCR, actin + GAPDH)	highest @ 1%: 1.3 (FC)*<br>lowest @ 5%: 0.6 (FC)*	n.g.	n.g.
Jacobs et al. (2013)	OPG	*TNFRSF11B*	hPDL cells ($) (n.g./n.g., n.g, method n.g., P4-6, subconfluency)	static	12h	1% (0.7cN/mm^2), 5% (3cN/mm^2), 10% (5.2cN/mm^2)	Flexercell Strain Unit FX 3000 + BioFlex® Plates coated with pronectin + vacuum	equibiaxial	increase (qPCR, actin + GAPDH)	5%: 2.7 (FC)*	increase (ELISA)	10%: 44.6 (ng/10^5 cells)* / control no OPG detectable
Jacobs et al. (2013)	RANKL	*TNFSF11*	hPDL cells ($) (n.g./n.g., n.g, method n.g., P4-6, subconfluency)	static	12h	1% (0.7cN/mm^2), 5% (3cN/mm^2), 10% (5.2cN/mm^2)	Flexercell Strain Unit FX 3000 + BioFlex® Plates coated with pronectin + vacuum	equibiaxial	increase followed by decrease (qPCR, actin + GAPDH)	highest @1%: 1.6 (FC)*<br>lowest @ 5%: 0.7 (FC)*	n.g.	n.g.
Jacobs et al. (2014)	COX-2	*PTGS2*	hPDL cells ($) (n.g./n.g., n.g, method n.g., P4-6, subconfluency)	static	12h	1% (0.7cN/mm^2), 5% (3cN/mm^2), 10% (5.2cN/mm^2)	Flexercell Strain Unit FX 3000 + Bioflex® Plates + vacuum	equibiaxial	increase (qPCR, actin + GAPDH)	10%: 31.4 (FC)	n.g.	n.g.
Jacobs et al. (2014)	IL-6	*IL6*	hPDL cells ($) (n.g./n.g., n.g, method n.g., P4-6, subconfluency)	static	12h	1% (0.7cN/mm2), 5% (3 cN/mm^2), 10% (5.2 cN/mm^2)	Flexercell Strain Unit FX 3000 + Bioflex® Plates + vacuum	equibiaxial	decrease followed by increase (qRT-PCR, actin + GAPDH)	lowest @ 1%: 0.5 (FC)*<br>highest @ 10%: 1.6 (FC)*	decrease followed by increase (ELISA)	lowest @ 1%: 5.6 (pg/ml)* / 0.6 (ratio-calc)<br>highest @ 10%: 13.8 (pg/ml)* / 1.5 (ratio-calc)
Jacobs et al. (2014)	MMP-8	*MMP8*	hPDL cells ($) (n.g./n.g., n.g, method n.g., P4-6, subconfluency)	static	12h	1% (0.7 cN/mm^2), 5% (3 cN/mm^2), 10% (5.2 cN/mm^2)	Flexercell Strain Unit FX 3000 + Bioflex® Plates + vacuum	equibiaxial	n.g.	n.g.	MMP8: increase (ELISA)<br>TIMP1/MMP8: increase followed by decrease (ELISA)	MMP8 @10%: 38.8 (pg/ml) / 12.1 (ratio-calc)<br>TIMP1/MMP8 highest @ 5%: 17.2 (rel×1000) / 2 (ratio-calc ×1000)<br>TIMP1/MMP8 lowest @ 10%: 2 (rel×1000)* / 0.2 (ratio-calc ×1000)
Jacobs et al. (2014)	PGE_2	PGE_2	hPDL cells ($) (n.g./n.g., n.g, method n.g., P4-6, subconfluency)	static	12h	1% (0.7cN/mm^2), 5% (3cN/mm^2), 10% (5.2cN/mm^2)	Flexercell Strain Unit FX 3000 + Bioflex® Plates + vacuum	equibiaxial	n.a.	n.a.	increase (ELISA)	10%: 47.9 (pg/ml), ration can not be calculated
Jacobs et al. (2014)	TIMP-1	*TIMP1*	hPDL cells ($) (n.g./n.g., n.g, method n.g., P4-6, subconfluency)	static	12h	1% (0.7cN/mm^2), 5% (3cN/mm^2), 10% (5.2cN/mm^2)	Flexercell Strain Unit FX 3000 + Bioflex® Plates + vacuum	equibiaxial	n.g.	n.g.	TIMP1: increase (ELISA)<br>TIMP1/MMP8: increase followed by decrease (ELISA)	TIMP1 @10%: 71.2 (qng/ml) / 4.5 (ratio-calc)<br>TIMP1/MMP8 highest @ 5%: 17.2 (rel×1000) / 2 (ratio-calc ×1000)<br>TIMP1/MMP8 lowest @ 10%: 2 (rel×1000)* / 0.2 (ratio-calc ×1000)
Jacobs et al. (2015)	OPG	*TNFRSF11B*	hPDL cells ($) (n.g./n.g., n.g, method n.g., P4-6, subconfluency)	static	12h	5% (3cN/mm^2), 10% (5.2cN/mm^2)	Flexercell Strain Unit FX 3000 + Bioflex® Plates + vacuum	equibiaxial	OPG: increase (qPCR, actin + GAPDH)<br>RANKL/OPG: temporary decrease	OPG @ 5%: 2.9 (FC)*<br>RANKL/OPG @ 5%: 0.2 (ratio)*	increase (ELISA)	5%: 13.7 (ng/ml)* / 3.1 (ratio-calc)
Jacobs et al. (2015)	RANKL	*TNFSF11*	hPDL cells ($) (n.g./n.g., n.g, method n.g., P4-6, subconfluency)	static	12h	5% (3cN/mm^2), 10% (5.2cN/mm^2)	Flexercell Strain Unit FX 3000 + Bioflex® Plates + vacuum	equibiaxial	RANKL: decrease followed by increase (qPCR, actin + GAPDH)<br>RANKL/OPG: temporary decrease	RANKL lowest @ 5%: 0.6 (FC)*<br>RANKL highest @ 10%: 2.4 (FC)*<br>RANKL/OPG @ 5%: 0.2 (ratio)*	n.g.	n.g.
Jacobs et al. (2018)	COX-2	*PTGS2*	hPDL cells ($) (n.g./n.g., n.g, method n.g., P4-6, subconfluency)	static	12h	3% (2cN/mm^2)	Flexercell Strain Unit FX 3000 + Bioflex® Plates + vacuum	equibiaxial	increase (qPCR; actin + GAPDH)	3.8 (FC)†	n.g.	n.g.
Jacobs et al. (2018)	MMP-8	*MMP8*	hPDL cells ($) (n.g./n.g., n.g, method n.g., P4-6, subconfluency)	static	12h	3% (2cN/mm^2)	Flexercell Strain Unit FX 3000 + Bioflex® Plates + vacuum	equibiaxial	n.g.	n.g.	increase (ELISA)	17.7 (pg/ml)* / 1.5 (ratio-calc)

[a] Entry given as reported in the study.
[b] All official gene symbols come from the HUGO Gene Nomenclature Committee (HGNC; URL: https://www.genenames.org) after checking specificity of primers with Primer-BLAST.
[c] Gender/Sex of donors: "M" – male, "F" – female; Tooth type: "PM" – premolar, "M" – molar; Cell density: given in cells/well if not otherwise mentioned.
[d] Frequencies labeled bold orange were converted to hertz (Hz) according to its definition using the information reported in the study (in brackets)
[e] Force type deduced from the description of the force apparatus given by the authors.
[f] Gene and protein expression: 1. conclusion of change (increase, decrease…) was given according to the defined criteria in Figure 2; 2. different markers to describe the amount of change; † Information derived from figures using Engauge Digitizer; *Folds calculated by measuring the graphs, without using the Engauge Digitizer; No makers: Information derived from figures by description in the articles

Reference	Gene/ Analyte[a]	Official gene symbol / abbreviation[b]	Cell (age/gender of donors, tooth type, isolation method, passages used, cell density)[a,c]	Force type (stat./ dyn.)[a]	Force duration and frequency[d]	Force magnitude[a]	Force apparatus[a]	Force type: equibiaxial or uniaxial[e]	Gene expression: Increase, decrease, no change (method w/ reference gene); Methods: qPCR, sqPCR, Northern blot[f]	Gene expression: When it reaches peak and peak's magnitude (fold change; times or ratio; unclear = ?)[f]	Protein expression: Increase, decrease, no change (method w/ reference); Methods: ELISA, WB, RIA, EMSA, IF[f]	Protein expression: When it reaches peak and peak's magnitude (times or ratio; unclear = ?)[f]
Jacobs et al. (2018)	PGE_2	PGE_2	hPDL cells ($) (n.g./n.g., n.g, method n.g., P4-6, subconfluency)	static	12h	3% ($2cN/mm^2$)	Flexercell Strain Unit FX 3000 + Bioflex® Plates + vacuum	equibiaxial	n.a.	n.a.	no change (ELISA)	
Jacobs et al. (2018)	TIMP-1	*TIMP1*	hPDL cells ($) (n.g./n.g., n.g, method n.g., P4-6, subconfluency)	static	12h	3% ($2cN/mm^2$)	Flexercell Strain Unit FX 3000 + Bioflex® Plates + vacuum	equibiaxial	n.g.	n.g.	increase (ELISA)	1947 (pg/ml)* / 1.3 (ratio-calc)
Jiang and Hua (2016)	ALP	*ALPP*	hPDL cells (12-18/n.g., PM, exp, P n.g., n.g.)	dynamic	0.1Hz (5s stretch and 5s relaxation) for 6h, 12h, 24h, 48h	5%	Flexcell FX-5000 Tension System + flexible-bottomed six-well plates coated with type I collagen (Sigma) + vacuum	equibiaxial	increase (sqPCR, GAPDH)	24h: 2.7 (FC)*	increase (WB, GAPDH)	24h: no quantitative information is given
Jiang and Hua (2016)	OCN	*BGLAP*	hPDL cells (12-18/n.g., PM, exp, P n.g., n.g.)	dynamic	0.1Hz (5s stretch and 5s relaxation) for 6h, 12h, 24h, 48h	5%	Flexcell FX-5000 Tension System + flexible-bottomed six-well plates coated with type I collagen (Sigma) + vacuum	equibiaxial	increase (sqPCR, GAPDH)	24h: 3 (FC)*	increase (WB, GAPDH)	24h: no quantitative information is given
Jiang and Hua (2016)	RUNX2	*RUNX2*	hPDL cells (12-18/n.g., PM, exp, P n.g., n.g.)	dynamic	0.1Hz (5s stretch and 5s relaxation) for 6h, 12h, 24h, 48h	5%	Flexcell FX-5000 Tension System + flexible-bottomed six-well plates coated with type I collagen (Sigma) + vacuum	equibiaxial	increase (sqPCR, GAPDH)	24h: 2.9 (FC)*	increase (WB, GAPDH)	24h: no quantitative information is given
Kaku et al. (2019)	CSF1	*CSF1*	hPDLCs (n.g./n.g., n.g., exp, P4-6, $1×10^5$)	dynamic	**0.5Hz** (30 cyc/min) for 48h	12%	Flexcell FX-2000 + silicon membrane + vaccum	equibiaxial	n.g.	n.g.	increase (ELISA)	301.9 (pg/ml)† / 3.1 (ratio-calc)
Kaku et al. (2019)	IL-1B	*IL1B*	hPDLCs (n.g./n.g., n.g., exp, P4-6, $1×10^5$)	dynamic	**0.5Hz** (30 cyc/min) for 48h	12%	Flexcell FX-2000 + silicon membrane + vaccum	equibiaxial	n.g.	n.g.	increase (ELISA)	12.1 (pg/ml)† / 1.6 (ratio-calc)
Kaku et al. (2019)	RANKL	*TNFSF11*	hPDLCs (n.g./n.g., n.g., exp, P4-6, $1×10^5$)	dynamic	**0.5Hz** (30 cyc/min) for 48h	12%	Flexcell FX-2000 + silicon membrane + vaccum	equibiaxial	n.g.	n.g.	increase (ELISA)	27.8 (pg/ml)† / 3.3 (ratio-calc)
Kaku et al. (2019)	TNFα	*TNF*	hPDLCs (n.g./n.g., n.g., exp, P4-6, $1×10^5$)	dynamic	**0.5Hz** (30 cyc/min) for 48h	12%	Flexcell FX-2000 + silicon membrane + vaccum	equibiaxial	n.g.	n.g.	increase (ELISA)	4.7 (pg/ml)† / 4.8 (ratio-calc)
Kanzaki et al. (2006)	OPG	*TNFRSF11B*	hPDL cells (n.g./n.g., n.g., exp, P4-8, n.g.)	dynamic	**0.5Hz** (1s stretch/1s relaxation) sqPCR for 48h; ELISA for 72h	15%	Flexercell Strain-Unit + type I collagen-coated silicone membrane + vacuum	equibiaxial	increase (sqPCR, β-actin)	1.7 (ratio)*	increase (ELISA)	277.1 (pmol/L)* / 1.3 (ratio-calc)
Kanzaki et al. (2006)	RANKL	*TNFSF11*	hPDL cells (n.g./n.g., n.g., exp, P4-8, n.g.)	dynamic	**0.5Hz** (1s stretch/1s relaxation) for 48h	15%	Flexercell Strain-Unit + type I collagen-coated silicone membrane + vacuum	equibiaxial	increase (sqPCR, β-actin)	12.9 (ratio)*	n.g.	n.g.
Kanzaki et al. (2006)	TGF-β	*TGFB1*	hPDL cells (n.g./n.g., n.g., exp, P4-8, n.g.)	dynamic	**0.5Hz** (1s stretch/1s relaxation) sqPCR for 48h; ELISA for 6h, 24h, 48h, 72h	15%	Flexercell Strain-Unit + type I collagen-coated silicone membrane + vacuum	equibiaxial	increase (sqPCR, β-actin)	1.5 (ratio)*	increase (ELISA)	72h: 2 (ng/ml)* / 1.1 (ratio-calc)
Kanzaki et al. (2019)	ARRAY	ARRAY	immortalized hPDLCs (n.g./n.g., n.g., gene transfection, n.g., n.g.)	dynamic	**0.5Hz** (1s stretch/1s relaxation) for 24h	15%	Flexercell Strain-Unit + type I collagen-coated silicone membrane + vacuum (Kanzaki et al 2006)	equibiaxial	SurePrint G3 Human miRNA microarray 8 × 60 K miRBase 16.0 (Agilent Technologies)	too many	n.a.	n.a.
Kanzaki et al. (2019)	OPG	*TNFRSF11B*	immortalized hPDLCs (n.g./n.g., n.g., gene transfection, n.g., n.g.)	dynamic	**0.5Hz** (1s stretch/1s relaxation) for 24h	15%	Flexercell Strain-Unit + type I collagen-coated silicone membrane + vacuum (Kanzaki et al 2006)	equibiaxial	increase (qPCR, RPS18)	2.2 (FC)*	increase (WB, n.g.)<br>increase (ELISA)	WB: 1.8 (ratio)*<br>ELISA: 16.3 (ng/ml)* / 1.3 (ratio-calc)
Kanzaki et al. (2019)	RANKL	*TNFSF11*	immortalized hPDLCs (n.g./n.g., n.g., gene transfection, n.g., n.g.)	dynamic	**0.5Hz** (1s stretch/1s relaxation) for 24h	15%	Flexercell Strain-Unit + type I collagen-coated silicone membrane + vacuum (Kanzaki et al 2006)	equibiaxial	increase (qPCR, RPS18)	9.9 (FC)*	n.g.	n.g.
Kikuiri et al. (2000)	ecNOS	*NOS3*	hPDL cells (14-17/n.g., PM, dig, P5-10, $4×10^5$)	dynamic	**0.1Hz** (elongation for 5s, relaxation for 5s) sqPCR for 3h,12h; WB for 3h, 6h	18%	Flexercell Strain Unit (Shimizu 1994) + flexible silicon rubber bottoms + vacuum	equibiaxial	ecNOS (sqPCR, GAPDH), expressed in both control and experimental groups	no quantitative information is given	control groups (WB): no expression<br>experimental group (WB): strong expression	no quantitative information is given
Kikuiri et al. (2000)	iNOS	*NOS2*	hPDL cells (14-17/n.g., PM, dig, P5-10, $4×10^5$)	dynamic	**0.1Hz** (elongation for 5s, relaxation for 5s) for 12h	18%	Flexercell Strain Unit (Shimizu 1994) + flexible silicon rubber bottoms + vacuum	equibiaxial	no expression (sqPCR, GAPDH)		no expression (WB)	

[a] Entry given as reported in the study.
[b] All official gene symbols come from the HUGO Gene Nomenclature Committee (HGNC; URL: https://www.genenames.org) after checking specificity of primers with Primer-BLAST.
[c] Gender/Sex of donors: "M" – male, "F" – female; Tooth type: "PM" – premolar, "M" – molar; Cell density: given in cells/well if not otherwise mentioned.
[d] Frequencies labeled bold orange were converted to hertz (Hz) according to its definition using the information reported in the study (in brackets)
[e] Force type deduced from the description of the force apparatus given by the authors.
[f] Gene and protein expression: 1. conclusion of change (increase, decrease…) was given according to the defined criteria in Figure 2; 2. different markers to describe the amount of change; † Information derived from figures using Engauge Digitizer; *Folds calculated by measuring the graphs, without using the Engauge Digitizer; No makers: Information derived from figures by description in the articles

Reference	Gene/ Analyte[a]	Official gene symbol / abbreviation[b]	Cell (age/gender of donors, tooth type, isolation method, passages used, cell density)[a,c]	Force type (stat./ dyn.)[a]	Force duration and frequency[d]	Force magnitude[a]	Force apparatus[a]	Force type: equibiaxial or uniaxial[e]	Gene expression: Increase, decrease, no change (method w/ reference gene); Methods: qPCR, sqPCR, Northern blot[f]	Gene expression: When it reaches peak and peak's magnitude (fold change; times or ratio; unclear = ?)[f]	Protein expression: Increase, decrease, no change (method w/ reference); Methods: ELISA, WB, RIA, EMSA, IF[f]	Protein expression: When it reaches peak and peak's magnitude (times or ratio; unclear = ?)[f]
Kim et al. (2007)	Osteocalcin	*BGLAP*	hPDL cells (n.g./n.g., n.g., exp, P5-10, 5×10⁴)	dynamic	**0.1Hz** (6 cyc/min: strain for 5s followed by a 5s relaxation) for 6d	9%	Flexcell strain unit FX-2000 + 35-mm Flexercell plate dish + vacuum	equibiaxial	increase (sqPCR, GAPDH)	75 (rel)* / 1.7 (ratio-calc)	n.g.	n.g.
Kim et al. (2007)	UNCL	*UNC50*	hPDL cells (n.g./n.g., n.g., exp, P5-10, 5×10⁴)	dynamic	**0.1Hz** (6 cyc/min: strain for 5s followed by a 5s relaxation) for 6d	9%	Flexcell strain unit FX-2000 + 35-mm Flexercell plate dish + vacuum	equibiaxial	increase (sqPCR, GAPDH)	117 (rel)* / 1.3 (ratio-calc)	n.g.	n.g.
Kletsas et al. (2002)	c-Fos	*FOS*	hPDL cells (n.g./n.g., n.g., exp, P3-4, 90% confluency)	static	0.5h, 1h, 3h, 6h, 12h, 24h	2.5%	Petriperm dish + plexiglass template with a convex surface + weight	equibiaxial	n.g.	n.g.	increase (WB)	3h: 225% (rel)* / 2.3 (ratio-calc)
Kletsas et al. (2002)	c-Jun	*JUN*	hPDL cells (n.g./n.g., n.g., exp, P3-4, 90% confluency)	static	0.5h, 1h, 3h, 6h, 12h, 24h	2.5%	Petriperm dish + plexiglass template with a convex surface + weight	equibiaxial	n.g.	n.g.	increase (WB)	1h: 186% (rel)* / 1.9 (ratio-calc)
Konstantonis et al. (2014)	ALP	*ALPP*	hPDL cells (n.g./n.g., n.g., exp, P3-6 & P20-24, n.g.)	dynamic	1Hz for 12h	8%	six station stretching apparatus + optically transparent silicone dishes pre-coated with fibronectin + moving clamp (Neidlinger-Wilke et al 2001)	uniaxial	"young cells" (P3-6): increase (qPCR, GAPDH)<br>"senescent cells" (P20-24): increase (qPCR, GAPDH)	"young cells":150% (rel)* / 1.5 (ratio-calc)<br>"senescent cells": 50% (rel)* / 1.5 (ratio-calc)	"young cells": ALP activity increase (colorimetric assay)<br>"senescent cells": ALP activity increase (colorimetric assay)	"young cells":150% (rel)* / 1.5 (ratio-calc)<br>"senescent cells": 95% (rel)* / 1.6 (ratio-calc)
Konstantonis et al. (2014)	c-fos	*FOS*	hPDL cells (n.g./n.g., n.g., exp, P3-6 & P20-24, n.g.)	dynamic	1Hz for 0.5h, 1h, 1.5h, 2h, 2.5h, 3h	8%	six station stretching apparatus + optically transparent silicone dishes pre-coated with fibronectin + moving clamp (Neidlinger-Wilke et al 2001)	uniaxial	"young cells" (P3-6): increase followed by decrease (qPCR, GAPDH)<br>"senescent cells" (P20-24): increase followed by decrease (qPCR, GAPDH)	"young cells" highest @ 0.5h: 1300% (rel)* / 1.3 (ratio-calc)<br>"young cells" lowest @ 3h: 50% (rel)* / 0.5 (ratio-calc)<br>"senescent cells" highest @ 1h: 740% (rel)* / 7.4 (ratio-calc)<br>"senescent cells" lowest @ 3h: 50% (rel)* / 0.5 (ratio-calc)	n.g.	n.g.
Konstantonis et al. (2014)	ERK / p-ERK	MAPK3; MAPK1	hPDL cells (n.g./n.g., n.g., exp, P3-6 & P20-24, n.g.)	dynamic	1Hz for 15min, 30min, 60min, 180min	8%	six station stretching apparatus + optically transparent silicone dishes pre-coated with fibronectin + moving clamp (Neidlinger-Wilke et al 2001)	uniaxial	n.g.	n.g.	"young cells" (P3-6): not reported<br>p-ERK in "senescent cells" (P20-24) (WB, actin): temporary increase<br>ERK in "senescent cells" (WB, actin): no change	no quantitative information is given
Konstantonis et al. (2014)	JNK / p-JNK	*MAPK8*	hPDL cells (n.g./n.g., n.g., exp, P3-6 & P20-24, n.g.)	dynamic	1Hz for 15min, 30min, 60min, 180min	8%	six station stretching apparatus + optically transparent silicone dishes pre-coated with fibronectin + moving clamp (Neidlinger-Wilke et al 2001)	uniaxial	n.g.	n.g.	"young cells" (P3-6): not reported<br>p-JNK in "senescent cells" (P20-24) (WB, actin): temporary increase<br>JNK in "senescent cells" (WB, actin): no change	no quantitative information is given
Konstantonis et al. (2014)	p38-MAPK / p-p38-MAPK	*MAPK14*	hPDL cells (n.g./n.g., n.g., exp, P3-6 & P20-24, n.g.)	dynamic	1Hz for 15min, 30min, 60min, 180min	8%	six station stretching apparatus + optically transparent silicone dishes pre-coated with fibronectin + moving clamp (Neidlinger-Wilke et al 2001)	uniaxial	n.g.	n.g.	"young cells" (P3-6): not reported<br>p-p38 in "senescent cells" (P20-24) (WB, actin): temporary increase<br>p38 in "senescent cells" (WB, actin): no change	no quantitative information is given
Kook and Lee (2012)	CDK2	*CDK2*	hPLF (20-30/M, PM, dig, P4-7, 80% confluency)	static	1h	1.5%	FX-4000 Tension Plus System + flexible bottomed six-well plates coated with COL I + vacuum	equibiaxial	n.g.	n.g.	decrease (WB, α-tubulin)	1 (?) * / 0.4 (ratio-calc)
Kook and Lee (2012)	CDK4	*CDK4*	hPLF (20-30/M, PM, dig, P4-7, 80% confluency)	static	1h	1.5%	FX-4000 Tension Plus System + flexible bottomed six-well plates coated with COL I + vacuum	equibiaxial	n.g.	n.g.	decrease (WB, α-tubulin)	1 (?) * / 0.3 (ratio-calc)
Kook and Lee (2012)	CyclinA	*CCNA1; CCNA2*	hPLF (20-30/M, PM, dig, P4-7, 80% confluency)	static	1h	1.5%	FX-4000 Tension Plus System + flexible bottomed six-well plates coated with COL I + vacuum	equibiaxial	n.g.	n.g.	decrease (WB, α-tubulin)	no quantitative information is given
Kook and Lee (2012)	CyclinD1	*CCND1*	hPLF (20-30/M, PM, dig, P4-7, 80% confluency)	static	1h	1.5%	FX-4000 Tension Plus System + flexible bottomed six-well plates coated with COL I + vacuum	equibiaxial	n.g.	n.g.	decrease (WB, α-tubulin)	1 (?) * / 0.4 (ratio-calc)
Kook and Lee (2012)	CyclinE	*CCNE1*	hPLF (20-30/M, PM, dig, P4-7, 80% confluency)	static	1h	1.5%	FX-4000 Tension Plus System + flexible bottomed six-well plates coated with COL I + vacuum	equibiaxial	n.g.	n.g.	decrease (WB, α-tubulin)	1 (?) * / 0.3 (ratio-calc)
Kook and Lee (2012)	ERK / p-ERK	MAPK3; MAPK1	hPLF (20-30/M, PM, dig, P4-7, 80% confluency)	static	1h	1.5%	FX-4000 Tension Plus System + flexible bottomed six-well plates coated with COL I + vacuum	equibiaxial	n.g.	n.g.	p-ERK: increase (ELISA)	0.5 (ng/ml)* / 3 (ratio-calc)
Kook and Lee (2012)	ERK / p-ERK	MAPK3; MAPK1	hPLF (20-30/M, PM, dig, P4-7, 80% confluency)	static	1h	1.5%, 3%, 5%, 10%	FX-4000 Tension Plus System + flexible bottomed six-well plates coated with COL I + vacuum	equibiaxial	n.g.	n.g.	p-ERK: temporary increase (WB, ERK)	p-ERK @ 1.5%: no quantitative information is given
Kook and Lee (2012)	JNK / p-JNK	*MAPK8*	hPLF (20-30/M, PM, dig, P4-7, 80% confluency)	static	1h	1.5%, 3%, 5%, 10%	FX-4000 Tension Plus System + flexible bottomed six-well plates coated with COL I + vacuum	equibiaxial	n.g.	n.g.	p-JNK: temporary increase (WB, JNK)	p-JNK @ 1.5%: no quantitative information given
Kook and Lee (2012)	JNK / p-JNK	*MAPK8*	hPLF (20-30/M, PM, dig, P4-7, 80% confluency)	static	1h	1.5%	FX-4000 Tension Plus System + flexible bottomed six-well plates coated with COL I + vacuum	equibiaxial	n.g.	n.g.	p-JNK: increase (WB, JNK)	1.8 (OD at 450nm) * / 7 (ratio-calc)
Kook and Lee (2012)	P21	*CDKN1A*	hPLF (20-30/M, PM, dig, P4-7, 80% confluency)	static	1h	1.5%, 3%, 5%, 10%	FX-4000 Tension Plus System + flexible bottomed six-well plates coated with COL I + vacuum	equibiaxial	n.g.	n.g.	increase (WB, α-tubulin)	3%: 3.6 (ratio)†
Kook and Lee (2012)	p21 / p-p21	*TCEAL1*	hPLF (20-30/M, PM, dig, P4-7, 80% confluency)	static	1h	1.5%	FX-4000 Tension Plus System + flexible bottomed six-well plates coated with COL I + vacuum	equibiaxial	n.g.	n.g.	p-p21: increase (WB, α-tubulin)	p-p21: 3.2 (FC) * / 3.2 (ratio)*

[a] Entry given as reported in the study.
[b] All official gene symbols come from the HUGO Gene Nomenclature Committee (HGNC; URL: https://www.genenames.org) after checking specificity of primers with Primer-BLAST.
[c] Gender/Sex of donors: "M" – male, "F" – female; Tooth type: "PM" – premolar, "M" – molar; Cell density: given in cells/well if not otherwise mentioned.
[d] Frequencies labeled bold orange were converted to hertz (Hz) according to its definition using the information reported in the study (in brackets)
[e] Force type deduced from the description of the force apparatus given by the authors.
[f] Gene and protein expression: 1. conclusion of change (increase, decrease…) was given according to the defined criteria in Figure 2; 2. different markers to describe the amount of change; † Information derived from figures using Engauge Digitizer; *Folds calculated by measuring the graphs, without using the Engauge Digitizer; No makers: Information derived from figures by description in the articles

Reference	Gene/ Analyte[a]	Official gene symbol / abbreviation[b]	Cell (age/gender of donors, tooth type, isolation method, passages used, cell density)[a,c]	Force type (stat./ dyn.)[a]	Force duration and frequency[d]	Force magnitude[a]	Force apparatus[a]	Force type: equibiaxial or uniaxial[e]	Gene expression: Increase, decrease, no change (method w/ reference gene); Methods: qPCR, sqPCR, Northern blot[f]	Gene expression: When it reaches peak and peak's magnitude (fold change; times or ratio; unclear = ?)[f]	Protein expression: Increase, decrease, no change (method w/ reference); Methods: ELISA, WB, RIA, EMSA, IF[f]	Protein expression: When it reaches peak and peak's magnitude (times or ratio; unclear = ?)[f]
Kook and Lee (2012)	P27	*CDKN1B*	hPLF (20-30/M, PM, dig, P4-7, 80% confluency)	static	1h	1.5%, 3%, 5%, 10%	FX-4000 Tension Plus System + flexible bottomed six-well plates coated with COL I + vacuum	equibiaxial	n.g.	n.g.	increase (WB, α-tubulin)	10%: 1.6 (ratio)†
Kook and Lee (2012)	p38-MAPK / p-p38-MAPK	*MAPK14*	hPLF (20-30/M, PM, dig, P4-7, 80% confluency)	static	1h	1.5%, 3%, 5%, 10%	FX-4000 Tension Plus System + flexible bottomed six-well plates coated with COL I + vacuum	equibiaxial	n.g.	n.g.	p-p38: temporary increase (WB, p38)	p-p38 @ 1.5%: no quantitative information is given
Kook and Lee (2012)	p38-MAPK / p-p38-MAPK	*MAPK14*	hPLF (20-30/M, PM, dig, P4-7, 80% confluency)	static	1h	1.5%	FX-4000 Tension Plus System + flexible bottomed six-well plates coated with COL I + vacuum	equibiaxial	n.g.	n.g.	p-p38: increase (ELISA)	4.2 (ng/ml)* / 6.8 (ratio-calc)
Kook and Lee (2012)	PCNA	*PCNA*	hPLF (20-30/M, PM, dig, P4-7, 80% confluency)	static	1h	1.5%	FX-4000 Tension Plus System + flexible bottomed six-well plates coated with COL I + vacuum	equibiaxial	n.g.	n.g.	decrease (WB, α-tubulin)	no quantitative information is given
Lee et al. (2012)	CCL-20	*CCL20*	hPDLF-hTERT (n.g./n.g., PM, dig, P n.g., 70% confluent)	dynamic	**0.2Hz** (12cyc/min: stretch for 2.5s followed by 2.5s of relaxation) for 3h, 6h, 12h, 24h, 48h	12%	Flexercell FX-4000 Strain Unit + 35-mm flexible-bottomed Uniflex culture plates with a centrally located rectangular portion (15.25 mm×24.18 mm) coated with type I collagen + vacuum	uniaxial	increase (sqPCR, GAPDH)	48h: 4.4 (ratio)†	n.g.	n.g.
Lee et al. (2012)	CCL-20	*CCL20*	hPDLF-hTERT (n.g./n.g., PM, dig, P n.g., 70% confluent)	dynamic	**0.2Hz** (12cyc/min: stretch for 2.5s followed by 2.5s of relaxation) for 24h	3%, 6%, 12%,15%	Flexercell FX-4000 Strain Unit + 35-mm flexible-bottomed Uniflex culture plates with a centrally located rectangular portion (15.25 mm×24.18 mm) coated with type I collagen + vacuum	uniaxial	increase (sqPCR, GAPDH)	12%: 4.0 (ratio)†	n.g.	n.g.
Lee et al. (2012)	hBD-1	*DEFB1*	hPDLF-hTERT (n.g./n.g., PM, dig, P n.g., 70% confluent)	dynamic	**0.2Hz** (12cyc/min: stretch for 2.5s followed by 2.5s of relaxation) for 3h, 6h, 12h, 24h, 48h	12%	Flexercell FX-4000 Strain Unit + 35-mm flexible-bottomed Uniflex culture plates with a centrally located rectangular portion (15.25 mm×24.18 mm) coated with type I collagen + vacuum	uniaxial	no change		n.g.	n.g.
Lee et al. (2012)	hBD-1	*DEFB1*	hPDLF-hTERT (n.g./n.g., PM, dig, P n.g., 70% confluent)	dynamic	**0.2Hz** (12cyc/min: stretch for 2.5s followed by 2.5s of relaxation) for 24h	3%, 6%, 12%, 15%	Flexercell FX-4000 Strain Unit + 35-mm flexible-bottomed Uniflex culture plates with a centrally located rectangular portion (15.25 mm×24.18 mm) coated with type I collagen + vacuum	uniaxial	no change		n.g.	n.g.
Lee et al. (2012)	hBD-2	*DEFB4A*	hPDLF-hTERT (n.g./n.g., PM, dig, P n.g., 70% confluent)	dynamic	**0.2Hz** (12cyc/min: stretch for 2.5s followed by 2.5s of relaxation) for 3h, 6h, 12h, 24h, 48h	12%	Flexercell FX-4000 Strain Unit + 35-mm flexible-bottomed Uniflex culture plates with a centrally located rectangular portion (15.25 mm×24.18 mm) coated with type I collagen + vacuum	uniaxial	increase (sqPCR, GAPDH)	12h…24h:.5.6 (ratio)†	n.g.	n.g.
Lee et al. (2012)	hBD-2	*DEFB4A*	hPDLF-hTERT (n.g./n.g., PM, dig, P n.g., 70% confluent)	dynamic	**0.2Hz** (12cyc/min: stretch for 2.5s followed by 2.5s of relaxation) for 24h	3%, 6%, 12%, 15%	Flexercell FX-4000 Strain Unit + 35-mm flexible-bottomed Uniflex culture plates with a centrally located rectangular portion (15.25 mm×24.18 mm) coated with type I collagen + vacuum	uniaxial	increase (sqPCR, GAPDH)	15%: 5.9 (ratio)†	n.g.	n.g.
Lee et al. (2012)	hBD-3	*DEFB103B*	hPDLF-hTERT (n.g./n.g., PM, dig, P n.g., 70% confluent)	dynamic	**0.2Hz** (12cyc/min: stretch for 2.5s followed by 2.5s of relaxation) for 3h, 6h, 12h, 24h, 48h	12%	Flexercell FX-4000 Strain Unit + 35-mm flexible-bottomed Uniflex culture plates with a centrally located rectangular portion (15.25 mm×24.18 mm) coated with type I collagen + vacuum	uniaxial	increase (sqPCR, GAPDH)	48h: 3 (ratio)†	n.g.	n.g.
Lee et al. (2012)	hBD-3	*DEFB103B*	hPDLF-hTERT (n.g./n.g., PM, dig, P n.g., 70% confluent)	dynamic	**0.2Hz** (12cyc/min: stretch for 2.5s followed by 2.5s of relaxation) for 24h	3%, 6%, 12%, 15%	Flexercell FX-4000 Strain Unit + 35-mm flexible-bottomed Uniflex culture plates with a centrally located rectangular portion (15.25 mm×24.18 mm) coated with type I collagen + vacuum	uniaxial	increase (sqPCR, GAPDH)	15%: 2.8 (ratio)†	n.g.	n.g.

[a] Entry given as reported in the study.
[b] All official gene symbols come from the HUGO Gene Nomenclature Committee (HGNC; URL: https://www.genenames.org) after checking specificity of primers with Primer-BLAST.
[c] Gender/Sex of donors: "M" – male, "F" – female; Tooth type: "PM" – premolar, "M" – molar; Cell density: given in cells/well if not otherwise mentioned.
[d] Frequencies labeled bold orange were converted to hertz (Hz) according to its definition using the information reported in the study (in brackets)
[e] Force type deduced from the description of the force apparatus given by the authors.
[f] Gene and protein expression: 1. conclusion of change (increase, decrease…) was given according to the defined criteria in Figure 2; 2. different markers to describe the amount of change; † Information derived from figures using Engauge Digitizer; *Folds calculated by measuring the graphs, without using the Engauge Digitizer; No makers: Information derived from figures by description in the articles

Reference	Gene/ Analyte[a]	Official gene symbol / abbreviation[b]	Cell (age/gender of donors, tooth type, isolation method, passages used, cell density)[a,c]	Force type (stat./ dyn.)[a]	Force duration and frequency[d]	Force magnitude[a]	Force apparatus[a]	Force type: equibiaxial or uniaxial[e]	Gene expression: Increase, decrease, no change (method w/ reference gene); Methods: qPCR, sqPCR, Northern blot[f]	Gene expression: When it reaches peak and peak's magnitude (fold change; times or ratio; unclear = ?)[f]	Protein expression: Increase, decrease, no change (method w/ reference); Methods: ELISA, WB, RIA, EMSA, IF[f]	Protein expression: When it reaches peak and peak's magnitude (times or ratio; unclear = ?)[f]
Lee et al. (2012)	IL-1β	*IL1B*	hPDLF-hTERT (n.g./n.g., PM, dig, P n.g., 70% confluent)	dynamic	**0.2Hz** (12cyc/min: stretch for 2.5s followed by 2.5s of relaxation) for 3h, 6h, 12h, 24h, 48h	12%	Flexercell FX-4000 Strain Unit + 35-mm flexible-bottomed Uniflex culture plates with a centrally located rectangular portion (15.25 mm×24.18 mm) coated with type I collagen + vacuum	uniaxial	increase (sqPCR, GAPDH)	48h: 3 (ratio)†	n.g.	n.g.
Lee et al. (2012)	IL-1β	*IL1B*	hPDLF-hTERT (n.g./n.g., PM, dig, P n.g., 70% confluent)	dynamic	**0.2Hz** (12cyc/min: stretch for 2.5s followed by 2.5s of relaxation) for 24h	3%, 6%, 12%, 15%	Flexercell FX-4000 Strain Unit + 35-mm flexible-bottomed Uniflex culture plates with a centrally located rectangular portion (15.25 mm×24.18 mm) coated with type I collagen + vacuum	uniaxial	increase (sqPCR, GAPDH)	15%: 3.3 (ratio)†	n.g.	n.g.
Lee et al. (2012)	IL-8	*CXCL8*	hPDLF-hTERT (n.g./n.g., PM, dig, P n.g., 70% confluent)	dynamic	**0.2Hz** (12cyc/min: stretch for 2.5s followed by 2.5s of relaxation) for 3h, 6h, 12h, 24h, 48h	12%	Flexercell FX-4000 Strain Unit + 35-mm flexible-bottomed Uniflex culture plates with a centrally located rectangular portion (15.25 mm×24.18 mm) coated with type I collagen + vacuum	uniaxial	increase (sqPCR, GAPDH)	48h: 2.6 (ratio)†	n.g.	n.g.
Lee et al. (2012)	IL-8	*CXCL8*	hPDLF-hTERT (n.g./n.g., PM, dig, P n.g., 70% confluent)	dynamic	**0.2Hz** (12cyc/min: stretch for 2.5s followed by 2.5s of relaxation) for 24h	3%, 6%, 12%,15%	Flexercell FX-4000 Strain Unit + 35-mm flexible-bottomed Uniflex culture plates with a centrally located rectangular portion (15.25 mm×24.18 mm) coated with type I collagen + vacuum	uniaxial	increase (sqPCR, GAPDH)	15%: 2.3 (ratio)†	n.g.	n.g.
Lee et al. (2012)	SIRT1	*SIRT1*	hPDLF-hTERT (n.g./n.g., PM, dig, P n.g., 70% confluent)	dynamic	**0.2Hz** (12cyc/min: stretch for 2.5s followed by 2.5s of relaxation) for 3h, 6h, 12h, 24h, 48h	12%	Flexercell FX-4000 Strain Unit + 35-mm flexible-bottomed Uniflex culture plates with a centrally located rectangular portion (15.25 mm×24.18 mm) coated with type I collagen + vacuum	uniaxial	increase (sqPCR, GADPDH)	24h: 3.4 (ratio)†	increase (WB, β-actin)	24h: 3.4 (ratio)†
Lee et al. (2012)	SIRT1	*SIRT1*	hPDLF-hTERT (n.g./n.g., PM, dig, P n.g., 70% confluent)	dynamic	**0.2Hz** (12cyc/min: stretch for 2.5s followed by 2.5s of relaxation) for 24h	3%, 6%, 12%, 15%	Flexercell FX-4000 Strain Unit + 35-mm flexible-bottomed Uniflex culture plates with a centrally located rectangular portion (15.25 mm×24.18 mm) coated with type I collagen + vacuum	uniaxial	increase (sqPCR, GAPDH)	12%: 3.2 (ratio)†	increase (WB, β-actin)	12%: 3.3 (ratio)†
Lee et al. (2012)	TLR-2	*TLR2*	hPDLF-hTERT (n.g./n.g., PM, dig, P n.g., 70% confluent)	dynamic	**0.2Hz** (12cyc/min: stretch for 2.5s followed by 2.5s of relaxation) for 3h, 6h, 12h, 24h, 48h	12%	Flexercell FX-4000 Strain Unit + 35-mm flexible-bottomed Uniflex culture plates with a centrally located rectangular portion (15.25 mm×24.18 mm) coated with type I collagen + vacuum	uniaxial	increase (sqPCR, GAPDH)	24h: 6.5 (ratio)†	n.g.	n.g.
Lee et al. (2012)	TLR-2	*TLR2*	hPDLF-hTERT (n.g./n.g., PM, dig, P n.g., 70% confluent)	dynamic	**0.2Hz** (12cyc/min: stretch for 2.5s followed by 2.5s of relaxation) for 24h	3%, 6%, 12%, 15%	Flexercell FX-4000 Strain Unit + 35-mm flexible-bottomed Uniflex culture plates with a centrally located rectangular portion (15.25 mm×24.18 mm) coated with type I collagen + vacuum	uniaxial	increase (sqPCR, GAPDH)	12%: 6.3 (ratio)†	n.g.	n.g.
Lee et al. (2012)	TLR-4	*TLR4*	hPDLF-hTERT (n.g./n.g., PM, dig, P n.g., 70% confluent)	dynamic	**0.2Hz** (12cyc/min: stretch for 2.5s followed by 2.5s of relaxation)for 3h, 6h, 12h, 24h, 48h	12%	Flexercell FX-4000 Strain Unit + 35-mm flexible-bottomed Uniflex culture plates with a centrally located rectangular portion (15.25 mm×24.18 mm) coated with type I collagen + vacuum	uniaxial	increase (sqPCR, GAPDH)	24h: 4.6 (ratio)†	n.g.	n.g.
Lee et al. (2012)	TLR-4	*TLR4*	hPDLF-hTERT (n.g./n.g., PM, dig, P n.g., 70% confluent)	dynamic	**0.2Hz** (12cyc/min: stretch for 2.5s followed by 2.5s of relaxation) for 24h	3%, 6%, 12%, 15%	Flexercell FX-4000 Strain Unit + 35-mm flexible-bottomed Uniflex culture plates with a centrally located rectangular portion (15.25 mm×24.18 mm) coated with type I collagen + vacuum	uniaxial	increase (sqPCR, GAPDH)	12%: 4.6 (ratio)†	n.g.	n.g.

[a] Entry given as reported in the study.
[b] All official gene symbols come from the HUGO Gene Nomenclature Committee (HGNC; URL: https://www.genenames.org) after checking specificity of primers with Primer-BLAST.
[c] Gender/Sex of donors: "M" – male, "F" – female; Tooth type: "PM" – premolar, "M" – molar; Cell density: given in cells/well if not otherwise mentioned.
[d] Frequencies labeled bold orange were converted to hertz (Hz) according to its definition using the information reported in the study (in brackets)
[e] Force type deduced from the description of the force apparatus given by the authors.
[f] Gene and protein expression: 1. conclusion of change (increase, decrease…) was given according to the defined criteria in Figure 2; 2. different markers to describe the amount of change; † Information derived from figures using Engauge Digitizer; *Folds calculated by measuring the graphs, without using the Engauge Digitizer; No makers: Information derived from figures by description in the articles

Reference	Gene/ Analyte[a]	Official gene symbol / abbreviation[b]	Cell (age/gender of donors, tooth type, isolation method, passages used, cell density)[a,c]	Force type (stat./ dyn.)[a]	Force duration and frequency[d]	Force magnitude[a]	Force apparatus[a]	Force type: equibiaxial or uniaxial[e]	Gene expression: Increase, decrease, no change (method w/ reference gene); Methods: qPCR, sqPCR, Northern blot[f]	Gene expression: When it reaches peak and peak's magnitude (fold change; times or ratio; unclear = ?)[f]	Protein expression: Increase, decrease, no change (method w/ reference); Methods: ELISA, WB, RIA, EMSA, IF[f]	Protein expression: When it reaches peak and peak's magnitude (times or ratio; unclear = ?)[f]
Lee et al. (2012)	TNF-α	*TNF*	hPDLF-hTERT (n.g./n.g., PM, dig, P n.g., 70% confluent)	dynamic	**0.2Hz** (12cyc/min: stretch for 2.5s followed by 2.5s of relaxation) for 3h, 6h, 12h, 24h, 48h	12%	Flexercell FX-4000 Strain Unit + 35-mm flexible-bottomed Uniflex culture plates with a centrally located rectangular portion (15.25 mm×24.18 mm) coated with type I collagen + vacuum	uniaxial	increase (sqPCR, GAPDH)	48h: 4.7 (ratio)†	n.g.	n.g.
Lee et al. (2012)	TNF-α	*TNF*	hPDLF-hTERT (n.g./n.g., PM, dig, P n.g., 70% confluent)	dynamic	**0.2Hz** (12cyc/min: stretch for 2.5s followed by 2.5s of relaxation) for 24h	3%, 6%, 12%, 15%	Flexercell FX-4000 Strain Unit + 35-mm flexible-bottomed Uniflex culture plates with a centrally located rectangular portion (15.25 mm×24.18 mm) coated with type I collagen + vacuum	uniaxial	increase (sqPCR, GAPDH)	15%: 4.1 (ratio)†	n.g.	n.g.
Lee et al. (2015)	ALP	*ALPP*	hPDL cells (n.g./n.g., n.g., n.g., P n.g., 3×10^5)	dynamic	**0.1Hz** (6cyc/min) for 48h	12%	Flexcell FX-5000 Tension Unit + BioFlex Culture Plate + vacuum	equibiaxial	increase (qPCR, β-actin)	3.8 (ratio)*	n.g.	n.g.
Lee et al. (2015)	CCL3	*CCL3*	hPDL cells (n.g./n.g., n.g., n.g., P n.g., 3×10^5)	dynamic	**0.1Hz** (6cyc/min) for 2h, 4h, 8h, 24h, 48h	12%	Flexcell FX-5000 Tension Unit + BioFlex Culture Plate + vacuum	equibiaxial	increase (qPCR, β-actin)	48h: 2.8 (ratio)†	n.g.	n.g.
Lee et al. (2015)	CCL5	*CCL5*	hPDL cells (n.g./n.g., n.g., n.g., P n.g., 3×10^5)	dynamic	**0.1Hz** (6cyc/min) for 2h, 4h, 8h, 24h, 48h	12%	Flexcell FX-5000 Tension Unit + BioFlex Culture Plate + vacuum	equibiaxial	increase (qPCR, β-actin)	48h: 7.9 (ratio)†	n.g.	n.g.
Lee et al. (2015)	CCR5	*CCR5*	hPDL cells (n.g./n.g., n.g., n.g., P n.g., 3×10^5)	dynamic	**0.1Hz** (6cyc/min) qPCR for 2h, 4h, 8h, 24h, 48h; WB for 1d, 2d, 3d, 4d	12%	Flexcell FX-5000 Tension Unit + BioFlex Culture Plate + vacuum	equibiaxial	increase (qPCR, β-actin)	48h: 11.3 (ratio)†	temporary increase (WB, β-actin)	no quantitative information is given
Lee et al. (2015)	Colα1 (collagen α1)	*COL1A1*	hPDL cells (n.g./n.g., n.g., n.g., P n.g., 3×10^5)	dynamic	**0.1Hz** (6cyc/min) for 48h	12%	Flexcell FX-5000 Tension Unit + BioFlex Culture Plate + vacuum	equibiaxial	increase (qPCR, β-actin)	4.1 (ratio)*	n.g.	n.g.
Lee et al. (2015)	IL-12	*IL12A*	hPDL cells (n.g./n.g., n.g., n.g., P n.g., 3×10^5)	dynamic	**0.1Hz** (6cyc/min) for 48h	12%	Flexcell FX-5000 Tension Unit + BioFlex Culture Plate + vacuum	equibiaxial	increase (qPCR, β-actin)	8.4 (ratio)*	n.g.	n.g.
Lee et al. (2015)	OCN	*BGLAP*	hPDL cells (n.g./n.g., n.g., n.g., P n.g., 3×10^5)	dynamic	**0.1Hz** (6cyc/min) for 48h	12%	Flexcell FX-5000 Tension Unit + BioFlex Culture Plate + vacuum	equibiaxial	increase (qPCR, β-actin)	4.2 (FC?)† / 4.7 (ratio-calc)	n.g.	n.g.
Lee et al. (2015)	OPG	*TNFRSF11B*	hPDL cells (n.g./n.g., n.g., n.g., P n.g., 3×10^5)	dynamic	**0.1Hz** (6cyc/min) for 48h	12%	Flexcell FX-5000 Tension Unit + BioFlex Culture Plate + vacuum	equibiaxial	increase (qPCR, β-actin)	7.7 (ratio)*	n.g.	n.g.
Lee et al. (2015)	Periostin	*POSTN*	hPDL cells (n.g./n.g., n.g., n.g., P n.g., 3×10^5)	dynamic	**0.1Hz** (6cyc/min) for 48h	12%	Flexcell FX-5000 Tension Unit + BioFlex Culture Plate + vacuum	equibiaxial	increase (qPCR, β-actin)	8.8 (ratio)*	n.g.	n.g.
Lee et al. (2015)	RANKL	*TNFSF11*	hPDL cells (n.g./n.g., n.g., n.g., P n.g., 3×10^5)	dynamic	**0.1Hz** (6cyc/min) for 48h	12%	Flexcell FX-5000 Tension Unit + BioFlex Culture Plate + vacuum	equibiaxial	increase (qPCR, β-actin)	1.9 (rel)* / 1.2 (ratio-calc)	n.g.	n.g.
Lee et al. (2015)	Runx2	*RUNX2*	hPDL cells (n.g./n.g., n.g., n.g., P n.g., 3×10^5)	dynamic	**0.1Hz** (6cyc/min) for 48h	12%	Flexcell FX-5000 Tension Unit + BioFlex Culture Plate + vacuum	equibiaxial	increase (qPCR, β-actin)	3.8 (ratio)*	n.g.	n.g.
Li et al. (2013)	ERK1/2 / p-ERK1/2	MAPK3; MAPK1	hPDL cells (12-20/n.g., PM, exp, P3-6, 80% confluence)	dynamic	0.5Hz (30cyc/min) for 12h, 24h, 48h	10%	Flexcell FX-5000 Tension Unit + flexible-bottomed BioFlex Culture Plates coated with type I collagen + vacuum	equibiaxial	n.g.	n.g.	p-ERK1/2: increase (WB, ERK)	p-ERK1/2 @ 24h: 3.2 (rel)† / 13.7 (ratio-calc)
Li et al. (2013)	ERK5 / p-ERK5	*MAPK7*	hPDL cells (12-20/n.g., PM, exp, P3-6, 80% confluence)	dynamic	0.5Hz (30cyc/min) for 12h, 24h, 48h	10%	Flexcell FX-5000 Tension Unit + flexible-bottomed BioFlex Culture Plates coated with type I collagen + vacuum	equibiaxial	n.g.	n.g.	p-ERK5: no change (WB, ERK5)	
Li et al. (2013)	JNK / p-JNK	*MAPK8*	hPDL cells (12-20/n.g., PM, exp, P3-6, 80% confluence)	dynamic	0.5Hz (30cyc/min) for 12h, 24h, 48h	10%	Flexcell FX-5000 Tension Unit + flexible-bottomed BioFlex Culture Plates coated with type I collagen + vacuum	equibiaxial	n.g.	n.g.	p-JNK: no change (WB, JNK)	
Li et al. (2013)	P38 / p-P38	*MAPK14*	hPDL cells (12-20/n.g., PM, exp, P3-6, 80% confluence)	dynamic	0.5Hz (30cyc/min) for 12h, 24h, 48h	10%	Flexcell FX-5000 Tension Unit + flexible-bottomed BioFlex Culture Plates coated with type I collagen + vacuum	equibiaxial	n.g.	n.g.	p-P38: no change (WB, P38)	
Li et al. (2013)	RUNX2	*RUNX2*	hPDL cells (12-20/n.g., PM, exp, P3-6, 80% confluence)	dynamic	0.5Hz (30cyc/min) for 12h, 24h, 48h	10%	Flexcell FX-5000 Tension Unit + flexible-bottomed BioFlex Culture Plates coated with type I collagen + vacuum	equibiaxial	increase followed by decrease (qPCR, GAPDH)	highest @ 24h: 3.2 (FC)*<br>lowest @ 48h: 0.2 (FC)*	increase followed by decrease (WB, GAPDH)	hightest @ 24h: 0.8 (rel)* / 2.0 (ratio-calc)<br>lowest @ 48h: 0.1 (rel)* / 0.4 (ratio-calc)

[a] Entry given as reported in the study.
[b] All official gene symbols come from the HUGO Gene Nomenclature Committee (HGNC; URL: https://www.genenames.org) after checking specificity of primers with Primer-BLAST.
[c] Gender/Sex of donors: "M" – male, "F" – female; Tooth type: "PM" – premolar, "M" – molar; Cell density: given in cells/well if not otherwise mentioned.
[d] Frequencies labeled bold orange were converted to hertz (Hz) according to its definition using the information reported in the study (in brackets)
[e] Force type deduced from the description of the force apparatus given by the authors.
[f] Gene and protein expression: 1. conclusion of change (increase, decrease…) was given according to the defined criteria in Figure 2; 2. different markers to describe the amount of change; † Information derived from figures using Engauge Digitizer; *Folds calculated by measuring the graphs, without using the Engauge Digitizer; No makers: Information derived from figures by description in the articles

Reference	Gene/ Analyte[a]	Official gene symbol / abbreviation[b]	Cell (age/gender of donors, tooth type, isolation method, passages used, cell density)[a,c]	Force type (stat./ dyn.)[a]	Force duration and frequency[d]	Force magnitude[a]	Force apparatus[a]	Force type: equibiaxial or uniaxial[e]	Gene expression: Increase, decrease, no change (method w/ reference gene); Methods: qPCR, sqPCR, Northern blot[f]	Gene expression: When it reaches peak and peak's magnitude (fold change; times or ratio; unclear = ?)[f]	Protein expression: Increase, decrease, no change (method w/ reference); Methods: ELISA, WB, RIA, EMSA, IF[f]	Protein expression: When it reaches peak and peak's magnitude (times or ratio; unclear = ?)[f]
Li et al. (2013)	SP7	*SP7*	hPDL cells (12-20/n.g., PM, exp, P3-6, 80% confluence)	dynamic	0.5Hz (30cyc/min) for 12h, 24h, 48h	10%	Flexcell FX-5000 Tension Unit + flexible-bottomed BioFlex Culture Plates coated with type I collagen + vacuum	equibiaxial	decrease (qPCR, GAPDH)	48h: 0.1 (FC)*	temporary increase (WB, GAPDH)	24h: 0.5 (rel)* / 1.3 (ratio-calc)
Li et al. (2013)	SPP1	*SPP1*	hPDL cells (12-20/n.g., PM, exp, P3-6, 80% confluence)	dynamic	0.5Hz (30cyc/min) for 12h, 24h, 48h	10%	Flexcell FX-5000 Tension Unit + flexible-bottomed BioFlex Culture Plates coated with type I collagen + vacuum	equibiaxial	increase (qPCR, GAPDH)	24h: 3.4 (FC)*	temporary increase (WB, GAPDH)	24h: 0.3 (rel)* / 2.8 (ratio-calc)
Li et al. (2014)	ERK1/2 / p-ERK1/2	MAPK3; MAPK1	hPDL cells (12-16/F, 12-16/M, PM, exp, P3-6, 80% confluence)	dynamic	0.5Hz (30cyc/min) for 24h	10%	Flexcell FX-5000 Tension Unit + six-well culture plates coated with type I collagen + vacuum	equibiaxial	n.g.	n.g.	p-ERK1/2: increase (WB, ERK)	p-ERK1/2: 0.5 (rel)* / 8.0 (ratio-calc)
Li et al. (2014)	HIF-1α	*HIF1A*	hPDL cells (12-16/F, 12-16/M, PM, exp, P3-6, 80% confluence)	dynamic	0.5Hz (30cyc/min) for 0h, 12h, 24h, 48h	10%	Flexcell FX-5000 Tension Unit + six-well culture plates coated with type I collagen + vacuum	equibiaxial	increase (qPCR, GAPDH)	24h: 2.4 (ratio)*	increase (WB, GAPDH)	24h: 1.1 (rel)* / 1.9 (ratio-calc)
Li et al. (2014)	JNK / p-JNK	*MAPK8*	hPDL cells (12-16/F, 12-16/M, PM, exp, P3-6, 80% confluence)	dynamic	0.5Hz (30cyc/min) for 24h	10%	Flexcell FX-5000 Tension Unit + six-well culture plates coated with type I collagen + vacuum	equibiaxial	n.g.	n.g.	p-JNK: increase (WB, JNK)	p-JNK: 0.6 (rel)* / 1.1 (ratio-calc)
Li et al. (2014)	P38 / p-P38	*MAPK14*	hPDL cells (12-16/F, 12-16/M, PM, exp, P3-6, 80% confluence)	dynamic	0.5Hz (30cyc/min) for 24h	10%	Flexcell FX-5000 Tension Unit + six-well culture plates coated with type I collagen + vacuum	equibiaxial	n.g.	n.g.	increase (WB, p38)	0.8 (rel)* / 1.4 (ratio-calc)
Li et al. (2014)	RUNX2	*RUNX2*	hPDL cells (12-16/F, 12-16/M, PM, exp, P3-6, 80% confluence)	dynamic	0.5Hz (30cyc/min) for 24h	10%	Flexcell FX-5000 Tension Unit + six-well culture plates coated with type I collagen + vacuum	equibiaxial	increase (qPCR, GAPDH)	3.3 (rel)* / 6.6 (ratio-calc)*	increase (WB, GAPDH)	0.1 (rel)* / 2.7 (ratio-calc)
Li et al. (2014)	SP7	*SP7*	hPDL cells (12-16/F, 12-16/M, PM, exp, P3-6, 80% confluence)	dynamic	0.5Hz (30cyc/min) for 24h	10%	Flexcell FX-5000 Tension Unit + six-well culture plates coated with type I collagen + vacuum	equibiaxial	increase (qPCR, GAPDH)	2.7 (rel)* / 5.7 (ratio)*	increase (WB, GAPDH)	0.05 (rel)* / 1.3 (ratio-calc)
Li et al. (2014)	SPP1	*SPP1*	hPDL cells (12-16/F, 12-16/M, PM, exp, P3-6, 80% confluence)	dynamic	0.5Hz (30cyc/min) for 24h	10%	Flexcell FX-5000 Tension Unit + six-well culture plates coated with type I collagen + vacuum	equibiaxial	increase (qPCR, GAPDH)	2.4 (rel)* / 4.8 (ratio)*	increase (WB, GAPDH)	0.02 (rel)* / 1.5 (ratio-calc)
Li et al. (2015)	Cx43	*GJA1*	hPDL cells ($) (n.g./n.g., n.g., n.g., P3-6, 75%-85% confluence)	dynamic	**0.005Hz** (3min/cyc) for 0.5h, 1h, 2h, 4h, 8h, 12h, 24h	5%	"custom-made tensile device + elastic membranes were made of polydimethyl-siloxane (PDMS) gel +motor"	uniaxial	increase (qPCR, GAPDH)	24h: 6.3 (ratio)*	increase (WB, GAPDH)	24h: 0.8 (rel)* / 2.1 (ratio-calc)
Li et al. (2015)	OPG	*TNFRSF11B*	hPDL cells ($) (n.g./n.g., n.g., n.g., P3-6, 75%-85% confluence)	dynamic	**0.005Hz** (3min/cyc) for 0.5h, 1h, 2h, 4h, 8h, 12h, 24h	5%	"custom-made tensile device + elastic membranes were made of polydimethyl-siloxane (PDMS) gel +motor"	uniaxial	increase (qPCR, GAPDH)	12h: 4.0 (ratio)*	increase (WB, GAPDH)	8h: 0.9 (rel)* / 4.4 (ratio-calc)
Li et al. (2015)	Osterix	*SP7*	hPDL cells ($) (n.g./n.g., n.g., n.g., P3-6, 75%-85% confluence)	dynamic	**0.005Hz** (3min/cyc) for 0.5h, 1h, 2h, 4h, 8h, 12h, 24h	5%	"custom-made tensile device + elastic membranes were made of polydimethyl-siloxane (PDMS) gel +motor"	uniaxial	increase (qPCR, GAPDH)	24h: 14.0 (ratio)*	increase followed by plateau (WB, GAPDH)	12h…24h: 1.6 (rel)* / 4.2 (ratio-calc)
Li et al. (2015)	RANKL	*TNFSF11*	hPDL cells ($) (n.g./n.g., n.g., n.g., P3-6, 75%-85% confluence)	dynamic	**0.005Hz** (3min/cyc) for 0.5h, 1h, 2h, 4h, 8h, 12h, 24h	5%	"custom-made tensile device + elastic membranes were made of polydimethyl-siloxane (PDMS) gel +motor"	uniaxial	increase (qPCR, GAPDH)	1h: 3.9 (ratio)*	increase (WB, GAPDH)	4h: 1.3 (rel)* / 3.3 (ratio-calc)
Li et al. (2015)	RUNX2	*RUNX2*	hPDL cells ($) (n.g./n.g., n.g., n.g., P3-6, 75%-85% confluence)	dynamic	**0.005Hz** (3min/cyc) for 0.5h, 1h, 2h, 4h, 8h, 12h, 24h	5%	"custom-made tensile device + elastic membranes were made of polydimethyl-siloxane (PDMS) gel +motor"	uniaxial	increase (qPCR, GAPDH)	24h: 12.0 (ratio)*	increase followed by plateau (WB, GAPDH)	12h…24h: 2 (rel)* / 1.4 (ratio-calc)
Liao and Hua (2013)	CSE	*SLC2A1*	hPDL cells (13-18/n.g., PM, dig, P3-8, Confluence)	static	30min, 60min, 90min, 120min	1.5%	Flexcell FX-5000 Tension System + flexible-bottomed six-well plates + vacuum	equibiaxial	increase (qPCR, GAPDH)	60min: 3.7 (ratio)*	n.g.	n.g.
Liao and Hua (2013)	OPG	*TNFRSF11B*	hPDL cells (13-18/n.g., PM, dig, P3-8, Confluence)	static	60min	1.5%	Flexcell FX-5000 Tension System + flexible-bottomed six-well plates + vacuum	equibiaxial	*OPG*: increase (qPCR, GAPDH)<br>*OPG/RANKL*: increase (qPCR, GAPDH)	1.4 (ratio)*<br>1.2 (ratio)*	OPG: increase (ELISA)<br>OPG/RANKL: increase (ELISA)	2127.7 (ng/mL)* / 1.2 (ratio-calc)<br>1.2 (rel)* / 1.7 (ratio-calc)
Liao and Hua (2013)	RANKL	*TNFSF11*	hPDL cells (13-18/n.g., PM, dig, P3-8, Confluence)	static	60min	1.5%	Flexcell FX-5000 Tension System + flexible-bottomed six-well plates + vacuum	equibiaxial	*RANKL*: increase (qPCR, GAPDH)<br>*OPG/RANKL*: increase (qPCR, GAPDH)	1.2 (ratio)*<br>1.2 (ratio)*	RANKL: decrease (ELISA)<br>OPG/RANKL: increase (ELISA)	1787.2 (ng/mL)* / 0.7 (ratio-calc)<br>1.2 (rel)* / 1.7 (ratio-calc)
Liu et al. (2012)	BGN	*BGN*	hPDL cells (12-15/n.g., PM, exp, P3-4, Confluent)	dynamic	**0.1Hz** (6cyc/min) for 24h	12%	Flexcell FX 3000 + six-well, flexible-bottomed plates + vacuum (Li et al 2010; Tang et al 2006)	equibiaxial	increase (qPCR, GAPDH)	1.9 (rel)* / 4.6 (ratio-calc)	n.g.	n.g.
Liu et al. (2012)	Col12A1	*COL12A1*	hPDL cells (12-15/n.g., PM, exp, P3-4, Confluent)	dynamic	**0.1Hz** (6cyc/min) for 24h	12%	Flexcell FX 3000 + six-well, flexible-bottomed plates + vacuum (Li et al 2010; Tang et al 2006)	equibiaxial	Increase (qPCR, GAPDH)	1.4 (rel)* / 3.9 (ratio-calc)	n.g.	n.g.

[a] Entry given as reported in the study.
[b] All official gene symbols come from the HUGO Gene Nomenclature Committee (HGNC; URL: https://www.genenames.org) after checking specificity of primers with Primer-BLAST.
[c] Gender/Sex of donors: "M" – male, "F" – female; Tooth type: "PM" – premolar, "M" – molar; Cell density: given in cells/well if not otherwise mentioned.
[d] Frequencies labeled bold orange were converted to hertz (Hz) according to its definition using the information reported in the study (in brackets)
[e] Force type deduced from the description of the force apparatus given by the authors.
[f] Gene and protein expression: 1. conclusion of change (increase, decrease…) was given according to the defined criteria in Figure 2; 2. different markers to describe the amount of change; † Information derived from figures using Engauge Digitizer; *Folds calculated by measuring the graphs, without using the Engauge Digitizer; No makers: Information derived from figures by description in the articles

Reference	Gene/ Analyte[a]	Official gene symbol / abbreviation[b]	Cell (age/gender of donors, tooth type, isolation method, passages used, cell density)[a,c]	Force type (stat./ dyn.)[a]	Force duration and frequency[d]	Force magnitude[a]	Force apparatus[a]	Force type: equibiaxial or uniaxial[e]	Gene expression: Increase, decrease, no change (method w/ reference gene); Methods: qPCR, sqPCR, Northern blot[f]	Gene expression: When it reaches peak and peak's magnitude (fold change; times or ratio; unclear = ?)[f]	Protein expression: Increase, decrease, no change (method w/ reference); Methods: ELISA, WB, RIA, EMSA, IF[f]	Protein expression: When it reaches peak and peak's magnitude (times or ratio; unclear = ?)[f]
Liu et al. (2012)	EGFR	*EGFR*	hPDL cells (12-15/n.g., PM, exp, P3-4, Confluent)	dynamic	**0.1Hz** (6cyc/min) for 24h	12%	Flexcell FX 3000 + six-well, flexible-bottomed plates + vacuum (Li et al 2010; Tang et al 2006)	equibiaxial	increase (qPCR, GAPDH)	1.8 (rel)* / 4.9 (ratio-calc)	n.g.	n.g.
Liu et al. (2012)	IGF-1	*IGF1*	hPDL cells (12-15/n.g., PM, exp, P3-4, Confluent)	dynamic	**0.1Hz** (6cyc/min) for 24h	12%	Flexcell FX 3000 + six-well, flexible-bottomed plates + vacuum (Li et al 2010; Tang et al 2006)	equibiaxial	increase (qPCR, GAPDH)	1.2 (rel)* / 6.0 (ratio-calc)	n.g.	n.g.
Liu et al. (2012)	ITGA1	*ITGA1*	hPDL cells (12-15/n.g., PM, exp, P3-4, Confluent)	dynamic	**0.1Hz** (6cyc/min) for 24h	12%	Flexcell FX 3000 + six-well, flexible-bottomed plates + vacuum (Li et al 2010; Tang et al 2006)	equibiaxial	decrease (qPCR, GAPDH)	0.6 (rel)* / 0.4 (ratio-calc)	n.g.	n.g.
Liu et al. (2012)	ITGA3	*ITGA3*	hPDL cells (12-15/n.g., PM, exp, P3-4, Confluent)	dynamic	**0.1Hz** (6cyc/min) for 24h	12%	Flexcell FX 3000 + six-well, flexible-bottomed plates + vacuum (Li et al 2010; Tang et al 2006)	equibiaxial	Increase (qPCR, GAPDH)	0.7 (rel)* / 3.3 (ratio-calc)	n.g.	n.g.
Liu et al. (2012)	MMP-2	*MMP2*	hPDL cells (12-15/n.g., PM, exp, P3-4, Confluent)	dynamic	**0.1Hz** (6cyc/min) for 24h	12%	Flexcell FX 3000 + six-well, flexible-bottomed plates + vacuum (Li et al 2010; Tang et al 2006)	equibiaxial	Increase (qPCR, GAPDH)	2.0 (rel)* / 3.6 (ratio-calc)	n.g.	n.g.
Liu et al. (2012)	MSX1	*MSX1*	hPDL cells (12-15/n.g., PM, exp, P3-4, Confluent)	dynamic	**0.1Hz** (6cyc/min) for 24h	12%	Flexcell FX 3000 + six-well, flexible-bottomed plates + vacuum (Li et al 2010; Tang et al 2006)	equibiaxial	increase (qPCR, GAPDH)	0.8 (rel)* / 4.7 (ratio-calc)	n.g.	n.g.
Liu et al. (2012)	SMAD7	*SMAD7*	hPDL cells (12-15/n.g., PM, exp, P3-4, Confluent)	dynamic	**0.1Hz** (6cyc/min) for 24h	12%	Flexcell FX 3000 + six-well, flexible-bottomed plates + vacuum (Li et al 2010; Tang et al 2006)	equibiaxial	increase (qPCR, GAPDH)	0.4 (rel)* / 2.1 (ratio-calc)	n.g.	n.g.
Liu et al. (2012)	TGFβR1	*TGFBR1*	hPDL cells (12-15/n.g., PM, exp, P3-4, Confluent)	dynamic	**0.1Hz** (6cyc/min) for 24h	12%	Flexcell FX 3000 + six-well, flexible-bottomed plates + vacuum (Li et al 2010; Tang et al 2006)	equibiaxial	decrease (qPCR, GAPDH)	0.4 (rel)* / 0.5 (ratio-calc)	n.g.	n.g.
Liu et al. (2017)	ALP	*ALPP*	hPDLSCs cells (37.9±7.2/n.g., PM and M, dig, P3, 95% confluence) from healthy (HPDLSCs) and patients w/ periodontitis (PPDLSCs: 38.9 ± 7.9/n.g., n.g., dig, P3, 95% confluence)	dynamic	0.1Hz for 12h	6%, 8%, 10%, 12%, 14%	Flexcell FX-4000T + 6-well Bioflex plates + vacuum	equibiaxial	HPDLSCs: increase (qPCR, β-actin)	HPDLSCs @ 12%: 1.6 (ratio)*	n.g.	n.g.
Liu et al. (2017)	C-fos	*FOS*	hPDLSCs cells (37.9±7.2/n.g., PM and M, dig, P3, 95% confluence) from healthy (HPDLSCs) and patients w/ periodontitis (PPDLSCs: 38.9 ± 7.9/n.g., n.g., dig, P3, 95% confluence)	dynamic	0.1Hz for 12h	6%, 8%, 10%, 12%, 14%	Flexcell FX-4000T + 6-well Bioflex plates + vacuum	equibiaxial	HPDLSCs: increase (qPCR, β-actin)	HPDLSCs @ 14%: 1.7 (ratio)*	n.g.	n.g.
Liu et al. (2017)	IL-1β	*IL1B*	hPDLSCs cells (37.9±7.2/n.g., PM and M, dig, P3, 95% confluence) from healthy (HPDLSCs) and patients w/ periodontitis (PPDLSCs: 38.9 ± 7.9/n.g., n.g., dig, P3, 95% confluence)	dynamic	0.1Hz for 12h	6%, 8%, 10%, 12%, 14%	Flexcell FX-4000T + 6-well Bioflex plates + vacuum	equibiaxial	n.g.	n.g.	HPDLSCs: increase followed by plateau (ELISA)	HPDLSCs @ 6%...14%: 3.2 (pg/10^6 cells)* / 1.5 (ratio)*
Liu et al. (2017)	IL-6	*IL6*	hPDLSCs cells (37.9±7.2/n.g., PM and M, dig, P3, 95% confluence) from healthy (HPDLSCs) and patients w/ periodontitis (PPDLSCs: 38.9 ± 7.9/n.g., n.g., dig, P3, 95% confluence)	dynamic	0.1Hz for 12h	6%, 8%, 10%, 12%, 14%	Flexcell FX-4000T + 6-well Bioflex plates + vacuum	equibiaxial	n.g.	n.g.	HPDLSCs: increase (ELISA)	HPDLSCs @ 14%: 585.7 (pg/10^6 cells)* / 11.7 (ratio)*
Liu et al. (2017)	IL-8	*CXCL8*	hPDLSCs cells (37.9±7.2/n.g., PM and M, dig, P3, 95% confluence) from healthy (HPDLSCs) and patients w/ periodontitis (PPDLSCs: 38.9 ± 7.9/n.g., n.g., dig, P3, 95% confluence)	dynamic	0.1Hz for 12h	6%, 8%, 10%, 12%, 14%	Flexcell FX-4000T + 6-well Bioflex plates + vacuum	equibiaxial	n.g.	n.g.	HPDLSCs: increase (ELISA)	HPDLSCs @ 14%: 466.7 (pg/10^6 cells)* / 128.2 (ratio)*
Liu et al. (2017)	OPG	*TNFRSF11B*	hPDLSCs cells (37.9±7.2/n.g., PM and M, dig, P3, 95% confluence) from healthy (HPDLSCs) and patients w/ periodontitis (PPDLSCs: 38.9 ± 7.9/n.g., n.g., dig, P3, 95% confluence)	dynamic	0.1Hz for 12h	6%, 8%, 10%, 12%, 14%	Flexcell FX-4000T + 6-well Bioflex plates + vacuum	equibiaxial	HPDLSCs: increase (qPCR, β-actin)	HPDLSCs @ 12%: 1.6 (ratio)*	n.g.	n.g.
Liu et al. (2017)	RANKL	*TNFSF11*	hPDLSCs cells (37.9±7.2/n.g., PM and M, dig, P3, 95% confluence) from healthy (HPDLSCs) and patients w/ periodontitis (PPDLSCs: 38.9 ± 7.9/n.g., n.g., dig, P3, 95% confluence)	dynamic	0.1Hz for 12h	6%, 8%, 10%, 12%, 14%	Flexcell FX-4000T + 6-well Bioflex plates + vacuum	equibiaxial	HPDLSCs: increase (qPCR, β-actin)	HPDLSCs @ 14%: 1.8 (ratio)*	n.g.	n.g.
Liu et al. (2017)	RUNX2	*RUNX2*	hPDLSCs cells (37.9±7.2/n.g., PM and M, dig, P3, 95% confluence) from healthy (HPDLSCs) and patients w/ periodontitis (PPDLSCs: 38.9 ± 7.9/n.g., n.g., dig, P3, 95% confluence)	dynamic	0.1Hz for 12h	6%, 8%, 10%, 12%, 14%	Flexcell FX-4000T + 6-well Bioflex plates + vacuum	equibiaxial	HPDLSCs: increase (qPCR, β-actin)	HPDLSCs @ 12%: 3.7 (ratio)*	n.g.	n.g.

[a] Entry given as reported in the study.
[b] All official gene symbols come from the HUGO Gene Nomenclature Committee (HGNC; URL: https://www.genenames.org) after checking specificity of primers with Primer-BLAST.
[c] Gender/Sex of donors: "M" – male, "F" – female; Tooth type: "PM" – premolar, "M" – molar; Cell density: given in cells/well if not otherwise mentioned.
[d] Frequencies labeled bold orange were converted to hertz (Hz) according to its definition using the information reported in the study (in brackets)
[e] Force type deduced from the description of the force apparatus given by the authors.
[f] Gene and protein expression: 1. conclusion of change (increase, decrease…) was given according to the defined criteria in Figure 2; 2. different markers to describe the amount of change; † Information derived from figures using Engauge Digitizer; *Folds calculated by measuring the graphs, without using the Engauge Digitizer; No makers: Information derived from figures by description in the articles

Reference	Gene/ Analyte[a]	Official gene symbol / abbreviation[b]	Cell (age/gender of donors, tooth type, isolation method, passages used, cell density)[a,c]	Force type (stat./ dyn.)[a]	Force duration and frequency[d]	Force magnitude[a]	Force apparatus[a]	Force type: equibiaxial or uniaxial[e]	Gene expression: Increase, decrease, no change (method w/ reference gene); Methods: qPCR, sqPCR, Northern blot[f]	Gene expression: When it reaches peak and peak's magnitude (fold change; times or ratio; unclear = ?)[f]	Protein expression: Increase, decrease, no change (method w/ reference); Methods: ELISA, WB, RIA, EMSA, IF[f]	Protein expression: When it reaches peak and peak's magnitude (times or ratio; unclear = ?)[f]
Liu et al. (2017)	TNF-α	*TNF*	hPDLSCs cells (37.9±7.2/n.g., PM and M, dig, P3, 95% confluence) from healthy (HPDLSCs) and patients w/ periodontitis (PPDLSCs: 38.9 ± 7.9/n.g., n.g., dig, P3, 95% confluence)	dynamic	0.1Hz for 12h	6%, 8%, 10%, 12%, 14%	Flexcell FX-4000T + 6-well Bioflex plates + vacuum	equibiaxial	n.g.	n.g.	HPDLSCs: increase followed by plateau (ELISA)	HPDLSCs @ 6%...14%: 18.6 (pg/10^6 cells)* / 1.2 (ratio)*
Long et al. (2001)	IL-10	*IL10*	hPDL cells ($) (18/F, 16/F, 22/M, 16/M, M, n.g., P6-20, 5×10^5)	dynamic	0.005Hz sqPCR for 4h, 24h, 48h; ELISA for 24h, 48h	6%	Flexercell unit + pronectin-coated six-well Flexercell plates + vacuum (Gassner et al 1999)	equibiaxial	increase (sqPCR, GAPDH)	24h: 781.3 (rel)* / not detectable in the control	increase (ELISA)	48h: 62.2 (pg/ml)* / control not detectable
Long et al. (2001)	IL-1β	*IL1B*	hPDL cells ($) (18/F, 16/F, 22/M, 16/M, M, n.g., P6-20, 5×10^5)	dynamic	0.005Hz for 24h	sqPCR: 3%, 6%, 10%, 15%; ELISA: 6%, 10%, 15%	Flexercell unit + pronectin-coated six-well Flexercell plates + vacuum (Gassner et al 1999)	equibiaxial	decrease followed by plateau then increase (sqPCR, GAPDH)	lowest @ 6%…10%: 11.4 (rel) / 0.1 (ratio-calc)<br>highest @15%: 152.3 (rel) / 1.5 (ratio-calc)	increase (ELISA)	15%: 85.7 (pg/ml)* / control n.g
Long et al. (2001)	IL-1β	*IL1B*	hPDL cells ($) (18/F, 16/F, 22/M, 16/M, M, n.g., P6-20, 5×10^5)	dynamic	0.005Hz sqPCR for 4h, 24h, 48h; ELISA for 24h, 48h	6%	Flexercell unit + pronectin-coated six-well Flexercell plates + vacuum (Gassner et al 1999)	equibiaxial	no expression (sqPCR, GAPDH)	no quantitative information is given	no expression (ELISA)	no quantitative information is given
Long et al. (2001)	IL-6	*IL6*	hPDL cells ($) (18/F, 16/F, 22/M, 16/M, M, n.g., P6-20, 5×10^5)	dynamic	0.005Hz sqPCR for 4h, 24h, 48h; ELISA for 24h, 48h	6%	Flexercell unit + pronectin-coated six-well Flexercell plates + vacuum (Gassner et al 1999)	equibiaxial	no expression (sqPCR, GAPDH)	no quantitative information is given	no expression (ELISA)	no quantitative information is given
Long et al. (2001)	IL-8	*CXCL8*	hPDL cells ($) (18/F, 16/F, 22/M, 16/M, M, n.g., P6-20, 5×10^5)	dynamic	0.005Hz sqPCR for 4h, 24h, 48h; ELISA for 24h, 48h	6%	Flexercell unit + pronectin-coated six-well Flexercell plates + vacuum (Gassner et al 1999)	equibiaxial	no expression (sqPCR, GAPDH)	no quantitative information is given	no expression (ELISA)	no quantitative information is given
Long et al. (2001)	TNF-α	*TNF*	hPDL cells ($) (18/F, 16/F, 22/M, 16/M, M, n.g., P6-20, 5×10^5)	dynamic	0.005Hz sqPCR for 4h, 24h, 48h; ELISA for 24h, 48h	6%	Flexercell unit + pronectin-coated six-well Flexercell plates + vacuum (Gassner et al 1999)	equibiaxial	no expression (sqPCR, GAPDH)	no quantitative information is given	n.g.	n.g.
Long et al. (2002)	COX-2	*PTGS2*	hPDL cells (18/F, 16/F, 18/M, 16/M, M, exp, P3-6, Confluent)	dynamic	0.005Hz for 4h, 24h, 48h	6%	Flexercell unit + collagen type 1-coated Bioflex II plates + vacuum	equibiaxial	decrease (sqPCR, GAPDH)	no quantitative information is given	n.g.	n.g.
Long et al. (2002)	iNOS	*NOS2*	hPDL cells (18/F, 16/F, 18/M, 16/M, M, exp, P3-6, Confluent)	dynamic	0.005Hz for 4h, 24h, 48h	6%	Flexercell unit + collagen type 1-coated Bioflex II plates + vacuum	equibiaxial	no change (sqPCR, GAPDH)	no quantitative information is given	n.g.	n.g.
Long et al. (2002)	MMP-1	*MMP1*	hPDL cells (18/F, 16/F, 18/M, 16/M, M, exp, P3-6, Confluent)	dynamic	0.005Hz for 4h, 24h, 48h	6%	Flexercell unit + collagen type 1-coated Bioflex II plates + vacuum	equibiaxial	no change (sqPCR, GAPDH)	no quantitative information is given	no expression (WB)	
Long et al. (2002)	MMP-3	*MMP3*	hPDL cells (18/F, 16/F, 18/M, 16/M, M, exp, P3-6, Confluent)	dynamic	0.005Hz for 4h, 24h, 48h	6%	Flexercell unit + collagen type 1-coated Bioflex II plates + vacuum	equibiaxial	no change (sqPCR, GAPDH)	no quantitative information is given	no expression (WB)	
Long et al. (2002)	PGE_2	PGE_2	hPDL cells (18/F, 16/F, 18/M, 16/M, M, exp, P3-6, Confluent)	dynamic	0.005Hz for 24h	1.8%, 3%, 6%, 10%, 12.5%	Flexercell unit + collagen type 1-coated Bioflex II plates + vacuum	equibiaxial	n.a.	n.a.	1.8%, 3%, 6%: no expression (RIA)<br>10%, 12.5%: increase (RIA)	12.5%: 102.8 (ng /10^6 cells) / control: no PGE_2 detectable
Long et al. (2002)	TIMP-II	*TIMP2*	hPDL cells (18/F, 16/F, 18/M, 16/M, M, exp, P3-6, Confluent)	dynamic	0.005Hz sqPCR for 4h, 24h; WB for 24h, 48h	6%	Flexercell unit + collagen type 1-coated Bioflex II plates + vacuum	equibiaxial	no change (sqPCR, GAPDH)	no quantitative information is given	no change (WB)	
Ma et al. (2015)	ARRAY	ARRAY	hPDL cells (11/F, PM, exp, P4-6, confluence)	dynamic	**0.1Hz** (6cyc/min: 5s stretch followed by 5s relaxation) for 6h, 24h	10%	Cell Strain Unit (CSU + flexible-bottomed culture dish made of elastic silicon rubber (Q7-4750, Dow Corning Co., Midland, MI, USA) + spherical cap	equibiaxial	Human Extracellular Matrix & Adhesion Molecules RT2 Profiler PCR Array (PAHS-013, SABiosciences)			
Ma et al. (2015)	Integrin α 5	*ITGAV*	hPDL cells (11/F, PM, exp, P4-6, confluence)	dynamic	**0.1Hz** (6cyc/min: 5s stretch followed by 5s relaxation) for 6h, 24h	10%	Cell Strain Unit (CSU + flexible-bottomed culture dish made of elastic silicon rubber (Q7-4750, Dow Corning Co., Midland, MI, USA) + spherical cap	equibiaxial	n.g.	n.g.	increase (WB, GAPDH)	24h: 1.1 (rel)* / 1.3 (ratio-calc)
Matsuda et al. (1998a)	ERK1/2 / p-ERK$^{(Tyr204)}$	MAPK3; MAPK1	hPDL cells (n.g./n.g., n.g., exp, P3-5, confluence)	dynamic	**0.1Hz** (6cyc/min: strain for 5s and then 5s relaxation) for 15min, 30min, 60min	9%	Flexercell Strain Unit Model FX-2000 (Banes et al 1985) + flexible substratum (25 mm dia., Flex I culture plate) + vacuum	equibiaxial	n.g.	n.g.	no change (WB)	no quantitative information is given
Matsuda et al. (1998a)	JNK / p-JNK$^{(Thr183/Tyr185)}$	*MAPK8*	hPDL cells (n.g./n.g., n.g., exp, P3-5, confluence)	dynamic	**0.1Hz** (6cyc/min: strain for 5s and then 5s relaxation) for 15min, 30min, 60min	9%	Flexercell Strain Unit Model FX-2000 (Banes et al 1985) + flexible substratum (25 mm dia., Flex I culture plate) + vacuum	equibiaxial	n.g.	n.g.	increase (WB)	no quantitative information is given

[a] Entry given as reported in the study.
[b] All official gene symbols come from the HUGO Gene Nomenclature Committee (HGNC; URL: https://www.genenames.org) after checking specificity of primers with Primer-BLAST.
[c] Gender/Sex of donors: "M" – male, "F" – female; Tooth type: "PM" – premolar, "M" – molar; Cell density: given in cells/well if not otherwise mentioned.
[d] Frequencies labeled bold orange were converted to hertz (Hz) according to its definition using the information reported in the study (in brackets)
[e] Force type deduced from the description of the force apparatus given by the authors.
[f] Gene and protein expression: 1. conclusion of change (increase, decrease…) was given according to the defined criteria in Figure 2; 2. different markers to describe the amount of change; † Information derived from figures using Engauge Digitizer; *Folds calculated by measuring the graphs, without using the Engauge Digitizer; No makers: Information derived from figures by description in the articles

Reference	Gene/ Analyte[a]	Official gene symbol / abbreviation[b]	Cell (age/gender of donors, tooth type, isolation method, passages used, cell density)[a,c]	Force type (stat./ dyn.)[a]	Force duration and frequency[d]	Force magnitude[a]	Force apparatus[a]	Force type: equibiaxial or uniaxial[e]	Gene expression: Increase, decrease, no change (method w/ reference gene); Methods: qPCR, sqPCR, Northern blot[f]	Gene expression: When it reaches peak and peak's magnitude (fold change; times or ratio; unclear = ?)[f]	Protein expression: Increase, decrease, no change (method w/ reference); Methods: ELISA, WB, RIA, EMSA, IF[f]	Protein expression: When it reaches peak and peak's magnitude (times or ratio; unclear = ?)[f]
Matsuda et al. (1998a)	p38 / p-p38$^{(Thr180/Tyr182)}$	*MAPK14*	hPDL cells (n.g./n.g., n.g., exp, P3-5, confluence)	dynamic	**0.1Hz** (6cyc/min: strain for 5s and then 5s relaxation) for 15min, 30min, 60min	9%	Flexercell Strain Unit Model FX-2000 (Banes et al 1985) + flexible substratum (25 mm dia., Flex I culture plate) + vacuum	equibiaxial	n.g.	n.g.	no change (WB)	no quantitative information is given
Matsuda et al. (1998b)	ALP	*ALPP*	hPDL cells (n.g./n.g., M, exp, P3-7, n.g.)	dynamic	**0.1Hz** (6cyc/min: strain for 5s followed by 5s relaxation) for 2d, 4d, 6d	9%,18%	Flexercell Strain Unit Model FX-2000 (Banes et al 1985) + flexible substratum (25 mm dia., Flex I culture plate) + vacuum	equibiaxial	n.g.	n.g.	9%: increase (ALP activity)<br>18%: increase (ALP activity)	9% @ 6d: 257.7 (U/mg protein)* / 1.4 (ratio-calc)<br>18% @ 4…6d: 230.8 (U/mg protein)* / 1.3 (ratio-calc)
Matsuda et al. (1998b)	EGF-R	*EGFR*	hPDL cells (n.g./n.g., M, exp, P3-7, n.g.)	dynamic	**0.1Hz** (6cyc/min: strain for 5s followed by 5s relaxation) for 4d	9%	Flexercell Strain Unit Model FX-2000 (Banes et al 1985) + flexible substratum (25 mm dia., Flex I culture plate) + vacuum	equibiaxial	n.g.	n.g.	decrease (WB)	0.4 (ratio)
Memmert et al. (2019)	ARRAY	ARRAY	hPDLCs (11-19/n.g., n.g., exp, P3-5, 80% confluence)	static	24h	3%, 20%	CESTRA cell strain device + BioFlex-II-culture plates + stepping motor (Deschner et al. 2012)	equibiaxial	PrimePCR™ Assay (Autophagy (SAB Target List) H96, Bio-Rad Laboratories, Munich, Germany)		n.g.	n.g.
Memmert et al. (2019)	ATG10	*ATG10*	hPDLCs (11-19/n.g., n.g., exp, P3-5, 80% confluence)	static	4h, 24h	3%, 20%	CESTRA cell strain device + BioFlex-II-culture plates + stepping motor (Deschner et al. 2012)	equibiaxial	3%: temporary decrease (qPCR, GAPDH)<br>20%: decrease (qPCR, GAPDH)	3% @ 4h: 0.9 (FC)†<br>20% @ 24h: 0.3 (FC)†	n.g.	n.g.
Memmert et al. (2019)	ATG4C	*ATG4C*	hPDLCs (11-19/n.g., n.g., exp, P3-5, 80% confluence)	static	4h, 24h	3%, 20%	CESTRA cell strain device + BioFlex-II-culture plates + stepping motor (Deschner et al. 2012)	equibiaxial	3%: no change (qPCR, GAPDH)<br>20%: decrease (qPCR, GAPDH)	20% @ 24h: 0.4 (FC)†	n.g.	n.g.
Memmert et al. (2019)	ATG7	*ATG7*	hPDLCs (11-19/n.g., n.g., exp, P3-5, 80% confluence)	static	4h, 24h	3%, 20%	CESTRA cell strain device + BioFlex-II-culture plates + stepping motor (Deschner et al. 2012)	equibiaxial	3%: temporary decrease (qPCR, GAPDH)<br>20%: decrease (qPCR, GAPDH)	3% @ 4h: 0.8 (FC)†<br>20% @ 24h: 0.4 (FC)†	n.g.	n.g.
Memmert et al. (2019)	BCL2	*BCL2*	hPDLCs (11-19/n.g., n.g., exp, P3-5, 80% confluence)	static	4h, 24h	3%, 20%	CESTRA cell strain device + BioFlex-II-culture plates + stepping motor (Deschner et al. 2012)	equibiaxial	3%: temporary decrease (qPCR, GAPDH)<br>20%: decrease (qPCR, GAPDH)	3% @ 4h: 0.7 (FC)†<br>20% @ 24h: 0.7 (FC)†	n.g.	n.g.
Memmert et al. (2019)	BID	*BID*	hPDLCs (11-19/n.g., n.g., exp, P3-5, 80% confluence)	static	4h, 24h	3%, 20%	CESTRA cell strain device + BioFlex-II-culture plates + stepping motor (Deschner et al. 2012)	equibiaxial	3%: decrease followed by increase (qPCR, GAPDH)<br>20%: no change (qPCR, GAPDH)	3% lowest @ 4h: 0.9 (FC)†<br>3% highest @ 24h: 1.9 (FC)†	n.g.	n.g.
Memmert et al. (2019)	DAPK1	*DAPK1*	hPDLCs (11-19/n.g., n.g., exp, P3-5, 80% confluence)	static	4h, 24h	3%, 20%	CESTRA cell strain device + BioFlex-II-culture plates + stepping motor (Deschner et al. 2012)	equibiaxial	3%: decrease (qPCR, GAPDH)<br>20%: decrease (qPCR, GAPDH)	3% @ 24h: 0.7 (FC)†<br>20% @ 24h: 0.3 (FC)†	n.g.	n.g.
Memmert et al. (2019)	LC3-I	*Map1lc3a*	hPDLCs (11-19/n.g., n.g., exp, P3-5, 80% confluence)	static	4h	20%	CESTRA cell strain device + BioFlex-II-culture plates + stepping motor (Deschner et al. 2012)	equibiaxial	n.g.	n.g.	increase (WB, GAPDH)	no quantitative information is given
Memmert et al. (2019)	LC3-II	*Map1lc3a*	hPDLCs (11-19/n.g., n.g., exp, P3-5, 80% confluence)	static	4h	20%	CESTRA cell strain device + BioFlex-II-culture plates + stepping motor (Deschner et al. 2012)	equibiaxial	n.g.	n.g.	increase (WB, GAPDH)	1.4 (ratio)†
Memmert et al. (2019)	PIK3CG	*PIK3CG*	hPDLCs (11-19/n.g., n.g., exp, P3-5, 80% confluence)	static	4h, 24h	3%, 20%	CESTRA cell strain device + BioFlex-II-culture plates + stepping motor (Deschner et al. 2012)	equibiaxial	3%: no change (qPCR, GAPDH)<br>20%: no change (qPCR, GAPDH)		n.g.	n.g.
Memmert et al. (2019)	SNCA	*SNCA*	hPDLCs (11-19/n.g., n.g., exp, P3-5, 80% confluence)	static	4h, 24h	3%, 20%	CESTRA cell strain device + BioFlex-II-culture plates + stepping motor (Deschner et al. 2012)	equibiaxial	3%: increase (qPCR, GAPDH)<br>20%: decrease (qPCR, GAPDH)	3% @ 24h: 1.2 (FC)†<br>20% @ 24h: 0.3 (FC)†	n.g.	n.g.
Memmert et al. (2019)	TP53	*TP53*	hPDLCs (11-19/n.g., n.g., exp, P3-5, 80% confluence)	static	4h, 24h	3%, 20%	CESTRA cell strain device + BioFlex-II-culture plates + stepping motor (Deschner et al. 2012)	equibiaxial	3%: increase (qPCR, GAPDH)<br>20%: temporary increase (qPCR, GAPDH)	3% @ 4h: 2.1 (FC)†<br>20% @ 4h: 4.2 (FC)†	n.g.	n.g.
Memmert et al. (2019)	UVRAG	*UVRAG*	hPDLCs (11-19/n.g., n.g., exp, P3-5, 80% confluence)	static	4h, 24h	3%, 20%	CESTRA cell strain device + BioFlex-II-culture plates + stepping motor (Deschner et al. 2012)	equibiaxial	3%: temporary decrease (qPCR, GAPDH)<br>20%: decrease (qPCR, GAPDH)	3% @ 4h: 0.7(FC)†<br>20% @ 24h: 0.5 (FC)†	n.g.	n.g.
Memmert et al. (2020)	ARRAY	ARRAY	hPDLFs (11-19/n.g., n.g., exp, P3-5, 80% confluency)	dynamic	0.1Hz for 24h	3%	CESTRA cell strain device + 6-well BioFlex plates coated with collagen type I + stepping motor	equibiaxial	PrimePCR assay (Autophagy [SAB Target List] H96,Bio-Rad Laboratories, Munich, Germany)		n.g.	n.g.
Memmert et al. (2020)	SQSTM1	*SQSTM1*	hPDLFs (11-19/n.g., n.g., exp, P3-5, 80% confluency)	static	qPCR for 8h, 16h, 24h; WB for 1h	3%	CESTRA cell strain device + 6-well BioFlex plates coated with collagen type I + stepping motor	equibiaxial	increase (qPCR, GAPDH)	16h: 1.5 (FC)†	no change (WB, GAPDH)	
Memmert et al. (2020)	SQSTM1	*SQSTM1*	hPDLFs (11-19/n.g., n.g., exp, P3-5, 80% confluency)	static	qPCR for 8h, 16h, 24h; WB for 1h	20%	CESTRA cell strain device + 6-well BioFlex plates coated with collagen type I + stepping motor	equibiaxial	decrease followed by increase (qPCR, GAPDH)	lowest @ 8h: 0.6 (FC)†<br>highest @ 16h: 1.8 (FC)†	increase (WB, GAPDH)	1.7 (ratio)†
Memmert et al. (2020)	SQSTM1	*SQSTM1*	hPDLFs (11-19/n.g., n.g., exp, P3-5, 80% confluency)	dynamic	0.1Hz qPCR for 8h, 16h, 24h; WB for 1h	3%	CESTRA cell strain device + 6-well BioFlex plates coated with collagen type I + stepping motor	equibiaxial	increase (qPCR, GAPDH)	24h: 3.2 (FC)†	increase (WB, GAPDH)	3.2 (ratio)†
Meng et al. (2010)	α-SMA	*ACTA2*	hPDL cells (12-17/n.g., n.g., exp, P3-4, 80% confluence)	dynamic	0.5 Hz for 1h, 3h, 6h, 12h	**0.4%** (4000µstrain)	"a uniaxial four point bending system" (Yu 2009; Sichuan University, patents CN2534576 and CN1425905) consists of a digital control part, an actuator, and cell culture plates)	uniaxial	increase (sqPCR, GAPDH)	12h: 8.4 (ratio)*	increase (WB, GAPDH)	12h: 2 (rel)* / 9.8 (ratio-calc)

[a] Entry given as reported in the study.
[b] All official gene symbols come from the HUGO Gene Nomenclature Committee (HGNC; URL: https://www.genenames.org) after checking specificity of primers with Primer-BLAST.
[c] Gender/Sex of donors: "M" – male, "F" – female; Tooth type: "PM" – premolar, "M" – molar; Cell density: given in cells/well if not otherwise mentioned.
[d] Frequencies labeled bold orange were converted to hertz (Hz) according to its definition using the information reported in the study (in brackets)
[e] Force type deduced from the description of the force apparatus given by the authors.
[f] Gene and protein expression: 1. conclusion of change (increase, decrease…) was given according to the defined criteria in Figure 2; 2. different markers to describe the amount of change; † Information derived from figures using Engauge Digitizer; *Folds calculated by measuring the graphs, without using the Engauge Digitizer; No makers: Information derived from figures by description in the articles

Reference	Gene/ Analyte[a]	Official gene symbol / abbreviation[b]	Cell (age/gender of donors, tooth type, isolation method, passages used, cell density)[a,c]	Force type (stat./ dyn.)[a]	Force duration and frequency[d]	Force magnitude[a]	Force apparatus[a]	Force type: equibiaxial or uniaxial[e]	Gene expression: Increase, decrease, no change (method w/ reference gene); Methods: qPCR, sqPCR, Northern blot[f]	Gene expression: When it reaches peak and peak's magnitude (fold change; times or ratio; unclear = ?)[f]	Protein expression: Increase, decrease, no change (method w/ reference); Methods: ELISA, WB, RIA, EMSA, IF[f]	Protein expression: When it reaches peak and peak's magnitude (times or ratio; unclear = ?)[f]
Miura et al. (2000)	PA	*PLAT; PLAU*	hPDL cells (12/M, 10/M, 11/F, exp, PM, P5-7 and P19-22, confluent)	dynamic	**0.1Hz** (6cyc/min: 5s elongation and 5s relaxation) for 5d	9%, 18%	Flexercell strain unit + culture plates coated with type I collagen (Flexcell)	equibiaxial	n.g.	n.g.	9%: increase (PA activity, chromogenic substrate assay)<br>15%: increase (PA activity, chromogenic substrate assay)	"young cells" (P5-7) @ 9%: 5.9 (mU/10^5cells)* / 1.6 (ratio-calc)<br>"old cells" (P19-22) @ 9%: 8.2 (mU/10^5cells)* / 2.2 (ratio-calc)<br>"young cells" (P5-7) @ 15%: 8 (mU/10^5cells)* / 2.2 (ratio-calc)<br>"old cells" (P19-22) @ 15%:10.9 (mU/10^5cells)* / 2.9 (ratio-calc)
Miura et al. (2000)	PA	*PLAT; PLAU*	hPDL cells (12/M, 10/M, 11/F, exp, PM, P5-7 and P19-22, confluent)	dynamic	**0.1Hz** (6cyc/min: 5s elongation and 5s relaxation) for 1d, 3d, 5d	18%	Flexercell strain unit + culture plates coated with type I collagen (Flexcell)	equibiaxial	n.g.	n.g.	"young cells" (P5-7): increase (PA activity, chromogenic substrate assay)<br>"old cells" (P19-22): increase (PA activity, chromogenic substrate assay)	"young cells" @ 5d: 7.8 (mU/10^5cells)* / 2.1 (ratio-calc)<br>"old cells" @ 5d: 11.8 (mU/10^5cells)* / 3 (ratio-calc)
Miura et al. (2000)	PA	*PLAT; PLAU*	hPDL cells (12/M, 10/M, 11/F, exp, PM, P5-7 and P19-22, confluent)	dynamic	**0.1Hz** (6cyc/min: 5s elongation and 5s relaxation) for duration n.g.	18%	Flexercell strain unit + culture plates coated with type I collagen (Flexcell)	equibiaxial	n.g.	n.g.	"young cells" (P5-7): increase (PA activity, chromogenic substrate assay)<br>"old cells" (P19-22): increase (PA activity, chromogenic substrate assay)	Donor 1, "young cells" : 7.9 (mU/10^5cells)† / 2.2 (ratio-calc)<br>Donor 1, "old cells" : 11.5 (mU/10^5cells)† / 3.1 (ratio-calc)<br>Donor 1, "young cells" : 2.5 (U/mg protein)† / 1.8 (ratio-calc)<br>Donor 1, "old cells" : 3.0 (U/mg protein)† / 2.2 (ratio-calc)<br>Donor 2, "young cells": 7.6 (mU/10^5cells)† / 2.3 (ratio-calc)<br>Donor 2, "old cells" : 12.3 (mU/10^5cells)† / 3.4 (ratio-calc)<br>Donor 2, "young cells" : 2.4 (U/mg protein)† / 1.6 (ratio-calc)<br>Donor 2, "old cells" : 2.9 (U/mg protein)† / 2.1 (ratio-calc)<br>Donor 3, "young cells" : 7.2 (mU/10^5cells)† / 2.4 (ratio-calc)<br>Donor 3, "old cells" : 12.6 (mU/10^5cells)† / 3.9 (ratio-calc)<br>Donor 3, "young cells" : 2.5 (U/mg protein)† / 1.6 (ratio-calc)<br>Donor 3, "old cells" : 2.9 (U/mg protein)† / 2.1 (ratio-calc)
Miura et al. (2000)	PAI-1	*SERPINE1*	hPDL cells (12/M, 10/M, 11/F, exp, PM, P5-7 and P19-22, confluent)	dynamic	**0.1Hz** (6cyc/min: 5s elongation and 5s relaxation) for duration n.g.	18%	Flexercell strain unit + culture plates coated with type I collagen (Flexcell)	equibiaxial	"young cells" (P5-7): no change (sqPCR, GAPDH)<br>"old cells" (P19-22): no change (sqPCR, GAPDH)	no quantitative information is given	n.g.	n.g.
Miura et al. (2000)	tPA	*PLAT*	hPDL cells (12/M, 10/M, 11/F, exp, PM, P5-7 and P19-22, confluent)	dynamic	**0.1Hz** (6cyc/min: 5s elongation and 5s relaxation) for duration n.g.	18%	Flexercell strain unit + culture plates coated with type I collagen (Flexcell)	equibiaxial	"young cells" (P5-7): increase (sqPCR, GAPDH)<br>"old cells" (P19-22): increase (sqPCR, GAPDH)	no quantitative information is given	"young cells": increase (WB)<br>"old cells": increase (WB)	no quantitative information is given
Miura et al. (2000)	uPA	*PLAU*	hPDL cells (12/M, 10/M, 11/F, exp, PM, P5-7 and P19-22, confluent)	dynamic	**0.1Hz** (6cyc/min: 5s elongation and 5s relaxation) for duration n.g.	18%	Flexercell strain unit + culture plates coated with type I collagen (Flexcell)	equibiaxial	"young cells" (P5-7): no expression (sqPCR, GAPDH)<br>"old cells" (P19-22): no expression (sqPCR, GAPDH)	no quantitative information is given	"young cells": no expression (WB)<br>"old cells": no expression (WB)	no quantitative information is given
Molina et al. (2001)	Focal Adhesion Kinase 1 ($p125^{FAK}$) / p-FAK	*PTK2*	hPDL cells (12-14/n.g., PM, exp, P3-6, confluence)	static	15min, 30min, 45min, 1h, 24h, 48h, 72h	2.5%	Petriperm dishes stretched by being placed on top of a spheroidal convex template (Saito et al1991) + weight	equibiaxial	n.g.	n.g.	p-FAK: increase followed by plateau then decrease (WB, FAK total)	p-FAK: highest @ 45…60min: 7.1% (rel)* / 2.3 (ratio-calc)<br>p-FAK: lowest @ 72min: 2% (rel)* / 0.6 (ratio-calc)
Monnouchi et al. (2011)	ACE	*ACE*	hPDL cells (30/F, 39/F, 26/m, M, dig, P5-6, Sub-confluence)	dynamic	**1Hz** (0.5s stretch and 0.5s relaxation per cycle) for 1h	8%, 12%	STB-140 (STREX Co) + culture chambers coated with type I collagen (Cell matrix I-P; Nitta Gelatin Inc., Osaka, Japan)	uniaxial	increase (sqPCR, GAPDH)	8%: 6.1 (FC)*	n.g.	n.g.
Monnouchi et al. (2011)	AGT	*AGT*	hPDL cells (30/F, 39/F, 26/m, M, dig, P5-6, Sub-confluence)	dynamic	**1Hz** (0.5s stretch and 0.5s relaxation per cycle) for 1h	8%	STB-140 (STREX Co) + culture chambers coated with type I collagen (Cell matrix I-P; Nitta Gelatin Inc., Osaka, Japan)	uniaxial	increase (qPCR, β-actin)	HPLF-2E: 1.8 (FC)*<br>HPLF-2D: 2.3 (FC)*<br>HPLF-3M: 1.6 (FC)*	n.g.	n.g.
Monnouchi et al. (2011)	ALP	*ALPP*	hPDL cells (30/F, 39/F, 26/m, M, dig, P5-6, Sub-confluence)	dynamic	**1Hz** (0.5s stretch and 0.5s relaxation per cycle) for 1h	8%	STB-140 (STREX Co) + culture chambers coated with type I collagen (Cell matrix I-P; Nitta Gelatin Inc., Osaka, Japan)	uniaxial	increase (qPCR, β-actin)	HPLF-2E: 2.3 (FC)*<br>HPLF-2D: 2.1 (FC)*<br>HPLF-3M: 2.9 (FC)*	n.g.	n.g.
Monnouchi et al. (2011)	AT1	*AGTR1*	hPDL cells (30/F, 39/F, 26/m, M, dig, P5-6, Sub-confluence)	dynamic	**1Hz** (0.5s stretch and 0.5s relaxation per cycle) for 1h	8%	STB-140 (STREX Co) + culture chambers coated with type I collagen (Cell matrix I-P; Nitta Gelatin Inc., Osaka, Japan)	uniaxial	increase (sqPCR, GAPDH)	HPLF-2E: no quantitative information is given<br>HPLF-2D: no quantitative information is given	increase (WB, β-actin)	HPLF-3M: no quantitative information is given

[a] Entry given as reported in the study.
[b] All official gene symbols come from the HUGO Gene Nomenclature Committee (HGNC; URL: https://www.genenames.org) after checking specificity of primers with Primer-BLAST.
[c] Gender/Sex of donors: "M" – male, "F" – female; Tooth type: "PM" – premolar, "M" – molar; Cell density: given in cells/well if not otherwise mentioned.
[d] Frequencies labeled bold orange were converted to hertz (Hz) according to its definition using the information reported in the study (in brackets)
[e] Force type deduced from the description of the force apparatus given by the authors.
[f] Gene and protein expression: 1. conclusion of change (increase, decrease…) was given according to the defined criteria in Figure 2; 2. different markers to describe the amount of change; † Information derived from figures using Engauge Digitizer; *Folds calculated by measuring the graphs, without using the Engauge Digitizer; No makers: Information derived from figures by description in the articles

Reference	Gene/ Analyte[a]	Official gene symbol / abbreviation[b]	Cell (age/gender of donors, tooth type, isolation method, passages used, cell density)[a,c]	Force type (stat./ dyn.)[a]	Force duration and frequency[d]	Force magnitude[a]	Force apparatus[a]	Force type: equibiaxial or uniaxial[e]	Gene expression: Increase, decrease, no change (method w/ reference gene); Methods: qPCR, sqPCR, Northern blot[f]	Gene expression: When it reaches peak and peak's magnitude (fold change; times or ratio; unclear = ?)[f]	Protein expression: Increase, decrease, no change (method w/ reference); Methods: ELISA, WB, RIA, EMSA, IF[f]	Protein expression: When it reaches peak and peak's magnitude (times or ratio; unclear = ?)[f]
Monnouchi et al. (2011)	AT2	*AGTR2*	hPDL cells (30/F, 39/F, 26/m, M, dig, P5-6, Sub-confluence)	dynamic	**1Hz** (0.5s stretch and 0.5s relaxation per cycle) for 1h	8%	STB-140 (STREX Co) + culture chambers coated with type I collagen (Cell matrix I-P; Nitta Gelatin Inc., Osaka, Japan)	uniaxial	no change (sqPCR, GAPDH)	HPLF-2E: no quantitative information is given<br>HPLF-2D: no quantitative information is given	no change (WB, β-actin)	HPLF-3M: no quantitative information is given
Monnouchi et al. (2011)	OPG	*TNFRSF11B*	hPDL cells (30/F, 39/F, 26/m, M, dig, P5-6, Sub-confluence)	dynamic	**1Hz** (0.5s stretch and 0.5s relaxation per cycle) for 1h	8%, 12%	STB-140 (STREX Co) + culture chambers coated with type I collagen (Cell matrix I-P; Nitta Gelatin Inc., Osaka, Japan)	uniaxial	increase (sqPCR, GAPDH)	HPDLF-2E: 12%: 2.2 (FC)*	n.g.	n.g.
Monnouchi et al. (2011)	RANKL	*TNFSF11*	hPDL cells (30/F, 39/F, 26/m, M, dig, P5-6, Sub-confluence)	dynamic	**1Hz** (0.5s stretch and 0.5s relaxation per cycle) for 1h	8%, 12%	STB-140 (STREX Co) + culture chambers coated with type I collagen (Cell matrix I-P; Nitta Gelatin Inc., Osaka, Japan)	uniaxial	decrease followed by inccrease (sqPCR, GAPDH)	HPDLF-2E: lowest @ 8%: 0.5 (FC)*<br>HPDLF-2E: highest @ 12%: 1.3 (FC)*	n.g.	n.g.
Monnouchi et al. (2011)	Renin	*REN*	hPDL cells (30/F, 39/F, 26/m, M, dig, P5-6, Sub-confluence)	dynamic	**1Hz** (0.5s stretch and 0.5s relaxation per cycle) for 1h	8%, 12%	STB-140 (STREX Co) + culture chambers coated with type I collagen (Cell matrix I-P; Nitta Gelatin Inc., Osaka, Japan)	uniaxial	increase (sqPCR, GAPDH)	8%: 2.4 (FC)*	n.g.	n.g.
Monnouchi et al. (2011)	TGF-β1	*TGFB1*	hPDL cells (30/F, 39/F, 26/m, M, dig, P5-6, Sub-confluence)	dynamic	**1Hz** (0.5s stretch and 0.5s relaxation per cycle) for 1h	8%	STB-140 (STREX Co) + culture chambers coated with type I collagen (Cell matrix I-P; Nitta Gelatin Inc., Osaka, Japan)	uniaxial	increase (qPCR, β-actin)	HPLF-2E: 2.6 (FC)*<br>HPLF-2D: 1.7 (FC)*<br>HPLF-3M: 2.1 (FC)*	n.g.	n.g.
Monnouchi et al. (2015)	IL-11	*IL11*	hPDL cells (30/F, 39/F, 26/M, M, exp, P5-7, 2×10^4/cm²)	dynamic	**1Hz** (0.5s stretch and 0.5s relaxation per cycle) for 1h	8%	STB-140 (STREX Co) + culture chambers coated with type I collagen (Cell matrix I-P; Nitta Gelatin Inc., Osaka, Japan) (Monnouchi 2011)	uniaxial	increase (qPCR, β-actin)	HPDLC-2D: 2.6 (FC)*<br>HPDLC-2E: 5.5 (FC)*<br>HPDLC-3M: 3.5 (FC)*	increase (ELISA)	HPDLC-2D: 124.0 (pg/ml)* / 4 (ratio-calc)<br>HPDLC-2E: 88.0 (pg/ml)* / 2.8 (ratio-calc)<br>HPDLC-3M: 217.0 (pg/ml)* / 2.3 (ratio-calc)
Nakashima et al. (2009)	Fibulin-5	*FBLN5*	hPDL cells (n.g./n.g., M, exp, P6, Confluent)	dynamic	**0.01Hz** (1/60Hz) for 7d	5%	STB-140 STREX cell stretch system (Strex Co) + elastic silicone chamber	uniaxial	increase (Northern blot, β-actin)	1.5 (ratio)	increase (WB, β-actin)	1.9 (ratio)
Narimiya et al. (2017)	MMP-12	*MMP12*	Human immortalized PDLCs (n.g./n.g., n.g., gene transfection, n.g., 4×10^5)	static	qPCR for 6h, 12h, 24h; ELISA for 24h WB for 24h	15%	Cell Extender version3 (MOLCURE, Tokyo, Japan) + Bioflex® plates (Flexcell) + actuator (Wada 2017)	equibiaxial	increase (qPCR, GAPDH)	24h: 10.5 (FC)*	increase (ELISA)<br>increase (WB)	ELISA: 420 (pg/ml)* / 1.5 (ratio-calc)<br>WB: no quantitative information given
Narimiya et al. (2017)	TIMP-1	*TIMP1*	Human immortalized PDLCs (n.g./n.g., n.g., gene transfection, n.g., 4×10^5)	static	24h	15%	Cell Extender version3 (MOLCURE, Tokyo, Japan) + Bioflex® plates (Flexcell) + actuator (Wada 2017)	equibiaxial	no change (qPCR, GAPDH)		n.g.	n.g.
Narimiya et al. (2017)	TIMP-2	*TIMP2*	Human immortalized PDLCs (n.g./n.g., n.g., gene transfection, n.g., 4×10^5)	static	24h	15%	Cell Extender version3 (MOLCURE, Tokyo, Japan) + Bioflex® plates (Flexcell) + actuator (Wada 2017)	equibiaxial	no change (qPCR, GAPDH)		n.g.	n.g.
Narimiya et al. (2017)	TIMP-3	*TIMP3*	Human immortalized PDLCs (n.g./n.g., n.g., gene transfection, n.g., 4×10^5)	static	24h	15%	Cell Extender version3 (MOLCURE, Tokyo, Japan) + Bioflex® plates (Flexcell) + actuator (Wada 2017)	equibiaxial	no change (qPCR, GAPDH)		n.g.	n.g.
Nazet et al. (2020)	ALP	*ALPP*	hPDLF (17-27/F, 17-27/M, M, n.g., P3-6, 7×10^4)	static	24h, 48h, 72h	35%	six-well bioflex membrane plates coated with collagen I + custom-made spherical cap silicone stamps	equibiaxial	n.g.	n.g.	increase followed by platform (ELISA)	48h...72h: 1.4 (ratio)†
Nazet et al. (2020)	ALP	*ALPP*	hPDLF (17-27/F, 17-27/M, M, n.g., P3-6, 7×10^4)	static	48h	7%, 10%, 16%. 35%	six-well bioflex membrane plates coated with collagen I + custom-made spherical cap silicone stamps	equibiaxial	n.g.	n.g.	increase (ELISA)	16%: 1.5 (ratio)†
Nazet et al. (2020)	COX-2	*PTGS2*	hPDLF (17-27/F, 17-27/M, M, n.g., P3-6, 7×10^4)	static	24h, 48h, 72h	35%	six-well bioflex membrane plates coated with collagen I + custom-made spherical cap silicone stamps	equibiaxial	temporary increase (qPCR, TBP/PPIB)	48h: 2.2 (FC)†	n.g.	n.g.
Nazet et al. (2020)	COX-2	*PTGS2*	hPDLF (17-27/F, 17-27/M, M, n.g., P3-6, 7×10^4)	static	48h	7%, 10%, 16%. 35%	six-well bioflex membrane plates coated with collagen I + custom-made spherical cap silicone stamps	equibiaxial	increase (qPCR, TBP/PPIB)	35%: 2.5 (FC)†	n.g.	n.g.
Nazet et al. (2020)	IL-6	*IL6*	hPDLF (17-27/F, 17-27/M, M, n.g., P3-6, 7×10^4)	static	24h, 48h, 72h	35%	six-well bioflex membrane plates coated with collagen I + custom-made spherical cap silicone stamps	equibiaxial	temporary decrease (qPCR, TBP/PPIB)	48h: 0.6 (FC)†	n.g.	n.g.
Nazet et al. (2020)	IL-6	*IL6*	hPDLF (17-27/F, 17-27/M, M, n.g., P3-6, 7×10^4)	static	48h	7%, 10%, 16%. 35%	six-well bioflex membrane plates coated with collagen I + custom-made spherical cap silicone stamps	equibiaxial	decrease (qPCR, TBP/PPIB)	35%: 0.7 (FC)†	n.g.	n.g.
Nemoto et al. (2010)	ARRAY	ARRAY	hPDL cells (20/n.g., 40/n.g., M, dig, P4-8, 5×10^5 cells/cm²)	dynamic	**0.017Hz (1/60Hz)** (conditions: 60s/returns; resting time: 29s) short time for 1h, 3h, 12h, 24h, 48h; long time for 1d, 3d, 5d, 7d	**5%** (Stretch length: 1.6mm, stretch ratio: 105%)	STB-140 (Strex Co) + 50 cm² silicon chambers coated with 50 mg/ml COL1 + stepping motor	uniaxial	Gene Chip Human Genome U133 plus (Agilent)	too many	n.g.	n.g.

[a] Entry given as reported in the study.
[b] All official gene symbols come from the HUGO Gene Nomenclature Committee (HGNC; URL: https://www.genenames.org) after checking specificity of primers with Primer-BLAST.
[c] Gender/Sex of donors: "M" – male, "F" – female; Tooth type: "PM" – premolar, "M" – molar; Cell density: given in cells/well if not otherwise mentioned.
[d] Frequencies labeled bold orange were converted to hertz (Hz) according to its definition using the information reported in the study (in brackets)
[e] Force type deduced from the description of the force apparatus given by the authors.
[f] Gene and protein expression: 1. conclusion of change (increase, decrease…) was given according to the defined criteria in Figure 2; 2. different markers to describe the amount of change; † Information derived from figures using Engauge Digitizer; *Folds calculated by measuring the graphs, without using the Engauge Digitizer; No makers: Information derived from figures by description in the articles

Reference	Gene/ Analyte[a]	Official gene symbol / abbreviation[b]	Cell (age/gender of donors, tooth type, isolation method, passages used, cell density)[a,c]	Force type (stat./ dyn.)[a]	Force duration and frequency[d]	Force magnitude[a]	Force apparatus[a]	Force type: equibiaxial or uniaxial[e]	Gene expression: Increase, decrease, no change (method w/ reference gene); Methods: qPCR, sqPCR, Northern blot[f]	Gene expression: When it reaches peak and peak's magnitude (fold change; times or ratio; unclear = ?)[f]	Protein expression: Increase, decrease, no change (method w/ reference); Methods: ELISA, WB, RIA, EMSA, IF[f]	Protein expression: When it reaches peak and peak's magnitude (times or ratio; unclear = ?)[f]
Nemoto et al. (2010)	COLIVα1	*COL4A1*	hPDL cells (20/n.g., 40/n.g., M, dig, P4-8, 5×10[5] cells/cm[2])	dynamic	**0.017Hz (1/60Hz)** (Conditions: 60s/returns; resting time: 29s) short time for 1h, 3h, 12h, 24h, 48h; long time for 1d, 3d, 5d, 7d	**5%** (Stretch length: 1.6mm, stretch ratio: 105%)	STB-140 (Strex Co) + 50 cm[2] silicon chambers coated with 50 mg/ml COL1 + stepping motor	uniaxial	short time: increase followed by plateau (qPCR, GAPDH)<br>long time: increase (qPCR, GAPDH)	short time @ 1h…48h: 0.3 (rel)* / 1.7 (ratio-calc)<br>long time @ 7d: 0.5 (rel)* / 5.4 (ratio-calc)	n.g.	n.g.
Nemoto et al. (2010)	COLIα1	*COL1A1*	hPDL cells (20/n.g., 40/n.g., M, dig, P4-8, 5×10[5] cells/cm[2])	dynamic	**0.017Hz (1/60Hz)** (Conditions: 60s/returns; resting time: 29s) short time for 1h, 3h, 12h, 24h, 48h; long time for 1d, 3d, 5d, 7d	**5%** (Stretch length: 1.6mm, stretch ratio: 105%)	STB-140 (Strex Co) + 50 cm[2] silicon chambers coated with 50 mg/ml COL1 + stepping motor	uniaxial	short time: decrease (qPCR, GAPDH)<br>long time: decrease (qPCR, GAPDH)	short time @ 24h: 0.3 (rel)* / 0.3 (ratio-calc)<br>long time @ 3d: 0.2 (rel)* / 0.3 (ratio-calc)	n.g.	n.g.
Nemoto et al. (2010)	COLXIIα1	*COL12A1*	hPDL cells (20/n.g., 40/n.g., M, dig, P4-8, 5×10[5] cells/cm[2])	dynamic	**0.017Hz (1/60Hz)** (conditions: 60s/returns; resting time: 29s) short time for 1h, 3h, 12h, 24h, 48h; long time for 1d, 3d, 5d, 7d	**5%** (Stretch length: 1.6mm, stretch ratio: 105%)	STB-140 (Strex Co) + 50 cm[2] silicon chambers coated with 50 mg/ml COL1 + stepping motor	uniaxial	short time: decrease (qPCR, GAPDH)<br>long time: decrease (qPCR, GAPDH)	short time @ 12h: 0.1 (rel)* / 0.3 (ratio-calc)<br>long time @ 1d: 0.2 (rel)* / 0.4 (ratio-calc)	n.g.	n.g.
Ngan et al. (1990)	cAMP	cAMP	hPDL cells (n.g./n.g., PM, dig, P4-6, 4×10[5] cells/cm[2])	static	5min, 15min, 30min, 60min	n.g.	Petriperm dishes + spheroidal convex template + weight	equibiaxial	n.a.	n.a.	increase (cAMP assay)	60min: 0.8 (pmol/10[4] PDL cells)* / 1.1 (ratio-calc)
Ngan et al. (1990)	PGE_2	PGE_2	hPDL cells (n.g./n.g., PM, dig, P4-6, 4×10[5] cells/cm[2])	static	5min, 15min, 30min, 60min	0.28%, 0.95%, 1.09%, 1.72%	Petriperm dishes + spheroidal convex template + weight	equibiaxial	n.a.	n.a.	0.28%: increase (RIA)<br>0.95%: temporary decrease (RIA)<br>1.09%: increase (RIA)<br>1.72%: increase (RIA)	0.28% @ 120min: 109 (pg/10[4] PDL cells)* / 1.1 (ratio-calc)<br>0.95% @ 15min: 98 (pg/10[4] PDL cells)* / 0.9 (ratio-calc)<br>1.09% @ 120min: 111 (pg/10[4] PDL cells)* / 1.1 (ratio-calc)<br>1.72% @ 30min: 101 (pg/10[4] PDL cells)* / 1.7 (ratio-calc)
Nogueira et al. (2014a)	TLR2	*TLR2*	hPDL cells (n.g./n.g., n.g., n.g., P3-5, 80% confluence)	dynamic	0.05Hz for 1d, 3d	3%, 20%	strain device (CESTRA) + six-well BioFlex® collagen-coated culture plates + stepping motor (Nokhbehsaim 2012; further reference to Rath-Deschner 2009)	equibiaxial	3%: increase followed by decrease (qPCR, GAPDH)<br>20%: decrease (qPCR, GAPDH)	3% highest @ 1d: 1.1 (FC)*<br>3% lowest @ 3d: 0.88 (FC)<br>20% @ 3d: 0.31 (FC)	n.g.	n.g.
Nogueira et al. (2014a)	TLR4	*TLR4*	hPDL cells (n.g./n.g., n.g., n.g., P3-5, 80% confluence)	dynamic	0.05Hz for 1d, 3d	3%, 20%	strain device (CESTRA) + six-well BioFlex® collagen-coated culture plates + stepping motor (Nokhbehsaim 2012; further reference to Rath-Deschner 2009)	equibiaxial	3%: decrease (qPCR, GAPDH)<br>20%: decrease (qPCR, GAPDH)	3% @ 3d: 0.88 (FC)<br>20% @ 3d: 0.48 (FC)	n.g.	n.g.
Nogueira et al. (2014a)	Visfatin	*NAMPT*	hPDL cells (n.g./n.g., n.g., n.g., P3-5, 80% confluence)	dynamic	0.05Hz for 1d, 3d	3%, 20%	strain device (CESTRA) + six-well BioFlex® collagen-coated culture plates + stepping motor (Nokhbehsaim 2012; further reference to Rath-Deschner 2009)	equibiaxial	1d: decrease followed by plateau (qPCR, GAPDH)<br>3d: decrease (qPCR, GAPDH)	3...20% @ 1d: 0.7 (FC)†<br>20% @ 3d: 0.6 (FC)†	n.g.	n.g.
Nogueira et al. (2014b)	COX2	*PTGS2*	hPDL cells (n.g./n.g., n.g., n.g., P3-5, 80% confluence)	dynamic	0.05Hz for 36h	3%, 20%	strain device (CESTRA) + six-well BioFlex® collagen-coated culture plates + stepping motor	equibiaxial	n.g.	n.g.	increase (ELISA)	20%: 210 (pg/mL)* / 1.8 (ratio-calc)
Nogueira et al. (2014b)	OPG	*TNFRSF11B*	hPDL cells (n.g./n.g., n.g., n.g., P3-5, 80% confluence)	dynamic	0.05Hz for 1d, 3d	20%	strain device (CESTRA) + six-well BioFlex® collagen-coated culture plates + stepping motor	equibiaxial	OPG: decrease followed by plateau (qPCR, GAPDH)<br>*RANKL/OPG: increase*	OPG @ 1d…3d: 0.2 (FC)*<br>*RANKL/OPG @ 1d: 4.5 (ratio)**	OPG: decrease (ELISA)<br>*RANKL/OPG: increase*	OPG @ 1d: 0.6 (ratio)*<br>*RANKL/OPG @ 3d: 1.4 (ratio)**
Nogueira et al. (2014b)	PGE_2	PGE_2	hPDL cells (n.g./n.g., n.g., n.g., P3-5, 80% confluence)	dynamic	0.05Hz for 1d, 3d	3%, 20%	strain device (CESTRA) + six-well BioFlex® collagen-coated culture plates + stepping motor	equibiaxial	n.a.	n.a.	1d: increase (ELISA)<br>3d: increase followed by plateau (ELISA)	20% @ 1d: 28.3 (ratio)†<br>3…20% @ 3d: 4.3 (ratio)†
Nogueira et al. (2014b)	RANKL	TNFSF11	hPDL cells (n.g./n.g., n.g., n.g., P3-5, 80% confluence)	dynamic	0.05Hz for 1d, 3d	20%	strain device (CESTRA) + six-well BioFlex® collagen-coated culture plates + stepping motor	equibiaxial	RANKL: decrease (qPCR, GAPDH)<br>*RANKL/OPG: increase*	RANKL @ 3d: 0.3 (FC)*<br>*RANKL/OPG @ 1d: 4.5 (ratio)**	RANKL: decrease (ELISA)<br>*RANKL/OPG: increase*	RANKL @ 1d: 0.6 (ratio)*<br>*RANKL/OPG @ 3d: 1.4 (ratio)**
Nokhbehsaim et al. (2010)	ALP	*ALPP*	hPDL cells (n.g./n.g., n.g., n.g., P3-5, n.g.)	dynamic	0.05Hz for 1d, 6d	3%, 20%	strain device (CESTRA) + six-well BioFlex® collagen-coated culture plates + stepping motor	equibiaxial	3%: increase followed by decrease (qPCR, GAPDH)<br>20%: decrease (qPCR, GAPDH)	3% highest @1d: 1.13 (FC)<br>3% lowest @ 6d: 0.59 (FC)<br>20% @ 6d: 0.34 (FC)	n.g.	n.g.
Nokhbehsaim et al. (2010)	COL1	*COL1A1; COL1A2*	hPDL cells (n.g./n.g., n.g., n.g., P3-5, n.g.)	dynamic	0.05Hz for 1d, 6d	3%, 20%	strain device (CESTRA) + six-well BioFlex® collagen-coated culture plates + stepping motor	equibiaxial	3%: decrease (qPCR, GAPDH)<br>20% decrease (qPCR, GAPDH)	3% @ 1d: 0.25 (FC)<br>20% @ 6d: 0.30 (FC)	n.g.	n.g.
Nokhbehsaim et al. (2010)	COX2	PTGS2	hPDL cells (n.g./n.g., n.g., n.g., P3-5, n.g.)	dynamic	0.05Hz for 1d, 6d	3%, 20%	strain device (CESTRA) + six-well BioFlex® collagen-coated culture plates + stepping motor	equibiaxial	3% temporary increase (qPCR, GAPDH)<br>20% increase (qPCR, GAPDH)	3% @ 1d: 1.29 (FC)<br>20% @ 6d: 3.32 (FC)	n.g.	n.g.
Nokhbehsaim et al. (2010)	IGF1	*IGF1*	hPDL cells (n.g./n.g., n.g., n.g., P3-5, n.g.)	dynamic	0.05Hz for 1d, 6d	3%, 20%	strain device (CESTRA) + six-well BioFlex® collagen-coated culture plates + stepping motor	equibiaxial	3%: decrease followed by plateau (qPCR, GAPDH)<br>20%: decrease (qPCR, GAPDH)	3% @ 1d...6d: 0.57 (FC)<br>20% @ 1d: 0.25 (FC)	n.g.	n.g.

[a] Entry given as reported in the study.
[b] All official gene symbols come from the HUGO Gene Nomenclature Committee (HGNC; URL: https://www.genenames.org) after checking specificity of primers with Primer-BLAST.
[c] Gender/Sex of donors: "M" – male, "F" – female; Tooth type: "PM" – premolar, "M" – molar; Cell density: given in cells/well if not otherwise mentioned.
[d] Frequencies labeled bold orange were converted to hertz (Hz) according to its definition using the information reported in the study (in brackets)
[e] Force type deduced from the description of the force apparatus given by the authors.
[f] Gene and protein expression: 1. conclusion of change (increase, decrease…) was given according to the defined criteria in Figure 2; 2. different markers to describe the amount of change; † Information derived from figures using Engauge Digitizer; *Folds calculated by measuring the graphs, without using the Engauge Digitizer; No makers: Information derived from figures by description in the articles

Reference	Gene/ Analyte[a]	Official gene symbol / abbreviation[b]	Cell (age/gender of donors, tooth type, isolation method, passages used, cell density)[a,c]	Force type (stat./ dyn.)[a]	Force duration and frequency[d]	Force magnitude[a]	Force apparatus[a]	Force type: equibiaxial or uniaxial[e]	Gene expression: Increase, decrease, no change (method w/ reference gene); Methods: qPCR, sqPCR, Northern blot[f]	Gene expression: When it reaches peak and peak's magnitude (fold change; times or ratio; unclear = ?)[f]	Protein expression: Increase, decrease, no change (method w/ reference); Methods: ELISA, WB, RIA, EMSA, IF[f]	Protein expression: When it reaches peak and peak's magnitude (times or ratio; unclear = ?)[f]
Nokhbehsaim et al. (2010)	IL-1B	*IL1B*	hPDL cells (n.g./n.g., n.g., n.g., P3-5, n.g.)	dynamic	0.05Hz for 1d, 6d	3%, 20%	strain device (CESTRA) + six-well BioFlex® collagen-coated culture plates + stepping motor	equibiaxial	3%: increase (qPCR, GAPDH)<br>20%: increase (qPCR, GAPDH)	3% @ 1d: 12.41 (FC)<br>20% @ 6d: 36.16 (FC)	n.g.	n.g.
Nokhbehsaim et al. (2010)	IL-8	*CXCL8*	hPDL cells (n.g./n.g., n.g., n.g., P3-5, n.g.)	dynamic	0.05Hz qPCR for 1d, 6d; ELISA for 1d	3%, 20%	strain device (CESTRA) + six-well BioFlex® collagen-coated culture plates + stepping motor	equibiaxial	3%: increase (qPCR, GAPDH)<br>20%: increase (qPCR, GAPDH)	3% @ 6d: 17.48 (FC)<br>20% @ 6d: 7.54 (FC)	3%: increase (ELISA)<br>20%: increase (ELISA)	3%: 394.16 (pg/10^5 cells) / 11.6 (ratio-calc)<br>20%: 109.47 (pg/10^5 cells) / 3.2 (ratio-calc)
Nokhbehsaim et al. (2010)	RUNX2	*RUNX2*	hPDL cells (n.g./n.g., n.g., n.g., P3-5, n.g.)	dynamic	0.05Hz for 1d, 6d	3%, 20%	strain device (CESTRA) + six-well BioFlex® collagen-coated culture plates + stepping motor	equibiaxial	3%: decrease (qPCR, GAPDH)<br>20%: decrease (qPCR, GAPDH)	3% @ 1d: 0.24 (FC)<br>20% @ 1d: 0.27 (FC)	n.g.	n.g.
Nokhbehsaim et al. (2010)	TGFB1	*TGFB1*	hPDL cells (n.g./n.g., n.g., n.g., P3-5, n.g.)	dynamic	0.05Hz for 1d, 6d	3%, 20%	strain device (CESTRA) + six-well BioFlex® collagen-coated culture plates + stepping motor	equibiaxial	3%: temporary decrease (qPCR, GAPDH)<br>20%: increase (qPCR, GAPDH)	3% @ 1d: 0.58 (FC)<br>20% @ 6d: 1.38 (FC)	n.g.	n.g.
Nokhbehsaim et al. (2010)	VEGF	*VEGFA*	hPDL cells (n.g./n.g., n.g., n.g., P3-5, n.g.)	dynamic	0.05Hz for 1d, 6d	3%, 20%	strain device (CESTRA) + six-well BioFlex® collagen-coated culture plates + stepping motor	equibiaxial	3%: temporary decrease (qPCR, GAPDH)<br>20%: increase (qPCR, GAPDH)	3% @ 1d: 0.70 (FC)<br>20% @ 6d: 2.51 (FC)	3%: decrease (ELISA)<br>20%: increase (ELISA)	3%: 431.81 (pg/10^5 cells) / 0.1 (ratio-calc)<br>20%: 4669.43 (pg/10^5 cells) / 1.4 (ratio-calc)
Nokhbehsaim et al. (2011a)	BMPR1A	*BMPR1A*	hPDL cells (8-17/F, 8-14/M, n.g., n.g., P3-5, 80% confluence)	dynamic	0.05Hz for 6d	3%	strain device (CESTRA) + six-well BioFlex® collagen-coated culture plates + stepping motor (Rath-Deschner 2009)	equibiaxial	increase (qPCR, GAPDH)	1.2 (FC)*	n.g.	n.g.
Nokhbehsaim et al. (2011a)	BMPR1B	*BMPR1B*	hPDL cells (8-17/F, 8-14/M, n.g., n.g., P3-5, 80% confluence)	dynamic	0.05Hz for 6d	3%	strain device (CESTRA) + six-well BioFlex® collagen-coated culture plates + stepping motor (Rath-Deschner 2009)	equibiaxial	no change (qPCR, GAPDH)		n.g.	n.g.
Nokhbehsaim et al. (2011a)	BMPR2	*BMPR2*	hPDL cells (8-17/F, 8-14/M, n.g., n.g., P3-5, 80% confluence)	dynamic	0.05Hz for 6d	3%	strain device (CESTRA) + six-well BioFlex® collagen-coated culture plates + stepping motor (Rath-Deschner 2009)	equibiaxial	decrease (qPCR, GAPDH)	0.8 (FC)*	n.g.	n.g.
Nokhbehsaim et al. (2011a)	Collagen I (COL1)	*COL1A1; COL1A2*	hPDL cells (8-17/F, 8-14/M, n.g., n.g., P3-5, 80% confluence)	dynamic	0.05Hz for 1d, 6d	3%	strain device (CESTRA) + six-well BioFlex® collagen-coated culture plates + stepping motor (Rath-Deschner 2009)	equibiaxial	decrease (qPCR, GAPDH)	1d: 0.3 (FC)*	n.g.	n.g.
Nokhbehsaim et al. (2011a)	RUNX2	*RUNX2*	hPDL cells (8-17/F, 8-14/M, n.g., n.g., P3-5, 80% confluence)	dynamic	0.05Hz for 1d, 6d	3%	strain device (CESTRA) + six-well BioFlex® collagen-coated culture plates + stepping motor	equibiaxial	decrease (qPCR, GAPDH)	1d: 0.2 (FC)*	n.g.	n.g.
Nokhbehsaim et al. (2011a)	TGF-β1	*TGFB1*	hPDL cells (8-17/F, 8-14/M, n.g., n.g., P3-5, 80% confluence)	dynamic	0.05Hz for 1d, 6d	3%	strain device (CESTRA) + six-well BioFlex® collagen-coated culture plates + stepping motor	equibiaxial	temporary decrease (qPCR, GAPDH)	1d: 0.6 (FC)*	n.g.	n.g.
Nokhbehsaim et al. (2011a)	TGF-βR1	*TGFBR1*	hPDL cells (8-17/F, 8-14/M, n.g., n.g., P3-5, 80% confluence)	dynamic	0.05Hz for 6d	3%	strain device (CESTRA) + six-well BioFlex® collagen-coated culture plates + stepping motor	equibiaxial	no change (qPCR, GAPDH)		n.g.	n.g.
Nokhbehsaim et al. (2011a)	TGF-βR2	*TGFBR2*	hPDL cells (8-17/F, 8-14/M, n.g., n.g., P3-5, 80% confluence)	dynamic	0.05Hz for 6d	3%	strain device (CESTRA) + six-well BioFlex® collagen-coated culture plates + stepping motor	equibiaxial	decrease (qPCR, GAPDH)	0.8 (FC)*	n.g.	n.g.
Nokhbehsaim et al. (2011a)	VEGF	*VEGFA*	hPDL cells (8-17/F, 8-14/M, n.g., n.g., P3-5, 80% confluence)	dynamic	0.05Hz for 1d, 6d	3%	strain device (CESTRA) + six-well BioFlex® collagen-coated culture plates + stepping motor	equibiaxial	temporary decrease (qPCR, GAPDH)	1d: 0.7 (FC)*	n.g.	n.g.
Nokhbehsaim et al. (2011b)	BMP-2	*BMP2*	hPDL cells (n.g./n.g., n.g., n.g., P3-5, 80% confluen)	dynamic	0.05Hz for 1d, 6d	3%, 20%	strain device (CESTRA) + six-well BioFlex® collagen-coated culture plates + stepping motor (Rath-Deschner 2009)	equibiaxial	3%: decrease (qPCR, GAPDH)<br>20%: increase (qPCR, GAPDH)	3% @ 6d: 0.59 (FC)<br>20% @ 1d: 2.6 (FC)	n.g.	n.g.
Nokhbehsaim et al. (2011b)	Follistatin	*FST*	hPDL cells (n.g./n.g., n.g., n.g., P3-5, 80% confluen)	dynamic	0.05Hz for 1d, 6d	3%, 20%	strain device (CESTRA) + six-well BioFlex® collagen-coated culture plates + stepping motor (Rath-Deschner 2009)	equibiaxial	3%: decrease (qPCR, GAPDH)<br>20%: decrease (qPCR, GAPDH)	3% @ 1d: 0.5 (FC)*<br>20% @ 6d: 0.6 (FC)*	n.g.	n.g.
Nokhbehsaim et al. (2011b)	MGP	*MGP*	hPDL cells (n.g./n.g., n.g., n.g., P3-5, 80% confluen)	dynamic	0.05Hz for 1d, 6d	3%, 20%	strain device (CESTRA) + six-well BioFlex® collagen-coated culture plates + stepping motor (Rath-Deschner 2009)	equibiaxial	3%: decrease (qPCR, GAPDH)<br>20%: decrease (qPCR, GAPDH)	3% @ 6d: 0.4 (FC)*<br>20% @ 6d: 0.3 (FC)*	n.g.	n.g.
Nokhbehsaim et al. (2011b)	Noggin	*NOG*	hPDL cells (n.g./n.g., n.g., n.g., P3-5, 80% confluen)	dynamic	0.05Hz for 1d, 6d	3%, 20%	strain device (CESTRA) + six-well BioFlex® collagen-coated culture plates + stepping motor (Rath-Deschner 2009)	equibiaxial	3%: decrease followed by plateau (qPCR, GAPDH)<br>20%: decrease followed by plateau (qPCR, GAPDH)	3% @ 1d...6d: 0.5 (FC)*<br>20% @ 1d...6d: 0.6 (FC)*	n.g.	n.g.
Nokhbehsaim et al. (2012)	COX2	*PTGS2*	hPDL cells (n.g./n.g., n.g., n.g., P3-5, n.g.)	dynamic	0.05Hz for 1d, 6d	3%, 20%	strain device (CESTRA) + six-well BioFlex® collagen-coated culture plates + stepping motor (Rath-Deschner 2009)	equibiaxial	3%: no change (qPCR, GAPDH)<br>20%: increase (qPCR, GAPDH)	20% @ 6d: 1.5 (FC)*	n.g.	n.g.
Nokhbehsaim et al. (2012)	IL10	*IL10*	hPDL cells (n.g./n.g., n.g., n.g., P3-5, n.g.)	dynamic	0.05Hz for 1d, 6d	3%, 20%	strain device (CESTRA) + six-well BioFlex® collagen-coated culture plates + stepping motor (Rath-Deschner 2009)	equibiaxial	3%: decrease followed by increase (qPCR, GAPDH)<br>20%: decrease (qPCR, GAPDH)	3% lowest @ 1d: 0.5 (FC)*<br>3% highest @ 6d: 1.6 (FC)*<br>20% @ 1d: 0.7 (FC)*	n.g.	n.g.
Nokhbehsaim et al. (2012)	IL1RN	*IL1RN*	hPDL cells (n.g./n.g., n.g., n.g., P3-5, n.g.)	dynamic	0.05Hz for 1d, 6d	3%, 20%	strain device (CESTRA) + six-well BioFlex® collagen-coated culture plates + stepping motor (Rath-Deschner 2009)	equibiaxial	3%: decrease followed by increase (qPCR, GAPDH)<br>20%: decrease followed by increase (qPCR, GAPDH)	3% lowest @ 1d: 0.7 (FC)*<br>3% highest @ 6d: 2.2 (FC)*<br>20% lowest @ 1d: 0.5 (FC)*<br>20% highest @ 6d: 2 (FC)*	n.g.	n.g.
Nokhbehsaim et al. (2012)	IL-1β	*IL1B*	hPDL cells (n.g./n.g., n.g., n.g., P3-5, n.g.)	dynamic	0.05Hz for 1d, 6d	3%, 20%	strain device (CESTRA) + six-well BioFlex® collagen-coated culture plates + stepping motor (Rath-Deschner 2009)	equibiaxial	3%: decrease (qPCR, GAPDH)<br>20%: decrease (qPCR, GAPDH)	3% @ 6d: 0.08 (FC)*<br>20% @ 6d: 0.5 (FC)*	n.g.	n.g.
Nokhbehsaim et al. (2012)	IL-6	*IL6*	hPDL cells (n.g./n.g., n.g., n.g., P3-5, n.g.)	dynamic	0.05Hz for qPCR 1d, 6d; ELISA for 1d, 2d	3%, 20%	strain device (CESTRA) + six-well BioFlex® collagen-coated culture plates + stepping motor (Rath-Deschner 2009)	equibiaxial	3%: increase (qPCR, GAPDH)<br>20%: increase (qPCR, GAPDH)	3% @ 1d: 2 (FC)*<br>20% @ 6d: 3.6 (FC)*	3%: decrease (ELISA)<br>20%: decrease followed by increase (ELISA)	3% @ 2d: 1.1 (pg/10^4 cells)†/ 0.04 (ratio-calc)<br>20% lowest @ 1d: 32.4 (pg/10^4 cells)† / 0.8 (ratio-calc)<br>20% highest @ 2d: 63.0 (pg/10^4 cells)† / 2.3 (ratio-calc)
Nokhbehsaim et al. (2012)	IL-8	*CXCL8*	hPDL cells (n.g./n.g., n.g., n.g., P3-5, n.g.)	dynamic	0.05Hz for 1d, 6d	3%, 20%	strain device (CESTRA) + six-well BioFlex® collagen-coated culture plates + stepping motor (Rath-Deschner 2009)	equibiaxial	3%: decrease (qPCR, GAPDH)<br>20%: decrease (qPCR, GAPDH)	3% @ 6d: 0.08 (FC)*<br>20% @ 1d: 0.8 (FC)*	n.g.	n.g.

[a] Entry given as reported in the study.
[b] All official gene symbols come from the HUGO Gene Nomenclature Committee (HGNC; URL: https://www.genenames.org) after checking specificity of primers with Primer-BLAST.
[c] Gender/Sex of donors: "M" – male, "F" – female; Tooth type: "PM" – premolar, "M" – molar; Cell density: given in cells/well if not otherwise mentioned.
[d] Frequencies labeled bold orange were converted to hertz (Hz) according to its definition using the information reported in the study (in brackets)
[e] Force type deduced from the description of the force apparatus given by the authors.
[f] Gene and protein expression: 1. conclusion of change (increase, decrease…) was given according to the defined criteria in Figure 2; 2. different markers to describe the amount of change; † Information derived from figures using Engauge Digitizer; *Folds calculated by measuring the graphs, without using the Engauge Digitizer; No makers: Information derived from figures by description in the articles

Reference	Gene/ Analyte[a]	Official gene symbol / abbreviation[b]	Cell (age/gender of donors, tooth type, isolation method, passages used, cell density)[a,c]	Force type (stat./ dyn.)[a]	Force duration and frequency[d]	Force magnitude[a]	Force apparatus[a]	Force type: equibiaxial or uniaxial[e]	Gene expression: Increase, decrease, no change (method w/ reference gene); Methods: qPCR, sqPCR, Northern blot[f]	Gene expression: When it reaches peak and peak's magnitude (fold change; times or ratio; unclear = ?)[f]	Protein expression: Increase, decrease, no change (method w/ reference); Methods: ELISA, WB, RIA, EMSA, IF[f]	Protein expression: When it reaches peak and peak's magnitude (times or ratio; unclear = ?)[f]
Ohzeki et al. (1999)	COX-1	*PTGS1*	hPDL cells (12/M, 10/M, 11/F , PM, exp, P5-7 and P19-22, Confluent)	dynamic	**0.1Hz** (6cyc/min: 5s elongation and 5s relaxation) for unclear	unclear	Flexercell strain unit (Shimizu et al 1994) + flexible-bottom culture plates coated with type I collagen (Flexcell) + vacuum	equibiaxial	increase (sqPCR, GAPDH)	no quantitative information is given	n.g.	n.g.
Ohzeki et al. (1999)	COX-2	*PTGS2*	hPDL cells (12/M, 10/M, 11/F , PM, exp, P5-7 and P19-22, Confluent)	dynamic	**0.1Hz** (6cyc/min: 5s elongation and 5s relaxation) for unclear	unclear	Flexercell strain unit (Shimizu et al 1994) + flexible-bottom culture plates coated with type I collagen (Flexcell) + vacuum	equibiaxial	increase (sqPCR, GAPDH)	no quantitative information is given	n.g.	n.g.
Ohzeki et al. (1999)	PGE_2	PGE_2	hPDL cells (12/M, 10/M, 11/F , PM, exp, P5-7 and P19-22, Confluent)	dynamic	**0.1Hz** (6cyc/min: 5s elongation and 5s relaxation) for 5d	9%, 18%	Flexercell strain unit (Shimizu et al 1994) + flexible-bottom culture plates coated with type I collagen (Flexcell) + vacuum	equibiaxial	n.a.	n.a.	"young cells" (P5-7): increase (RIA)<br>"aged cells" (P19-22): increase (RIA)	"young cells" @ 18%: 5.9 (ng/10^6 cells) / 5.6 (ratio-calc)<br>"aged cells" @ 18%: 11.6 (ng/10^6 cells) / 9.4 (ratio-calc)
Ohzeki et al. (1999)	PGE_2	PGE_2	hPDL cells (12/M, 10/M, 11/F , PM, exp, P5-7 and P19-22, Confluent)	dynamic	**0.1Hz** (6cyc/min: 5s elongation and 5s relaxation) for 1d, 3d, 5d	18%	Flexercell strain unit (Shimizu et al 1994) + flexible-bottom culture plates coated with type I collagen (Flexcell) + vacuum	equibiaxial	n.a.	n.a.	"young cells" (P5-7): increase (RIA)<br>"aged cells" (P19-22): increase (RIA)	"young cells" @ 5d: 5.9 (ng/10^6 cells) / 11.2 (ratio-calc)<br>"aged cells" @ 5d: 12.1 (ng/10^6 cells) / 17.3 (ratio-calc)
Ohzeki et al. (1999)	PGE_2	PGE_2	hPDL cells (12/M, 10/M, 11/F , PM, exp, P5-7 and P19-22, Confluent)	dynamic	**0.1Hz** (6cyc/min: 5s elongation and 5s relaxation) for 5d	18%	Flexercell strain unit (Shimizu et al 1994) + flexible-bottom culture plates coated with type I collagen (Flexcell) + vacuum	equibiaxial	n.a.	n.a.	increase (RIA)	"young cells" (P5-7) (donor 1): 8.3 (ng/10^6 cells) / 13.2 (ratio-calc)<br>"aged cells" (P19-22) (donor 1): 12.0 (ng/10^6 cells) / 24 (ratio-calc)<br>"young cells" (P5-7) (donor 1): 2.5 (ug/mg protein) / 8.1 (ratio-calc)<br>"aged cells" (P19-22) (donor 1): 2.9 (ug/mg protein) / 11.6 (ratio-calc)<br>"young cells" (P5-7) (donor 2): 9.3 (ng/10^6 cells) / 18.6 (ratio-calc)<br>"aged cells" (P19-22) (donor 2): 14.0 (ng/10^6 cells) / 18.7 (ratio-calc)<br>"young cells" (P5-7) (donor 2): 2.6 (ug/mg protein) / 8.4 (ratio-calc)<br>"aged cells" (P19-22) (donor 2): 3.2 (ug/mg protein) / 12.8 (ratio-calc)<br>"young cells" (P5-7) (donor 3): 9.3 (ng/10^6 cells) / 14.8 (ratio-calc)<br>"aged cells" (P19-22) (donor 3): 12.0 (ng/10^6 cells) / 19.0 (ratio-calc)<br>"young cells" (P5-7) (donor 3): 2.5 (ug/mg protein) / 8.2 (ratio-calc)<br>"aged cells" (P19-22) (donor 3): 3.0 (ug/mg protein) / 10.7 (ratio-calc)
Ozawa et al. (1997)	PA	*PLAT; PLAU*	hPDL cells (n.g./n.g., n.g., exp, P n.g., Confluent)	dynamic	**0.1Hz** (6cyc/min: 5s elongation and 5s relaxation) sqPCR for 3d; Photometry for 1d, 3d, 5d	18%	Flexercell strain unit (Banes et al 1985) + flexible-bottom culture plates coated with type I collagen (Flexcell) + vacuum	equibiaxial	n.a.	n.a.	increase (PA activity; photometric)	5d: 8.1 (mU/10^5 cells) / 1.9 (ratio-calc)
Ozawa et al. (1997)	PAI-1	*SERPINE1*	hPDL cells (n.g./n.g., n.g., exp, P n.g., Confluent)	dynamic	**0.1Hz** (6cyc/min: 5s elongation and 5s relaxation) sqPCR for 3d; ELISA for 1d, 3d, 5d	18%	Flexercell strain unit (Banes et al 1985) + flexible-bottom culture plates coated with type I collagen (Flexcell) + vacuum	equibiaxial	no change (sqPCR, GAPDH)	no quantitative information is given	no change (ELISA)	
Ozawa et al. (1997)	tPA	*PLAT*	hPDL cells (n.g./n.g., n.g., exp, P n.g., Confluent)	dynamic	**0.1Hz** (6cyc/min: 5s elongation and 5s relaxation) sqPCR for 3d; Photometry for 1d, 3d, 5d	18%	Flexercell strain unit (Banes et al 1985) + flexible-bottom culture plates coated with type I collagen (Flexcell) + vacuum	equibiaxial	increase (sqPCR, GAPDH)	no quantitative information is given	n.g.	n.g.
Padial-Molina et al. (2013)	POSTN	*POSTN*	hPDL cells (35/F, 29/M, PM, n.g., P4-7, 2.5×10^5)	dynamic	**0.1Hz** (6cyc/min) for 24h, 4d, 7d	14%	Flexcell FX-5000 Tension System + BioFlex Culture Plates coated with Collagen I + vacuum	equibiaxial	no change (sqPCR, GAPDH)		no change (WB)	

[a] Entry given as reported in the study.
[b] All official gene symbols come from the HUGO Gene Nomenclature Committee (HGNC; URL: https://www.genenames.org) after checking specificity of primers with Primer-BLAST.
[c] Gender/Sex of donors: "M" – male, "F" – female; Tooth type: "PM" – premolar, "M" – molar; Cell density: given in cells/well if not otherwise mentioned.
[d] Frequencies labeled bold orange were converted to hertz (Hz) according to its definition using the information reported in the study (in brackets)
[e] Force type deduced from the description of the force apparatus given by the authors.
[f] Gene and protein expression: 1. conclusion of change (increase, decrease…) was given according to the defined criteria in Figure 2; 2. different markers to describe the amount of change; † Information derived from figures using Engauge Digitizer; *Folds calculated by measuring the graphs, without using the Engauge Digitizer; No makers: Information derived from figures by description in the articles

Reference	Gene/ Analyte[a]	Official gene symbol / abbreviation[b]	Cell (age/gender of donors, tooth type, isolation method, passages used, cell density)[a,c]	Force type (stat./ dyn.)[a]	Force duration and frequency[d]	Force magnitude[a]	Force apparatus[a]	Force type: equibiaxial or uniaxial[e]	Gene expression: Increase, decrease, no change (method w/ reference gene); Methods: qPCR, sqPCR, Northern blot[f]	Gene expression: When it reaches peak and peak's magnitude (fold change; times or ratio; unclear = ?)[f]	Protein expression: Increase, decrease, no change (method w/ reference); Methods: ELISA, WB, RIA, EMSA, IF[f]	Protein expression: When it reaches peak and peak's magnitude (times or ratio; unclear = ?)[f]
Padial-Molina et al. (2013)	βIGH3	*TGFBI*	hPDL cells (35/F, 29/M, PM, n.g., P4-7, 2.5×10^5)	dynamic	**0.1Hz** (6cyc/min) for 24h, 4d, 7d	14%	Flexcell FX-5000 Tension System + BioFlex Culture Plates coated with Collagen I + vacuum	equibiaxial	no change (sqPCR, GAPDH)		no change (WB)	
Pan et al. (2014)	Cofilin/p-Cofilin	*CFL1*	hPDL cells (n.g./n.g., n.g., dig, P4-8, 95% confluence)	dynamic	0.1Hz for 6h, 24h	10%	Flexercell Tension Plus system FX-5000T + collagen I-coated 6-well Bioflex plates + vacuum	equibiaxial	n.g.	n.g.	cofilin: no change (WB, β-actin)<br>p-cofilin: increase (WB, β-actin)	p-cofilin: 24h: 2.5 (ratio)*
Pan et al. (2014)	RhoA / GTP-RhoA	*RHOA*	hPDL cells (n.g./n.g., n.g., dig, P4-8, 95% confluence)	dynamic	0.1Hz for 6h, 24h	10%	Flexercell Tension Plus system FX-5000T + collagen I-coated 6-well Bioflex plates + vacuum	equibiaxial	n.g.	n.g.	RhoA: no change (WB, β-actin)<br>GTP-RhoA: increase (WB, β-actin)	GTP-RhoA: 24h: 1.7 (ratio)*
Pan et al. (2014)	Rho-GDIα	*ARHGDIA*	hPDL cells (n.g./n.g., n.g., dig, P4-8, 95% confluence)	dynamic	0.1Hz for 6h, 24h	10%	Flexercell Tension Plus system FX-5000T + collagen I-coated 6-well Bioflex plates + vacuum	equibiaxial	n.g.	n.g.	decrease (WB, β-actin)	24h: 0.5 (ratio)*
Pan et al. (2014)	ROCK	*ROCK1; ROCK2*	hPDL cells (n.g./n.g., n.g., dig, P4-8, 95% confluence)	dynamic	0.1Hz for 6h, 24h	10%	Flexercell Tension Plus system FX-5000T + collagen I-coated 6-well Bioflex plates + vacuum	equibiaxial	n.g.	n.g.	increase (WB, β-actin)	ROCK: 24h: 2.4 (ratio)*
Papadopoulou et al. (2017)	c-fos	*FOS*	hPDL cells (3 donors: 9-20/n.g., n.g., exp, P3-6, n.g.)	static	15min, 30min, 60min, 180min	8%	in-house designed device prepared by Controla (Advanced Technology Equipment, Athens, Greece) + silicone dishes + moving clamp	uniaxial	temporary increase (qPCR, GAPDH)	donor A @ 30min: 4.0 ((FC)†<br>donor B @ 30min: 6.7 (FC)†<br>donor C @ 1h: 4.2 (FC)†	n.g.	n.g.
Papadopoulou et al. (2017)	c-fos	*FOS*	hPDL cells (3 donors: 9-20/n.g., n.g., exp, P3-6, n.g.)	dynamic	1Hz for 15min, 30min, 60min, 180min	8%	six station stretching appratus + optically transparent silicone dishes pre-coated with fibronectin + moving clamp (Konstantonis et al 2014; further reference to Neidlinger-Wilke et al 2001)	uniaxial	temporary increase (qPCR, GAPDH)	donor A @ 1h: 8.3 ((FC)†<br>donor B @ 1h: 10.6 (FC)†<br>donor C @ 1h: 10.9 (FC)†	n.g.	n.g.
Papadopoulou et al. (2017)	c-jun	*JUN*	hPDL cells (3 donors: 9-20/n.g., n.g., exp, P3-6, n.g.)	static	15min, 30min, 60min, 180min	8%	in-house designed device prepared by Controla (Advanced Technology Equipment, Athens, Greece). + silicone dishes + moving clamp	uniaxial	temporary increase (qPCR, GAPDH)	donor A @ 1h: 1.6 (FC)†<br>donor B @ 1h: 1.7 (FC)†<br>donor C @ 1h: 1.7 (FC)†	n.g.	n.g.
Papadopoulou et al. (2017)	c-jun	*JUN*	hPDL cells (3 donors: 9-20/n.g., n.g., exp, P3-6, n.g.)	dynamic	1Hz for 15min, 30min, 60min, 180min	8%	six station stretching appratus + optically transparent silicone dishes pre-coated with fibronectin + moving clamp (Konstantonis et al 2014; further reference to Neidlinger-Wilke et al 2001)	uniaxial	temporary increase (qPCR, GAPDH)	donor A @ 1h: 2.3 (FC)†<br>donor B @ 1h: 1.7 (FC)†<br>donor C @ 1h: 4.2 (FC)†	n.g.	n.g.
Papadopoulou et al. (2017)	ERK (pan ERK) / p-ERK1/2	MAPK3; MAPK1	hPDL cells (3 donors: 9-20/n.g., n.g., exp, P3-6, n.g.)	static	15min, 30min, 60min, 180min	8%	in-house designed device prepared by Controla (Advanced Technology Equipment, Athens, Greece). + silicone dishes + moving clamp	uniaxial	n.g.	n.g.	ERK1/2: no change (WB, actin)<br>p-ERK1/2: temporary increase (WB, actin)	ERK1/2: no quantitative information is given<br>p-ERK1/2: no quantitative information is given
Papadopoulou et al. (2017)	ERK (pan ERK) / p-ERK1/2	MAPK3; MAPK1	hPDL cells (3 donors: 9-20/n.g., n.g., exp, P3-6, n.g.)	dynamic	1Hz for 15min, 30min, 60min, 180min	8%	six station stretching appratus + optically transparent silicone dishes pre-coated with fibronectin + moving clamp (Konstantonis et al 2014; further reference to Neidlinger-Wilke et al 2001)	uniaxial	n.g.	n.g.	ERK: no change (WB, actin)<br>p-ERK1/2: temporary increase (WB, actin)	ERK: no quantitative information is given<br>p-ERK1/2: no quantitative information is given
Papadopoulou et al. (2017)	JNKs / p-JNKs	*MAPK8; MAPK9; MAPK10*	hPDL cells (3 donors: 9-20/n.g., n.g., exp, P3-6, n.g.)	static	15min, 30min, 60min, 180min	8%	in-house designed device prepared by Controla (Advanced Technology Equipment, Athens, Greece) + silicone dishes + moving clamp	uniaxial	n.g.	n.g.	JNKs: no change (WB, actin)<br>p-JNKs: temporary increase (WB, actin)	JNKs: no quantitative information is given<br>p-JNKs: no quantitative information is given
Papadopoulou et al. (2017)	JNKs / p-JNKs	*MAPK8; MAPK9; MAPK10*	hPDL cells (3 donors: 9-20/n.g., n.g., exp, P3-6, n.g.)	dynamic	1Hz for 15min, 30min, 60min, 180min	8%	six station stretching appratus + optically transparent silicone dishes pre-coated with fibronectin + moving clamp (Konstantonis et al 2014; further reference to Neidlinger-Wilke et al 2001)	uniaxial	n.g.	n.g.	JNKs: no change (WB, actin)<br>p-JNKs: temporary increase (WB, actin)	JNKs: no quantitative information is given<br>p-JNKs: no quantitative information is given
Papadopoulou et al. (2017)	p38 MAPK / p-p38$^{(Thr180/Tyr182)}$	*MAPK14*	hPDL cells (3 donors: 9-20/n.g., n.g., exp, P3-6, n.g.)	static	15min, 30min, 60min, 180min	8%	in-house designed device prepared by Controla (Advanced Technology Equipment, Athens, Greece). + silicone dishes + moving clamp	uniaxial	n.g.	n.g.	p-p38: temporary increase (WB, actin)<br>p38: no change (WB, actin)	p-p38: no quantitative information is given<br>p38: no quantitative information is given
Papadopoulou et al. (2017)	p38 MAPK / p-p38$^{(Thr180/Tyr182)}$	*MAPK14*	hPDL cells (3 donors: 9-20/n.g., n.g., exp, P3-6, n.g.)	dynamic	1Hz for 15min, 30min, 60min, 180min	8%	six station stretching appratus + optically transparent silicone dishes pre-coated with fibronectin + moving clamp (Konstantonis et al 2014; further reference to Neidlinger-Wilke et al 2001)	uniaxial	n.g.	n.g.	p-p38: temporary increase (WB, actin)<br>p38: no change (WB, actin)	p-p38: no quantitative information is given<br>p38: no quantitative information is given
Papadopoulou et al. (2019)	ALP	*ALPP*	hPDLF (n.g./n.g., n.g., exp, P n.g., n.g.)	dynamic	1Hz for 18h	8%	six station stretching apparatus + optically transparent silicone dishes pre-coated with fibronectin + moving clamp (Neidlinger-Wilke et al 2001)	uniaxial	increase (qPCR, GAPDH)	2.3 (FC)†	n.g.	n.g.
Papadopoulou et al. (2019)	c-fos	*FOS*	hPDLF (n.g./n.g., n.g., exp, P n.g., n.g.)	dynamic	1Hz for 0.5h	8%	six station stretching apparatus + optically transparent silicone dishes pre-coated with fibronectin + moving clamp (Neidlinger-Wilke et al 2001)	uniaxial	increase (qPCR, GAPDH)	4.2 (FC)†	n.g.	n.g.
Papadopoulou et al. (2019)	JNK / p-JNK$^{(Thr183/Tyr185)}$	*MAPK8*	hPDLF (n.g./n.g., n.g., exp, P n.g., n.g.)	dynamic	1Hz for 0.33h (20min)	8%	six station stretching apparatus + optically transparent silicone dishes pre-coated with fibronectin + moving clamp (Neidlinger-Wilke et al 2001)	uniaxial	n.g.	n.g.	JNK: no change (WB, GAPDH)<br>p-JNK$^{(Thr183/Tyr185)}$: increase (WB, GAPDH)	no quantitative information is given
Papadopoulou et al. (2019)	OPN	*SPP1*	hPDLF (n.g./n.g., n.g., exp, P n.g., n.g.)	dynamic	1Hz for 18h	8%	six station stretching apparatus + optically transparent silicone dishes pre-coated with fibronectin + moving clamp (Neidlinger-Wilke et al 2001)	uniaxial	increase (qPCR, GAPDH)	1.6 (FC)†	n.g.	n.g.

[a] Entry given as reported in the study.
[b] All official gene symbols come from the HUGO Gene Nomenclature Committee (HGNC; URL: https://www.genenames.org) after checking specificity of primers with Primer-BLAST.
[c] Gender/Sex of donors: "M" – male, "F" – female; Tooth type: "PM" – premolar, "M" – molar; Cell density: given in cells/well if not otherwise mentioned.
[d] Frequencies labeled bold orange were converted to hertz (Hz) according to its definition using the information reported in the study (in brackets)
[e] Force type deduced from the description of the force apparatus given by the authors.
[f] Gene and protein expression: 1. conclusion of change (increase, decrease…) was given according to the defined criteria in Figure 2; 2. different markers to describe the amount of change; † Information derived from figures using Engauge Digitizer; *Folds calculated by measuring the graphs, without using the Engauge Digitizer; No makers: Information derived from figures by description in the articles

Reference	Gene/ Analyte[a]	Official gene symbol / abbreviation[b]	Cell (age/gender of donors, tooth type, isolation method, passages used, cell density)[a,c]	Force type (stat./ dyn.)[a]	Force duration and frequency[d]	Force magnitude[a]	Force apparatus[a]	Force type: equibiaxial or uniaxial[e]	Gene expression: Increase, decrease, no change (method w/ reference gene); Methods: qPCR, sqPCR, Northern blot[f]	Gene expression: When it reaches peak and peak's magnitude (fold change; times or ratio; unclear = ?)[f]	Protein expression: Increase, decrease, no change (method w/ reference); Methods: ELISA, WB, RIA, EMSA, IF[f]	Protein expression: When it reaches peak and peak's magnitude (times or ratio; unclear = ?)[f]
Papadopoulou et al. (2019)	p38 / p-p38$^{(Thr180/Tyr182)}$	*MAPK14*	hPDLF (n.g./n.g., n.g., exp, P n.g., n.g.)	dynamic	1Hz for 0.33h (20min)	8%	six station stretching apparatus + optically transparent silicone dishes pre-coated with fibronectin + moving clamp (Neidlinger-Wilke et al 2001)	uniaxial	n.g.	n.g.	p38: no change (WB, GAPDH)<br>p38$^{(Thr180/Tyr182)}$: increase (WB, GAPDH)	no quantitative information is given
Papadopoulou et al. (2019)	pan ERK / p-ERK1/2$^{(Thr202/Tyr204)}$	*MAPK3;*<br>*MAPK1*	hPDLF (n.g./n.g., n.g., exp, P n.g., n.g.)	dynamic	1Hz for 0.33h (20min)	8%	six station stretching apparatus + optically transparent silicone dishes pre-coated with fibronectin + moving clamp (Neidlinger-Wilke et al 2001)	uniaxial	n.g.	n.g.	pan ERK: no change (WB, GAPDH)<br>p-ERK1/2$^{(Thr202/Tyr204)}$: increase (WB, GAPDH)	no quantitative information is given
Pelaez et al. (2017)	ARRAY	ARRAY	hPDLSC cells (n.g./n.g., M, dig, P2-3, 85–90 % confluent)	dynamic	0.5Hz for 2h	5%	"custom-built bioreactor system" + chambers + linear actuator	uniaxial	SurePrint G3 Human v.16 miRNA Array Kit (8x60K, Release 16.0, Agilent)			
Pelaez et al. (2017)	Cx43	*GJA1*	hPDLSC cells (n.g./n.g., M, dig, P2-3, 85–90 % confluent)	dynamic	0.5Hz for 2h	5%	"custom-built bioreactor system" + chambers + linear actuator	uniaxial	increase (qPCR, GAPDH)	2.0 (rel)* / 2.0 (ratio-calc)	n.g.	n.g.
Pelaez et al. (2017)	GATA4	*GATA4*	hPDLSC cells (n.g./n.g., M, dig, P2-3, 85–90 % confluent)	dynamic	0.5Hz for 2h	5%	"custom-built bioreactor system" + chambers + linear actuator	uniaxial	increase (qPCR, GAPDH)	1.9 (rel)* / 3.3 (ratio-calc)	n.g.	n.g.
Pelaez et al. (2017)	MEF2C	*MEF2C*	hPDLSC cells (n.g./n.g., M, dig, P2-3, 85–90 % confluent)	dynamic	0.5Hz for 2h	5%	"custom-built bioreactor system" + chambers + linear actuator	uniaxial	increase (qPCR, GAPDH)	3.6 (rel)* / 20.0 (ratio-calc)	n.g.	n.g.
Pelaez et al. (2017)	MYH7	*MYH7*	hPDLSC cells (n.g./n.g., M, dig, P2-3, 85–90 % confluent)	dynamic	0.5Hz for 2h	5%	"custom-built bioreactor system" + chambers + linear actuator	uniaxial	increase (qPCR, GAPDH)	15.7 (rel)* / 16.5 (ratio-calc)	n.g.	n.g.
Pelaez et al. (2017)	MYL2	*MYL2*	hPDLSC cells (n.g./n.g., M, dig, P2-3, 85–90 % confluent)	dynamic	0.5Hz for 2h	5%	"custom-built bioreactor system" + chambers + linear actuator	uniaxial	increase (qPCR, GAPDH)	1.9 (rel)* / 2.0 (ratio-calc)	n.g.	n.g.
Pelaez et al. (2017)	MYL7	*MYL7*	hPDLSC cells (n.g./n.g., M, dig, P2-3, 85–90 % confluent)	dynamic	0.5Hz for 2h	5%	"custom-built bioreactor system" + chambers + linear actuator	uniaxial	increase (qPCR, GAPDH)	16.2 (rel)* / 17.1 (ratio-calc)	n.g.	n.g.
Pelaez et al. (2017)	Nitric oxide	Nitric oxide	hPDLSC cells (n.g./n.g., M, dig, P2-3, 85–90 % confluent)	dynamic	0.5Hz for 2h	5%	"custom-built bioreactor system" + chambers + linear actuator	uniaxial	n.a.	n.a.	increase (photometric)	20 min: 1.048 (relative NO levels)* / 1.07 (ratio-calc)
Pelaez et al. (2017)	Nkx2.5	*NKX2-5*	hPDLSC cells (n.g./n.g., M, dig, P2-3, 85–90 % confluent)	dynamic	0.5Hz for 2h	5%	"custom-built bioreactor system" + chambers + linear actuator	uniaxial	increase (qPCR, GAPDH)	5.1 (rel)* / 2 (ratio-calc)	n.g.	n.g.
Pelaez et al. (2017)	NPPA	*NPPA*	hPDLSC cells (n.g./n.g., M, dig, P2-3, 85–90 % confluent)	dynamic	0.5Hz for 2h	5%	"custom-built bioreactor system" + chambers + linear actuator	uniaxial	increase (qPCR, GAPDH)	3.6 (rel)* / 1.9 (ratio-calc)	n.g.	n.g.
Pelaez et al. (2017)	NPPB	*NPPB*	hPDLSC cells (n.g./n.g., M, dig, P2-3, 85–90 % confluent)	dynamic	0.5Hz for 2h	5%	"custom-built bioreactor system" + chambers + linear actuator	uniaxial	increase (qPCR, GAPDH)	1.2 (rel)* / 2.4 (ratio-calc)	n.g.	n.g.
Pelaez et al. (2017)	TNNT2	*TNNT2*	hPDLSC cells (n.g./n.g., M, dig, P2-3, 85–90 % confluent)	dynamic	0.5Hz for 2h	5%	"custom-built bioreactor system" + chambers + linear actuator	uniaxial	increase (qPCR, GAPDH)	2.1 (rel)* / 1.3 (ratio-calc)	n.g.	n.g.
Pelaez et al. (2017)	TPM1	*TPM1*	hPDLSC cells (n.g./n.g., M, dig, P2-3, 85–90 % confluent)	dynamic	0.5Hz for 2h	5%	"custom-built bioreactor system" + chambers + linear actuator	uniaxial	increase (qPCR, GAPDH)	2.5 (rel)* / 6.8 (ratio-calc)	n.g.	n.g.
Peverali et al. (2001)	AP-1	not clear	hPDL fibroblasts (n.g./n.g., n.g., exp, P3-6, 80% confluency)	static	15min, 30min	2.5%	Petriperm dish + template with a convex surface + weight	equibiaxial	n.g.	n.g.	increase (EMSA)	no quantitative information is given
Peverali et al. (2001)	C-FOS	*FOS*	hPDL fibroblasts (n.g./n.g., n.g., exp, P3-6, 80% confluency)	static	15min, 30min	2.5%	Petriperm dish + template with a convex surface + weight	equibiaxial	n.g.	n.g.	increase (in-gel kinase assay)	no quantitative information is given
Peverali et al. (2001)	C-JUN	*JUN*	hPDL fibroblasts (n.g./n.g., n.g., exp, P3-6, 80% confluency)	static	7min, 15min, 30min	2.5%	Petriperm dish + template with a convex surface + weight	equibiaxial	n.g.	n.g.	increase (in-gel kinase assay)	no quantitative information is given
Pinkerton et al. (2008)	ARRAY	ARRAY	hPDL cells (n.g./n.g., PM, exp, P4, 3×10^5)	dynamic	**0.01Hz** (Strain for 6s every 90s) for 6h, 12h, 24h	12%	Flexercell FX-4000 Strain Unit + 6-well, 35 mm flexible-bottomed Uniflex culture plates with a centrally located, rectangular type I collagen-coated culture strip (15.25 mm×24.18 mm) + vacuum	uniaxial	RT^2 Profiler PCR Array System (Superarray Bioscience Corp.) testing the expression of 79 genes encoding common cytokines	ARRAY	n.g.	n.g.
Qin and Hua (2016)	ALP	*ALPP*	hPDL cells (12-14/n.g., PM, n.g., P3-8, 1×10^6 cells/ml)	dynamic	0.5Hz (2s) for 1h, 3h, 6h	5%	n.g.	n.g.	not reported with reference to force (qPCR, GAPDH)		temporary decrease (WB, GAPDH)	1h: 0.03 (rel)* / 0.2 (ratio-calc)
Qin and Hua (2016)	Col-1	*COL1A1*	hPDL cells (12-14/n.g., PM, n.g., P3-8, 1×10^6 cells/ml)	dynamic	0.5Hz (2s) for 1h, 3h, 6h	5%	n.g.	n.g.	not reported with reference to force (qPCR, GAPDH)		increase (WB, GAPDH)	3h: 0.16 (rel)* / 3.2 (ratio-calc)
Qin and Hua (2016)	OCN	*BGLAP*	hPDL cells (12-14/n.g., PM, n.g., P3-8, 1×10^6 cells/ml)	dynamic	0.5Hz (2s) for 1h, 3h, 6h	5%	n.g.	n.g.	not reported with reference to force (qPCR, GAPDH)		temporary decrease followed by temporary increase (WB, GAPDH)	lowest @ 1h: 0.09 (rel)* / 0.7 (ratio-calc)<br>highest @ 3h: 0.2 (rel)* / 1.5 (ratio-calc)
Rath-Deschner et al. (2009)	IGF1	*IGF1*	hPDL cells (n.g./n.g., n.g., exp, P3-5, 80% confluency)	static	qPCR for 4h, 24h; WB for 24h	3%, 20%	loading platform with cylindrical posts + collagen type I-coated BioFlex plates (Flexcells) + screws (Deschner et al 2007)	equibiaxial	3%: temporary increase (qPCR, GAPDH)<br>20%: decrease followed by plateau (qPCR, GAPDH)	3% @ 4h: 171.69% (rel) / 1.7 (ratio-calc)<br>20% @ 4h...24h: 54.42% (rel) / 0.5 (ratio-calc)	3%: increase (WB, β-actin)<br>20%: no change (WB, β-actin)	no quantitative information is given
Rath-Deschner et al. (2009)	IGF1R	*IGF1R*	hPDL cells (n.g./n.g., n.g., exp, P3-5, 80% confluency)	static	4h, 24h	3%, 20%	loading platform with cylindrical posts + collagen type I-coated BioFlexs plates (Flexcells) + screws (Deschner et al 2007)	equibiaxial	3%: increase followed by plateau (qPCR, GAPDH)<br>20%: decrease (qPCR, GAPDH)	3% @ 4h: 115.72% (rel) / 1.2 (ratio-calc)<br>20% @ 24h: 82.97% (rel) / 0.8 (ratio-calc)	n.g.	n.g.
Rath-Deschner et al. (2009)	IGF2	*IGF2*	hPDL cells (n.g./n.g., n.g., exp, P3-5, 80% confluency)	static	4h, 24h	3%, 20%	loading platform with cylindrical posts + collagen type I-coated BioFlexs plates (Flexcells) + screws (Deschner et al 2007)	equibiaxial	3%: increase followed by plateau (qPCR, GAPDH)<br>20%: decrease followed by plateau (qPCR, GAPDH)	3% @ 4h: 112.49% (rel) / 1.1 (ratio-calc)<br>20% @ 4h...24h: 82.97% (rel) / 0.8 (ratio-calc)	n.g.	n.g.
Rath-Deschner et al. (2009)	IGFBP1	*IGFBP1*	hPDL cells (n.g./n.g., n.g., exp, P3-5, 80% confluency)	static	qPCR for 4h, 24h; WB for 24h	3%, 20%	loading platform with cylindrical posts + collagen type I-coated BioFlexs plates (Flexcells) + screws (Deschner et al 2007)	equibiaxial	3%: decrease followed by increase (qPCR, GAPDH)<br>20%: increase (qPCR, GAPDH)	3% lowest @ 4h: 65.99% (rel) / 0.7 (ratio-calc)<br>3% highest @ 24h: 144.50% (rel) / 1.4 (ratio-calc)<br>20% @ 24h: 291.49% (rel) / 2.9 (ratio-calc)	3%: no change (WB, β-actin)<br>20%: increase (WB, β-actin)	no quantitative information is given

[a] Entry given as reported in the study.
[b] All official gene symbols come from the HUGO Gene Nomenclature Committee (HGNC; URL: https://www.genenames.org) after checking specificity of primers with Primer-BLAST.
[c] Gender/Sex of donors: "M" – male, "F" – female; Tooth type: "PM" – premolar, "M" – molar; Cell density: given in cells/well if not otherwise mentioned.
[d] Frequencies labeled bold orange were converted to hertz (Hz) according to its definition using the information reported in the study (in brackets)
[e] Force type deduced from the description of the force apparatus given by the authors.
[f] Gene and protein expression: 1. conclusion of change (increase, decrease…) was given according to the defined criteria in Figure 2; 2. different markers to describe the amount of change; † Information derived from figures using Engauge Digitizer; *Folds calculated by measuring the graphs, without using the Engauge Digitizer; No makers: Information derived from figures by description in the articles

Reference	Gene/ Analyte[a]	Official gene symbol / abbreviation[b]	Cell (age/gender of donors, tooth type, isolation method, passages used, cell density)[a,c]	Force type (stat./ dyn.)[a]	Force duration and frequency[d]	Force magnitude[a]	Force apparatus[a]	Force type: equibiaxial or uniaxial[e]	Gene expression: Increase, decrease, no change (method w/ reference gene); Methods: qPCR, sqPCR, Northern blot[f]	Gene expression: When it reaches peak and peak's magnitude (fold change; times or ratio; unclear = ?)[f]	Protein expression: Increase, decrease, no change (method w/ reference); Methods: ELISA, WB, RIA, EMSA, IF[f]	Protein expression: When it reaches peak and peak's magnitude (times or ratio; unclear = ?)[f]
Rath-Deschner et al. (2009)	IGFBP3	*IGFBP3*	hPDL cells (n.g./n.g., n.g., exp, P3-5, 80% confluency)	static	qPCR for 4h, 24h; WB for 24h	3%, 20%	loading platform with cylindrical posts + collagen type I-coated BioFlexs plates (Flexcells) + screws (Deschner et al 2007)	equibiaxial	3%: decrease (qPCR, GAPDH)<br>20%: increase followed by decrease (qPCR, GAPDH)	3% @ 24h: 84.33% (rel) / 0.8 (ratio-calc)<br>20% @ highest at 4h:158.27% (rel) / 1.6 (ratio-calc)<br>20% lowest at 24h:75.80% (rel) / 0.8 (ratio-calc)	3%: no change (WB, β-actin)<br>20%: increase (WB, β-actin)	no quantitative information is given
Rath-Deschner et al. (2009)	IGFBP5	*IGFBP5*	hPDL cells (n.g./n.g., n.g., exp, P3-5, 80% confluency)	static	qPCR for 4h, 24h; WB for 24h	3%, 20%	loading platform with cylindrical posts + collagen type I-coated BioFlexs plates (Flexcells) + screws (Deschner et al 2007)	equibiaxial	3%: increase (qPCR, GAPDH)<br>20%: increase followed by decrease (qPCR, GAPDH)	3% @ 4h: 136.06% (rel) / 1.4 (ratio-calc)<br>20% @ highest at 4h:127.34% (rel) / 1.3 (ratio-calc)<br>20% @ lowest at 24h: 69.00% (rel) / 0.7 (ratio-calc)	3%: increase (WB, β-actin)<br>20%: no change (WB, β-actin)	no quantitative information is given
Rath-Deschner et al. (2009)	IRS1	*IRS1*	hPDL cells (n.g./n.g., n.g., exp, P3-5, 80% confluency)	static	4h, 24h	3%, 20%	loading platform with cylindrical posts + collagen type I-coated BioFlexs plates (Flexcells) + screws (Deschner et al 2007)	equibiaxial	3%: temporary decrease (qPCR, GAPDH)<br>20%: increase followed by decrease (qPCR, GAPDH)	3% @ 4h: 82.46% (rel) / 0.8 (ratio-calc)<br>20% highest at 4h: 112.39% (rel) / 1.1 (ratio-calc)<br>20% lowest at 24h: 86.49% (rel) / 0.9 (ratio-calc)	n.g.	n.g.
Rath-Deschner et al. (2009)	PCNA	*PCNA*	hPDL cells (n.g./n.g., n.g., exp, P3-5, 80% confluency)	static	4h, 24h, 48h	3%, 20%	loading platform with cylindrical posts + collagen type I-coated BioFlexs plates (Flexcells) + screws (Deschner et al 2007)	equibiaxial	3%: increase (qPCR, GAPDH)<br>20%: increase (qPCR, GAPDH)	3% @ 48h: 114.97% (rel) / 1.1 (ratio-calc)<br>20% @ 48h: 146.32% (rel) / 1.5 (ratio-calc)	n.g.	n.g.
Ren et al. (2015)	ATF4	*ATF4*	hPDL cells (12-18, PM, exp, P4-6, 80% confluency)	dynamic	0.5Hz for 1h, 3h, 6h, 12h, 18h, 24h	10%	Flexercell FX-4000 Strain Unit + six-well 35mm silicone membrane culture plates coated with type I collagen + vacuum	equibiaxial	increase (qPCR, GAPDH)	1h: 1.7 (FC)*	n.g.	n.g.
Ren et al. (2015)	BSP	*IBSP*	hPDL cells (12-18, PM, exp, P4-6, 80% confluency)	dynamic	0.5Hz for 1h, 3h, 6h, 12h, 18h, 24h	10%	Flexercell FX-4000 Strain Unit + six-well 35mm silicone membrane culture plates coated with type I collagen + vacuum	equibiaxial	increase (qPCR, GAPDH)	24h: 3.2 (FC)*	n.g.	n.g.
Ren et al. (2015)	ERK1/2 / p-ERK1/2	MAPK3; MAPK1	hPDL cells (12-18, PM, exp, P4-6, 80% confluency)	dynamic	0.5Hz for 1h, 3h, 6h, 12h, 18h, 24h	10%	Flexercell FX-4000 Strain Unit + six-well 35mm silicone membrane culture plates coated with type I collagen + vacuum	equibiaxial	n.g.	n.g.	ERK1/2: no change (WB, GAPDH)<br>p-ERK1/2: increase (WB, GAPDH)	ERK1/2: no quantitative information is given<br>p-ERK1/2: 3h: 0.7 (rel)* / 6.2 (ratio-calc)
Ren et al. (2015)	OCN	*BGLAP*	hPDL cells (12-18, PM, exp, P4-6, 80% confluency)	dynamic	0.5Hz for 1h, 3h, 6h, 12h, 18h, 24h	10%	Flexercell FX-4000 Strain Unit + six-well 35mm silicone membrane culture plates coated with type I collagen + vacuum	equibiaxial	increase followed by plateau (qPCR, GAPDH)	18…24h: 1.5 (FC)*	n.g.	n.g.
Ren et al. (2015)	RUNX2 / p-RUNX2	*RUNX2*	hPDL cells (12-18, PM, exp, P4-6, 80% confluency)	dynamic	0.5Hz for 1h, 3h, 6h, 12h, 18h, 24h	10%	Flexercell FX-4000 Strain Unit + six-well 35mm silicone membrane culture plates coated with type I collagen + vacuum	equibiaxial	RUNX2: increase (qPCR, GAPDH)	RUNX2 @ 3h: 2.9 (FC)*	RUNX2: temporary increase followed by plateau then temporary decrease (WB, GAPDH)<br>p-RUNX2: increase followed by plateau (WB, GAPDH)	RUNX2: highest @ 3h…6h: 0.7 (rel)† / 1.9 (ratio-calc)<br>RUNX2: lowest @12h: 0.2 (rel)† / 0.6 (ratio-calc)<br>p-RUNX2 @ 3h…6h: 0.4 (rel)* / 9.5 (ratio-calc)
Ren et al. (2015)	SP7	*SP7*	hPDL cells (12-18, PM, exp, P4-6, 80% confluency)	dynamic	0.5Hz for 1h, 3h, 6h, 12h, 18h, 24h	10%	Flexercell FX-4000 Strain Unit + six-well 35mm silicone membrane culture plates coated with type I collagen + vacuum	equibiaxial	increase (qPCR, GAPDH)	12h: 4.3 (FC)*	n.g.	n.g.
Ritter et al. (2007)	ARRAY	ARRAY	hPDL cells (12-14/n.g., PM, exp, P3-6, near-confluence)	static	6h	2.5%	Petriperm dish + template made of brass + weight (Saito et al 1991)	equibiaxial	apoptosis and NFκ-B pathway GEArray Q Series kit (SuperArray, Bethesda, Md.)	ARRAY		
Ritter et al. (2007)	BAD	*BAD*	hPDL cells (12-14/n.g., PM, exp, P3-6, near-confluence)	static	6h	2.5%	Petriperm dish + template made of brass + weight (Saito et al 1991)	equibiaxial	increase (qPCR, β-actin)	5.2 (FC)	n.g.	n.g.
Ritter et al. (2007)	CRADD	*CRADD*	hPDL cells (12-14/n.g., PM, exp, P3-6, near-confluence)	static	6h	2.5%	Petriperm dish + template made of brass + weight (Saito et al. 1991)	equibiaxial	increase (qPCR, β-actin)	2.1 (FC)	n.g.	n.g.
Ritter et al. (2007)	FAS	*FAS*	hPDL cells (12-14/n.g., PM, exp, P3-6, near-confluence)	static	6h	2.5%	Petriperm dish + template made of brass + weight (Saito et al. 1991)	equibiaxial	increase (qPCR, β-actin)	2.0 (FC)	n.g.	n.g.
Ritter et al. (2007)	IL1β	*IL1B*	hPDL cells (12-14/n.g., PM, exp, P3-6, near-confluence)	static	6h	2.5%	Petriperm dish + template made of brass + weight (Saito et al. 1991)	equibiaxial	increase (qPCR, β-actin)	5.8 (FC)	n.g.	n.g.
Ritter et al. (2007)	NFkB	*NFKB1*	hPDL cells (12-14/n.g., PM, exp, P3-6, near-confluence)	static	6h	2.5%	Petriperm dish + template made of brass + weight (Saito et al. 1991)	equibiaxial	increase (qPCR, β-actin)	1.7 (FC)	n.g.	n.g.
Saminathan et al. (2012)	ARRAY	ARRAY	hPDL cells (n.g./n.g., n.g., exp, P3-4, confluence)	dynamic	**0.01Hz (1/95Hz)** (square waveform: 5s (0.2Hz) every 90s) for 6h, 12h, 24h	12%	Flexercell FX-4000 strain unit + six-well, 35 mm flexible-bottomed UniFlex® Series culture plates containing a centrally located rectangular strip (15.25×24.18 mm) coated with type I collagen + vacuum	uniaxial	extracellular matrix and adhesion molecules using the RT² Profiler PCR Array System (SABiosciences)	ARRAY	n.g.	n.g.
Saminathan et al. (2012)	Caspases 3/7 (combined assay)	*CASP3; CASP7*	hPDL cells (n.g./n.g., n.g., exp, P3-4, confluence)	dynamic	**0.01Hz (1/95Hz)** (square waveform: 5s (0.2Hz) every 90s) for 6h, 12h, 24h	12%	Flexercell FX-4000 strain unit + six-well, 35 mm flexible-bottomed UniFlex® Series culture plates containing a centrally located rectangular strip (15.25×24.18 mm) coated with type I collagen + vacuum	uniaxial	n.g.	n.g.	decrease (Caspase-Glo 3/7 Assay)	6h: 100.87 (RLU×10³) / 0.9 (ratio-calc)
Shen et al. (2014)	ALP	*ALPP*	hPDLSC cells (12-24/n.g., PM, dig, P4-6, 100% confluence)	dynamic	0.1Hz for 6h, 12h, 24h	12%	Flexcell FX-4000T Tension Plus System + 6-well, flexible-bottomed culture plate coated with type I collagen (Sigma)+ vacuum	equibiaxial	increase (qPCR, β-actin)	24h: 13.8 (rel)* / 2.5 (ratio-calc)	increase (WB, GAPDH)	24h: 347.2 (rel)* / 1.5 (ratio-calc)
Shen et al. (2014)	CD146	*MCAM*	hPDLSC cells (12-24/n.g., PM, dig, P4-6, 100% confluence)	dynamic	0.1Hz for 6h, 12h, 24h	12%	Flexcell FX-4000T Tension Plus System + 6-well, flexible-bottomed culture plate coated with type I collagen (Sigma)+ vacuum	equibiaxial	decrease (qPCR, β-actin)	24h: 1.2 (rel)* / 0.4 (ratio-calc)	decrease (WB, GAPDH)	24h: 158.5 (rel) * / 0.8 (ratio-calc)

[a] Entry given as reported in the study.
[b] All official gene symbols come from the HUGO Gene Nomenclature Committee (HGNC; URL: https://www.genenames.org) after checking specificity of primers with Primer-BLAST.
[c] Gender/Sex of donors: "M" – male, "F" – female; Tooth type: "PM" – premolar, "M" – molar; Cell density: given in cells/well if not otherwise mentioned.
[d] Frequencies labeled bold orange were converted to hertz (Hz) according to its definition using the information reported in the study (in brackets)
[e] Force type deduced from the description of the force apparatus given by the authors.
[f] Gene and protein expression: 1. conclusion of change (increase, decrease…) was given according to the defined criteria in Figure 2; 2. different markers to describe the amount of change; † Information derived from figures using Engauge Digitizer; *Folds calculated by measuring the graphs, without using the Engauge Digitizer; No makers: Information derived from figures by description in the articles

Reference	Gene/ Analyte[a]	Official gene symbol / abbreviation[b]	Cell (age/gender of donors, tooth type, isolation method, passages used, cell density)[a,c]	Force type (stat./ dyn.)[a]	Force duration and frequency[d]	Force magnitude[a]	Force apparatus[a]	Force type: equibiaxial or uniaxial[e]	Gene expression: Increase, decrease, no change (method w/ reference gene); Methods: qPCR, sqPCR, Northern blot[f]	Gene expression: When it reaches peak and peak's magnitude (fold change; times or ratio; unclear = ?)[f]	Protein expression: Increase, decrease, no change (method w/ reference); Methods: ELISA, WB, RIA, EMSA, IF[f]	Protein expression: When it reaches peak and peak's magnitude (times or ratio; unclear = ?)[f]
Shen et al. (2014)	OCN	*BGLAP*	hPDLSC cells (12-24/n.g., PM, dig, P4-6, 100% confluence)	dynamic	0.1Hz for 6h, 12h, 24h	12%	Flexcell FX-4000T Tension Plus System + 6-well, flexible-bottomed culture plate coated with type I collagen (Sigma)+ vacuum	equibiaxial	increase (qPCR, β-actin)	24h: 10.5 (rel)* / 2.6 (ratio-calc)	increase (WB, GAPDH)	24h: 279.2 (rel) * / 1.5 (ratio-calc)
Shen et al. (2014)	Runx2	*RUNX2*	hPDLSC cells (12-24/n.g., PM, dig, P4-6, 100% confluence)	dynamic	0.1Hz for 6h, 12h, 24h	12%	Flexcell FX-4000T Tension Plus System + 6-well, flexible-bottomed culture plate coated with type I collagen (Sigma)+ vacuum	equibiaxial	increase (qPCR, β-actin)	24h: 13.5 (rel)* / 3.4 (ratio-calc)	increase (WB, GAPDH)	24h: 415.1 (rel)* / 1.3 (ratio-calc)
Shimizu et al. (1994)	IL-1β	*IL1B*	hPDL cells (12/M, PM, exp, P4, confluent)	dynamic	**0.1Hz** (6cyc/min) for 1d, 3d, 5d	9%, 18%	Flexercell Strain Unit + flexible bottomed culture plates (Flexcell Corp) + vacuum	equibiaxial	n.g.	n.g.	9%: increase followed by plateau (RIA)<br>18%: increase followed by plateau (RIA)	9% @ 3d…5d: 27.9 (fmol/10^5 cells)* / 1.2 (ratio-calc)<br>18% @ 3d…5d: 42.3 (fmol/10^5 cells)* / 1.8 (ratio-calc)
Shimizu et al. (1995)	IL-1β	*IL1B*	hPDL cells (12/M, PM, exp, P4, confluent)	dynamic	**0.1Hz** (6cyc/min) for 1d, 3d, 5d	18%	Flexercell Strain Unit + flexible bottomed culture plates (Flexcell Corp) + vacuum	equibiaxial	n.g.	n.g.	increase followed by plateau (radioactivity)	3d...5d: 54.1 (fmol/10^5 cells)* / 2.1 (ratio-calc)
Shimizu et al. (1995)	PGE_2	PGE_2	hPDL cells (12/M, PM, exp, P4, confluent)	dynamic	**0.1Hz** (6cyc/min) for 1d, 3d, 5d	18%	Flexercell Strain Unit + flexible bottomed culture plates (Flexcell Corp) + vacuum	equibiaxial	n.a.	n.a.	increase (RIA)	5d: 7.9 (ng/10^6 cells)* / 19.8 (ratio-calc)
Shimizu et al. (1997)	ICE	*CASP1*	hPDL cells (18/F, 19/F, 23/F, PM, exp, P5-6 and P18-20, n.g.)	dynamic	**0.1Hz** (6cyc/min) for 3d	9%, 18%	Flexcell strain unit + flexible-bottomed culture plates + vacuum (Shimizu 1994; further reference to Banes 1985)	equibiaxial	"young cells" (P5-6): no change (sqPCR, GAPDH)<br>"old cells" (P18-20): no change (sqPCR, GAPDH)	no quantitative information is given	n.g.	n.g.
Shimizu et al. (1997)	IL-1β	*IL1B*	hPDL cells (18/F, 19/F, 23/F, PM, exp, P5-6 and P18-20, n.g.)	dynamic	**0.1Hz** (6cyc/min) RIA for 1d, 3d, 5d sqPCR for 3d	18%	Flexcell strain unit + flexible-bottomed culture plates + vacuum (Shimizu 1994; further reference to Banes 1985)	equibiaxial	"young cells" (P5-6): increase (sqPCR, GAPDH)<br>"old cells" (P18-20): increase (sqPCR, GAPDH)	no quantitative information is given	"young cells": increase followed by plateau (RIA)<br>"old cells" increase (RIA)	"young cells" @ 3d…5d: 40 (fmol/10^5 cells)* / 2.1 (ratio-calc)<br>"old cells" @ 5d: 60 (fmol/10^5 cells)* / 3 (ratio-calc)
Shimizu et al. (1997)	IL-1β	*IL1B*	hPDL cells (18/F, 19/F, 23/F, PM, exp, P5-6 and P18-20, n.g.)	dynamic	**0.1Hz** (6cyc/min) for 5d	9%, 18%	Flexcell strain unit + flexible-bottomed culture plates + vacuum (Shimizu 1994; further reference to Banes 1985)	equibiaxial	n.g.	n.g.	"young cells" (P5-6): increase (RIA)<br>"old cells" (P18-20): increase (RIA)	"young cells" @ 18%: 41.6 (fmol/10^5 cells)* / 2.1 (ratio-calc)<br>"old cells" @ 18%: 61.3 (fmol/10^5 cells)* / 3.1 (ratio-calc)
Shimizu et al. (1997)	IL-1β	*IL1B*	hPDL cells (18/F, 19/F, 23/F, PM, exp, P5-6 and P18-20, n.g.)	dynamic	**0.1Hz** (6cyc/min) for 5d	n.g.	Flexcell strain unit + flexible-bottomed culture plates + vacuum (Shimizu 1994; further reference to Banes 1985)	equibiaxial	n.g.	n.g.	Donor 1, "young cells" (P5-6): increase (RIA)<br>Donor 1, "old cells" (P18-20): increase (RIA)<br>Donor 2, "young cells" (P5-6): increase (RIA)<br>Donor 2, "old cells" (P18-20): increase (RIA)<br>Donor 3, "young cells" (P5-6): increase (RIA)<br>Donor 3, "old cells" (P18-20): increase (RIA)	Donor 1, "young cells": 39.8 (fmol/10^5 cells)† / 2.1 (ratio-calc)<br>Donor 1, "old cells" 60.2 (fmol/10^5 cells)† / 3.0 (ratio-calc)<br>Donor 2, "young cells": 35.0 (fmol/10^5 cells)† / 2.4 (ratio-calc)<br>Donor 2, "old cells": 51.7 (fmol/10^5 cells)† / 3.3 (ratio-calc)<br>Donor 3, "young cells": 39.3 (fmol/10^5 cells)† / 2.2 (ratio-calc)<br>Donor 3, "old cells": 55.3 (fmol/10^5 cells)† / 3.2 (ratio-calc)
Shimizu et al. (1998)	COX-1	*PTGS1*	hPDL cells (12/M, PM, exp, P5, confluent)	dynamic	**0.1Hz** (6cyc/min) for 3d	18%	vacuum unit from Flexcell Corporation	equibiaxial	no change (sqPCR, GAPDH)	no quantitative information is given	n.g.	n.g.
Shimizu et al. (1998)	COX-2	*PTGS2*	hPDL cells (12/M, PM, exp, P5, confluent)	dynamic	**0.1Hz** (6cyc/min) for 6h, 24h, 3d, 5d	18%	Flexcell strain unit + flexible-bottomed culture plates + vacuum (Shimizu 1994; further reference to Banes 1985)	equibiaxial	increase (sqPCR, GAPDH)	no quantitative information is given	n.g.	n.g.
Shimizu et al. (1998)	PGE_2	PGE_2	hPDL cells (12/M, PM, exp, P5, confluent)	dynamic	**0.1Hz** (6cyc/min) for 1d, 2d, 3d, 4d, 5d	18%	vacuum unit from Flexcell Corporation	equibiaxial	n.a.	n.a.	increase (RIA)	5d: 9.1(ng/10^6 cells) / 10.1 (ratio-calc)
Spencer and Lallier (2009)	OPG	*TNFRSF11B*	hPDL cells (16-35/n.g., n.g., n.g., P8-15, n.g.)	static	12h	10%	Petriperm dish + two pieces of acrylic + screws	equibiaxial	increase (sqPCR, S15rRNA)	2.3 (ratio)*	n.g.	n.g.
Spencer and Lallier (2009)	Plexin A1	*PLXNA1*	hPDL cells (16-35/n.g., n.g., n.g., P8-15, n.g.)	static	12h	10%	Petriperm dish + two pieces of acrylic + screws	equibiaxial	decrease (sqPCR, S15rRNA)	0.3 (ratio)*	n.g.	n.g.
Spencer and Lallier (2009)	Plexin B1	*PLXNB1*	hPDL cells (16-35/n.g., n.g., n.g., P8-15, n.g.)	static	12h	10%	Petriperm dish + two pieces of acrylic + screws	equibiaxial	no change (sqPCR, S15rRNA)		n.g.	n.g.
Spencer and Lallier (2009)	Plexin C1	*PLXNC1*	hPDL cells (16-35/n.g., n.g., n.g., P8-15, n.g.)	static	12h	10%	Petriperm dish + two pieces of acrylic + screws	equibiaxial	increase (sqPCR, S15rRNA)	3.5 (ratio)*	n.g.	n.g.
Spencer and Lallier (2009)	RANKL	*TNFSF11*	hPDL cells (16-35/n.g., n.g., n.g., P8-15, n.g.)	static	12h	10%	Petriperm dish + two pieces of acrylic + screws	equibiaxial	decrease (sqPCR, S15rRNA)	0.2 (ratio)*	n.g.	n.g.
Spencer and Lallier (2009)	rRNA S15	*RPS15*	hPDL cells (16-35/n.g., n.g., n.g., P8-15, n.g.)	static	12h	10%	Petriperm dish + two pieces of acrylic + screws	equibiaxial	no change (sqPCR, S15rRNA)		n.g.	n.g.
Spencer and Lallier (2009)	Sem3A	*SEMA3A*	hPDL cells (16-35/n.g., n.g., n.g., P8-15, n.g.)	static	12h	10%	Petriperm dish + two pieces of acrylic + screws	equibiaxial	no change (sqPCR, S15rRNA)		n.g.	n.g.
Spencer and Lallier (2009)	Sem3C	*SEMA3C*	hPDL cells (16-35/n.g., n.g., n.g., P8-15, n.g.)	static	12h	10%	Petriperm dish + two pieces of acrylic + screws	equibiaxial	no change (sqPCR, S15rRNA)		n.g.	n.g.
Spencer and Lallier (2009)	Sem3D	*SEMA3D*	hPDL cells (16-35/n.g., n.g., n.g., P8-15, n.g.)	static	12h	10%	Petriperm dish + two pieces of acrylic + screws	equibiaxial	increase (sqPCR, S15rRNA)	22 (ratio)*	n.g.	n.g.
Spencer and Lallier (2009)	Sem3E	*SEMA3E*	hPDL cells (16-35/n.g., n.g., n.g., P8-15, n.g.)	static	12h	10%	Petriperm dish + two pieces of acrylic + screws	equibiaxial	no change (sqPCR, S15rRNA)		n.g.	n.g.
Spencer and Lallier (2009)	Sem4A	*SEMA4A*	hPDL cells (16-35/n.g., n.g., n.g., P8-15, n.g.)	static	12h	10%	Petriperm dish + two pieces of acrylic + screws	equibiaxial	no change (sqPCR, S15rRNA)		n.g.	n.g.

[a] Entry given as reported in the study.
[b] All official gene symbols come from the HUGO Gene Nomenclature Committee (HGNC; URL: https://www.genenames.org) after checking specificity of primers with Primer-BLAST.
[c] Gender/Sex of donors: "M" – male, "F" – female; Tooth type: "PM" – premolar, "M" – molar; Cell density: given in cells/well if not otherwise mentioned.
[d] Frequencies labeled bold orange were converted to hertz (Hz) according to its definition using the information reported in the study (in brackets)
[e] Force type deduced from the description of the force apparatus given by the authors.
[f] Gene and protein expression: 1. conclusion of change (increase, decrease…) was given according to the defined criteria in Figure 2; 2. different markers to describe the amount of change; † Information derived from figures using Engauge Digitizer; *Folds calculated by measuring the graphs, without using the Engauge Digitizer; No makers: Information derived from figures by description in the articles

Reference	Gene/ Analyte[a]	Official gene symbol / abbreviation[b]	Cell (age/gender of donors, tooth type, isolation method, passages used, cell density)[a,c]	Force type (stat./ dyn.)[a]	Force duration and frequency[d]	Force magnitude[a]	Force apparatus[a]	Force type: equibiaxial or uniaxial[e]	Gene expression: Increase, decrease, no change (method w/ reference gene); Methods: qPCR, sqPCR, Northern blot[f]	Gene expression: When it reaches peak and peak's magnitude (fold change; times or ratio; unclear = ?)[f]	Protein expression: Increase, decrease, no change (method w/ reference); Methods: ELISA, WB, RIA, EMSA, IF[f]	Protein expression: When it reaches peak and peak's magnitude (times or ratio; unclear = ?)[f]
Spencer and Lallier (2009)	Sem4C	*SEMA4C*	hPDL cells (16-35/n.g., n.g., n.g., P8-15, n.g.)	static	12h	10%	Petriperm dish + two pieces of acrylic + screws	equibiaxial	no change (sqPCR, S15rRNA)		n.g.	n.g.
Spencer and Lallier (2009)	Sem4D	*SEMA4D*	hPDL cells (16-35/n.g., n.g., n.g., P8-15, n.g.)	static	12h	10%	Petriperm dish + two pieces of acrylic + screws	equibiaxial	no change (sqPCR, S15rRNA)		n.g.	n.g.
Spencer and Lallier (2009)	Sem4F	*SEMA4F*	hPDL cells (16-35/n.g., n.g., n.g., P8-15, n.g.)	static	12h	10%	Petriperm dish + two pieces of acrylic + screws	equibiaxial	no change (sqPCR, S15rRNA)		n.g.	n.g.
Spencer and Lallier (2009)	Sem5A	*SEMA5A*	hPDL cells (16-35/n.g., n.g., n.g., P8-15, n.g.)	static	12h	10%	Petriperm dish + two pieces of acrylic + screws	equibiaxial	no change (sqPCR, S15rRNA)		n.g.	n.g.
Spencer and Lallier (2009)	Sem5B	*SEMA5B*	hPDL cells (16-35/n.g., n.g., n.g., P8-15, n.g.)	static	12h	10%	Petriperm dish + two pieces of acrylic + screws	equibiaxial	increase (sqPCR, S15rRNA)	8 (ratio)*	n.g.	n.g.
Spencer and Lallier (2009)	Sem6B	*SEMA6B*	hPDL cells (16-35/n.g., n.g., n.g., P8-15, n.g.)	static	12h	10%	Petriperm dish + two pieces of acrylic + screws	equibiaxial	no change (sqPCR, S15rRNA)		n.g.	n.g.
Spencer and Lallier (2009)	Sem6C	*SEMA6C*	hPDL cells (16-35/n.g., n.g., n.g., P8-15, n.g.)	static	12h	10%	Petriperm dish + two pieces of acrylic + screws	equibiaxial	no change (sqPCR, S15rRNA)		n.g.	n.g.
Spencer and Lallier (2009)	Sem7A	*SEMA7A*	hPDL cells (16-35/n.g., n.g., n.g., P8-15, n.g.)	static	12h	10%	Petriperm dish + two pieces of acrylic + screws	equibiaxial	decrease (sqPCR, S15rRNA)	0.07 (ratio)*	n.g.	n.g.
Spencer and Lallier (2009)	B1 integrin	*ITGB1*	hPDL cells (16-35/n.g., n.g., n.g., P8-15, n.g.)	static	12h	10%	Petriperm dish + two pieces of acrylic + screws	equibiaxial	no change (sqPCR, S15rRNA)		n.g.	n.g.
Steinberg et al. (2011)	MAP4	*MAP4*	hPDL cells (14/n.g., PM, exp, P6-10, 75-85% confluence)	static	6h, 12h, 18h, 24h	2.5%	Lumox culture dishes (Greiner Bio-One) + template with convex surface + weight (Hasegawa et al 1985)	equibiaxial	n.g.	n.g.	temporary decrease (WB)	6h: 36% (% of control) / 0.4 (ratio-calc)
Steinberg et al. (2011)	Myo IC, cytoplasmic	*MYO1C*	hPDL cells (14/n.g., PM, exp, P6-10, 75-85% confluence)	static	6h, 12h, 18h, 24h	2.5%	Lumox culture dishes (Greiner Bio-One) + template with convex surface + weight (Hasegawa et al 1985)	equibiaxial	n.g.	n.g.	increase (WB)	18h: 257% (% of control) / 2.6 (ratio-calc)
Steinberg et al. (2011)	NM1	*MYO1C*	hPDL cells (14/n.g., PM, exp, P6-10, 75-85% confluence)	static	6h, 12h, 18h, 24h	2.5%	Lumox culture dishes (Greiner Bio-One) + template with convex surface + weight (Hasegawa et al 1985)	equibiaxial	n.g.	n.g.	decrease (WB)	18h: 42% (% of control) / 0.4 (ratio-calc)
Steinberg et al. (2011)	PROTEOMIC S	PROTEOMICS	hPDL cells (14/n.g., PM, exp, P6-10, 75-85% confluence)	static	3h, 6h, 12h	2.5%	Lumox culture dishes (Greiner Bio-One) + template with convex surface + weight (Hasegawa et al 1985)	equibiaxial	n.a.	n.a.	1D-SDS-PAGE of subcellular protein fractions; LC-ESI-MS/MS; MASCOT search	
Steinberg et al. (2011)	Talin	*TLN1; TLN2*	hPDL cells (14/n.g., PM, exp, P6-10, 75-85% confluence)	static	6h, 12h, 18h, 24h	2.5%	Lumox culture dishes (Greiner Bio-One) + template with convex surface + weight (Hasegawa et al 1985)	equibiaxial	n.g.	n.g.	temporary decrease (WB)	6h: 53% (% of control) / 0.5 (ratio-calc)
Sun et al. (2016)	COL1	*COL1A1*	hPDL cells (18/M, 20/M, 20/F, 24/F, PM and M, dig, P3-5, confluency)	dynamic	0.5Hz for 4h per day for 1d, 5d	12%	FX-5000T Flexcell Tension Plus unit + Bioflex Flexcell + vacuum	uniaxial	temporary decrease (qPCR, GAPDH)	1d: 0.7 (ratio)*	decrease (WB, GAPDH)	1d: 0.6 (ratio)*
Sun et al. (2016)	IL-1β	*IL1B*	hPDL cells (18/M, 20/M, 20/F, 24/F, PM and M, dig, P3-5, confluency)	dynamic	0.5Hz for 4h per day for 1d, 5d	12%	FX-5000T Flexcell Tension Plus unit + Bioflex Flexcell + vacuum	uniaxial	n.g.	n.g.	increase (ELISA)	5d: 53 (pg/ml)* / control not detectable
Sun et al. (2016)	RUNX2	*RUNX2*	hPDL cells (18/M, 20/M, 20/F, 24/F, PM and M, dig, P3-5, confluency)	dynamic	0.5Hz for 4h per day for 1d, 5d	12%	FX-5000T Flexcell Tension Plus unit + Bioflex Flexcell + vacuum	uniaxial	increase followed by decrease (qPCR, GAPDH)	highest @ 1d: 2.0 (ratio)*<br>lowest @ 5d: 0.9 (ratio)*	increase followed by decrease (WB, GAPDH)	highest @ 1d: 1.5 (ratio)*<br>lowest @ 5d: 0.8 (ratio)*
Sun et al. (2016)	TNF-α	*TNF*	hPDL cells (18/M, 20/M, 20/F, 24/F, PM and M, dig, P3-5, confluency)	dynamic	0.5Hz for 4h per day for 1d, 5d	12%	FX-5000T Flexcell Tension Plus unit + Bioflex Flexcell + vacuum	uniaxial	n.g.	n.g.	increase (ELISA)	5d: 41 (pg/ml)* / control not detectable
Sun et al. (2017)	COL1	*COL1A1*	hPDL cells (18-28/n.g., n.g., dig, P3-5, 70-80% confluence)	dynamic	0.5Hz for 12h, 24h, 48h	12%	FX-5000T™ Flexercell Tension Plus™ + COL-I-coated silicone Bioflex® culture plates + vacuum	uniaxial	decrease (qPCR, GAPDH)	24h: 0.5 (ratio)*	decrease (WB,GAPDH)	24h: 0.4 (ratio)*
Sun et al. (2017)	IL-1β	*IL1B*	hPDL cells (18-28/n.g., n.g., dig, P3-5, 70-80% confluence)	dynamic	0.5Hz for 12h, 24h, 48h	12%	FX-5000T™ Flexercell Tension Plus™ + COL-I-coated silicone Bioflex® culture plates + vacuum	uniaxial	n.g.	n.g.	increase (ELISA)	48h: 89.76 (pg/ml) / 89.8 (ratio-calc)
Sun et al. (2017)	RUNX2	*RUNX2*	hPDL cells (18-28/n.g., n.g., dig, P3-5, 70-80% confluence)	dynamic	0.5Hz for 12h, 24h, 48h	12%	FX-5000T™ Flexercell Tension Plus™ + COL-I-coated silicone Bioflex® culture plates + vacuum	uniaxial	temporary decrease (qPCR, GAPDH)	12h: 0.5 (ratio)	temporary decrease (WB,GAPDH)	12h: 0.8 (ratio)*
Sun et al. (2017)	TNF-α	*TNF*	hPDL cells (18-28/n.g., n.g., dig, P3-5, 70-80% confluence)	dynamic	0.5Hz for 12h, 24h, 48h	12%	FX-5000T™ Flexercell Tension Plus™ + COL-I-coated silicone Bioflex® culture plates + vacuum	uniaxial	n.g.	n.g.	increase (ELISA)	48h: 77.52 (pg/ml) / 77.5 (ratio-calc)
Suzuki et al. (2014)	BMP-2	*BMP2*	hPDL cells (19-29/n.g., M, n.g., P3-10, confluent)	dynamic	**0.017Hz** (1/60Hz) for 6h	3%, 5%, 10%	STB-140 STREX cell stretch system (Strex Co) + silicone resin chamber (size 32×32 mm, STB-CH-10.0, Strex Co.) + motor	uniaxial	increase (qPCR, GAPDH)	10%: 5.3 (ratio)*	n.g.	n.g.
Suzuki et al. (2014)	BMP-2	*BMP2*	hPDL cells (19-29/n.g., M, n.g., P3-10, confluent)	dynamic	**0.017Hz** (1/60Hz) for 6h, 12h, 24h	5%	STB-140 STREX cell stretch system (Strex Co) + silicone resin chamber (size 32×32 mm, STB-CH-10.0, Strex Co.) + motor	uniaxial	increase (qPCR, GAPDH)	6h: 5 (ratio)*	n.g.	n.g.
Suzuki et al. (2014)	BMP-4	*BMP4*	hPDL cells (19-29/n.g., M, n.g., P3-10, confluent)	dynamic	**0.017Hz** (1/60Hz) for 6h	5%	STB-140 STREX cell stretch system (Strex Co) + silicone resin chamber (size 32×32 mm, STB-CH-10.0, Strex Co.) + motor	uniaxial	increase (qPCR, GAPDH)	1.3 (ratio)*	n.g.	n.g.
Suzuki et al. (2014)	COX-2	*PTGS2*	hPDL cells (19-29/n.g., M, n.g., P3-10, confluent)	dynamic	**0.017Hz** (1/60Hz) for 6h	5%	STB-140 STREX cell stretch system (Strex Co) + silicone resin chamber (size 32×32 mm, STB-CH-10.0, Strex Co.) + motor	uniaxial	increase (qPCR, GAPDH)	6h: 66.3 (rel)* / 25.2 (ratio-calc)	n.g.	n.g.
Suzuki et al. (2014)	EP1	*PTGER1*	hPDL cells (19-29/n.g., M, n.g., P3-10, confluent)	dynamic	**0.017Hz** (1/60Hz) for 6h	5%	STB-140 STREX cell stretch system (Strex Co) + silicone resin chamber (size 32×32 mm, STB-CH-10.0, Strex Co.) + motor	uniaxial	not detectable (sqPCR, GAPDH)		n.g.	n.g.

[a] Entry given as reported in the study.
[b] All official gene symbols come from the HUGO Gene Nomenclature Committee (HGNC; URL: https://www.genenames.org) after checking specificity of primers with Primer-BLAST.
[c] Gender/Sex of donors: "M" – male, "F" – female; Tooth type: "PM" – premolar, "M" – molar; Cell density: given in cells/well if not otherwise mentioned.
[d] Frequencies labeled bold orange were converted to hertz (Hz) according to its definition using the information reported in the study (in brackets)
[e] Force type deduced from the description of the force apparatus given by the authors.
[f] Gene and protein expression: 1. conclusion of change (increase, decrease…) was given according to the defined criteria in Figure 2; 2. different markers to describe the amount of change; † Information derived from figures using Engauge Digitizer; *Folds calculated by measuring the graphs, without using the Engauge Digitizer; No makers: Information derived from figures by description in the articles

Reference	Gene/ Analyte[a]	Official gene symbol / abbreviation[b]	Cell (age/gender of donors, tooth type, isolation method, passages used, cell density)[a,c]	Force type (stat./ dyn.)[a]	Force duration and frequency[d]	Force magnitude[a]	Force apparatus[a]	Force type: equibiaxial or uniaxial[e]	Gene expression: Increase, decrease, no change (method w/ reference gene); Methods: qPCR, sqPCR, Northern blot[f]	Gene expression: When it reaches peak and peak's magnitude (fold change; times or ratio; unclear = ?)[f]	Protein expression: Increase, decrease, no change (method w/ reference); Methods: ELISA, WB, RIA, EMSA, IF[f]	Protein expression: When it reaches peak and peak's magnitude (times or ratio; unclear = ?)[f]
Suzuki et al. (2014)	EP2	*PTGER2*	hPDL cells (19-29/n.g., M, n.g., P3-10, confluent)	dynamic	**0.017Hz** (1/60Hz) for 6h	5%	STB-140 STREX cell stretch system (Strex Co) + silicone resin chamber (size 32×32 mm, STB-CH-10.0, Strex Co.) + motor	uniaxial	increase (sqPCR, GAPDH)	1.6 (ratio)*	n.g.	n.g.
Suzuki et al. (2014)	EP3	*PTGER3*	hPDL cells (19-29/n.g., M, n.g., P3-10, confluent)	dynamic	**0.017Hz** (1/60Hz) for 6h	5%	STB-140 STREX cell stretch system (Strex Co) + silicone resin chamber (size 32×32 mm, STB-CH-10.0, Strex Co.) + motor	uniaxial	no change (sqPCR, GAPDH)		n.g.	n.g.
Suzuki et al. (2014)	EP4	*PTGER4*	hPDL cells (19-29/n.g., M, n.g., P3-10, confluent)	dynamic	**0.017Hz** (1/60Hz) for 6h	5%	STB-140 STREX cell stretch system (Strex Co) + silicone resin chamber (size 32×32 mm, STB-CH-10.0, Strex Co.) + motor	uniaxial	increase (sqPCR, GAPDH)	3.6 (ratio)*	n.g.	n.g.
Suzuki et al. (2014)	ERK1/2 / p-ERK1/2	MAPK3; MAPK1	hPDL cells (19-29/n.g., M, n.g., P3-10, confluent)	dynamic	**0.017Hz** (1/60Hz) for 15min, 45min	5%	STB-140 STREX cell stretch system (Strex Co) + silicone resin chamber (size 32×32 mm, STB-CH-10.0, Strex Co.) + motor	uniaxial	n.g.	n.g.	ERK1/2: no change (WB)<br>p-ERK1/2: increase (WB)	ERK1/2: no quantitative information is given<br>p-ERK1/2 @ 45min: 6.3 (rel)* / 3.9 (ratio-calc)
Suzuki et al. (2014)	IGF-1	*IGF1*	hPDL cells (19-29/n.g., M, n.g., P3-10, confluent)	dynamic	**0.017Hz** (1/60Hz) for 6h	5%	STB-140 STREX cell stretch system (Strex Co) + silicone resin chamber (size 32×32 mm, STB-CH-10.0, Strex Co.) + motor	uniaxial	decrease (qPCR, GAPDH)	0.4 (ratio)*	n.g.	n.g.
Suzuki et al. (2014)	JNK / p-JNK	*MAPK8*	hPDL cells (19-29/n.g., M, n.g., P3-10, confluent)	dynamic	**0.017Hz** (1/60Hz) for 15min, 45min	5%	STB-140 STREX cell stretch system (Strex Co) + silicone resin chamber (size 32×32 mm, STB-CH-10.0, Strex Co.) + motor	uniaxial	n.g.	n.g.	JNK: no change (WB)<br>p-JNK: increase (WB)	JNK: no quantitative information is given<br>p-JNK @ 45min: 32 (rel)* / 10 (ratio-calc)
Suzuki et al. (2014)	p38 / p-p38	*MAPK14*	hPDL cells (19-29/n.g., M, n.g., P3-10, confluent)	dynamic	**0.017Hz** (1/60Hz) for 15min, 45min	5%	STB-140 STREX cell stretch system (Strex Co) + silicone resin chamber (size 32×32 mm, STB-CH-10.0, Strex Co.) + motor	uniaxial	n.g.	n.g.	p38: no change (WB)<br>p-p38: increase (WB)	p38: no quantitative information is given<br>p-p38 @ 45min: 4 (rel)* / 1.9 (ratio-calc)
Suzuki et al. (2014)	PGE_2	PGE_2	hPDL cells (19-29/n.g., M, n.g., P3-10, confluent)	dynamic	**0.017Hz** (1/60Hz) for 6h	5%	STB-140 STREX cell stretch system (Strex Co) + silicone resin chamber (size 32×32 mm, STB-CH-10.0, Strex Co.) + motor	uniaxial	n.a.	n.a.	increase followed by plateau (ELISA)	1h…6h: 229.9 (pg/ml)* / 2.9 (ratio-calc)
Symmank et al. (2019)	GDF15	*GDF15*	hPDLFs (n.g./n.g., n.g., n.g., P4-6, confluence)	static	3h, 6h, 12h	5%	Flexcell FX-3000™ Tension System + pronectin-coated Biofex plates + vacuum	equibiaxial	increase (qPCR, GAPDH and ACTB)	3h: 2.9 (FC)†	increase (ELISA)	12h: 615.5 (pg/ml)† / 4.4 (ratio-calc)
Takano et al. (2009)	COL1	*COL1A1*	hPDL cells (14-16/n.g., PM, exp, P6-9, confluent)	static	12h	5%, 10%	STREX system + STREX-chamber ST-CH-10 (STREX Co.) + manual device (STB-10; STREX Co.)	uniaxial	increase (qPCR, β-actin)	10%: 1.9 (ratio)*	increase (ELISA)	10%: 2.4 (ug/ml)* / 1.7 (ratio-calc)
Takano et al. (2009)	MMP-1	*MMP1*	hPDL cells (14-16/n.g., PM, exp, P6-9, confluent)	static	12h	5%, 10%	STREX system + STREX-chamber ST-CH-10 (STREX Co.) + manual device (STB-10; STREX Co.)	uniaxial	increase (qPCR, β-actin)	10%: 1.6 (rel)* / 2 (ratio-calc)	increase (ELISA)	10%: 3.8 (ug/ml)* / 1.6 (ratio-calc)
Tang et al. (2012)	Osx	*SP7*	hPDLSCs (12-18/n.g., PM, dig, CD146 enrichment, P2, 1×10^5 cells/cm^2)	dynamic	0.5Hz for 3h, 6h, 12h, 24h	**0.3%** (3000μstrain)	four-point bending strain unit (SXG4201, University of Electronic Science and Technology of China, China) + force-loading plates made from bottom part of the 75 cm^2 cell culture flasks (with canted neck (No.353135, BD Falcon) + actuator (Li et al 2009, Wang et al 2010; further reference to Liu 2006)	uniaxial	increase (qPCR, GAPDH)	24h: 15.4 (rel)* / 8.6 (ratio-calc)	increase (WB, GAPDH)	24h: 1.2 (rel)* / 1.4 (ratio-calc)
Tang et al. (2012)	Runx2	*RUNX2*	hPDLSCs (12-18/n.g., PM, dig, CD146 enrichment, P2, 1×10^5 cells/cm^2)	dynamic	0.5Hz for 3h, 6h, 12h, 24h	**0.3%** (3000μstrain)	four-point bending strain unit (SXG4201, University of Electronic Science and Technology of China, China) + force-loading plates made from bottom part of the 75 cm^2 cell culture flasks (with canted neck (No.353135, BD Falcon) + actuator (Li et al 2009, Wang et al 2010; further reference to Liu 2006)	uniaxial	increase (qPCR, GAPDH)	24h: 9.6 (rel)* / 7.4 (ratio-calc)	increase followed by plateau (WB, GAPDH)?	12h…24h: 1.1 (rel)* / 1.4 (ratio-calc)
Tang et al. (2012)	Satb2	*SATB2*	hPDLSCs (12-18/n.g., PM, dig, CD146 enrichment, P2, 1×10^5 cells/cm^2)	dynamic	0.5Hz for 3h, 6h, 12h, 24h	**0.3%** (3000μstrain)	four-point bending strain unit (SXG4201, University of Electronic Science and Technology of China, China) + force-loading plates made from bottom part of the 75 cm^2 cell culture flasks (with canted neck (No.353135, BD Falcon) + actuator (Li et al 2009, Wang et al 2010; further reference to Liu 2006)	uniaxial	increase follwed by plateau (qPCR, GAPDH)	6h…24h: 4.5 (rel)* / 4.1 (ratio-calc)	increase followed by plateau (WB, GAPDH)	6h…24h: 1.2 (rel)* / 1.3 (ratio-calc)
Tantilertanant et al. (2019a)	IL6	*IL6*	hPDLCs (n.g./n.g., n.g., 3 donors, exp, P3-8, 2x10^5)	dynamic	**1Hz** (frequency of 60 rpm) for 2h, 6h	10%	uniaxial stretch apparatus developed at the Faculty of Dentistry, Chulalongkorn University + gelatin-coated silicone membranes (2.5×2cm^2; Silastic T-4, Dow Corning, GmbH, Germany)	equibiaxial	donor 1: increase (qPCR, GAPDH)<br>donor 2: increase (qPCR, GAPDH)<br>donor 3: increase (qPCR, GAPDH)	donor 1: 6h: 9.4 (FC)†<br>donor 2: 6h: 10.5 (FC)†<br>donor 3: 6h: 9.9 (FC)†	n.a.	n.a.
Tantilertanant et al. (2019a)	IL6R	*IL6R*	hPDLCs (n.g./n.g., n.g., 3 donors, exp, P3-8, 2x10^5)	dynamic	**1Hz** (frequency of 60 rpm) for 2h	10%	uniaxial stretch apparatus developed at the Faculty of Dentistry, Chulalongkorn University + gelatin-coated silicone membranes (2.5×2cm^2; Silastic T-4, Dow Corning, GmbH, Germany)	equibiaxial	increase (qPCR, GAPDH)	3.2 (FC)†	n.g.	n.g.
Tantilertanant et al. (2019a)	MMP1	*MMP1*	hPDLCs (n.g./n.g., n.g., 3 donors, exp, P3-8, 2x10^5)	dynamic	**1Hz** (frequency of 60 rpm) for 6h	10%	uniaxial stretch apparatus developed at the Faculty of Dentistry, Chulalongkorn University + gelatin-coated silicone membranes (2.5×2cm^2; Silastic T-4, Dow Corning, GmbH, Germany)	equibiaxial	increase (qPCR, GAPDH)	6h: 1.5 (FC)†	n.g.	n.g.
Tantilertanant et al. (2019a)	MMP14	*MMP14*	hPDLCs (n.g./n.g., n.g., 3 donors, exp, P3-8, 2x10^5)	dynamic	**1Hz** (frequency of 60 rpm) for 6h	10%	uniaxial stretch apparatus developed at the Faculty of Dentistry, Chulalongkorn University + gelatin-coated silicone membranes (2.5×2cm^2; Silastic T-4, Dow Corning, GmbH, Germany)	equibiaxial	6h: increase (qPCR, GAPDH)	6h: 1.4 (FC)†	n.g.	n.g.

[a] Entry given as reported in the study.
[b] All official gene symbols come from the HUGO Gene Nomenclature Committee (HGNC; URL: https://www.genenames.org) after checking specificity of primers with Primer-BLAST.
[c] Gender/Sex of donors: "M" – male, "F" – female; Tooth type: "PM" – premolar, "M" – molar; Cell density: given in cells/well if not otherwise mentioned.
[d] Frequencies labeled bold orange were converted to hertz (Hz) according to its definition using the information reported in the study (in brackets)
[e] Force type deduced from the description of the force apparatus given by the authors.
[f] Gene and protein expression: 1. conclusion of change (increase, decrease…) was given according to the defined criteria in Figure 2; 2. different markers to describe the amount of change; † Information derived from figures using Engauge Digitizer; *Folds calculated by measuring the graphs, without using the Engauge Digitizer; No makers: Information derived from figures by description in the articles

Reference	Gene/ Analyte[a]	Official gene symbol / abbreviation[b]	Cell (age/gender of donors, tooth type, isolation method, passages used, cell density)[a,c]	Force type (stat./ dyn.)[a]	Force duration and frequency[d]	Force magnitude[a]	Force apparatus[a]	Force type: equibiaxial or uniaxial[e]	Gene expression: Increase, decrease, no change (method w/ reference gene); Methods: qPCR, sqPCR, Northern blot[f]	Gene expression: When it reaches peak and peak's magnitude (fold change; times or ratio; unclear = ?)[f]	Protein expression: Increase, decrease, no change (method w/ reference); Methods: ELISA, WB, RIA, EMSA, IF[f]	Protein expression: When it reaches peak and peak's magnitude (times or ratio; unclear = ?)[f]
Tantilertanant et al. (2019a)	MMP2	*MMP2*	hPDLCs (n.g./n.g., n.g., 3 donors, exp, P3-8, 2x10^5)	dynamic	**1Hz** (frequency of 60 rpm) for 6h	10%	uniaxial stretch apparatus developed at the Faculty of Dentistry, Chulalongkorn University + gelatin-coated silicone membranes (2.5×2cm^2; Silastic T-4, Dow Corning, GmbH, Germany)	equibiaxial	increase (qPCR, GAPDH)	6h: 1.4 (FC)†	n.g.	n.g.
Tantilertanant et al. (2019a)	MMP3	*MMP3*	hPDLCs (n.g./n.g., n.g., 3 donors, exp, P3-8, 2x10^5)	dynamic	**1Hz** (frequency of 60 rpm) for 6h	10%	uniaxial stretch apparatus developed at the Faculty of Dentistry, Chulalongkorn University + gelatin-coated silicone membranes (2.5×2cm^2; Silastic T-4, Dow Corning, GmbH, Germany)	equibiaxial	increase (qPCR, GAPDH)	6h: 1.5 (FC)†		
Tantilertanant et al. (2019a)	MMP8	*MMP8*	hPDLCs (n.g./n.g., n.g., 3 donors, exp, P3-8, 2x10^5)	dynamic	**1Hz** (frequency of 60 rpm) for 6h	10%	uniaxial stretch apparatus developed at the Faculty of Dentistry, Chulalongkorn University + gelatin-coated silicone membranes (2.5×2cm^2; Silastic T-4, Dow Corning, GmbH, Germany)	equibiaxial	no change (qPCR, GAPDH)		n.g.	n.g.
Tantilertanant et al. (2019a)	TIMP1	*TIMP1*	hPDLCs (n.g./n.g., n.g., 3 donors, exp, P3-8, 2x10^5)	dynamic	**1Hz** (frequency of 60 rpm) for 6h	10%	uniaxial stretch apparatus developed at the Faculty of Dentistry, Chulalongkorn University + gelatin-coated silicone membranes (2.5×2cm^2; Silastic T-4, Dow Corning, GmbH, Germany)	equibiaxial	no change (qPCR, GAPDH)		n.g.	n.g.
Tantilertanant et al. (2019a)	TIMP2	*TIMP2*	hPDLCs (n.g./n.g., n.g., 3 donors, exp, P3-8, 2x10^5)	dynamic	**1Hz** (frequency of 60 rpm) for 6h	10%	uniaxial stretch apparatus developed at the Faculty of Dentistry, Chulalongkorn University + gelatin-coated silicone membranes (2.5×2cm^2; Silastic T-4, Dow Corning, GmbH, Germany)	equibiaxial	no change (qPCR, GAPDH)		n.g.	n.g.
Tantilertanant et al. (2019b)	ATP	ATP	hPDL cells (n.g./n.g., M, exp, P3-4, 2×10^5)	dynamic	**1Hz** (frequency of 60 rpm) for 10min, 20min, 30min, 60min, 120min	10%	uniaxial stretch apparatus developed at the Faculty of Dentistry, Chulalongkorn University + gelatin-coated silicone membranes (2.5×2cm^2; Silastic T-4, Dow Corning, GmbH, Germany)	uniaxial	n.a.	n.a.	increase (Luminescence)	10min: 4.7 (ratio)
Tantilertanant et al. (2019b)	BMP2	*BMP2*	hPDL cells (n.g./n.g., M, exp, P3-4, 2×10^5)	dynamic	**1Hz** (frequency of 60 rpm) for 2h, 6h	10%	uniaxial stretch apparatus developed at the Faculty of Dentistry, Chulalongkorn University + gelatin-coated silicone membranes (2.5×2cm^2; Silastic T-4, Dow Corning, GmbH, Germany)	uniaxial	increase (sqPCR, GAPDH)	6h: 7.2 (FC)*	n.g.	n.g.
Tantilertanant et al. (2019b)	BMP6	*BMP6*	hPDL cells (n.g./n.g., M, exp, P3-4, 2×10^5)	dynamic	**1Hz** (frequency of 60 rpm) for 2h, 6h	10%	uniaxial stretch apparatus developed at the Faculty of Dentistry, Chulalongkorn University + gelatin-coated silicone membranes (2.5×2cm^2; Silastic T-4, Dow Corning, GmbH, Germany)	uniaxial	increase (sqPCR, GAPDH)	6h: 7.2 (FC)*	n.g.	n.g.
Tantilertanant et al. (2019b)	BMP9	*GDF2*	hPDL cells (n.g./n.g., M, exp, P3-4, 2×10^5)	dynamic	**1Hz** (frequency of 60 rpm) sqPCR: for 2h, 6h; ELISA: for 48h	10%	uniaxial stretch apparatus developed at the Faculty of Dentistry, Chulalongkorn University + gelatin-coated silicone membranes (2.5×2cm^2; Silastic T-4, Dow Corning, GmbH, Germany)	uniaxial	increase (sqPCR, GAPDH)	6h: 6.5 (FC)*	conditioned medium: increase (ELISA)<br>cell lysate: increase (ELISA)	conditioned medium: 282.9 (pg)† / 11.0 (ratio-calc)<br>cell lysate increase: 16.24 (pg)† / 3.7 (ratio-calc)
Tantilertanant et al. (2019b)	Noggin	*NOG*	hPDL cells (n.g./n.g., M, exp, P3-4, 2×10^5)	dynamic	**1Hz** (frequency of 60 rpm) for 2h, 6h	10%	uniaxial stretch apparatus developed at the Faculty of Dentistry, Chulalongkorn University + gelatin-coated silicone membranes (2.5×2cm^2; Silastic T-4, Dow Corning, GmbH, Germany)	uniaxial	increase (sqPCR, GAPDH)	6h: 2.4 (FC)*	n.g.	n.g.
Tantilertanant et al. (2019b)	$P2Y_1$	*P2RY1*	hPDL cells (n.g./n.g., M, exp, P3-4, 2×10^5)	dynamic	**1Hz** (frequency of 60 rpm) for 2h, 6h	10%	uniaxial stretch apparatus developed at the Faculty of Dentistry, Chulalongkorn University + gelatin-coated silicone membranes (2.5×2cm^2; Silastic T-4, Dow Corning, GmbH, Germany)	uniaxial	increase (sqPCR, GAPDH)	6h: 3.5 (FC)*	n.g.	n.g.
Tsuji et al. (2004)	MMP-1	*MMP1*	hPDL cells (n.g./n.g., PM, exp, P n.g., 1×10^5)	dynamic	**0.17Hz (1/6Hz)** (10cyc/min) for 48h	20%	Flexercell Strain Unit + culture plates coated with type I collagen (Flex I) + vacuum	equibiaxial	no change (sqPCR, GAPDH)		n.g.	n.g.
Tsuji et al. (2004)	MMP-2	*MMP2*	hPDL cells (n.g./n.g., PM, exp, P n.g., 1×10^5)	dynamic	**0.17Hz (1/6Hz)** (10cyc/min) for 48h	20%	Flexercell Strain Unit + culture plates coated with type I collagen (Flex I) + vacuum	equibiaxial	increase (sqPCR, GAPDH)	0.8 (rel)* / 1.6 (ratio-calc)	n.g.	n.g.
Tsuji et al. (2004)	OPG	*TNFRSF11B*	hPDL cells (n.g./n.g., PM, exp, P n.g., 1×10^5)	dynamic	**0.17Hz (1/6Hz)** (10cyc/min) sqPCR for 12h, 24h, 48h, 72h, 120h; qPCR for 48h	20%	Flexercell Strain Unit + culture plates coated with type I collagen (Flex I) + vacuum	equibiaxial	increase (sqPCR, GAPDH)<br>increase (qPCR, β-actin)	sqPCR @ 48h: 2.2 (ratio)*<br>qPCR @ 48h: 34.8 (rel)* / 6.4 (ratio-calc)	n.g.	n.g.
Tsuji et al. (2004)	OPG	*TNFRSF11B*	hPDL cells (n.g./n.g., PM, exp, P n.g., 1×10^5)	dynamic	**0.17Hz (1/6Hz)** (10cyc/min) for 48h	sqPCR for 5%, 20%, 25%; ELISA for 20%	Flexercell Strain Unit + culture plates coated with type I collagen (Flex I) + vacuum	equibiaxial	increase (sqPCR, GAPDH)	20%: 1.5 (rel)*/ 2.2 (ratio-calc)	increase (ELISA)	20%: 339 (pM) / 3.0 (ratio-calc)

[a] Entry given as reported in the study.
[b] All official gene symbols come from the HUGO Gene Nomenclature Committee (HGNC; URL: https://www.genenames.org) after checking specificity of primers with Primer-BLAST.
[c] Gender/Sex of donors: "M" – male, "F" – female; Tooth type: "PM" – premolar, "M" – molar; Cell density: given in cells/well if not otherwise mentioned.
[d] Frequencies labeled bold orange were converted to hertz (Hz) according to its definition using the information reported in the study (in brackets)
[e] Force type deduced from the description of the force apparatus given by the authors.
[f] Gene and protein expression: 1. conclusion of change (increase, decrease…) was given according to the defined criteria in Figure 2; 2. different markers to describe the amount of change; † Information derived from figures using Engauge Digitizer; *Folds calculated by measuring the graphs, without using the Engauge Digitizer; No makers: Information derived from figures by description in the articles

Reference	Gene/ Analyte[a]	Official gene symbol / abbreviation[b]	Cell (age/gender of donors, tooth type, isolation method, passages used, cell density)[a,c]	Force type (stat./ dyn.)[a]	Force duration and frequency[d]	Force magnitude[a]	Force apparatus[a]	Force type: equibiaxial or uniaxial[e]	Gene expression: Increase, decrease, no change (method w/ reference gene); Methods: qPCR, sqPCR, Northern blot[f]	Gene expression: When it reaches peak and peak's magnitude (fold change; times or ratio; unclear = ?)[f]	Protein expression: Increase, decrease, no change (method w/ reference); Methods: ELISA, WB, RIA, EMSA, IF[f]	Protein expression: When it reaches peak and peak's magnitude (times or ratio; unclear = ?)[f]
Tsuji et al. (2004)	RANKL	*TNFSF11*	hPDL cells (n.g./n.g., PM, exp, P n.g., 1×10^{5})	dynamic	**0.17Hz (1/6Hz)** (10cyc/min) for 48h	20%	Flexercell Strain Unit + culture plates coated with type I collagen (Flex I) + vacuum	equibiaxial	no change (sqPCR, GAPDH)		n.g.	n.g.
Tsuji et al. (2004)	TIMP-1	*TIMP1*	hPDL cells (n.g./n.g., PM, exp, P n.g., 1×10^{5})	dynamic	**0.17Hz (1/6Hz)** (10cyc/min) for 48h	20%	Flexercell Strain Unit + culture plates coated with type I collagen (Flex I) + vacuum	equibiaxial	increase (sqPCR, GAPDH)	0.9 (rel)* / 2.3 (ratio-calc)	n.g.	n.g.
Tsuji et al. (2004)	TIMP-2	*TIMP2*	hPDL cells (n.g./n.g., PM, exp, P n.g., 1×10^{5})	dynamic	**0.17Hz (1/6Hz)** (10cyc/min) for 48h	20%	Flexercell Strain Unit + culture plates coated with type I collagen (Flex I) + vacuum	equibiaxial	increase (sqPCR, GAPDH)	1.1 (rel)* / 1.8 (ratio-calc)	n.g.	n.g.
Tsuruga et al. (2009)	Fibrillin-1	*FBN1*	hPDL cells (n.g./n.g., M, exp, P3-6, confluent)	dynamic	**0.017Hz** (1/60Hz)) for 7d	5%	STB-140 STREX cell stretch system (Strex Co) + silicone chamber precoated with type I collagen + motor	uniaxial	no change (Northern blot, β-actin)	no quantitative information is given	increase (WB, β-actin)	increase of 27% (rel) / 1.3 (ratio-calc)
Tsuruga et al. (2009)	Fibrillin-2	*FBN2*	hPDL cells (n.g./n.g., M, exp, P3-6, confluent)	dynamic	**0.017Hz** (1/60Hz) for 7d	5%	STB-140 STREX cell stretch system (Strex Co) + silicone chamber precoated with type I collagen + motor	uniaxial	no change (Northern blot, β-actin)	no quantitative information is given	increase (WB, β-actin)	increase of 23% (rel) / 1.2 (ratio-calc)
Tsuruga et al. (2009)	MMP-2	*MMP2*	hPDL cells (n.g./n.g., M, exp, P3-6, confluent)	dynamic	**0.017Hz** (1/60Hz) for 7d	5%	STB-140 STREX cell stretch system (Strex Co) + silicone chamber precoated with type I collagen + motor	uniaxial	n.g.	n.g.	increase (WB, β-actin)	2.5 (ratio)
Tsuruga et al. (2012)	Fibulin-5	*FBLN5*	hPDL cells (n.g./n.g., PM, exp, P3-6, confluent)	dynamic	**0.017Hz** (1/60Hz) for 7d	5%	STB-140 STREX cell stretch system (Strex Co) + silicone chamber precoated with type I collagen + motor	uniaxial	n.g.	n.g.	FBLN5: no change (WB, β-actin)<br>LTBP-2/Fibulin-5: decrease	FBLN5: no quantitative information reported;<br>LTBP-2/Fibulin-5: 41% (% of control) / 0.4 (ratio-calc)
Tsuruga et al. (2012)	LTBP-2	*LTBP2*	hPDL cells (n.g./n.g., PM, exp, P3-6, confluent)	dynamic	**0.017Hz** (1/60Hz) for 7d	5%	STB-140 STREX cell stretch system (Strex Co) + silicone chamber precoated with type I collagen + motor	uniaxial	no change (Norhtern blot, β-actin)		cell lysates: decrease (WB, β-actin)<br>cell surface and extracellular proteins (WB, β-actin): biotinylated decrease; nonbiotinylated no change<br>medium: increase (WB, β-actin)<br>LTBP-2/Fibulin-5: decrease	cell lysates: 0.29 (ratio)<br>cell surface and extracellular proteins: biotinylated and nonbiotinylated: no quantity is given<br>medium: no quantity is given<br>41% (rel) / 0.4 (ratio-calc)
Wada et al. (2017)	COX-2	*PTGS2*	immortalized human PDLCs via gene transfection (n.g./n.g., n.g., dig, P n.g., 4.0×10^{5})	static	6h, 12h, 24h	15%	Cell Extender (ver. 3, Molcure, Tokyo, Japan) + Bioflex® plates (Flexcell®) + moving screw	equibiaxial	increase (qPCR, GAPDH)	12h: 3.8 (FC)*	n.g.	n.g.
Wada et al. (2017)	IL-1β	*IL1B*	immortalized human PDLCs via gene transfection (n.g./n.g., n.g., dig, P n.g., 4.0×10^{5})	static	6h, 12h, 24h	15%	Cell Extender (ver. 3, Molcure, Tokyo, Japan) + Bioflex® plates (Flexcell®) + moving screw	equibiaxial	increase (qPCR, GAPDH)	24h: 5.2 (FC)*	n.g.	n.g.
Wada et al. (2017)	IL-6	*IL6*	immortalized human PDLCs via gene transfection (n.g./n.g., n.g., dig, P n.g., 4.0×10^{5})	static	6h, 12h, 24h	15%	Cell Extender (ver. 3, Molcure, Tokyo, Japan) + Bioflex® plates (Flexcell®) + moving screw	equibiaxial	increase (qPCR, GAPDH)	6h: 21 (FC)*	n.g.	n.g.
Wada et al. (2017)	IL-6	*IL6*	immortalized human PDLCs via gene transfection (n.g./n.g., n.g., dig, P n.g., 4.0×10^{5})	dynamic	0.5Hz for n.g.	15%	Cell Extender (ver. 3, Molcure, Tokyo, Japan) + Bioflex® plates (Flexcell®) + moving screw	equibiaxial	increase (qPCR, GAPDH)	duration n.g.: 72.6 (FC)*	n.g.	n.g.
Wada et al. (2017)	Osteopontin	*SPP1*	immortalized human PDLCs via gene transfection (n.g./n.g., n.g., dig, P n.g., 4.0×10^{5})	static	6h, 12h, 24h	15%	Cell Extender (ver. 3, Molcure, Tokyo, Japan) + Bioflex® plates (Flexcell®) + moving screw	equibiaxial	increase (qPCR, GAPDH)	6h: 27 (FC)*	increase (WB, β-actin)	2.2 (ratio)*
Wada et al. (2017)	Runx2	*RUNX2*	immortalized human PDLCs via gene transfection (n.g./n.g., n.g., dig, P n.g., 4.0×10^{5})	static	6h, 12h, 24h	15%	Cell Extender (ver. 3, Molcure, Tokyo, Japan) + Bioflex® plates (Flexcell®) + moving screw	equibiaxial	increase (qPCR, GAPDH)	24h: 1.7 (FC)*	n.g.	n.g.
Wada et al. (2017)	TNF-α	*TNF*	immortalized human PDLCs via gene transfection (n.g./n.g., n.g., dig, P n.g., 4.0×10^{5})	static	6h, 12h, 24h	15%	Cell Extender (ver. 3, Molcure, Tokyo, Japan) + Bioflex® plates (Flexcell®) + moving screw	equibiaxial	increase (qPCR, GAPDH)	12h: 5.4 (FC)*	n.g.	n.g.
Wang et al. (2011)	ARRAY	ARRAY	hPDL cells (12-16/n.g., PM, dig, P4, 1×10^{5} cells/ml)	dynamic	0.5Hz for 2h	0.5% (5000μstrain)	four-point bending system + force-loading plates made out of the bottoms of 250 ml cell culture flasks (Falcon) 8×4 cm^2 in size and 1.15 mm thick	uniaxial	CapitalBio human whole-genome oligonucleotide chip 35k (CapitalBio Co, Beijing, China) spotted with 35,000 genes		n.a.	n.a.
Wang et al. (2011)	BHLHB2	*DEC1*	hPDL cells (12-16/n.g., PM, dig, P4, 1×10^{5} cells/ml)	dynamic	0.5Hz for 2h	0.5% (5000μstrain)	four-point bending system + force-loading plates made out of the bottoms of 250 ml cell culture flasks (Falcon) 8×4 cm^2 in size and 1.15 mm thick	uniaxial	increase (qPCR, GAPDH)	4.4 (FC)*	n.g.	n.g.
Wang et al. (2011)	CCL2	*CCL2*	hPDL cells (12-16/n.g., PM, dig, P4, 1×10^{5} cells/ml)	dynamic	0.5Hz for 2h	0.5% (5000μstrain)	four-point bending system + force-loading plates made out of the bottoms of 250 ml cell culture flasks (Falcon) 8×4 cm^2 in size and 1.15 mm thick	uniaxial	increase (qPCR, GAPDH)	3.9 (FC)*	n.g.	n.g.
Wang et al. (2011)	CDC42EP2	*CDC42EP2*	hPDL cells (12-16/n.g., PM, dig, P4, 1×10^{5} cells/ml)	dynamic	0.5Hz for 2h	0.5% (5000μstrain)	four-point bending system + force-loading plates made out of the bottoms of 250 ml cell culture flasks (Falcon) 8×4 cm^2 in size and 1.15 mm thick	uniaxial	increase (qPCR, GAPDH)	4.9 (FC)*	n.g.	n.g.
Wang et al. (2011)	COX-2	*PTGS2*	hPDL cells (12-16/n.g., PM, dig, P4, 1×10^{5} cells/ml)	dynamic	0.5Hz for 2h	0.5% (5000μstrain)	four-point bending system + force-loading plates made out of the bottoms of 250 ml cell culture flasks (Falcon) 8×4 cm^2 in size and 1.15 mm thick	uniaxial	increase (qPCR, GAPDH)	6.4 (FC)*	n.g.	n.g.
Wang et al. (2011)	IER3	*IER3*	hPDL cells (12-16/n.g., PM, dig, P4, 1×10^{5} cells/ml)	dynamic	0.5Hz for 2h	0.5% (5000μstrain)	four-point bending system + force-loading plates made out of the bottoms of 250 ml cell culture flasks (Falcon) 8×4 cm^2 in size and 1.15 mm thick	uniaxial	increase (qPCR, GAPDH)	4.2 (FC)*	n.g.	n.g.

[a] Entry given as reported in the study.
[b] All official gene symbols come from the HUGO Gene Nomenclature Committee (HGNC; URL: https://www.genenames.org) after checking specificity of primers with Primer-BLAST.
[c] Gender/Sex of donors: "M" – male, "F" – female; Tooth type: "PM" – premolar, "M" – molar; Cell density: given in cells/well if not otherwise mentioned.
[d] Frequencies labeled bold orange were converted to hertz (Hz) according to its definition using the information reported in the study (in brackets)
[e] Force type deduced from the description of the force apparatus given by the authors.
[f] Gene and protein expression: 1. conclusion of change (increase, decrease…) was given according to the defined criteria in Figure 2; 2. different markers to describe the amount of change; † Information derived from figures using Engauge Digitizer; *Folds calculated by measuring the graphs, without using the Engauge Digitizer; No makers: Information derived from figures by description in the articles

Reference	Gene/ Analyte[a]	Official gene symbol / abbreviation[b]	Cell (age/gender of donors, tooth type, isolation method, passages used, cell density)[a,c]	Force type (stat./ dyn.)[a]	Force duration and frequency[d]	Force magnitude[a]	Force apparatus[a]	Force type: equibiaxial or uniaxial[e]	Gene expression: Increase, decrease, no change (method w/ reference gene); Methods: qPCR, sqPCR, Northern blot[f]	Gene expression: When it reaches peak and peak's magnitude (fold change; times or ratio; unclear = ?)[f]	Protein expression: Increase, decrease, no change (method w/ reference); Methods: ELISA, WB, RIA, EMSA, IF[f]	Protein expression: When it reaches peak and peak's magnitude (times or ratio; unclear = ?)[f]
Wang et al. (2011)	KLF10	*KLF10*	hPDL cells (12-16/n.g., PM, dig, P4, 1×10^5 cells/ml)	dynamic	0.5Hz for 2h	0.5% (5000μstrain)	four-point bending system + force-loading plates made out of the bottoms of 250 ml cell culture flasks (Falcon) 8×4 cm^2 in size and 1.15 mm thick	uniaxial	increase (qPCR, GAPDH)	6.2 (FC)*	n.g.	n.g.
Wang et al. (2011)	SPRY2	*SPRY2*	hPDL cells (12-16/n.g., PM, dig, P4, 1×10^5 cells/ml)	dynamic	0.5Hz for 2h	0.5% (5000μstrain)	four-point bending system + force-loading plates made out of the bottoms of 250 ml cell culture flasks (Falcon) 8×4 cm^2 in size and 1.15 mm thick	uniaxial	increase (qPCR, GAPDH)	4.1 (FC)*	n.g.	n.g.
Wang et al. (2013)	Caspase-3, Pro- / Caspase-3, cleaved	*CASP3*	hPDL cells (n.g./n.g., PM, dig, P4-8, 95% confluency)	dynamic	0.1Hz for 6h, 24h	20%	Flexcell Tension Plus system FX-5000T + collagen I-coated six-well Bioflex plates (Flexcel) + vacuum	equibiaxial	n.g.	n.g.	Pro-Caspase 3: no change (WB, β-actin)<br>Cleaved caspase-3: increase (WB, β-actin)	Pro-Caspase-3: no quantitative information is given<br>Cleaved caspase-3: 24h: 2.1 (ratio)*
Wang et al. (2013)	PARP	*PARP1*	hPDL cells (n.g./n.g., PM, dig, P4-8, 95% confluency)	dynamic	0.1Hz for 6h, 24h	20%	Flexcell Tension Plus system FX-5000T + collagen I-coated six-well Bioflex plates (Flexcel) + vacuum	equibiaxial	n.g.	n.g.	116kD PARP: no change (WB, β-actin)<br>85kD PARP: increase (WB, β-actin)	116kD PARP: no quantitative information is given<br>85kD PARP: 24h: 2.2 (ratio)*
Wang et al. (2013)	RhoGDIα	*ARHGDIA*	hPDL cells (n.g./n.g., PM, dig, P4-8, 95% confluency)	dynamic	0.1Hz for 6h, 24h	20%	Flexcell Tension Plus system FX-5000T + collagen I-coated six-well Bioflex plates (Flexcel) + vacuum	equibiaxial	n.g.	n.g.	decrease (WB, β-actin)	24h: 0.4 (ratio)*
Wang et al. (2018)	Actin (f-actin)	*ACTB*	hPDL cells (n.g./n.g., n.g., n.g., P n.g., 1×10^5 cells/cm^2)	dynamic	0.5Hz for 2h, 6h	**0.4%** (4000μstrain)	four-point bending strength device (west China college of Stomatology, Sichuan University, number of national patents of RP China: CN2534576 and CN1425905) (Hu 2015)	uniaxial	n.g.	n.g.	increase (WB, β-actin)	6h: 1.2 (rel)* / 1.7 (ratio-calc)
Wang et al. (2018)	Akt	*AKT1*	hPDL cells (n.g./n.g., n.g., n.g., P n.g., 1×10^5 cells/cm^2)	dynamic	0.5Hz for 2h, 6h	**0.4%** (4000μstrain)	four-point bending strength device (west China college of Stomatology, Sichuan University, number of national patents of RP China: CN2534576 and CN1425905) (Hu 2015)	uniaxial	n.g.	n.g.	increase followed by plateau (WB, β-actin)	2h...6h: 1.2 (rel)* / 2 (ratio-calc)
Wang et al. (2018)	Girdin	*CCDC88A*	hPDL cells (n.g./n.g., n.g., n.g., P n.g., 1×10^5 cells/cm^2)	dynamic	0.5Hz for 2h, 6h	**0.4%** (4000μstrain)	four-point bending strength device (west China college of Stomatology, Sichuan University, number of national patents of RP China: CN2534576 and CN1425905) (Hu 2015)	uniaxial	n.g.	n.g.	increase (WB, β-actin)	6h: 2.7 (rel)* / 1.7 (ratio-calc)
Wang et al. (2019a)	circRNA3154<br>circRNA5034<br>circRNA3133<br>circRNA5045<br>circRNA1818<br>circRNA1358	circRNA3154<br>circRNA5034<br>circRNA3133<br>circRNA5045<br>circRNA1818<br>circRNA1358	hPDLSC (14-16/n.g., PM, dig and limited dilution, P3, 5×10^5)	dynamic	1.0Hz for 12h	10%	Flexcell FX-5000 + Flexcell amino silicone-bottom plates were coated with a 0.6 g/L collagen I solution (Sigma-Aldrich) + vacuum	equibiaxial	increase (qPCR, GAPDH)<br>increase (qPCR, GAPDH)<br>increase (qPCR, GAPDH)<br>increase (qPCR, GAPDH)<br>decrease (qPCR, GAPDH)<br>decrease (qPCR, GAPDH)	3.7 (FC)*<br>2.7 (FC)*<br>4.5 (FC)*<br>7.0 (FC)*<br>0.3 (FC)*<br>0.3 (FC)*	n.a.	n.a.
Wang et al. (2019a)	OCN	*BGLAP*	hPDLSC (14-16/n.g., PM, dig and limited dilution, P3, 5×10^5)	dynamic	1.0Hz for 12h	10%	Flexcell FX-5000 + Flexcell amino silicone-bottom plates were coated with a 0.6 g/L collagen I solution (Sigma-Aldrich) + vacuum	equibiaxial	increase (qPCR, GAPDH)	5.8 (FC)*	n.g.	n.g.
Wang et al. (2019a)	RNA-Seq	RNA-SEQ	hPDLSC (14-16/n.g., PM, dig and limited dilution, P3, 5×10^5)	dynamic	1.0Hz for 12h	10%	Flexcell FX-5000 + Flexcell amino silicone-bottom plates were coated with a 0.6 g/L collagen I solution (Sigma-Aldrich) + vacuum	equibiaxial	Illumina Hiseq. 4000 with focus on circRNA species	n.a.	n.a.	n.a.
Wang et al. (2019a)	RUNX2	*RUNX2*	hPDLSC (14-16/n.g., PM, dig and limited dilution, P3, 5×10^5)	dynamic	1.0Hz for 12h	10%	Flexcell FX-5000 + Flexcell amino silicone-bottom plates were coated with a 0.6 g/L collagen I solution (Sigma-Aldrich) + vacuum	equibiaxial	increase (qPCR, GAPDH)	2.5 (FC)*	n.g.	n.g.
Wang et al. (2019a)	SP7	*SP7*	hPDLSC (14-16/n.g., PM, dig and limited dilution, P3, 5×10^5)	dynamic	1.0Hz for 12h	10%	Flexcell FX-5000 + Flexcell amino silicone-bottom plates were coated with a 0.6 g/L collagen I solution (Sigma-Aldrich) + vacuum	equibiaxial	increase (qPCR, GAPDH)	4.5 (FC)*	n.g.	n.g.
Wang et al. (2019b)	Cbfα1	*RUNX2*	hPDLCs (18-30/n.g., M, dig, P2-5, confluence)	dynamic	**0.1Hz** (6cyc/min, 5s on and 5s off) qPCR for 12h, 24h, 48h; WB for 24h, 48h	12%	Flexcell FX-5000TM + Bioflex plates + vaccum	equibiaxial	increase (qPCR, β-actin)	48h: 1.3 (FC)†	increase (WB, GAPDH)	48h: no quantitative information is given
Wang et al. (2019b)	COL-1	*COL1A1*	hPDLCs (18-30/n.g., M, dig, P2-5, confluence)	dynamic	**0.1Hz** (6cyc/min, 5s on and 5s off) qPCR for 12h, 24h, 48h; WB for 24h, 48h	12%	Flexcell FX-5000TM + Bioflex plates + vaccum	equibiaxial	increase (qPCR, β-actin)	48h: 1.6 (FC)†	increase (WB, GAPDH)	48h: no quantitative information is given
Wang et al. (2019b)	OCN	*BGLAP*	hPDLCs (18-30/n.g., M, dig, P2-5, confluence)	dynamic	**0.1Hz** (6cyc/min, 5s on and 5s off) qPCR for 12h, 24h, 48h; WB for 24h, 48h	12%	Flexcell FX-5000TM + Bioflex plates + vaccum	equibiaxial	increase (qPCR, β-actin)	48h: 1.3 (FC)†	increase (WB, GAPDH)	24h: no quantitative information is given

[a] Entry given as reported in the study.
[b] All official gene symbols come from the HUGO Gene Nomenclature Committee (HGNC; URL: https://www.genenames.org) after checking specificity of primers with Primer-BLAST.
[c] Gender/Sex of donors: "M" – male, "F" – female; Tooth type: "PM" – premolar, "M" – molar; Cell density: given in cells/well if not otherwise mentioned.
[d] Frequencies labeled bold orange were converted to hertz (Hz) according to its definition using the information reported in the study (in brackets)
[e] Force type deduced from the description of the force apparatus given by the authors.
[f] Gene and protein expression: 1. conclusion of change (increase, decrease…) was given according to the defined criteria in Figure 2; 2. different markers to describe the amount of change; † Information derived from figures using Engauge Digitizer; *Folds calculated by measuring the graphs, without using the Engauge Digitizer; No makers: Information derived from figures by description in the articles

Reference	Gene/ Analyte[a]	Official gene symbol / abbreviation[b]	Cell (age/gender of donors, tooth type, isolation method, passages used, cell density)[a,c]	Force type (stat./ dyn.)[a]	Force duration and frequency[d]	Force magnitude[a]	Force apparatus[a]	Force type: equibiaxial or uniaxial[e]	Gene expression: Increase, decrease, no change (method w/ reference gene); Methods: qPCR, sqPCR, Northern blot[f]	Gene expression: When it reaches peak and peak's magnitude (fold change; times or ratio; unclear = ?)[f]	Protein expression: Increase, decrease, no change (method w/ reference); Methods: ELISA, WB, RIA, EMSA, IF[f]	Protein expression: When it reaches peak and peak's magnitude (times or ratio; unclear = ?)[f]
Wang et al. (2019b)	OSX	*SP7*	hPDLCs (18-30/n.g., M, dig, P2-5, confluence)	dynamic	**0.1Hz** (6cyc/min, 5s on and 5s off) qPCR for 12h, 24h, 48h; WB for 24h, 48h	12%	Flexcell FX-5000TM + Bioflex plates + vaccum	equibiaxial	increase followed by plateau (qPCR, β-actin)	24h...48h: 1.5 (FC)†	increase (WB, GAPDH)	48h: no quantitative information is given
Wang et al. (2019b)	TAZ	*TAZ*	hPDLCs (18-30/n.g., M, dig, P2-5, confluence)	dynamic	**0.1Hz** (6cyc/min, 5s on and 5s off) qPCR for 12h, 24h, 48h; WB for 24h, 48h	12%	Flexcell FX-5000TM + Bioflex plates + vaccum	equibiaxial	increase (qPCR, β-actin)	48h: (FC)†	increase (WB, GAPDH)	48h: no quantitative information is given
Wei et al. (2014)	ARRAY	ARRAY	hPDLSCs cells (12-16/ n.g., PM, dig, P2-3, 80% confluence)	dynamic	1.0Hz for 12h	10%	Flexcell FX-5000 Tension system + Flexcell Amino silicone bottomed plates coated with 0.6mg/mL collagen I solution (Sigma Aldrich) + vacuum	equibiaxial	Paraflo™ miRNA Microarray Assay			
Wei et al. (2014)	BSP	*IBSP*	hPDLSCs cells (12-16/ n.g., PM, dig, P2-3, 80% confluence)	dynamic	1.0Hz for 12h	10%	Flexcell FX-5000 Tension system + Flexcell Amino silicone bottomed plates coated with 0.6mg/mL collagen I solution (Sigma Aldrich) + vacuum	equibiaxial	increase (qPCR, GAPDH)	2.8 (ratio)*	increase (WB, β-actin)	no quantitative information is given
Wei et al. (2014)	OCN	*BGLAP*	hPDLSCs cells (12-16/ n.g., PM, dig, P2-3, 80% confluence)	dynamic	1.0Hz for 12h	10%	Flexcell FX-5000 Tension system + Flexcell Amino silicone bottomed plates coated with 0.6mg/mL collagen I solution (Sigma Aldrich) + vacuum	equibiaxial	increase (qPCR, GAPDH)	2.1 (ratio)*	increase (WB, β-actin)	no quantitative information is given
Wei et al. (2014)	Runx2	*RUNX2*	hPDLSCs cells (12-16/ n.g., PM, dig, P2-3, 80% confluence)	dynamic	1.0Hz for 12h	10%	Flexcell FX-5000 Tension system + Flexcell Amino silicone bottomed plates coated with 0.6mg/mL collagen I solution (Sigma Aldrich) + vacuum	equibiaxial	increase (qPCR, GAPDH)	1.8 (ratio)*	increase (WB, β-actin)	no quantitative information is given
Wei et al. (2015)	ACVR2B	*ACVR2B*	hPDLSCs cells (10-14/n.g., n.g., dig, P3, 80% confluence)	dynamic	1.0 Hz for 6h, 12h, 24h, 48h	10%	Flexcell FX-5000 Tension system + Flexcell Amino silicone bottomed plates coated with 0.6mg/mL collagen I solution (Sigma Aldrich) + vacuum	equibiaxial	n.g.	n.g.	decrease (WB, β-actin)	24h: 0.6 (rel)* / 0.7 (ratio-calc)
Wei et al. (2015)	ALP	*ALPP*	hPDLSCs cells (10-14/n.g., n.g., dig, P3, 80% confluence)	dynamic	1.0Hz for 6h, 12h, 24h, 48h	10%	Flexcell FX-5000 Tension system + Flexcell Amino silicone bottomed plates coated with 0.6mg/mL collagen I solution (Sigma Aldrich) + vacuum	equibiaxial	n.g.	n.g.	increase (ALP activity)	48h: 0.6 [Sigma unit/(min * mg protein)]* / 11.0 (ratio-calc)
Wei et al. (2015)	OCN	*BGLAP*	hPDLSCs cells (10-14/n.g., n.g., dig, P3, 80% confluence)	dynamic	1.0Hz for 6h, 12h, 24h, 48h	10%	Flexcell FX-5000 Tension system + Flexcell Amino silicone bottomed plates coated with 0.6mg/mL collagen I solution (Sigma Aldrich) + vacuum	equibiaxial	increase (qPCR, GAPDH)	24h: 3.5 (FC)*	n.g.	n.g.
Wei et al. (2015)	Runx2	*RUNX2*	hPDLSCs cells (10-14/n.g., n.g., dig, P3, 80% confluence)	dynamic	1.0Hz for 6h, 12h, 24h, 48h	10%	Flexcell FX-5000 Tension system + Flexcell Amino silicone bottomed plates coated with 0.6mg/mL collagen I solution (Sigma Aldrich) + vacuum	equibiaxial	increase (qPCR, GAPDH)	48h: 1.8 (FC)*	n.g.	n.g.
Wescott et al. (2007)	ARRAY	ARRAY	hPDL fibroblasts (n.g./n.g., PM, exp, P4, 3×10^5)	dynamic	**0.01Hz (1/96Hz)** (intermittent deformation of 12% for 6s every 90s) for 6h, 12h, 24h	12%	Flexercell FX-4000 Strain Unit + six-well, 35-mm flexible-bottomed Uniflex culture plates + vacuum	uniaxial	Osteogenic RT² Profiler PCR Array (Superarray Bioscience Corp.)	too many	n.g.	n.g.
Wolf et al. (2014)	HMGB1	*HMGB1*	hPDL cells (12-14/n.g., PM, n.g., P n.g., confluence)	static	8h	20%	loading platform with cylindrical posts + collagen type I-coated BioFlex plates (Flexcells Int.) + screws (Deschner et al., 2007, Rath-Deschner et al 2009)	equibiaxial	n.g.	n.g.	increase (ELISA)	1.4 (ratio)*
Wu et al. (2015)	mDia1	*DIAPH1*	hPDL cells (n.g./n.g., PM, n.g., P3-6, 95% confluence)	dynamic	0.1Hz for 6h, 24h	10%	Flexercell Tension Plus system FX-5000T + six-well Bioflex plates + vacuum	equibiaxial	n.g.	n.g.	increase followed with platform (WB, GAPDH)	6h...24h: 1.1 (ratio)*
Wu et al. (2015)	Profilin-1	*PFN1*	hPDL cells (n.g./n.g., PM, n.g., P3-6, 95% confluence)	dynamic	0.1Hz for 6h, 24h	10%	Flexercell Tension Plus system FX-5000T + six-well Bioflex plates + vacuum	equibiaxial	n.g.	n.g.	increase (WB, GAPDH)	24h: 2.4 (ratio)*
Wu et al. (2015)	RhoA-GTP	*RHOA*	hPDL cells (n.g./n.g., PM, n.g., P3-6, 95% confluence)	dynamic	0.1Hz for 6h, 24h	10%	Flexercell Tension Plus system FX-5000T + six-well Bioflex plates + vacuum	equibiaxial	n.g.	n.g.	increase (WB, GAPDH)	24h: 4.9 (ratio)*
Wu et al. (2015)	Rho-GDIα	*ARHGDIA*	hPDL cells (n.g./n.g., PM, n.g., P3-6, 95% confluence)	dynamic	0.1Hz for 6h, 24h	10%	Flexercell Tension Plus system FX-5000T + six-well Bioflex plates + vacuum	equibiaxial	n.g.	n.g.	decrease (WB, GAPDH)	24h: 0.5 (ratio)*
Wu et al. (2016)	Caspase 7, Pro- / Caspase 7, cleaved	*CASP7*	hPDL cells (11-13/n.g., PM, exp, P4-6, 70-80% confluence)	dynamic	**0.1Hz** (6cyc/min: 5s stretch and 5s relaxation) for 6h, 24h	20%	Cell Strain Unit (CSU) + elastic silicon rubber membrane + spherical cap (step motor) (Hao et al 2009)	equibiaxial	n.g.	n.g.	Pro-caspase 7: increase (WB, GAPDH)<br>Cleaved caspase 7: increase (WB, GAPDH)	Pro-Caspase 3 @ 24h: 0.3 (rel)† / 21.1 (ratio-calc)<br>Cleaved caspase 7 @ 24h: 0.6 (rel)† / 6.3 (ratio-calc)
Wu et al. (2016)	Caspase 8, cleaved (18kDa)	*CASP8*	hPDL cells (11-13/n.g., PM, exp, P4-6, 70-80% confluence)	dynamic	**0.1Hz** (6cyc/min: 5s stretch and 5s relaxation) for 6h, 24h	20%	Cell Strain Unit (CSU) + elastic silicon rubber membrane + spherical cap (step motor) (Hao et al 2009)	equibiaxial	n.g.	n.g.	increase (WB, GAPDH)	24h: 0.2 (rel)† / 4.9 (ratio-calc)

[a] Entry given as reported in the study.
[b] All official gene symbols come from the HUGO Gene Nomenclature Committee (HGNC; URL: https://www.genenames.org) after checking specificity of primers with Primer-BLAST.
[c] Gender/Sex of donors: "M" – male, "F" – female; Tooth type: "PM" – premolar, "M" – molar; Cell density: given in cells/well if not otherwise mentioned.
[d] Frequencies labeled bold orange were converted to hertz (Hz) according to its definition using the information reported in the study (in brackets)
[e] Force type deduced from the description of the force apparatus given by the authors.
[f] Gene and protein expression: 1. conclusion of change (increase, decrease…) was given according to the defined criteria in Figure 2; 2. different markers to describe the amount of change; † Information derived from figures using Engauge Digitizer; *Folds calculated by measuring the graphs, without using the Engauge Digitizer; No makers: Information derived from figures by description in the articles

Reference	Gene/ Analyte[a]	Official gene symbol / abbreviation[b]	Cell (age/gender of donors, tooth type, isolation method, passages used, cell density)[a,c]	Force type (stat./ dyn.)[a]	Force duration and frequency[d]	Force magnitude[a]	Force apparatus[a]	Force type: equibiaxial or uniaxial[e]	Gene expression: Increase, decrease, no change (method w/ reference gene); Methods: qPCR, sqPCR, Northern blot[f]	Gene expression: When it reaches peak and peak's magnitude (fold change; times or ratio; unclear = ?)[f]	Protein expression: Increase, decrease, no change (method w/ reference); Methods: ELISA, WB, RIA, EMSA, IF[f]	Protein expression: When it reaches peak and peak's magnitude (times or ratio; unclear = ?)[f]
Wu et al. (2016)	Caspase 8, cleaved (43/45kDa)	*CASP8*	hPDL cells (11-13/n.g., PM, exp, P4-6, 70-80% confluence)	dynamic	**0.1Hz** (6cyc/min: 5s stretch and 5s relaxation) for 6h, 24h	20%	Cell Strain Unit (CSU) + elastic silicon rubber membrane + spherical cap (step motor) (Hao et al 2009)	equibiaxial	n.g.	n.g.	increase (WB, GAPDH)	24h: 0.5 (rel)† / 8.3 (ratio-calc)
Wu et al. (2016)	Caspase 8, Pro-	*CASP8*	hPDL cells (11-13/n.g., PM, exp, P4-6, 70-80% confluence)	dynamic	**0.1Hz** (6cyc/min: 5s stretch and 5s relaxation) for 6h, 24h	20%	Cell Strain Unit (CSU) + elastic silicon rubber membrane + spherical cap (step motor) (Hao et al 2009)	equibiaxial	n.g.	n.g.	increase (WB, GAPDH)	24h: 0.6 (rel)† / 8.4 (ratio-calc)
Wu et al. (2016)	Caspase 9, Pro- / Caspase 9, cleaved	*CASP9*	hPDL cells (11-13/n.g., PM, exp, P4-6, 70-80% confluence)	dynamic	**0.1Hz** (6cyc/min: 5s stretch and 5s relaxation) for 6h, 24h	20%	Cell Strain Unit (CSU) + elastic silicon rubber membrane + spherical cap (step motor) (Hao et al 2009)	equibiaxial	n.g.	n.g.	Pro-caspase 9: increase (WB, GAPDH)<br>Cleaved caspase 9: increase (WB, GAPDH)	Pro-caspase 9 @ 24h: 0.8 (rel)† 31.8 (ratio-calc)<br>Cleaved caspase 9 @ 24h: 0.7 (rel)† / 7.7 (ratio-calc)
Wu et al. (2016)	Caspase-3, cleaved	*CASP3*	hPDL cells (11-13/n.g., PM, exp, P4-6, 70-80% confluence)	dynamic	**0.1Hz** (6cyc/min: 5s stretch and 5s relaxation) for 6h, 24h	20%	Cell Strain Unit (CSU) + elastic silicon rubber membrane + spherical cap (step motor) (Hao et al 2009)	equibiaxial	n.g.	n.g.	increase (WB, GAPDH)	24h: 0.1 (rel)* / 1.7 (ratio-calc)
Wu et al. (2017)	ARRAY	ARRAY	hPDL cells (11/F, 12/F, 13/F, PM, exp, P4, confluence)	dynamic	**0.1Hz** (6cyc/min: 5s stretch and 5s relaxation) qPCR for 24h; WB for 6h, 24h	1%, 10%, 20%	Cell Strain Unit (CSU) + elastic silicon rubber membrane + spherical cap (step motor) (Hao et al 2009)	equibiaxial	Human Cytoskeleton Regulators RT[2] ProfilerTM PCR Array (PAHS-088, SABiosciences, Frederick, MD)	ARRAY	n.a.	n.a.
Wu et al. (2017)	CDC42EP2	*CDC42EP2*	hPDL cells (11/F, 12/F, 13/F, PM, exp, P4, confluence)	dynamic	**0.1Hz** (6cyc/min: 5s stretch and 5s relaxation) qPCR for 24h; WB for 6h, 24h	1%, 10%, 20%	Cell Strain Unit (CSU) + elastic silicon rubber membrane + spherical cap (step motor) (Hao et al 2009)	equibiaxial	n.a.	n.a.	decrease (WB, GAPDH)	no quantitative information is given
Wu et al. (2017)	STMN1	*STMN1*	hPDL cells (11/F, 12/F, 13/F, PM, exp, P4, confluence)	dynamic	**0.1Hz** (6cyc/min: 5s stretch and 5s relaxation) qPCR for 24h; WB for 6h, 24h	1%, 10%, 20%	Cell Strain Unit (CSU) + elastic silicon rubber membrane + spherical cap (step motor) (Hao et al 2009)	equibiaxial	n.a.	n.a.	decrease (WB, GAPDH)	no quantitative information is given
Wu et al. (2017)	WASL	*WASL*	hPDL cells (11/F, 12/F, 13/F, PM, exp, P4, confluence)	dynamic	**0.1Hz** (6cyc/min: 5s stretch and 5s relaxation) for 24h	1%, 10%, 20%	Cell Strain Unit (CSU) + elastic silicon rubber membrane + spherical cap (step motor) (Hao et al 2009)	equibiaxial	n.a.	n.a.	increase (WB, GAPDH)	no quantitative information is given
Wu et al. (2019a)	BSP	*IBSP*	hPDLCs (14-25/n.g., M, exp, P3-6, 70-80% confluence)	dynamic	0.1Hz for 24h	10%	Flexcell FX-5000TM + six-well Bioflex plates coated with type I collagen + vaccum	equibiaxial	increase (qPCR, GAPDH)	1.6 (FC)†	n.g.	n.g.
Wu et al. (2019a)	CAP	*HACD1*	hPDLCs (14-25/n.g., M, exp, P3-6, 70-80% confluence)	dynamic	0.1Hz for 24h	10%	Flexcell FX-5000TM + six-well Bioflex plates coated with type I collagen + vaccum	equibiaxial	increase (qPCR, GAPDH)	2.4 (FC)†	n.g.	n.g.
Wu et al. (2019a)	CEMP1	*AMDHD2*	hPDLCs (14-25/n.g., M, exp, P3-6, 70-80% confluence)	dynamic	0.1Hz for 24h	10%	Flexcell FX-5000TM + six-well Bioflex plates coated with type I collagen + vaccum	equibiaxial	increase (qPCR, GAPDH)	2.0 (FC)†	increase (WB, GAPDH)	no quantitative information is given
Wu et al. (2019a)	CTGF	*CCN2*	hPDLCs (14-25/n.g., M, exp, P3-6, 70-80% confluence)	dynamic	0.1Hz for 24h	10%	Flexcell FX-5000TM + six-well Bioflex plates coated with type I collagen + vaccum	equibiaxial	decrease (qPCR, GAPDH)	0.8 (FC)†	n.g.	n.g.
Wu et al. (2019a)	DVL2	*DVL2*	hPDLCs (14-25/n.g., M, exp, P3-6, 70-80% confluence)	dynamic	0.1Hz for 24h	10%	Flexcell FX-5000TM + six-well Bioflex plates coated with type I collagen + vaccum	equibiaxial	increase (qPCR, GAPDH)	3.1 (FC)†	n.g.	n.g.
Wu et al. (2019a)	GDF5	*GDF5*	hPDLCs (14-25/n.g., M, exp, P3-6, 70-80% confluence)	dynamic	0.1Hz for 24h	10%	Flexcell FX-5000TM + six-well Bioflex plates coated with type I collagen + vaccum	equibiaxial	increase (qPCR, GAPDH)	4.7 (FC)†	n.g.	n.g.
Wu et al. (2019a)	GLI2	*GLI2*	hPDLCs (14-25/n.g., M, exp, P3-6, 70-80% confluence)	dynamic	0.1Hz for 24h	10%	Flexcell FX-5000TM + six-well Bioflex plates coated with type I collagen + vaccum	equibiaxial	increase (qPCR, GAPDH)	1.6 (FC)†	n.g.	n.g.
Wu et al. (2019a)	LATS1	*LATS1*	hPDLCs (14-25/n.g., M, exp, P3-6, 70-80% confluence)	dynamic	0.1Hz for 24h	10%	Flexcell FX-5000TM + six-well Bioflex plates coated with type I collagen + vaccum	equibiaxial	increase (qPCR, GAPDH)	1.5 (FC)†	n.g.	n.g.
Wu et al. (2019a)	LIMD1	*LIMD1*	hPDLCs (14-25/n.g., M, exp, P3-6, 70-80% confluence)	dynamic	0.1Hz for 24h	10%	Flexcell FX-5000TM + six-well Bioflex plates coated with type I collagen + vaccum	equibiaxial	increase (qPCR, GAPDH)	2.9 (FC)†	n.g.	n.g.
Wu et al. (2019a)	MSX2	*MSX2*	hPDLCs (14-25/n.g., M, exp, P3-6, 70-80% confluence)	dynamic	0.1Hz for 24h	10%	Flexcell FX-5000TM + six-well Bioflex plates coated with type I collagen + vaccum	equibiaxial	increase (qPCR, GAPDH)	1.5 (FC)†	n.g.	n.g.
Wu et al. (2019a)	OCN	*BGLAP*	hPDLCs (14-25/n.g., M, exp, P3-6, 70-80% confluence)	dynamic	0.1Hz for 24h	10%	Flexcell FX-5000TM + six-well Bioflex plates coated with type I collagen + vaccum	equibiaxial	increase (qPCR, GAPDH)	2.1 (FC)†	n.g.	n.g.

[a] Entry given as reported in the study.
[b] All official gene symbols come from the HUGO Gene Nomenclature Committee (HGNC; URL: https://www.genenames.org) after checking specificity of primers with Primer-BLAST.
[c] Gender/Sex of donors: "M" – male, "F" – female; Tooth type: "PM" – premolar, "M" – molar; Cell density: given in cells/well if not otherwise mentioned.
[d] Frequencies labeled bold orange were converted to hertz (Hz) according to its definition using the information reported in the study (in brackets)
[e] Force type deduced from the description of the force apparatus given by the authors.
[f] Gene and protein expression: 1. conclusion of change (increase, decrease…) was given according to the defined criteria in Figure 2; 2. different markers to describe the amount of change; † Information derived from figures using Engauge Digitizer; *Folds calculated by measuring the graphs, without using the Engauge Digitizer; No makers: Information derived from figures by description in the articles

Reference	Gene/ Analyte[a]	Official gene symbol / abbreviation[b]	Cell (age/gender of donors, tooth type, isolation method, passages used, cell density)[a,c]	Force type (stat./ dyn.)[a]	Force duration and frequency[d]	Force magnitude[a]	Force apparatus[a]	Force type: equibiaxial or uniaxial[e]	Gene expression: Increase, decrease, no change (method w/ reference gene); Methods: qPCR, sqPCR, Northern blot[f]	Gene expression: When it reaches peak and peak's magnitude (fold change; times or ratio; unclear = ?)[f]	Protein expression: Increase, decrease, no change (method w/ reference); Methods: ELISA, WB, RIA, EMSA, IF[f]	Protein expression: When it reaches peak and peak's magnitude (times or ratio; unclear = ?)[f]
Wu et al. (2019a)	RUNX2	*RUNX2*	hPDLCs (14-25/n.g., M, exp, P3-6, 70-80% confluence)	dynamic	0.1Hz for 24h	10%	Flexcell FX-5000TM + six-well Bioflex plates coated with type I collagen + vaccum	equibiaxial	increase (qPCR, GAPDH)	1.6 (FC)†	increase (WB, GAPDH)	no quantitative information is given
Wu et al. (2019a)	SATB2	*SATB2*	hPDLCs (14-25/n.g., M, exp, P3-6, 70-80% confluence)	dynamic	0.1Hz for 24h	10%	Flexcell FX-5000TM + six-well Bioflex plates coated with type I collagen + vaccum	equibiaxial	increase (qPCR, GAPDH)	1.7 (FC)†	n.g.	n.g.
Wu et al. (2019a)	SPP1	*SPP1*	hPDLCs (14-25/n.g., M, exp, P3-6, 70-80% confluence)	dynamic	0.1Hz for 24h	10%	Flexcell FX-5000TM + six-well Bioflex plates coated with type I collagen + vaccum	equibiaxial	increase (qPCR, GAPDH)	2.3 (FC)†	increase (WB, GAPDH)	no quantitative information is given
Wu et al. (2019a)	TEAD1	*TEAD1*	hPDLCs (14-25/n.g., M, exp, P3-6, 70-80% confluence)	dynamic	0.1Hz for 24h	10%	Flexcell FX-5000TM + six-well Bioflex plates coated with type I collagen + vaccum	equibiaxial	increase (qPCR,GAPDH)	1.3 (FC)†	n.g.	n.g.
Wu et al. (2019a)	TEAD2	*TEAD2*	hPDLCs (14-25/n.g., M, exp, P3-6, 70-80% confluence)	dynamic	0.1Hz for 24h	10%	Flexcell FX-5000TM + six-well Bioflex plates coated with type I collagen + vaccum	equibiaxial	increase (qPCR, GAPDH)	2.2 (FC)†	n.g.	n.g.
Wu et al. (2019a)	WTIP	*WTIP*	hPDLCs (14-25/n.g., M, exp, P3-6, 70-80% confluence)	dynamic	0.1Hz for 24h	10%	Flexcell FX-5000TM + six-well Bioflex plates coated with type I collagen + vaccum	equibiaxial	increase (qPCR, GAPDH)	1.8 (FC)†	n.g.	n.g.
Wu et al. (2019a)	WWTR1	*TAZ*	hPDLCs (14-25/n.g., M, exp, P3-6, 70-80% confluence)	dynamic	0.1Hz for 24h	10%	Flexcell FX-5000TM + six-well Bioflex plates coated with type I collagen + vaccum	equibiaxial	increase (qPCR, GAPDH)	3.7 (FC)†	n.g.	n.g.
Wu et al. (2019a)	YAP1	*YAP1*	hPDLCs (14-25/n.g., M, exp, P3-6, 70-80% confluence)	dynamic	0.1Hz for 24h	10%	Flexcell FX-5000TM + six-well Bioflex plates coated with type I collagen + vaccum	equibiaxial	increase (qPCR, GAPDH)	2.2 (FC)†	n.g.	n.g.
Wu et al. (2019b)	Caspase 3, Pro- / Caspase 3, cleaved	*CASP3*	hPDLFs (11-13/n.g., PM, exp, P4-6, confluence)	dynamic	**0.1Hz** (6cyc/min: 5s stretch and 5s relaxation) for 6h, 24h	20%	Cell Strain Unit (CSU) + elastic silicon rubber membrane + spherical cap (step motor) (Hao et al 2009)	equibiaxial	n.g.	n.g.	Pro-caspase-3 (35 kDa): increase (WB, GAPDH)<br>Cleaved caspase-3 (ca. 17 kDa): increase (WB, GAPDH)<br>Cleaved caspase-3: increase (colorimetric assay)	Pro-caspase-3: 12h: 0.6 (rel)* / 1.2 (ratio-calc)<br>Cleaved caspase-3: WB @ 24h: 0.1 (rel)* / 3.3 (ratio-calc)<br>Cleaved caspase-3: colorimetric assay @ 24h: 1.5 (ratio)*
Wu et al. (2019b)	Caspase 5, Pro- / Caspase 5, cleaved	*CASP5*	hPDLFs (11-13/n.g., PM, exp, P4-6, confluence)	dynamic	**0.1Hz** (6cyc/min: 5s stretch and 5s relaxation) for 6h, 24h	20%	Cell Strain Unit (CSU) + elastic silicon rubber membrane + spherical cap (step motor) (Hao et al 2009)	equibiaxial	n.g.	n.g.	Pro-caspase-5 (45 kDa): decrease (WB, GAPDH)<br>Cleaved caspase-5 (20 kDa): increase (WB, GAPDH)<br>Cleaved caspase-5: increase followed by plateau (colorimetric assay)	Pro-caspase-5: 24h: 0.2 (rel)† / 0.6 (ratio-calc)<br>Cleaved caspase-5: WB @ 24h: 0.2 (rel)† / 15.0 (ratio-calc)<br>Cleaved caspase-5: colorimetric assay @ 6h…24h: 1.7 (ratio)*
Xu et al. (2011)	ARRAY	ARRAY	hPDL cells (12/F, PM, exp, P4, confluence)	dynamic	**0.1Hz** (6cyc/min) for 6h, 24h	20%	Cell Strain Unit (CSU) + elastic silicon rubber membrane + spherical cap (step motor) (Hao et al 2009)	equibiaxial	Human Apoptosis RT[2] Profiler PCR Array (PAHS-012; Superarray) with *B2M*, *GAPDH* and *ACTB* as reference genes	too many	n.g.	n.g.
Xu et al. (2012)	Cx43	*GJA1*	hPDL cells (12/F, 15/M, PM, exp, P4-6, confluence)	dynamic	**0.1Hz** (6cyc/min) for 0.5h, 1h, 24h	1%, 10%, 20%	Cell Strain Unit (CSU) + elastic silicon rubber membrane + spherical cap (step motor) (Hao et al 2009)	equibiaxial	1% strain: temporary decrease followed by temporary increase (qPCR, β-actin)<br>10% strain: temporary decrease (qPCR, β-actin)<br>20% strain: decrease followed by plateau then increase (qPCR, β-actin)	1% strain lowest @ 0.5h: 0.2 (rel)† / 0.2 (ratio-calc)<br>1% strain highest @ 1h: 2.1 (rel)† / 1.9 (ratio-calc)<br>10% strain lowest@ 0.5h: 0.2 (rel)† / 0.2 (ratio-calc)<br>20% strain lowest @ 0.5h…1h: 0.4 (rel)† / 0.4 (ratio-calc)<br>20% strain highest @ 24h: 1.8 (rel)† / 2 (ratio-calc)	n.g.	n.g.
Xu et al. (2015)	α-SMA	*ACTA2*	hPDL cells (n.g./n.g., PM, exp, P3-5, 80% confluence)	dynamic	0.5Hz qPCR for 6h; ELISA for 0h, 1h, 3h, 6h, 12h	**0.2%** (2000μstrain), **0.4%** (4000μstrain)	a uniaxial four-point bending system (developed at Sichuan University, patents CN2534576 and CN1425905)	uniaxial	2000μstrain: increase (qPCR, GAPDH)<br>4000μstrain: increase (qPCR, GAPDH)	2000 μstrain: 1.2 (ratio)*<br>4000 μstrain: 2.6 (ratio)*	2000 μstrain: increase (ELISA)<br>4000 μstrain: increase (ELISA)	2000 μstrain @ 12h: 348.5 (pg/ml)* / 1.4 (ratio-calc)<br>4000 μstrain @ 12h: 393.9 (pg/ml)* / 1.6 (ratio-calc)
Xu et al. (2017)	Periostin	*POSTN*	hPDL cells (n.g./n.g., PM and M, exp, P3, n.g.)	dynamic	0.5Hz for 0h, 12h, 24h, 48h	10%	Flexcell FX-5000 Tension system + Flexcell Amino silicone bottomed plates coated with 0.6mg/mL collagen I solution (Sigma Aldrich) + vacuum (Wei et al 2014)	equibiaxial	increase (qPCR, GAPDH)	48h: 3.3 (FC)*	increase (WB, α-tubulin)	48h: 3.6 (ratio)*
Xu et al. (2017)	TGF-β	*TGFB1*	hPDL cells (n.g./n.g., PM and M, exp, P3, n.g.)	dynamic	0.5Hz for 0h, 12h, 24h, 48h	10%	Flexcell FX-5000 Tension system + Flexcell Amino silicone bottomed plates coated with 0.6mg/mL collagen I solution (Sigma Aldrich) + vacuum (Wei et al 2014)	equibiaxial	increase (qPCR, GAPDH)	48h: 3.5 (FC)*	increase (WB, α-tubulin)	no quantitative information is given
Yamaguchi and Shimizu (1994)	ALP	*ALPP*	hPDL fibroblasts (12/M, 10/M, 11/F, 3 donors, PM, exp, donor#1 P4, donor#2 P6, donor#3 P4, confluent)	dynamic	**0.1Hz** (6cyc/min: 5s elongation and 5s relaxation) for 3d	24%	Flexercell strain unit + flexible-bottom culture plates coated with type I collagen (Flexcell) + vacuum (Banes et al 1985)	equibiaxial	n.g.	n.g.	donor 1: decrease (ALP activity)<br>donor 2: decrease (ALP activity)<br>donor 3: decrease (ALP activity)	donor 1: 10.3 (mU/10^5 cells) / 0.6 (ratio-calc)<br>donor 2: 10.0 (mU/10^5 cells) / 0.6 (ratio-calc)<br>donor 3: 10.6 (mU/10^5 cells) / 0.6 (ratio-calc)
Yamaguchi et al. (1994)	PGE_2	PGE_2	hPDL fibroblasts (12/M, 19/F, 11/M, PM, exp, P4, 1×10^5)	dynamic	**0.1Hz** (6cyc/min: 5s on and 5s off) for 1d, 3d, 5d	18%	Flexercell strain unit + flexible-bottom culture plates coated with type I collagen (Flexcell) + vacuum (Banes et al 1985)	equibiaxial	n.a.	n.a.	increase (RIA)	5d: 8.9 (ng/10^6 cells)* / 17.8 (ratio-calc)
Yamaguchi et al. (1994)	PGE_2	PGE_2	hPDL fibroblasts (12/M, 19/F, 11/M, PM, exp, P4, 1×10^5)	dynamic	**0.1Hz** (6cyc/min: 5s on and 5s off) for 5d	9%, 12%, 15%, 18%, 21%, 24%	Flexercell strain unit + flexible-bottom culture plates coated with type I collagen (Flexcell) + vacuum (Banes et al 1985)	equibiaxial	n.a.	n.a.	increase (RIA)	24%: 14 (ng/10^6 cells)* / 28 (ratio-calc)

[a] Entry given as reported in the study.
[b] All official gene symbols come from the HUGO Gene Nomenclature Committee (HGNC; URL: https://www.genenames.org) after checking specificity of primers with Primer-BLAST.
[c] Gender/Sex of donors: "M" – male, "F" – female; Tooth type: "PM" – premolar, "M" – molar; Cell density: given in cells/well if not otherwise mentioned.
[d] Frequencies labeled bold orange were converted to hertz (Hz) according to its definition using the information reported in the study (in brackets)
[e] Force type deduced from the description of the force apparatus given by the authors.
[f] Gene and protein expression: 1. conclusion of change (increase, decrease…) was given according to the defined criteria in Figure 2; 2. different markers to describe the amount of change; † Information derived from figures using Engauge Digitizer; *Folds calculated by measuring the graphs, without using the Engauge Digitizer; No makers: Information derived from figures by description in the articles

Reference	Gene/ Analyte[a]	Official gene symbol / abbreviation[b]	Cell (age/gender of donors, tooth type, isolation method, passages used, cell density)[a,c]	Force type (stat./ dyn.)[a]	Force duration and frequency[d]	Force magnitude[a]	Force apparatus[a]	Force type: equibiaxial or uniaxial[e]	Gene expression: Increase, decrease, no change (method w/ reference gene); Methods: qPCR, sqPCR, Northern blot[f]	Gene expression: When it reaches peak and peak's magnitude (fold change; times or ratio; unclear = ?)[f]	Protein expression: Increase, decrease, no change (method w/ reference); Methods: ELISA, WB, RIA, EMSA, IF[f]	Protein expression: When it reaches peak and peak's magnitude (times or ratio; unclear = ?)[f]
Yamaguchi et al. (1996)	ALP	*ALPP*	hPDL fibroblasts (12/M, PM, exp, P n.g., 1×10^5)	dynamic	**0.1Hz** (6cyc/min: 5s on and 5s off) for 1d, 3d, 5d	24%	Flexercell strain unit + flexible-bottom culture plates coated with type I collagen (Flexcell) + vacuum (Banes et al 1990)	equibiaxial	n.g.	n.g.	decrease followed by plateau (ALP activity)	3d…5d: 10.7 (mU/10^5 cells)* / 0.6 (ratio-calc)
Yamaguchi et al. (1996)	ALP	*ALPP*	hPDL fibroblasts (12/M, PM, exp, P n.g., 1×10^5)	dynamic	**0.1Hz** (6cyc/min: 5s on and 5s off) ALP activity for 5d; sqPCR for 3d	ALP activity for 9%, 12%, 15%, 18%, 21%, 24%; sqPCR for 12%, 24%	Flexercell strain unit + flexible-bottom culture plates coated with type I collagen (Flexcell) + vacuum (Banes et al 1990)	equibiaxial	decrease (Northern blot, β-actin)	no quantitative information is given	decrease (ALP activity)	24%: 9.4 (mU/10^5 cells)* / 0.5 (ratio-calc)
Yamaguchi et al. (1997)	PAI-1	*SERPINE1*	hPDL fibroblasts (12/M, 10/M, 11/F, exp, 3 donors, P unclear, confluent)	static	5d	18%	Flexercell strain unit + flexible-bottom culture plates coated with type I collagen (Flexcell) + vacuum (Banes et al 1985)	equibiaxial	no change (sqPCR, GAPDH)	no quantitative information is given	n.g.	n.g.
Yamaguchi et al. (1997)	Plasminogen activator	*PLAT; PLAU*	hPDL fibroblasts (12/M, 10/M, 11/F, exp, 3 donors, P unclear, confluent)	static	1d, 3d, 5d	18%	Flexercell strain unit + flexible-bottom culture plates coated with type I collagen (Flexcell) + vacuum (Banes et al 1985)	equibiaxial	n.g.	n.g.	increase (PA activity, photometric)	5d: 7.5 (mU/10^5 cells)* / 2.2 (ratio-calc)
Yamaguchi et al. (1997)	Plasminogen activator	*PLAT; PLAU*	hPDL fibroblasts (12/M, 10/M, 11/F, exp, 3 donors, P unclear, confluent)	static	5d	9%, 18%	Flexercell strain unit + flexible-bottom culture plates coated with type I collagen (Flexcell) + vacuum (Banes et al 1985)	equibiaxial	n.g.	n.g.	increase (PA activity, photometric)	18%: 7.9 (mU/10^5 cells)* / 2.2 (ratio-calc)
Yamaguchi et al. (1997)	tPA	*PLAT*	hPDL fibroblasts (12/M, 10/M, 11/F, exp, 3 donors, P unclear, confluent)	static	5d	18%	Flexercell strain unit + flexible-bottom culture plates coated with type I collagen (Flexcell) + vacuum (Banes et al 1985)	equibiaxial	increase (sqPCR, GAPDH)	no quantitative information is given	increase (WB)	no quantitative information is given
Yamaguchi et al. (1997)	uPA	*PLAU*	hPDL fibroblasts (12/M, 10/M, 11/F, exp, 3 donors, P unclear, confluent)	static	5d	18%	Flexercell strain unit + flexible-bottom culture plates coated with type I collagen (Flexcell) + vacuum (Banes et al 1985)	equibiaxial	not detectable (sqPCR, GAPDH)	no quantitative information is given	not detectable (WB)	no quantitative information is given
Yamaguchi et al. (2002)	ALP	*ALPP*	hPDL fibroblasts (n.g./n.g., PM, n.g., P8, confluent)	dynamic	**0.5Hz** (30cyc/min: 1s on and 1s off) for 30min, 90min, 6h	15%	a strain unit (Flexercell) + type I collagen-coated, silicon membrane) culture plates (Flex I; Flexercell) + vacuum	equibiaxial	temporary decrease (sqPCR, GAPDH)	90min: 0.8 (ratio)*	n.g.	n.g.
Yamaguchi et al. (2002)	c-fos	*FOS*	hPDL fibroblasts (n.g./n.g., PM, n.g., P8, confluent)	dynamic	**0.5Hz** (30cyc/min: 1s on and 1s off) for 30min, 90min, 6h	15%	a strain unit (Flexercell) + type I collagen-coated, silicon membrane) culture plates (Flex I; Flexercell) + vacuum	equibiaxial	increase (sqPCR, GAPDH)	30min: 55.8 (ratio)*	n.g.	n.g.
Yamaguchi et al. (2002)	COL-I	*COL1A1*	hPDL fibroblasts (n.g./n.g., PM, n.g., P8, confluent)	dynamic	**0.5Hz** (30cyc/min: 1s on and 1s off) for 30min, 90min, 6h	15%	a strain unit (Flexercell) + type I collagen-coated, silicon membrane) culture plates (Flex I; Flexercell) + vacuum	equibiaxial	temporary increase (sqPCR, GAPDH)	30min: 1.2 (ratio)*	n.g.	n.g.
Yamaguchi et al. (2002)	COL-III	*COL3A1*	hPDL fibroblasts (n.g./n.g., PM, n.g., P8, confluent)	dynamic	**0.5Hz** (30cyc/min: 1s on and 1s off) for 30min, 90min, 6h	15%	a strain unit (Flexercell) + type I collagen-coated, silicon membrane) culture plates (Flex I; Flexercell) + vacuum	equibiaxial	decrease (sqPCR, GAPDH)	6h: 0.6 (ratio)*	n.g.	n.g.
Yamaguchi et al. (2002)	MGP	*MGP*	hPDL fibroblasts (n.g./n.g., PM, n.g., P8, confluent)	dynamic	**0.5Hz** (30cyc/min: 1s on and 1s off) for 30min, 90min, 6h	15%	a strain unit (Flexercell) + type I collagen-coated, silicon membrane) culture plates (Flex I; Flexercell) + vacuum	equibiaxial	increase (sqPCR, GAPDH)	6h: 1.5 (ratio)*	n.g.	n.g.
Yamaguchi et al. (2002)	ON	*SPARC*	hPDL fibroblasts (n.g./n.g., PM, n.g., P8, confluent)	dynamic	**0.5Hz** (30cyc/min: 1s on and 1s off) for 30min, 90min, 6h	15%	a strain unit (Flexercell) + type I collagen-coated, silicon membrane) culture plates (Flex I; Flexercell) + vacuum	equibiaxial	no change (sqPCR, GAPDH)		n.g.	n.g.
Yamaguchi et al. (2002)	OPN	*SPP1*	hPDL fibroblasts (n.g./n.g., PM, n.g., P8, confluent)	dynamic	**0.5Hz** (30cyc/min: 1s on and 1s off) for 30min, 90min, 6h	15%	a strain unit (Flexercell) + type I collagen-coated, silicon membrane) culture plates (Flex I; Flexercell) + vacuum	equibiaxial	decrease (sqPCR, GAPDH)	6h: 0.7 (ratio)*	n.g.	n.g.
Yamaguchi et al. (2004)	Cathepsin B	*CTSB*	hPDL fibroblasts (15-18/F and 15-18/F, PM, exp, P n.g., 4×10^6)	static	12h	0.28%, 0.95%, 1.72%, 2.50%	Petriperm dish + spheroidal convex template + weight	equibiaxial	n.g.	n.g.	increase (ELISA)	2.5%: 15 (ng/g of cellular protein)* / 2.4 (ratio-calc)
Yamaguchi et al. (2004)	Cathepsin B	*CTSB*	hPDL fibroblasts (15-18/F and 15-18/F, PM, exp, P n.g., 4×10^6)	static	sqPCR for 12h; ELISA for 3h, 6h, 9h, 12h, 24h	2.50%	Petriperm dish + spheroidal convex template + weight	equibiaxial	increase (sqPCR, GAPDH)	no quantitative information is given	increase (ELISA)	12h: 15 (ng/g of cellular protein)* / 1.6 (ratio-calc)
Yamaguchi et al. (2004)	Cathepsin L	*CTSL*	hPDL fibroblasts (15-18/F and 15-18/F, PM, exp, P n.g., 4×10^6)	static	12h	0.28%, 0.95%, 1.72%, 2.50%	Petriperm dish + spheroidal convex template + weight	equibiaxial	n.g.	n.g.	increase (ELISA)	2.5%: 22 (ng/g of cellular protein)* / 3.1 (ratio-calc)
Yamaguchi et al. (2004)	Cathepsin L	*CTSL*	hPDL fibroblasts (15-18/F and 15-18/F, PM, exp, P n.g., 4×10^6)	static	3h, 6h, 9h, 12h, 24h	2.50%	Petriperm dish + spheroidal convex template + weight	equibiaxial	increase (sqPCR, GAPDH)	no quantitative information is given	increase (ELISA)	12h: 20.8 (ng/g of cellular protein)* / 2.7 (ratio-calc)

[a] Entry given as reported in the study.
[b] All official gene symbols come from the HUGO Gene Nomenclature Committee (HGNC; URL: https://www.genenames.org) after checking specificity of primers with Primer-BLAST.
[c] Gender/Sex of donors: "M" – male, "F" – female; Tooth type: "PM" – premolar, "M" – molar; Cell density: given in cells/well if not otherwise mentioned.
[d] Frequencies labeled bold orange were converted to hertz (Hz) according to its definition using the information reported in the study (in brackets)
[e] Force type deduced from the description of the force apparatus given by the authors.
[f] Gene and protein expression: 1. conclusion of change (increase, decrease…) was given according to the defined criteria in Figure 2; 2. different markers to describe the amount of change; † Information derived from figures using Engauge Digitizer; *Folds calculated by measuring the graphs, without using the Engauge Digitizer; No makers: Information derived from figures by description in the articles

Reference	Gene/ Analyte[a]	Official gene symbol / abbreviation[b]	Cell (age/gender of donors, tooth type, isolation method, passages used, cell density)[a,c]	Force type (stat./ dyn.)[a]	Force duration and frequency[d]	Force magnitude[a]	Force apparatus[a]	Force type: equibiaxial or uniaxial[e]	Gene expression: Increase, decrease, no change (method w/ reference gene); Methods: qPCR, sqPCR, Northern blot[f]	Gene expression: When it reaches peak and peak's magnitude (fold change; times or ratio; unclear = ?)[f]	Protein expression: Increase, decrease, no change (method w/ reference); Methods: ELISA, WB, RIA, EMSA, IF[f]	Protein expression: When it reaches peak and peak's magnitude (times or ratio; unclear = ?)[f]
Yamashiro et al. (2007)	ACY1	*ACY1*	hPDL fibroblasts (21/F, 24/F, 17/F, 22/M, n.g., n.g., P5-9, 2×10^5)	dynamic	**0.1Hz** (6cyc/min) for 0.5h, 1h, 2h, 16h	18%	Flexercell Strain Unit + flexible-bottomed culture plates (FLEX II) + vacuum (Myokai et al 2003; Banes et al 1990)	equibiaxial	increase (qPCR, β-actin)	duration n.g.: 2.7 (ratio)*	n.g.	n.g.
Yamashiro et al. (2007)	ADRB2	*ADRB2*	hPDL fibroblasts (21/F, 24/F, 17/F, 22/M, n.g., n.g., P5-9, 2×10^5)	dynamic	**0.1Hz** (6cyc/min) for 0.5h, 1h, 2h, 16h	18%	Flexercell Strain Unit + flexible-bottomed culture plates (FLEX II) + vacuum (Myokai et al 2003; Banes et al 1990)	equibiaxial	increase (qPCR, β-actin)	duration n.g.: 1.6 (ratio)*	n.g.	n.g.
Yamashiro et al. (2007)	ARRAY	ARRAY	hPDL fibroblasts (21/F, 24/F, 17/F, 22/M, n.g., n.g., P5-9, 2×10^5)	dynamic	**0.1Hz** (6cyc/min) for 0.5h, 1h, 2h, 16h	18%	Flexercell Strain Unit + flexible-bottomed culture plates (FLEX II) + vacuum (Myokai et al 2003; Banes et al 1990)	equibiaxial	Human Genome Focus GeneChip probe array #900377 (Affymetrix)		n.a.	n.a.
Yamashiro et al. (2007)	ATF1	*ATF1*	hPDL fibroblasts (21/F, 24/F, 17/F, 22/M, n.g., n.g., P5-9, 2×10^5)	dynamic	**0.1Hz** (6cyc/min) for 0.5h, 1h, 2h, 16h	18%	Flexercell Strain Unit + flexible-bottomed culture plates (FLEX II) + vacuum (Myokai et al 2003; Banes et al 1990)	equibiaxial	decrease (qPCR, β-actin)	duration n.g.: 0.6 (ratio)*	n.g.	n.g.
Yamashiro et al. (2007)	BCL2	*BCL2*	hPDL fibroblasts (21/F, 24/F, 17/F, 22/M, n.g., n.g., P5-9, 2×10^5)	dynamic	**0.1Hz** (6cyc/min) for 0.5h, 1h, 2h, 16h	18%	Flexercell Strain Unit + flexible-bottomed culture plates (FLEX II) + vacuum (Myokai et al 2003; Banes et al 1990)	equibiaxial	increase (qPCR, β-actin)	duration n.g.: 1.3 (ratio)*	n.g.	n.g.
Yamashiro et al. (2007)	CASP3	*CASP3*	hPDL fibroblasts (21/F, 24/F, 17/F, 22/M, n.g., n.g., P5-9, 2×10^5)	dynamic	**0.1Hz** (6cyc/min) for 0.5h, 1h, 2h, 16h	18%	Flexercell Strain Unit + flexible-bottomed culture plates (FLEX II) + vacuum (Myokai et al 2003; Banes et al 1990)	equibiaxial	increase (qPCR, β-actin)	duration n.g.: 1.2 (ratio)*	n.g.	n.g.
Yamashiro et al. (2007)	FOS	*FOS*	hPDL fibroblasts (21/F, 24/F, 17/F, 22/M, n.g., n.g., P5-9, 2×10^5)	dynamic	**0.1Hz** (6cyc/min) for 0.5h, 1h, 2h, 16h	18%	Flexercell Strain Unit + flexible-bottomed culture plates (FLEX II) + vacuum (Myokai et al 2003; Banes et al 1990)	equibiaxial	increase (qPCR, β-actin)	duration n.g.: 2.0 (ratio)*	n.g.	n.g.
Yamashiro et al. (2007)	GOSR1	*GOSR1*	hPDL fibroblasts (21/F, 24/F, 17/F, 22/M, n.g., n.g., P5-9, 2×10^5)	dynamic	**0.1Hz** (6cyc/min) for 0.5h, 1h, 2h, 16h	18%	Flexercell Strain Unit + flexible-bottomed culture plates (FLEX II) + vacuum (Myokai et al 2003; Banes et al 1990)	equibiaxial	decrease (qPCR, β-actin)	duration n.g.: 0.4 (ratio)*	n.g.	n.g.
Yamashiro et al. (2007)	TP53BP2	*TP53BP2*	hPDL fibroblasts (21/F, 24/F, 17/F, 22/M, n.g., n.g., P5-9, 2×10^5)	dynamic	**0.1Hz** (6cyc/min) for 0.5h, 1h, 2h, 16h	18%	Flexercell Strain Unit + flexible-bottomed culture plates (FLEX II) + vacuum (Myokai et al 2003; Banes et al 1990)	equibiaxial	increase (qPCR, β-actin)	duration n.g.: 1.4 (ratio)*	n.g.	n.g.
Yang et al. (2006)	ALP	*ALPP*	hPDLCs (10-13/n.g., PM, exp, P4-8, confluent)	dynamic	**0.05Hz** (cycle of 3/min) for 2h, 4h, 6h, 12h, 24h	310-320 grams force	"A new model to apply intermittent mechanical stress on cells" (Zhang 1999)	uniaxial	n.g.	n.g.	increase (biochemistry test)	4h: 3 (unit/10^4 cellls)* / 4.3 (ratio-calc)
Yang et al. (2006)	OCN	*BGLAP*	hPDLCs (10-13/n.g., PM, exp, P4-8, confluent)	dynamic	**0.05Hz** (cycle of 3/min) for 2h, 4h, 6h, 12h, 24h	310-320 grams force	"A new model to apply intermittent mechanical stress on cells" (Zhang 1999)	uniaxial	n.g.	n.g.	increase (RIA)	12h: 1.6 (ng/10^4 cellls)* / 8 (ratio-calc)
Yang et al. (2006)	OPG	*TNFRSF11B*	hPDLCs (10-13/n.g., PM, exp, P4-8, confluent)	dynamic	**0.05Hz** (cycle of 3/min) for 2h, 4h, 6h, 12h, 24h	310-320 grams force	"A new model to apply intermittent mechanical stress on cells" (Zhang 1999)	uniaxial	decrease (in-situ hybridization staining)	4h: 0.3 (optical density)* / 0.5 (ratio-calc)	decrease (ELISA)	24h: 38.9 (10^{-15} mol)* / 0.9 (ratio-calc)
Yang et al. (2010)	ALP	*ALPP*	hPDLCs (10-13/n.g., PM, dig, P4-6, 80% confluent)	dynamic	**0.005Hz** (cycle of 3 minutes) for 0.5h,1h, 2h, 4h, 6h, 12h, 24h	12%	"A new model to apply intermittent mechanical stress on cells" (Yang et al 2006; further reference to Zhang 1999)	uniaxial	temporary increase (sqPCR, β-actin)	4h: 0.8 (rel)* / t_0 = 0	n.g.	n.g.
Yang et al. (2010)	CBFA1	*RUNX2*	hPDLCs (10-13/n.g., PM, dig, P4-6, 80% confluent)	dynamic	**0.005Hz** (cycle of 3 minutes) for 0.5h,1h, 2h, 4h, 6h, 12h, 24h	12%	"A new model to apply intermittent mechanical stress on cells" (Yang et al 2006; further reference to Zhang 1999)	uniaxial	increase followed by decrease (sqPCR, β-actin)	highest @ 1h: 2.0 (optical density)* / 3.3 (ratio-calc relative to t_0) lowest @ 12h: 0.3 (optical density)* / 0.5 (ratio-calc relative to t_0)	n.g.	n.g.
Yang et al. (2010)	OPG	*TNFRSF11B*	hPDLCs (10-13/n.g., PM, dig, P4-6, 80% confluent)	dynamic	**0.005Hz** (cycle of 3 minutes) for 0.5h,1h, 2h, 4h, 6h, 12h, 24h	12%	"A new model to apply intermittent mechanical stress on cells" (Yang et al 2006; further reference to Zhang 1999)	uniaxial	increase followed by decrease (sqPCR, β-actin)	highest @ 2h: 1.5 (rel)* / 1.4 (ratio-calc relative to t_0) lowest @ 6h: 0.3 (rel)* / 0.3 (ratio-calc relative to t_0)	n.g.	n.g.
Yang et al. (2010)	OPN	*SPP1*	hPDLCs (10-13/n.g., PM, dig, P4-6, 80% confluent)	dynamic	**0.005Hz** (cycle of 3 minutes) for 0.5h,1h, 2h, 4h, 6h, 12h, 24h	12%	"A new model to apply intermittent mechanical stress on cells" (Yang et al 2006; further reference to Zhang 1999)	uniaxial	increase (sqPCR, β-actin)	0.5h: 1.8 (rel)* / t_0 = 0	n.g.	n.g.

[a] Entry given as reported in the study.
[b] All official gene symbols come from the HUGO Gene Nomenclature Committee (HGNC; URL: https://www.genenames.org) after checking specificity of primers with Primer-BLAST.
[c] Gender/Sex of donors: "M" – male, "F" – female; Tooth type: "PM" – premolar, "M" – molar; Cell density: given in cells/well if not otherwise mentioned.
[d] Frequencies labeled bold orange were converted to hertz (Hz) according to its definition using the information reported in the study (in brackets)
[e] Force type deduced from the description of the force apparatus given by the authors.
[f] Gene and protein expression: 1. conclusion of change (increase, decrease…) was given according to the defined criteria in Figure 2; 2. different markers to describe the amount of change; † Information derived from figures using Engauge Digitizer; *Folds calculated by measuring the graphs, without using the Engauge Digitizer; No makers: Information derived from figures by description in the articles

Reference	Gene/ Analyte[a]	Official gene symbol / abbreviation[b]	Cell (age/gender of donors, tooth type, isolation method, passages used, cell density)[a,c]	Force type (stat./ dyn.)[a]	Force duration and frequency[d]	Force magnitude[a]	Force apparatus[a]	Force type: equibiaxial or uniaxial[e]	Gene expression: Increase, decrease, no change (method w/ reference gene); Methods: qPCR, sqPCR, Northern blot[f]	Gene expression: When it reaches peak and peak's magnitude (fold change; times or ratio; unclear = ?)[f]	Protein expression: Increase, decrease, no change (method w/ reference); Methods: ELISA, WB, RIA, EMSA, IF[f]	Protein expression: When it reaches peak and peak's magnitude (times or ratio; unclear = ?)[f]
Yang et al. (2010)	RANKL	*TNFSF11*	hPDLCs (10-13/n.g., PM, dig, P4-6, 80% confluent)	dynamic	**0.005Hz** (cycle of 3 minutes) for 0.5h,1h, 2h, 4h, 6h, 12h, 24h	12%	"A new model to apply intermittent mechanical stress on cells" (Yang et al 2006; further reference to Zhang 1999)	uniaxial	increase (sqPCR, β-actin)	24h: 0.7 (optical density)† / 14.0 (ratio-calc relative to t_0)	n.g.	n.g.
Yang et al. (2015)	IL-6	*IL6*	hPDLCs (n.g./n.g., PM, n.g., P4-6, 3×10^5)	dynamic	**0.1Hz** (6cyc/min) for 2h,4h, 8h, 24h, 48h	12%	Flexcell FX-5000™ Tension Unit + BioFlex culture plate + vacuum	equibiaxial	increase (qPCR, β-actin)	48h: 20.0 (ratio)	n.g.	n.g.
Yang et al. (2015)	MMP-1	*MMP1*	hPDLCs (n.g./n.g., PM, n.g., P4-6, 3×10^5)	dynamic	**0.1Hz** (6cyc/min) for 2h,4h, 8h, 24h, 48h	12%	Flexcell FX-5000™ Tension Unit + BioFlex culture plate + vacuum	equibiaxial	increase (qPCR, β-actin)	48h: 4.0 (ratio)*	n.g.	n.g.
Yang et al. (2015)	MMP-2	*MMP2*	hPDLCs (n.g./n.g., PM, n.g., P4-6, 3×10^5)	dynamic	**0.1Hz** (6cyc/min) for 2h,4h, 8h, 24h, 48h	12%	Flexcell FX-5000™ Tension Unit + BioFlex culture plate + vacuum	equibiaxial	increase (qPCR, β-actin)	48h: 8.0 (ratio)	n.g.	n.g.
Yang et al. (2015)	Rxfp1	*RXFP1*	hPDLCs (n.g./n.g., PM, n.g., P4-6, 3×10^5)	dynamic	**0.1Hz** (6cyc/min) for 2h, 4h, 8h, 24h	12%	Flexcell FX-5000™ Tension Unit + BioFlex culture plate + vacuum	equibiaxial	increase (sqPCR, β-actin)<br>increase (qPCR, β-actin)	sqPCR: no quantitative information is given<br>qPCR @ 24h: 8.5 (ratio)*	n.g.	n.g.
Yang et al. (2015)	Rxfp2	*RXFP2*	hPDLCs (n.g./n.g., PM, n.g., P4-6, 3×10^5)	dynamic	**0.1Hz** (6cyc/min) for 2h,4h, 8h, 24h	12%	Flexcell FX-5000™ Tension Unit + BioFlex culture plate + vacuum	equibiaxial	no change (sqPCR, β-actin)<br>qPCR is not given	no quantitative information is given	n.g.	n.g.
Yang et al. (2015)	VEGF	*VEGFA*	hPDLCs (n.g./n.g., PM, n.g., P4-6, 3×10^5)	dynamic	**0.1Hz** (6cyc/min) for 2h,4h, 8h, 24h, 48h	12%	Flexcell FX-5000™ Tension Unit + BioFlex culture plate + vacuum	equibiaxial	increase (qPCR, β-actin)	48h: 20.9 (ratio)	n.g.	n.g.
Yang et al. (2016)	ATF4	*ATF4*	hPDLCs (12-16/n.g., PM, exp, P3-4, 80% confluences)	dynamic	0.5Hz (30cyc/min) for 1h, 3h, 6h, 12h, 24h	10%	Flexcell® FX-5000™ Tension System + "six-well culture plates with 35-mm silicone membrane coated on the bottom" + vacuum	equibiaxial	increase (qPCR,GAPDH)	24h: 1.9 (FC)*	increase (WB, GAPDH)	1h: 2.2 (ratio)*
Yang et al. (2016)	Bip	*HSPA5*	hPDLCs (12-16/n.g., PM, exp, P3-4, 80% confluences)	dynamic	0.5Hz (30cyc/min) for 1h, 3h, 6h, 12h, 24h	10%	Flexcell® FX-5000™ Tension System + "six-well culture plates with 35-mm silicone membrane coated on the bottom" + vacuum	equibiaxial	increase (qPCR,GAPDH)	24h: 2.5 (FC)*	n.g.	n.g.
Yang et al. (2016)	BSP	*IBSP*	hPDLCs (12-16/n.g., PM, exp, P3-4, 80% confluences)	dynamic	0.5Hz (30cyc/min) for 1h, 3h, 6h, 12h, 24h	10%	Flexcell® FX-5000™ Tension System + "six-well culture plates with 35-mm silicone membrane coated on the bottom" + vacuum	equibiaxial	increase (qPCR,GAPDH)	24h: 2.0 (FC)*	n.g.	n.g.
Yang et al. (2016)	eIF2α / p-eIF2α	*EIF2AK3*	hPDLCs (12-16/n.g., PM, exp, P3-4, 80% confluences)	dynamic	0.5Hz (30cyc/min) for 1h, 3h, 6h, 12h, 24h	10%	Flexcell® FX-5000™ Tension System + "six-well culture plates with 35-mm silicone membrane coated on the bottom" + vacuum	equibiaxial	n.g.	n.g.	eIF2α: no change (WB, GAPDH)<br>p-eIF2α: increase (WB, GAPDH)	eIF2α: no quantitative information is given<br>p-eIF2α @ 6h: 0.6 (rel)* / 15 (ratio-calc)
Yang et al. (2016)	OCN	*BGLAP*	hPDLCs (12-16/n.g., PM, exp, P3-4, 80% confluences)	dynamic	0.5Hz (30cyc/min) for 1h, 3h, 6h, 12h, 24h	10%	Flexcell® FX-5000™ Tension System + "six-well culture plates with 35-mm silicone membrane coated on the bottom" + vacuum	equibiaxial	increase (qPCR,GAPDH)	24h: 3.2 (FC)*	n.g.	n.g.
Yang et al. (2016)	PERK	*EIF2AK3*	hPDLCs (12-16/n.g., PM, exp, P3-4, 80% confluences)	dynamic	0.5Hz (30cyc/min) for 1h, 3h, 6h, 12h, 24h	10%	Flexcell® FX-5000™ Tension System + "six-well culture plates with 35-mm silicone membrane coated on the bottom" + vacuum	equibiaxial	n.g.	n.g.	increase (WB, GAPDH)	6h: 0.7 (rel)* / 2.3 (ratio-calc)
Yang et al. (2016)	Xbp1	*XBP1*	hPDLCs (12-16/n.g., PM, exp, P3-4, 80% confluences)	dynamic	0.5Hz (30cyc/min) for 1h, 3h, 6h, 12h, 24h	10%	Flexcell® FX-5000™ Tension System + "six-well culture plates with 35-mm silicone membrane coated on the bottom" + vacuum	equibiaxial	increase (qPCR,GAPDH)	24h: 3.2 (FC)*	n.g.	n.g.
Yang et al. (2018)	ALP	*ALPP*	hPDLCs (12-24/n.g., PM, dig, P3-8, 80% confluence)	dynamic	0.1Hz (5s stress and 5s rest) for 24h	10%	Flexercell FX-4000 Strain Unit + silicon membranes of wells coated with type I collagen (BioFlex) + vacuum (Chang et al 2015)	equibiaxial	increase (qPCR, GAPDH)	1.8 (FC)*	n.g.	n.g.
Yang et al. (2018)	COL1	*COL1A1*	hPDLCs (12-24/n.g., PM, dig, P3-8, 80% confluence)	dynamic	0.1Hz (5s stress and 5s rest) for 24h	10%	Flexercell FX-4000 Strain Unit + silicon membranes of wells coated with type I collagen (BioFlex) + vacuum (Chang et al 2015)	equibiaxial	increase (qPCR, GAPDH)	1.7 (FC)*	n.g.	n.g.
Yang et al. (2018)	CTGF	*CCN2*	hPDLCs (12-24/n.g., PM, dig, P3-8, 80% confluence)	dynamic	0.1Hz (5s stress and 5s rest) for 24h	10%	Flexercell FX-4000 Strain Unit + silicon membranes of wells coated with type I collagen (BioFlex) + vacuum (Chang et al 2015)	equibiaxial	increase (qPCR, GAPDH)	3.0 (FC)	n.g.	n.g.
Yang et al. (2018)	CYR61	*CCN1*	hPDLCs (12-24/n.g., PM, dig, P3-8, 80% confluence)	dynamic	0.1Hz (5s stress and 5s rest) for 24h	10%	Flexercell FX-4000 Strain Unit + silicon membranes of wells coated with type I collagen (BioFlex) + vacuum (Chang et al 2015)	equibiaxial	increase (qPCR, GAPDH)	1.5 (FC)	n.g.	n.g.

[a] Entry given as reported in the study.
[b] All official gene symbols come from the HUGO Gene Nomenclature Committee (HGNC; URL: https://www.genenames.org) after checking specificity of primers with Primer-BLAST.
[c] Gender/Sex of donors: "M" – male, "F" – female; Tooth type: "PM" – premolar, "M" – molar; Cell density: given in cells/well if not otherwise mentioned.
[d] Frequencies labeled bold orange were converted to hertz (Hz) according to its definition using the information reported in the study (in brackets)
[e] Force type deduced from the description of the force apparatus given by the authors.
[f] Gene and protein expression: 1. conclusion of change (increase, decrease…) was given according to the defined criteria in Figure 2; 2. different markers to describe the amount of change; † Information derived from figures using Engauge Digitizer; *Folds calculated by measuring the graphs, without using the Engauge Digitizer; No makers: Information derived from figures by description in the articles

Reference	Gene/ Analyte[a]	Official gene symbol / abbreviation[b]	Cell (age/gender of donors, tooth type, isolation method, passages used, cell density)[a,c]	Force type (stat./ dyn.)[a]	Force duration and frequency[d]	Force magnitude[a]	Force apparatus[a]	Force type: equibiaxial or uniaxial[e]	Gene expression: Increase, decrease, no change (method w/ reference gene); Methods: qPCR, sqPCR, Northern blot[f]	Gene expression: When it reaches peak and peak's magnitude (fold change; times or ratio; unclear = ?)[f]	Protein expression: Increase, decrease, no change (method w/ reference); Methods: ELISA, WB, RIA, EMSA, IF[f]	Protein expression: When it reaches peak and peak's magnitude (times or ratio; unclear = ?)[f]
Yang et al. (2018)	OCN	*BGLAP*	hPDLCs (12-24/n.g., PM, dig, P3-8, 80% confluence)	dynamic	0.1Hz (5s stress and 5s rest) qPCR for 24 h; WB for 72h	10%	Flexercell FX-4000 Strain Unit + silicon membranes of wells coated with type I collagen (BioFlex) + vacuum (Chang et al 2015)	equibiaxial	increase (qPCR, GAPDH)	2.0 (FC)*	increase (WB, GAPDH)	1.8 (ratio)*
Yang et al. (2018)	OPN	*SPP1*	hPDLCs (12-24/n.g., PM, dig, P3-8, 80% confluence)	dynamic	0.1Hz (5s stress and 5s rest) qPCR for 24 h WB for 72h	10%	Flexercell FX-4000 Strain Unit + silicon membranes of wells coated with type I collagen (BioFlex) + vacuum (Chang et al 2015)	equibiaxial	increase (qPCR, GAPDH)	3.3 (FC)*	increase (WB, GAPDH)	2.4 (ratio)*
Yang et al. (2018)	OSX	*SP7*	hPDLCs (12-24/n.g., PM, dig, P3-8, 80% confluence)	dynamic	0.1Hz (5s stress and 5s rest) for 24h	10%	Flexercell FX-4000 Strain Unit + silicon membranes of wells coated with type I collagen (BioFlex) + vacuum (Chang et al 2015)	equibiaxial	increase (qPCR, GAPDH)	1.7 (FC)*	n.g.	n.g.
Yang et al. (2018)	RUNX2	*RUNX2*	hPDLCs (12-24/n.g., PM, dig, P3-8, 80% confluence)	dynamic	0.1Hz (5s stress and 5s rest) for 24h	10%	Flexercell FX-4000 Strain Unit + silicon membranes of wells coated with type I collagen (BioFlex) + vacuum (Chang et al 2015)	equibiaxial	increase (qPCR, GAPDH)	1.8 (FC)*	n.g.	n.g.
Yang et al. (2018)	YAP	*YAP1*	hPDLCs (12-24/n.g., PM, dig, P3-8, 80% confluence)	dynamic	0.1Hz (5s stress and 5s rest) for 72h	10%	Flexercell FX-4000 Strain Unit + silicon membranes of wells coated with type I collagen (BioFlex) + vacuum (Chang et al 2015)	equibiaxial	n.g.	n.g.	nucleus YAP: increase (WB, GAPDH)<br>cytoplasm YAP: decrease (WB, GAPDH)	nucleus YAP: 11.9 (ratio)*<br>cytoplasm YAP: no quantitative information is given
Yoshino et al. (2003)	PEDF	*SERPINF1*	hPDL fibroblasts (n.g./n.g., M, exp, P4-6, confluency)	dynamic	**0.2Hz** (12cyc/min: stretch for 2.5s followed by 2.5s of relaxation) sqPCR duration n.g.; WB for 24h	14%	Flexercell Strain Unit + type I collagen (35-mm 6-well) + vacuum	equibiaxial	no change (sqPCR, GAPDH)	no quantitative information is given	decrease (WB, GAPDH)	0.4 (rel)* / 0.7 (ratio-calc)
Yoshino et al. (2003)	VEGF	*VEGFA*	hPDL fibroblasts (n.g./n.g., M, exp, P4-6, confluency)	dynamic	**0.2Hz** (12cyc/min: stretch for 2.5s followed by 2.5s of relaxation) for 24h	7%, 14%, 21%	Flexercell Strain Unit + type I collagen (35-mm 6-well) + vacuum	equibiaxial	n.g.	n.g.	increase (ELISA)	14%: 1.4 (ng/2×10^5 cellls)* / 2.3 (ratio-calc)
Yoshino et al. (2003)	VEGF	*VEGFA*	hPDL fibroblasts (n.g./n.g., M, exp, P4-6, confluency)	dynamic	**0.2Hz** (12cyc/min: stretch for 2.5s followed by 2.5s of relaxation) sqPCR duration n.g.; ELISA for 12h, 24h, 36h, 48h	14%	Flexercell Strain Unit + type I collagen (35-mm 6-well) + vacuum	equibiaxial	increase (sqPCR, GAPDH)	no quantitative information is given	increase (ELISA)	48h: 3.1 (ng/2×10^5 cellls)* / 2.8 (ratio-calc)
Yu et al. (2018)	COL1	*COL1A1*	hPDLCs (18-30/n.g., M, dig, P3-5, 3×10^5)	dynamic	0.5Hz (30cyc/min) for 24h, 48h, 72h	12%	Flexcell 5000 Tension System + 6-well, flexible-bottomed culture plates (Flexcell) which were coated with type I collagen + vacuum	equibiaxial	increase (qPCR, ACTB)	48h: 1.3 (FC)*	increase (WB, β-actin)	no quantitative information is given
Yu et al. (2018)	Cyclin D1	*CCND1*	hPDLCs (18-30/n.g., M, dig, P3-5, 3×10^5)	dynamic	0.5Hz (30cyc/min) for 24h, 48h, 72h	12%	Flexcell 5000 Tension System + 6-well, flexible-bottomed culture plates (Flexcell) which were coated with type I collagen + vacuum	equibiaxial	n.g.	n.g.	decrease (WB, β-actin)	no quantitative information is given
Yu et al. (2018)	LEF1	*LEF1*	hPDLCs (18-30/n.g., M, dig, P3-5, 3×10^5)	dynamic	0.5Hz (30cyc/min) for 24h, 48h, 72h	12%	Flexcell 5000 Tension System + 6-well, flexible-bottomed culture plates (Flexcell) which were coated with type I collagen + vacuum	equibiaxial	n.g.	n.g.	decrease (WB, β-actin)	no quantitative information is given
Yu et al. (2018)	RUNX2	*RUNX2*	hPDLCs (18-30/n.g., M, dig, P3-5, 3×10^5)	dynamic	0.5Hz (30cyc/min) for 24h, 48h, 72h	12%	Flexcell 5000 Tension System + 6-well, flexible-bottomed culture plates (Flexcell) which were coated with type I collagen + vacuum	equibiaxial	increase (qPCR, ACTB)	48h: 3.4 (FC)*	increase (WB, β-actin)	no quantitative information is given
Yu et al. (2018)	SP7	*SP7*	hPDLCs (18-30/n.g., M, dig, P3-5, 3×10^5)	dynamic	0.5Hz (30cyc/min) for 24h, 48h, 72h	12%	Flexcell 5000 Tension System + 6-well, flexible-bottomed culture plates (Flexcell) which were coated with type I collagen + vacuum	equibiaxial	increase (qPCR, ACTB)	72h: 3.3 (FC)*	increase (WB, β-actin)	no quantitative information is given
Yu et al. (2018)	β-Catenin / β-Catenin, active	*CTNNB1*	hPDLCs (18-30/n.g., M, dig, P3-5, 3×10^5)	dynamic	0.5Hz (30cyc/min) for 24h, 48h, 72h	12%	Flexcell 5000 Tension System + 6-well, flexible-bottomed culture plates (Flexcell) which were coated with type I collagen + vacuum	equibiaxial	n.g.	n.g.	β-catenin: no change (WB, β-actin)<br>active β-catenin: decrease (WB, β-actin)	β-catenin: no quantitative information is given<br>active β-catenin: no quantitative information is given

[a] Entry given as reported in the study.
[b] All official gene symbols come from the HUGO Gene Nomenclature Committee (HGNC; URL: https://www.genenames.org) after checking specificity of primers with Primer-BLAST.
[c] Gender/Sex of donors: "M" – male, "F" – female; Tooth type: "PM" – premolar, "M" – molar; Cell density: given in cells/well if not otherwise mentioned.
[d] Frequencies labeled bold orange were converted to hertz (Hz) according to its definition using the information reported in the study (in brackets)
[e] Force type deduced from the description of the force apparatus given by the authors.
[f] Gene and protein expression: 1. conclusion of change (increase, decrease…) was given according to the defined criteria in Figure 2; 2. different markers to describe the amount of change; † Information derived from figures using Engauge Digitizer; *Folds calculated by measuring the graphs, without using the Engauge Digitizer; No makers: Information derived from figures by description in the articles

Reference	Gene/ Analyte[a]	Official gene symbol / abbreviation[b]	Cell (age/gender of donors, tooth type, isolation method, passages used, cell density)[a,c]	Force type (stat./ dyn.)[a]	Force duration and frequency[d]	Force magnitude[a]	Force apparatus[a]	Force type: equibiaxial or uniaxial[e]	Gene expression: Increase, decrease, no change (method w/ reference gene); Methods: qPCR, sqPCR, Northern blot[f]	Gene expression: When it reaches peak and peak's magnitude (fold change; times or ratio; unclear = ?)[f]	Protein expression: Increase, decrease, no change (method w/ reference); Methods: ELISA, WB, RIA, EMSA, IF[f]	Protein expression: When it reaches peak and peak's magnitude (times or ratio; unclear = ?)[f]
Yuda et al. (2015)	CTGF/CCN2	*CCN2*	hPDLCs (23/M. 25/F, 21/F, PM, 3 donors, n.g., P n.g., subconfluence)	dynamic	**1Hz** (60cyc/min: 0.5s stretch and 0.5s relaxation per cycle) qPCR for 1h; ELISA for 3h	8%	STB-140 (STREX, Osaka, Japan) + flexiblebottomed culture chambers coated with type I collagen (Cellmatrix I-P, Nitta Gelatin Inc, Osaka, Japan) + motor	uniaxial	donor 1: increase (qPCR, GAPDH)<br>donor 2: increase (qPCR, GAPDH)<br>donor 3: increase (qPCR, GAPDH)	donor 1: 2 (FC)*<br>donor 2: 2.4 (FC)*<br>donor 3: 1.3 (FC)*	donor 1: Increase (ELISA)<br>donor 2: Increase (ELISA)<br>donor 3: Increase (ELISA)	donor 1: 91.2 (pg/mg)* / 1.9 (ratio-calc)<br>donor 2: 82.4 (pg/mg)* / 1.6 (ratio-calc)<br>donor 3: 57.4 (pg/mg)* / 1.6 (ratio-calc)
Zhao et al. (2016)	ASC	*PYCARD*	hPDLCs (11-13/n.g., PM, exp, P4-6, confluence)	dynamic	**0.1Hz** (6cyc/min: 5s stretch and 5s relaxation) for 6h, 24h	20%	Flexcell Tension Plus system FX-5000T + six-well Bioflex plates + vacuum	equibiaxial	decrease (qPCR, GAPDH)	6h: 1.2 (ratio)*	increase followed by decrease (WB, GAPDH)	highest @ 6h: 0.8 (rel)* / 1.3 (ratio-calc)<br>lowest @ 24h: 0.3 (rel)* / 0.5 (ratio-calc)
Zhao et al. (2016)	Caspase 1	*CASP1*	hPDLCs (11-13/n.g., PM, exp, P4-6, confluence)	dynamic	**0.1Hz** (6 cyc/min: 5s stretch and 5s relaxation) for 6h, 24h	20%	Flexcell Tension Plus system FX-5000T + six-well Bioflex plates + vacuum	equibiaxial	increase followed by decrease (qPCR, GAPDH)	highest @ 6h: 1.2 (ratio)*<br>lowest @ 24h: 0.6 (ratio)*	Pro-caspase-1 (50 kDa): temporary increase (WB, GAPDH)<br>Caspase-1 (20 kDa): increase (WB, GAPDH)<br>Caspase-1-activity: increase (caspase colorimetric assay kit)	Pro-caspase-1 @ 6h: 5.2 (ratio)*<br>Caspase-1@ 6h: 6.7 (ratio)*<br>Caspase-1 activity @ 6h: 1.5 (ratio)*
Zhao et al. (2016)	Caspase 5	*CASP5*	hPDLCs (11-13/n.g., PM, exp, P4-6, confluence)	dynamic	**0.1Hz** (6 cyc/min: 5s stretch and 5s relaxation) for 6h, 24h	20%	Flexcell Tension Plus system FX-5000T + six-well Bioflex plates + vacuum	equibiaxial	increase (qPCR, GAPDH)	6h: 10 (ratio)*	Pro-caspase-5 (48 kDa): increase (WB, GAPDH)<br>Caspase-5 (20 kDa): increase (WB, GAPDH)<br>Caspase-5-activity: increase (caspase colorimetric assay kit)	Pro-caspase-5 @ 6h: 2.8 (ratio)*<br>Caspase-5 @ 6h: 2.5 (ratio)*<br>Caspase-5-activity @ 6h: 1.6 (ratio)*
Zhao et al. (2016)	IL-1β	*IL1B*	hPDLCs (11-13/n.g., PM, exp, P4-6, confluence)	dynamic	**0.1Hz** (6 cyc/min: 5s stretch and 5s relaxation) qPCR and WB for 0h, 6h, 24h; ELISA for 1h, 2h, 4h, 6h, 12h, 24h	20%	Flexcell Tension Plus system FX-5000T + six-well Bioflex plates + vacuum	equibiaxial	increase followed by decrease (qPCR, GAPDH)	highest @ 6h: 1.4 (ratio)*<br>lowest @ 24h: 0.6 (ratio)*	Pro-IL-1β (31 kDa): increase (WB, GAPDH)<br>IL-1β (17 kDa): increase (WB, GAPDH)<br>IL-1β in the culture medium: temporary increase (ELISA)	Pro-IL-1β @ 6h: 1.7 (ratio)*<br>IL-1β @6h: 1.8 (ratio)*<br>IL-1β in the culture medium @ 6h: 5.5 (ratio)*
Zhao et al. (2016)	NLRP1	*NLRP1*	hPDLCs (11-13/n.g., PM, exp, P4-6, confluence)	dynamic	**0.1Hz** (6cyc/min: 5s stretch and 5s relaxation) for 6h, 24h	20%	Flexcell Tension Plus system FX-5000T + six-well Bioflex plates + vacuum	equibiaxial	decrease (qPCR, GAPDH)	24h: 0.4 (ratio)*	increase (WB, GAPDH)	6h: 2.1 (ratio)*
Zhao et al. (2016)	NLRP3	*NLRP3*	hPDLCs (11-13/n.g., PM, exp, P4-6, confluence)	dynamic	**0.1Hz** (6cyc/min: 5s stretch and 5s relaxation) for 6h, 24h	20%	Flexcell Tension Plus system FX-5000T + six-well Bioflex plates + vacuum	equibiaxial	increase followed by decrease (qPCR, GAPDH)	highest @ 6h: 5.3 (ratio)*<br>lowest @ 24h: 0.5 (ratio)*	increase (WB, GAPDH)	24h: 8.3 (ratio)*
Zhao et al. (2017)	Caspase-5, Pro- / Caspase-5	*CASP5*	hPDLCs (11-13/n.g., PM, exp, P4-6, confluence)	dynamic	**0.1Hz** (6 cyc/min: 5s stretch followed by 5s relaxation) for 6h, 24h	10%, 20%	Cell Strain Unit (CSU) + elastic silicon rubber membrane + spherical cap (step motor) (Hao et al 2009)	equibiaxial	Caspase 5 @ 10%: increase (qPCR, β-actin)<br>Caspase 5 @ 20%: increase (qPCR, β-actin)	Caspase 5 @ 10% + 24h: 11.2 (FC)*<br>Caspase 5 @ 20% + 24h: 15.2 (FC)*	Caspase 5: 10%: increase (WB, GAPDH)<br>Caspase 5: 20%: increase (WB, GAPDH)<br>Pro-caspase 5: 10%: increase (WB, GAPDH)<br>Pro-caspase 5: 20%: increase (WB, GAPDH)	Caspase 5 @ 10% + 24h: 0.4 (rel)† / 36.2 (ratio-calc)<br>Caspase 5 @ 20% + 24h: 0.8 (rel)† / 69.1 (ratio-calc)<br>Pro-caspase 5 @ 10% + 24h: 0.5 (rel)† / 7.9 (ratio-calc)<br>Pro-caspase 5 @ 20% + 24h: 0.7 (rel)† / 12.2 (ratio-calc)
Zhuang et al. (2019)	GSDMD	*GSDMD*	hPDLCs (11-16/n.g., PM, exp, P4-6, 80-90% confluence)	dynamic	**0.1Hz** (6cyc/min: 5s stretch and 5s relaxation) for 6h, 24h	20%	Flexcell Tension Plus system FX-5000T + six-well BioFlex plates + vacuum	equibiaxial	increase (qPCR, GAPDH)	24h: 6.6 (ratio)*	GSDMD (53 kDa): no change (WB, GAPDH)<br>GSDMD (31 kDa): increase (WB, GAPDH)	GSDMD (31 kDa) @ 6h: 0.7 (rel)* / 1.8 (ratio-calc)
Zhuang et al. (2019)	IL-18	*IL18*	hPDLCs (11-16/n.g., PM, exp, P4-6, 80-90% confluence)	dynamic	**0.1Hz** (6cyc/min: 5s stretch and 5s relaxation) WB for 6h, 24h; ELISA for 1h, 2h, 4h, 6h, 12h, 24h	20%	Flexcell Tension Plus system FX-5000T + six-well BioFlex plates + vacuum	equibiaxial	n.g.	n.g.	increase followed by decrease (WB, GAPDH)<br>IL-18 in culture medium: increase (ELISA)	highest @ 6h: 0.7 (rel)* / 1.4 (ratio-calc)<br>lowest @ 24h: 0.3 (rel)* / 0.6 (ratio-calc)<br>IL-18 in culture medium 6h: 6.6 (ratio)*
Zhuang et al. (2019)	IL-1β	*IL1B*	hPDLCs (11-16/n.g., PM, exp, P4-6, 80-90% confluence)	dynamic	**0.1Hz** (6cyc/min: 5s stretch and 5s relaxation) WB for 6h, 24h; ELISA for 1h, 2h, 4h, 6h, 12h, 24h	20%	Flexcell Tension Plus system FX-5000T + six-well BioFlex plates + vacuum	equibiaxial	n.g.	n.g.	Pro-IL-1β (31 kDa): increase followed by decrease (WB, GAPDH)<br>Mature-IL-1β (17 kDa): decrease (WB, GAPDH)<br>IL-1β in culture medium: increase (ELISA)	Pro-IL-1β highest @ 6h: 0.8 (rel)* / 1.1 (ratio-calc)<br>Pro-IL-1β lowest @24h: 0.6 (rel)* / 0.9 (ratio-calc)<br>Mature-IL-1β @ 24h: 0.3 (rel)* / 0.6 (ratio-calc)<br>IL-1β in culture medium 6h: 10.4 ((ratio)*
Ziegler et al. (2010)	ARRAY	ARRAY	hPDLFs (12-14/n.g., PM, exp, P8-12, near-confluence)	static	0.5h, 3h, 6h	2.5%	Lumox dish + template with convex surface + weight (Hasegawa et al 1985)	equibiaxial	RT^2-Profiler qPCR-Array (SA Biosciences) not further specified	too many!	n.a.	n.a.
Ziegler et al. (2010)	FAK / p-FAK	*PTK2*	hPDLFs (12-14/n.g., PM, exp, P8-12, near-confluence)	static	0.25h, 0.5h, 1h, 3h, 6h	2.5%	Lumox dish + template with convex surface + weight (Hasegawa et al 1985)	equibiaxial	n.g.	n.g.	FAK: increase (WB, β-actin)<br>p-FAK: increase (WB, β-actin)	FAK @ 6h: 42.8 (rel)* / 1.3 (ratio-calc)<br>p-FAK: 0.25h: 35.9 (rel)* / 1.1 (ratio-calc)
Ziegler et al. (2010)	Intergrin β3	*ITGB3*	hPDLFs (12-14/n.g., PM, exp, P8-12, near-confluence)	static	0.25h, 0.5h, 1h, 3h, 6h	2.5%	Lumox dish + template with convex surface + weight (Hasegawa et al 1985)	equibiaxial	n.g.	n.g.	increase (WB, β-actin)	6h: 44 (rel)* / 1.3 (ratio)*

[a] Entry given as reported in the study.
[b] All official gene symbols come from the HUGO Gene Nomenclature Committee (HGNC; URL: https://www.genenames.org) after checking specificity of primers with Primer-BLAST.
[c] Gender/Sex of donors: "M" – male, "F" – female; Tooth type: "PM" – premolar, "M" – molar; Cell density: given in cells/well if not otherwise mentioned.
[d] Frequencies labeled bold orange were converted to hertz (Hz) according to its definition using the information reported in the study (in brackets)
[e] Force type deduced from the description of the force apparatus given by the authors.
[f] Gene and protein expression: 1. conclusion of change (increase, decrease…) was given according to the defined criteria in Figure 2; 2. different markers to describe the amount of change; † Information derived from figures using Engauge Digitizer; *Folds calculated by measuring the graphs, without using the Engauge Digitizer; No makers: Information derived from figures by description in the articles

Reference	Gene/ Analyte[a]	Official gene symbol / abbreviation[b]	Cell (age/gender of donors, tooth type, isolation method, passages used, cell density)[a,c]	Force type (stat./ dyn.)[a]	Force duration and frequency[d]	Force magnitude[a]	Force apparatus[a]	Force type: equibiaxial or uniaxial[e]	Gene expression: Increase, decrease, no change (method w/ reference gene); Methods: qPCR, sqPCR, Northern blot[f]	Gene expression: When it reaches peak and peak's magnitude (fold change; times or ratio; unclear = ?)[f]	Protein expression: Increase, decrease, no change (method w/ reference); Methods: ELISA, WB, RIA, EMSA, IF[f]	Protein expression: When it reaches peak and peak's magnitude (times or ratio; unclear = ?)[f]
Ziegler et al. (2010)	p38-MAP-kinase / p-p38-MAP-kinase(Thr180/ Tyr182)	*MAPK14*	hPDLFs (12-14/n.g., PM, exp, P8-12, near-confluence)	static	0.25h, 0.5h, 1h, 3h, 6h	2.5%	Lumox dish + template with convex surface + weight (Hasegawa et al 1985)	equibiaxial	n.g.	n.g.	p38: decrease followed by increase (WB, β-actin)<br>p-p38: increase (WB, β-actin)	p38 lowest @ 0.5h: 29.6 (rel)* / 0.9 (ratio-calc)<br>p38 highest @ 6h: 43.9 (rel)* / 1.3 (ratio-calc)<br>p-p38 @ 6h: 36.1 (rel)* / 1.1 (ratio-calc)
Ziegler et al. (2010)	p44/42-MAP-kinase / p-p44/42-MAP-kinase(Thr202/ Tyr204)	MAPK3; MAPK1	hPDLFs (12-14/n.g., PM, exp, P8-12, near-confluence)	static	0.25h, 0.5h, 1h, 3h, 6h	2.5%	Lumox dish + template with convex surface + weight (Hasegawa et al 1985)	equibiaxial	n.g.	n.g.	p44/42: increase followed by decrease (WB, β-actin)<br>p-p44/42: decrease followed by increase (WB, β-actin)	p44/42: highest @ 0.5h: 55.6 (rel)* / 1.1 (ratio-calc)<br>p44/42: lowest @ 3h: 45 (rel)* / 0.9 (ratio-calc)<br>p-p44/42: highest @ 6h: 37.8 (rel)* / 1.1 (ratio-calc)<br>p-p44/42: lowest @ 1h: 31.7 (rel)* / 0.9 (ratio-calc)

References

Abiko, Y., Shimizu, N., Yamaguchi, M., Suzuki, H., and Takiguchi, H. (1998). Effect of aging on functional changes of periodontal tissue cells. *Ann. Periodontol.* 3(1), 350-369. doi: 10.1902/annals.1998.3.1.350.

Agarwal, S., Long, P., Seyedain, A., Piesco, N., Shree, A., and Gassner, R. (2003). A central role for the nuclear factor-κB pathway in anti-inflammatory and proinflammatory actions of mechanical strain. *FASEB J.* 17(8), 899-901. doi: 10.1096/fj.02-0901fje.

Arima, M., Hasegawa, D., Yoshida, S., Mitarai, H., Tomokiyo, A., Hamano, S., et al. (2019). R-spondin 2 promotes osteoblastic differentiation of immature human periodontal ligament cells through the Wnt/beta-catenin signaling pathway. *J. Periodontal Res.* 54(2), 143-153. doi: 10.1111/jre.12611.

Basdra, E.K., Kohl, A., and Komposch, G. (1996). Mechanical stretching of periodontal ligament fibroblasts--a study on cytoskeletal involvement. *J. Orofac. Orthop.* 57(1), 24-30. doi: 10.1007/BF02189045.

Basdra, E.K., Papavassiliou, A.G., and Huber, L.A. (1995). Rab and rho GTPases are involved in specific response of periodontal ligament fibroblasts to mechanical stretching. *Biochim. Biophys. Acta* 1268(2), 209-213. doi: 10.1016/0167-4889(95)00090-f.

Bolcato-Bellemin, A.L., Elkaim, R., Abehsera, A., Fausser, J.L., Haikel, Y., and Tenenbaum, H. (2000). Expression of mRNAs encoding for alpha and beta integrin subunits, MMPs, and TIMPs in stretched human periodontal ligament and gingival fibroblasts. *J. Dent. Res.* 79(9), 1712-1716. doi: 10.1177/00220345000790091201.

Chang, M., Lin, H., Fu, H., Wang, B., Han, G., and Fan, M. (2017). MicroRNA-195-5p regulates osteogenic differentiation of periodontal ligament cells under mechanical loading. *J. Cell. Physiol.* 232(12), 3762-3774. doi: 10.1002/jcp.25856.

Chang, M., Lin, H., Luo, M., Wang, J., and Han, G. (2015). Integrated miRNA and mRNA expression profiling of tension force-induced bone formation in periodontal ligament cells. *In Vitro Cell. Dev. Biol. Anim.* 51(8), 797-807. doi: 10.1007/s11626-015-9892-0.

Chen, Y., Mohammed, A., Oubaidin, M., Evans, C.A., Zhou, X., Luan, X., et al. (2015). Cyclic stretch and compression forces alter microRNA-29 expression of human periodontal ligament cells. *Gene* 566(1), 13-17. doi: 10.1016/j.gene.2015.03.055.

Chen, Y.J., Shie, M.Y., Hung, C.J., Wu, B.C., Liu, S.L., Huang, T.H., et al. (2014). Activation of focal adhesion kinase induces extracellular signal-regulated kinase-mediated osteogenesis in tensile force-subjected periodontal ligament fibroblasts but not in osteoblasts. *J. Bone Miner. Metab.* 32(6), 671-682. doi: 10.1007/s00774-013-0549-3.

Chiba, M., and Mitani, H. (2004). Cytoskeletal changes and the system of regulation of alkaline phosphatase activity in human periodontal ligament cells induced by mechanical stress. *Cell Biochem. Funct.* 22(4), 249-256. doi: 10.1002/cbf.1097.

Cho, J.H., Lee, S.K., Lee, J.W., and Kim, E.C. (2010). The role of heme oxygenase-1 in mechanical stress- and lipopolysaccharide-induced osteogenic differentiation in human periodontal ligament cells. *Angle Orthod.* 80(4), 552-559. doi: 10.2319/091509-520.1.

Deschner, B., Rath, B., Jager, A., Deschner, J., Denecke, B., Memmert, S., et al. (2012). Gene analysis of signal transduction factors and transcription factors in periodontal ligament cells following application of dynamic strain. *J. Orofac. Orthop.* 73(6), 486-495, 497. doi: 10.1007/s00056-012-0104-1.

Diercke, K., Kohl, A., Lux, C.J., and Erber, R. (2011). Strain-dependent up-regulation of ephrin-B2 protein in periodontal ligament fibroblasts contributes to osteogenesis during tooth movement. *J. Biol. Chem.* 286(43), 37651-37664. doi: 10.1074/jbc.M110.166900.

Doi, T., Ohno, S., Tanimoto, K., Honda, K., Tanaka, N., Ohno-Nakahara, M., et al. (2003). Mechanical stimuli enhances the expression of RGD-CAP/betaig-h3 in the periodontal ligament. *Arch. Oral Biol.* 48(8), 573-579. doi: 10.1016/s0003-9969(03)00103-1.

Fujihara, C., Yamada, S., Ozaki, N., Takeshita, N., Kawaki, H., Takano-Yamamoto, T., et al. (2010). Role of mechanical stress-induced glutamate signaling-associated molecules in cytodifferentiation of periodontal ligament cells. *J. Biol. Chem.* 285(36), 28286-28297. doi: 10.1074/jbc.M109.097303.

Goto, K.T., Kajiya, H., Nemoto, T., Tsutsumi, T., Tsuzuki, T., Sato, H., et al. (2011). Hyperocclusion stimulates osteoclastogenesis via CCL2 expression. *J. Dent. Res.* 90(6), 793-798. doi: 10.1177/0022034511400742.

Hao, Y., Xu, C., Sun, S.Y., and Zhang, F.Q. (2009). Cyclic stretching force induces apoptosis in human periodontal ligament cells via caspase-9. *Arch. Oral Biol.* 54(9), 864-870. doi: 10.1016/j.archoralbio.2009.05.012.

He, Y., Macarak, E.J., Korostoff, J.M., and Howard, P.S. (2004). Compression and tension: differential effects on matrix accumulation by periodontal ligament fibroblasts in vitro. *Connect Tissue Res.* 45(1), 28-39. doi: 10.1080/03008200490278124.

He, Y., Xu, H., Xiang, Z., Yu, H., Xu, L., Guo, Y., et al. (2019). YAP regulates periodontal ligament cell differentiation into myofibroblast interacted with RhoA/ROCK pathway. *J. Cell. Physiol.* 234(4), 5086-5096. doi: 10.1002/jcp.27312.

Howard, P.S., Kucich, U., Taliwal, R., and Korostoff, J.M. (1998). Mechanical forces alter extracellular matrix synthesis by human periodontal ligament fibroblasts. *J. Periodontal Res.* 33(8), 500-508. doi: 10.1111/j.1600-0765.1998.tb02350.x.

Huelter-Hassler, D., Tomakidi, P., Steinberg, T., and Jung, B.A. (2017). Orthodontic strain affects the Hippo-pathway effector YAP concomitant with proliferation in human periodontal ligament fibroblasts. *Eur. J. Orthod.* 39(3), 251-257. doi: 10.1093/ejo/cjx012.

Hülter-Hassler, D., Wein, M., Schulz, S.D., Proksch, S., Steinberg, T., Jung, B.A., et al. (2017). Biomechanical strain-induced modulation of proliferation coincides with an ERK1/2-independent nuclear YAP localization. *Exp. Cell Res.* 361(1), 93-100. doi: 10.1016/j.yexcr.2017.10.006.

Jacobs, C., Grimm, S., Ziebart, T., Walter, C., and Wehrbein, H. (2013). Osteogenic differentiation of periodontal fibroblasts is dependent on the strength of mechanical strain. *Arch. Oral Biol.* 58(7), 896-904. doi: 10.1016/j.archoralbio.2013.01.009.

Jacobs, C., Schramm, S., Dirks, I., Walter, C., Pabst, A., Meila, D., et al. (2018). Mechanical loading increases pro-inflammatory effects of nitrogen-containing bisphosphonate in human periodontal fibroblasts. *Clin. Oral Investig.* 22(2), 901-907. doi: 10.1007/s00784-017-2168-1.

Jacobs, C., Walter, C., Ziebart, T., Dirks, I., Schramm, S., Grimm, S., et al. (2015). Mechanical loading influences the effects of bisphosphonates on human periodontal ligament fibroblasts. *Clin. Oral Investig.* 19(3), 699-708. doi: 10.1007/s00784-014-1284-4.

Jacobs, C., Walter, C., Ziebart, T., Grimm, S., Meila, D., Krieger, E., et al. (2014). Induction of IL-6 and MMP-8 in human periodontal fibroblasts by static tensile strain. *Clin. Oral Investig.* 18(3), 901-908. doi: 10.1007/s00784-013-1032-1.

Jiang, Z., and Hua, Y. (2016). Hydrogen sulfide promotes osteogenic differentiation of human periodontal ligament cells via p38-MAPK signaling pathway under proper tension stimulation. *Arch. Oral Biol.* 72, 8-13. doi: 10.1016/j.archoralbio.2016.08.008.

Kaku, M., Yamamoto, T., Yashima, Y., Izumino, J., Kagawa, H., Ikeda, K., et al. (2019). Acetaminophen reduces apical root resorption during orthodontic tooth movement in rats. *Arch. Oral Biol.* 102, 83-92. doi: 10.1016/j.archoralbio.2019.04.002.

Kanzaki, H., Chiba, M., Sato, A., Miyagawa, A., Arai, K., Nukatsuka, S., et al. (2006). Cyclical tensile force on periodontal ligament cells inhibits osteoclastogenesis through OPG induction. *J. Dent. Res.* 85(5), 457-462. doi: 10.1177/154405910608500512.

Kanzaki, H., Wada, S., Yamaguchi, Y., Katsumata, Y., Itohiya, K., Fukaya, S., et al. (2019). Compression and tension variably alter Osteoprotegerin expression via miR-3198 in periodontal ligament cells. *BMC Mol. Cell Biol.* 20(1), 6. doi: 10.1186/s12860-019-0187-2.

Kikuiri, T., Hasegawa, T., Yoshimura, Y., Shirakawa, T., and Oguchi, H. (2000). Cyclic tension force activates nitric oxide production in cultured human periodontal ligament cells. *J. Periodontol.* 71(4), 533-539. doi: 10.1902/jop.2000.71.4.533.

Kim, H.J., Choi, Y.S., Jeong, M.J., Kim, B.O., Lim, S.H., Kim, D.K., et al. (2007). Expression of UNCL during development of periodontal tissue and response of periodontal ligament fibroblasts to mechanical stress in vivo and in vitro. *Cell Tissue Res.* 327(1), 25-31. doi: 10.1007/s00441-006-0304-3.

Kletsas, D., Basdra, E.K., and Papavassiliou, A.G. (2002). Effect of protein kinase inhibitors on the stretch-elicited c-Fos and c-Jun up-regulation in human PDL osteoblast-like cells. *J. Cell. Physiol.* 190(3), 313-321. doi: 10.1002/jcp.10052.

Konstantonis, D., Papadopoulou, A., Makou, M., Eliades, T., Basdra, E., and Kletsas, D. (2014). The role of cellular senescence on the cyclic stretching-mediated activation of MAPK and ALP expression and activity in human periodontal ligament fibroblasts. *Exp. Gerontol.* 57, 175-180. doi: 10.1016/j.exger.2014.05.010.

Kook, S.H., and Lee, J.C. (2012). Tensile force inhibits the proliferation of human periodontal ligament fibroblasts through Ras-p38 MAPK up-regulation. *J. Cell. Physiol.* 227(3), 1098-1106. doi: 10.1002/jcp.22829.

Lee, S.I., Park, K.H., Kim, S.J., Kang, Y.G., Lee, Y.M., and Kim, E.C. (2012). Mechanical stress-activated immune response genes via Sirtuin 1 expression in human periodontal ligament cells. *Clin. Exp. Immunol.* 168(1), 113-124. doi: 10.1111/j.1365-2249.2011.04549.x.

Lee, S.Y., Yoo, H.I., and Kim, S.H. (2015). CCR5-CCL Axis in PDL during Orthodontic Biophysical Force Application. *J. Dent. Res.* 94(12), 1715-1723. doi: 10.1177/0022034515603926.

Li, L., Han, M., Li, S., Wang, L., and Xu, Y. (2013). Cyclic tensile stress during physiological occlusal force enhances osteogenic differentiation of human periodontal ligament cells via ERK1/2-Elk1 MAPK pathway. *DNA Cell Biol.* 32(9), 488-497. doi: 10.1089/dna.2013.2070.

Li, L., Han, M.X., Li, S., Xu, Y., and Wang, L. (2014). Hypoxia regulates the proliferation and osteogenic differentiation of human periodontal ligament cells under cyclic tensile stress via mitogen-activated protein kinase pathways. *J. Periodontol.* 85(3), 498-508. doi: 10.1902/jop.2013.130048.

Li, S., Zhang, H., Li, S., Yang, Y., Huo, B., and Zhang, D. (2015). Connexin 43 and ERK regulate tension-induced signal transduction in human periodontal ligament fibroblasts. *J. Orthop. Res.* 33(7), 1008-1014. doi: 10.1002/jor.22830.

Liao, C., and Hua, Y. (2013). Effect of hydrogen sulphide on the expression of osteoprotegerin and receptor activator of NF-kappaB ligand in human periodontal ligament cells induced by tension-force stimulation. *Arch. Oral Biol.* 58(12), 1784-1790. doi: 10.1016/j.archoralbio.2013.08.004.

Liu, J., Li, Q., Liu, S., Gao, J., Qin, W., Song, Y., et al. (2017). Periodontal Ligament Stem Cells in the Periodontitis Microenvironment Are Sensitive to Static Mechanical Strain. *Stem Cells Int.* 2017, 1380851. doi: 10.1155/2017/1380851.

Liu, M., Dai, J., Lin, Y., Yang, L., Dong, H., Li, Y., et al. (2012). Effect of the cyclic stretch on the expression of osteogenesis genes in human periodontal ligament cells. *Gene* 491(2), 187-193. doi: 10.1016/j.gene.2011.09.031.

Long, P., Hu, J., Piesco, N., Buckley, M., and Agarwal, S. (2001). Low magnitude of tensile strain inhibits IL-1beta-dependent induction of pro-inflammatory cytokines and induces synthesis of IL-10 in human periodontal ligament cells in vitro. *J. Dent. Res.* 80(5), 1416-1420. doi: 10.1177/00220345010800050601.

Long, P., Liu, F., Piesco, N.P., Kapur, R., and Agarwal, S. (2002). Signaling by mechanical strain involves transcriptional regulation of proinflammatory genes in human periodontal ligament cells in vitro. *Bone* 30(4), 547-552. doi: 10.1016/s8756-3282(02)00673-7.

Ma, J., Zhao, D., Wu, Y., Xu, C., and Zhang, F. (2015). Cyclic stretch induced gene expression of extracellular matrix and adhesion molecules in human periodontal ligament cells. *Arch. Oral Biol.* 60(3), 447-455. doi: 10.1016/j.archoralbio.2014.11.019.

Matsuda, N., Morita, N., Matsuda, K., and Watanabe, M. (1998a). Proliferation and differentiation of human osteoblastic cells associated with differential activation of MAP kinases in response to epidermal growth factor, hypoxia, and mechanical stress in vitro. *Biochem. Biophys. Res. Commun.* 249(2), 350-354. doi: 10.1006/bbrc.1998.9151.

[a] Entry given as reported in the study.
[b] All official gene symbols come from the HUGO Gene Nomenclature Committee (HGNC; URL: https://www.genenames.org) after checking specificity of primers with Primer-BLAST.
[c] Gender/Sex of donors: "M" – male, "F" – female; Tooth type: "PM" – premolar, "M" – molar; Cell density: given in cells/well if not otherwise mentioned.
[d] Frequencies labeled bold orange were converted to hertz (Hz) according to its definition using the information reported in the study (in brackets)
[e] Force type deduced from the description of the force apparatus given by the authors.
[f] Gene and protein expression: 1. conclusion of change (increase, decrease…) was given according to the defined criteria in Figure 2; 2. different markers to describe the amount of change; † Information derived from figures using Engauge Digitizer; *Folds calculated by measuring the graphs, without using the Engauge Digitizer; No makers: Information derived from figures by description in the articles

Matsuda, N., Yokoyama, K., Takeshita, S., and Watanabe, M. (1998b). Role of epidermal growth factor and its receptor in mechanical stress-induced differentiation of human periodontal ligament cells in vitro. *Arch. Oral Biol.* 43(12), 987-997. doi: 10.1016/s0003-9969(98)00079-x.

Memmert, S., Damanaki, A., Weykopf, B., Rath-Deschner, B., Nokhbehsaim, M., Gotz, W., et al. (2019). Autophagy in periodontal ligament fibroblasts under biomechanical loading. *Cell Tissue Res.* doi: 10.1007/s00441-019-03063-1.

Memmert, S., Nogueira, A.V.B., Damanaki, A., Nokhbehsaim, M., Rath-Deschner, B., Götz, W., et al. (2020). Regulation of the autophagy-marker Sequestosome 1 in periodontal cells and tissues by biomechanical loading. *J. Orofac. Orthop.* 81(1), 10-21. doi: 10.1007/s00056-019-00197-3.

Meng, Y., Han, X., Huang, L., Bai, D., Yu, H., He, Y., et al. (2010). Orthodontic mechanical tension effects on the myofibroblast expression of alpha-smooth muscle actin. *Angle Orthod.* 80(5), 912-918. doi: 10.2319/101609-578.1.

Miura, S., Yamaguchi, M., Shimizu, N., and Abiko, Y. (2000). Mechanical stress enhances expression and production of plasminogen activator in aging human periodontal ligament cells. *Mech. Ageing Dev.* 112(3), 217-231. doi: 10.1016/s0047-6374(99)00095-0.

Molina, T., Kabsch, K., Alonso, A., Kohl, A., Komposch, G., and Tomakidi, P. (2001). Topographic changes of focal adhesion components and modulation of p125FAK activation in stretched human periodontal ligament fibroblasts. *J. Dent. Res.* 80(11), 1984-1989. doi: 10.1177/00220345010800110701.

Monnouchi, S., Maeda, H., Fujii, S., Tomokiyo, A., Kono, K., and Akamine, A. (2011). The roles of angiotensin II in stretched periodontal ligament cells. *J. Dent. Res.* 90(2), 181-185. doi: 10.1177/0022034510382118.

Monnouchi, S., Maeda, H., Yuda, A., Hamano, S., Wada, N., Tomokiyo, A., et al. (2015). Mechanical induction of interleukin-11 regulates osteoblastic/cementoblastic differentiation of human periodontal ligament stem/progenitor cells. *J. Periodontal Res.* 50(2), 231-239. doi: 10.1111/jre.12200.

Nakashima, K., Tsuruga, E., Hisanaga, Y., Ishikawa, H., and Sawa, Y. (2009). Stretching stimulates fibulin-5 expression and controls microfibril bundles in human periodontal ligament cells. *J. Periodontal Res.* 44(5), 622-627. doi: 10.1111/j.1600-0765.2008.01170.x.

Narimiya, T., Wada, S., Kanzaki, H., Ishikawa, M., Tsuge, A., Yamaguchi, Y., et al. (2017). Orthodontic tensile strain induces angiogenesis via type IV collagen degradation by matrix metalloproteinase-12. *J. Periodontal Res.* 52(5), 842-852. doi: 10.1111/jre.12453.

Nazet, U., Schröder, A., Spanier, G., Wolf, M., Proff, P., and Kirschneck, C. (2020). Simplified method for applying static isotropic tensile strain in cell culture experiments with identification of valid RT-qPCR reference genes for PDL fibroblasts. *Eur. J. Orthod.* 42(4), 359-370. doi: 10.1093/ejo/cjz052.

Nemoto, T., Kajiya, H., Tsuzuki, T., Takahashi, Y., and Okabe, K. (2010). Differential induction of collagens by mechanical stress in human periodontal ligament cells. *Arch. Oral Biol.* 55(12), 981-987. doi: 10.1016/j.archoralbio.2010.08.004.

Ngan, P., Saito, S., Saito, M., Lanese, R., Shanfeld, J., and Davidovitch, Z. (1990). The interactive effects of mechanical stress and interleukin-1 beta on prostaglandin E and cyclic AMP production in human periodontal ligament fibroblasts in vitro: comparison with cloned osteoblastic cells of mouse (MC3T3-E1). *Arch. Oral Biol.* 35(9), 717-725. doi: 10.1016/0003-9969(90)90094-Q.

Nogueira, A.V., Nokhbehsaim, M., Eick, S., Bourauel, C., Jäger, A., Jepsen, S., et al. (2014a). Regulation of visfatin by microbial and biomechanical signals in PDL cells. *Clin. Oral Investig.* 18(1), 171-178. doi: 10.1007/s00784-013-0935-1.

Nogueira, A.V., Nokhbehsaim, M., Eick, S., Bourauel, C., Jäger, A., Jepsen, S., et al. (2014b). Biomechanical loading modulates proinflammatory and bone resorptive mediators in bacterial-stimulated PDL cells. *Mediators Inflamm.* 2014, 425421. doi: 10.1155/2014/425421.

Nokhbehsaim, M., Deschner, B., Bourauel, C., Reimann, S., Winter, J., Rath, B., et al. (2011a). Interactions of enamel matrix derivative and biomechanical loading in periodontal regenerative healing. *J. Periodontol.* 82(12), 1725-1734. doi: 10.1902/jop.2011.100678.

Nokhbehsaim, M., Deschner, B., Winter, J., Bourauel, C., Jäger, A., Jepsen, S., et al. (2012). Anti-inflammatory effects of EMD in the presence of biomechanical loading and interleukin-1β in vitro. *Clin. Oral Investig.* 16(1), 275-283. doi: 10.1007/s00784-010-0505-8.

Nokhbehsaim, M., Deschner, B., Winter, J., Bourauel, C., Rath, B., Jager, A., et al. (2011b). Interactions of regenerative, inflammatory and biomechanical signals on bone morphogenetic protein-2 in periodontal ligament cells. *J. Periodontal Res.* 46(3), 374-381. doi: 10.1111/j.1600-0765.2011.01357.x.

Nokhbehsaim, M., Deschner, B., Winter, J., Reimann, S., Bourauel, C., Jepsen, S., et al. (2010). Contribution of orthodontic load to inflammation-mediated periodontal destruction. *J. Orofac. Orthop.* 71(6), 390-402. doi: 10.1007/s00056-010-1031-7.

Ohzeki, K., Yamaguchi, M., Shimizu, N., and Abiko, Y. (1999). Effect of cellular aging on the induction of cyclooxygenase-2 by mechanical stress in human periodontal ligament cells. *Mech. Ageing Dev.* 108(2), 151-163. doi: 10.1016/s0047-6374(99)00006-8.

Ozawa, Y., Shimizu, N., and Abiko, Y. (1997). Low-energy diode laser irradiation reduced plasminogen activator activity in human periodontal ligament cells. *Lasers Surg. Med.* 21(5), 456-463. doi: 10.1002/(sici)1096-9101(1997)21:5<456::aid-lsm7>3.0.co;2-p.

Padial-Molina, M., Volk, S.L., Rodriguez, J.C., Marchesan, J.T., Galindo-Moreno, P., and Rios, H.F. (2013). Tumor necrosis factor-alpha and Porphyromonas gingivalis lipopolysaccharides decrease periostin in human periodontal ligament fibroblasts. *J. Periodontol.* 84(5), 694-703. doi: 10.1902/jop.2012.120078.

Pan, J., Wang, T., Wang, L., Chen, W., and Song, M. (2014). Cyclic strain-induced cytoskeletal rearrangement of human periodontal ligament cells via the Rho signaling pathway. *PloS One* 9(3), e91580. doi: 10.1371/journal.pone.0091580.

Papadopoulou, A., Iliadi, A., Eliades, T., and Kletsas, D. (2017). Early responses of human periodontal ligament fibroblasts to cyclic and static mechanical stretching. *Eur. J. Orthod.* 39(3), 258-263. doi: 10.1093/ejo/cjw075.

Papadopoulou, A., Todaro, A., Eliades, T., and Kletsas, D. (2019). Effect of hyperglycaemic conditions on the response of human periodontal ligament fibroblasts to mechanical stretching. *Eur. J. Orthod.* doi: 10.1093/ejo/cjz051.

Pelaez, D., Acosta Torres, Z., Ng, T.K., Choy, K.W., Pang, C.P., and Cheung, H.S. (2017). Cardiomyogenesis of periodontal ligament-derived stem cells by dynamic tensile strain. *Cell Tissue Res.* 367(2), 229-241. doi: 10.1007/s00441-016-2503-x.

Peverali, F.A., Basdra, E.K., and Papavassiliou, A.G. (2001). Stretch-mediated activation of selective MAPK subtypes and potentiation of AP-1 binding in human osteoblastic cells. *Mol. Med.* 7(1), 68-78.

Pinkerton, M.N., Wescott, D.C., Gaffey, B.J., Beggs, K.T., Milne, T.J., and Meikle, M.C. (2008). Cultured human periodontal ligament cells constitutively express multiple osteotropic cytokines and growth factors, several of which are responsive to mechanical deformation. *J. Periodontal Res.* 43(3), 343-351. doi: 10.1111/j.1600-0765.2007.01040.x.

Qin, J., and Hua, Y. (2016). Effects of hydrogen sulfide on the expression of alkaline phosphatase, osteocalcin and collagen type I in human periodontal ligament cells induced by tension force stimulation. *Mol. Med. Rep.* 14(4), 3871-3877. doi: 10.3892/mmr.2016.5680.

Rath-Deschner, B., Deschner, J., Reimann, S., Jager, A., and Gotz, W. (2009). Regulatory effects of biomechanical strain on the insulin-like growth factor system in human periodontal cells. *J. Biomech.* 42(15), 2584-2589. doi: 10.1016/j.jbiomech.2009.07.013.

Ren, D., Wei, F., Hu, L., Yang, S., Wang, C., and Yuan, X. (2015). Phosphorylation of Runx2, induced by cyclic mechanical tension via ERK1/2 pathway, contributes to osteodifferentiation of human periodontal ligament fibroblasts. *J. Cell. Physiol.* 230(10), 2426-2436. doi: 10.1002/jcp.24972.

Ritter, N., Mussig, E., Steinberg, T., Kohl, A., Komposch, G., and Tomakidi, P. (2007). Elevated expression of genes assigned to NF-kappaB and apoptotic pathways in human periodontal ligament fibroblasts following mechanical stretch. *Cell Tissue Res.* 328(3), 537-548. doi: 10.1007/s00441-007-0382-x.

Saminathan, A., Vinoth, K.J., Wescott, D.C., Pinkerton, M.N., Milne, T.J., Cao, T., et al. (2012). The effect of cyclic mechanical strain on the expression of adhesion-related genes by periodontal ligament cells in two-dimensional culture. *J. Periodontal Res.* 47(2), 212-221. doi: 10.1111/j.1600-0765.2011.01423.x.

Shen, T., Qiu, L., Chang, H., Yang, Y., Jian, C., Xiong, J., et al. (2014). Cyclic tension promotes osteogenic differentiation in human periodontal ligament stem cells. *Int. J. Clin. Exp. Pathol.* 7(11), 7872-7880.

Shimizu, N., Goseki, T., Yamaguchi, M., Iwasawa, T., Takiguchi, H., and Abiko, Y. (1997). In vitro cellular aging stimulates interleukin-1 beta production in stretched human periodontal-ligament-derived cells. *J. Dent. Res.* 76(7), 1367-1375. doi: 10.1177/00220345970760070601.

Shimizu, N., Ozawa, Y., Yamaguchi, M., Goseki, T., Ohzeki, K., and Abiko, Y. (1998). Induction of COX-2 expression by mechanical tension force in human periodontal ligament cells. *J. Periodontol.* 69(6), 670-677. doi: 10.1902/jop.1998.69.6.670.

Shimizu, N., Yamaguchi, M., Goseki, T., Ozawa, Y., Saito, K., Takiguchi, H., et al. (1994). Cyclic-tension force stimulates interleukin-1 beta production by human periodontal ligament cells. *J. Periodontal Res.* 29(5), 328-333. doi: 10.1111/j.1600-0765.1994.tb01230.x.

Shimizu, N., Yamaguchi, M., Goseki, T., Shibata, Y., Takiguchi, H., Iwasawa, T., et al. (1995). Inhibition of prostaglandin E2 and interleukin 1-beta production by low-power laser irradiation in stretched human periodontal ligament cells. *J. Dent. Res.* 74(7), 1382-1388. doi: 10.1177/00220345950740071001.

Spencer, A.Y., and Lallier, T.E. (2009). Mechanical tension alters semaphorin expression in the periodontium. *J. Periodontol.* 80(10), 1665-1673. doi: 10.1902/jop.2009.090212.

Steinberg, T., Ziegler, N., Alonso, A., Kohl, A., Mussig, E., Proksch, S., et al. (2011). Strain response in fibroblasts indicates a possible role of the Ca(2+)-dependent nuclear transcription factor NM1 in RNA synthesis. *Cell Calcium* 49(4), 259-271. doi: 10.1016/j.ceca.2011.03.001.

Sun, C., Chen, L., Shi, X., Cao, Z., Hu, B., Yu, W., et al. (2016). Combined effects of proinflammatory cytokines and intermittent cyclic mechanical strain in inhibiting osteogenicity in human periodontal ligament cells. *Cell Biol. Int.* 40(9), 999-1007. doi: 10.1002/cbin.10641.

Sun, C., Liu, F., Cen, S., Chen, L., Wang, Y., Sun, H., et al. (2017). Tensile strength suppresses the osteogenesis of periodontal ligament cells in inflammatory microenvironments. *Mol. Med. Rep.* 16(1), 666-672. doi: 10.3892/mmr.2017.6644.

Suzuki, R., Nemoto, E., and Shimauchi, H. (2014). Cyclic tensile force up-regulates BMP-2 expression through MAP kinase and COX-2/PGE2 signaling pathways in human periodontal ligament cells. *Exp. Cell Res.* 323(1), 232-241. doi: 10.1016/j.yexcr.2014.02.013.

Symmank, J., Zimmermann, S., Goldschmitt, J., Schiegnitz, E., Wolf, M., Wehrbein, H., et al. (2019). Mechanically-induced GDF15 Secretion by Periodontal Ligament Fibroblasts Regulates Osteogenic Transcription. *Sci. Rep.* 9(1), 11516. doi: 10.1038/s41598-019-47639-x.

Takano, M., Yamaguchi, M., Nakajima, R., Fujita, S., Kojima, T., and Kasai, K. (2009). Effects of relaxin on collagen type I released by stretched human periodontal ligament cells. *Orthod. Craniofac. Res.* 12(4), 282-288. doi: 10.1111/j.1601-6343.2009.01463.x.

Tang, N., Zhao, Z., Zhang, L., Yu, Q., Li, J., Xu, Z., et al. (2012). Up-regulated osteogenic transcription factors during early response of human periodontal ligament stem cells to cyclic tensile strain. *Arch. Med. Sci.* 8(3), 422-430. doi: 10.5114/aoms.2012.28810.

Tantilertanant, Y., Niyompanich, J., Everts, V., Supaphol, P., Pavasant, P., and Sanchavanakit, N. (2019a). Cyclic tensile force-upregulated IL6 increases MMP3 expression by human periodontal ligament cells. *Arch. Oral Biol.* 107, 104495. doi: 10.1016/j.archoralbio.2019.104495.

Tantilertanant, Y., Niyompanich, J., Everts, V., Supaphol, P., Pavasant, P., and Sanchavanakit, N. (2019b). Cyclic tensile force stimulates BMP9 synthesis and in vitro mineralization by human periodontal ligament cells. *J. Cell. Physiol.* 234(4), 4528-4539. doi: 10.1002/jcp.27257.

Tsuji, K., Uno, K., Zhang, G.X., and Tamura, M. (2004). Periodontal ligament cells under intermittent tensile stress regulate mRNA expression of osteoprotegerin and tissue inhibitor of matrix metalloprotease-1 and -2. *J. Bone Miner. Metab.* 22(2), 94-103. doi: 10.1007/s00774-003-0456-0.

Tsuruga, E., Nakashima, K., Ishikawa, H., Yajima, T., and Sawa, Y. (2009). Stretching modulates oxytalan fibers in human periodontal ligament cells. *J. Periodontal Res.* 44(2), 170-174. doi: 10.1111/j.1600-0765.2008.01099.x.

Tsuruga, E., Oka, K., Hatakeyama, Y., Isokawa, K., and Sawa, Y. (2012). Latent transforming growth factor-beta binding protein 2 negatively regulates coalescence of oxytalan fibers induced by stretching stress. *Connect Tissue Res.* 53(6), 521-527. doi: 10.3109/03008207.2012.702816.

Wada, S., Kanzaki, H., Narimiya, T., and Nakamura, Y. (2017). Novel device for application of continuous mechanical tensile strain to mammalian cells. *Biol. Open* 6(4), 518-524. doi: 10.1242/bio.023671.

Wang, H., Feng, C., Jin, Y., Tan, W., and Wei, F. (2019a). Identification and characterization of circular RNAs involved in mechanical force-induced periodontal ligament stem cells. *J. Cell. Physiol.* 234(7), 10166-10177. doi: 10.1002/jcp.27686.

Wang, L., Pan, J., Wang, T., Song, M., and Chen, W. (2013). Pathological cyclic strain-induced apoptosis in human periodontal ligament cells through the RhoGDIalpha/caspase-3/PARP pathway. *PloS One* 8(10), e75973. doi: 10.1371/journal.pone.0075973.

Wang, Y., Hu, B., Hu, R., Tong, X., Zhang, M., Xu, C., et al. (2019b). TAZ contributes to osteogenic differentiation of periodontal ligament cells under tensile stress. *J. Periodontal Res.* doi: 10.1111/jre.12698.

Wang, Y., Li, Y., Fan, X., Zhang, Y., Wu, J., and Zhao, Z. (2011). Early proliferation alteration and differential gene expression in human periodontal ligament cells subjected to cyclic tensile stress. *Arch. Oral Biol.* 56(2), 177-186. doi: 10.1016/j.archoralbio.2010.09.009.

Wang, Y.F., Zuo, Z.H., Luo, P., Pang, F.S., and Hu, J.T. (2018). The effect of cyclic tensile force on the actin cytoskeleton organization and morphology of human periodontal ligament cells. *Biochem. Biophys. Res. Commun.* 506(4), 950-955. doi: 10.1016/j.bbrc.2018.10.163.

Wei, F., Liu, D., Feng, C., Zhang, F., Yang, S., Hu, Y., et al. (2015). microRNA-21 mediates stretch-induced osteogenic differentiation in human periodontal ligament stem cells. *Stem Cells Dev.* 24(3), 312-319. doi: 10.1089/scd.2014.0191.

Wei, F.L., Wang, J.H., Ding, G., Yang, S.Y., Li, Y., Hu, Y.J., et al. (2014). Mechanical force-induced specific MicroRNA expression in human periodontal ligament stem cells. *Cells Tissues Organs* 199(5-6), 353-363. doi: 10.1159/000369613.

Wescott, D.C., Pinkerton, M.N., Gaffey, B.J., Beggs, K.T., Milne, T.J., and Meikle, M.C. (2007). Osteogenic gene expression by human periodontal ligament cells under cyclic tension. *J. Dent. Res.* 86(12), 1212-1216. doi: 10.1177/154405910708601214.

Wolf, M., Lossdorfer, S., Kupper, K., and Jager, A. (2014). Regulation of high mobility group box protein 1 expression following mechanical loading by orthodontic forces in vitro and in vivo. *Eur. J. Orthod.* 36(6), 624-631. doi: 10.1093/ejo/cjt037.

Wu, J., Song, M., Li, T., Zhu, Z., and Pan, J. (2015). The Rho-mDia1 signaling pathway is required for cyclic strain-

[a] Entry given as reported in the study.
[b] All official gene symbols come from the HUGO Gene Nomenclature Committee (HGNC; URL: https://www.genenames.org) after checking specificity of primers with Primer-BLAST.
[c] Gender/Sex of donors: "M" – male, "F" – female; Tooth type: "PM" – premolar, "M" – molar; Cell density: given in cells/well if not otherwise mentioned.
[d] Frequencies labeled bold orange were converted to hertz (Hz) according to its definition using the information reported in the study (in brackets)
[e] Force type deduced from the description of the force apparatus given by the authors.
[f] Gene and protein expression: 1. conclusion of change (increase, decrease…) was given according to the defined criteria in Figure 2; 2. different markers to describe the amount of change; † Information derived from figures using Engauge Digitizer; *Folds calculated by measuring the graphs, without using the Engauge Digitizer; No makers: Information derived from figures by description in the articles

induced cytoskeletal rearrangement of human periodontal ligament cells. *Exp. Cell Res.* 337(1), 28-36. doi: 10.1016/j.yexcr.2015.07.016.

Wu, Y., Ou, Y., Liao, C., Liang, S., and Wang, Y. (2019a). High-throughput sequencing analysis of the expression profile of microRNAs and target genes in mechanical force-induced osteoblastic/cementoblastic differentiation of human periodontal ligament cells. *Am. J. Transl. Res.* 11(6), 3398-3411.

Wu, Y., Zhao, D., Zhuang, J., Zhang, F., and Xu, C. (2016). Caspase-8 and Caspase-9 Functioned Differently at Different Stages of the Cyclic Stretch-Induced Apoptosis in Human Periodontal Ligament Cells. *PloS One* 11(12), e0168268. doi: 10.1371/journal.pone.0168268.

Wu, Y., Zhuang, J., Zhao, D., and Xu, C. (2019b). Interaction between caspase-3 and caspase-5 in the stretch-induced programmed cell death in the human periodontal ligament cells. *J. Cell. Physiol.* 234(8), 13571-13581. doi: 10.1002/jcp.28035.

Wu, Y., Zhuang, J., Zhao, D., Zhang, F., Ma, J., and Xu, C. (2017). Cyclic stretch-induced the cytoskeleton rearrangement and gene expression of cytoskeletal regulators in human periodontal ligament cells. *Acta Odontol. Scand.* 75(7), 507-516. doi: 10.1080/00016357.2017.1347823.

Xu, C., Fan, Z., Shan, W., Hao, Y., Ma, J., Huang, Q., et al. (2012). Cyclic stretch influenced expression of membrane connexin 43 in human periodontal ligament cell. *Arch. Oral Biol.* 57(12), 1602-1608. doi: 10.1016/j.archoralbio.2012.07.002.

Xu, C., Hao, Y., Wei, B., Ma, J., Li, J., Huang, Q., et al. (2011). Apoptotic gene expression by human periodontal ligament cells following cyclic stretch. *J. Periodontal Res.* 46(6), 742-748. doi: 10.1111/j.1600-0765.2011.01397.x.

Xu, H., Bai, D., Ruest, L.B., Feng, J.Q., Guo, Y.W., Tian, Y., et al. (2015). Expression analysis of alpha-smooth muscle actin and tenascin-C in the periodontal ligament under orthodontic loading or in vitro culture. *Int. J. Oral Sci.* 7(4), 232-241. doi: 10.1038/ijos.2015.26.

Xu, H.Y., Nie, E.M., Deng, G., Lai, L.Z., Sun, F.Y., Tian, H., et al. (2017). Periostin is essential for periodontal ligament remodeling during orthodontic treatment. *Mol. Med. Rep.* 15(4), 1800-1806. doi: 10.3892/mmr.2017.6200.

Yamaguchi, M., Ozawa, Y., Nogimura, A., Aihara, N., Kojima, T., Hirayama, Y., et al. (2004). Cathepsins B and L increased during response of periodontal ligament cells to mechanical stress in vitro. *Connect Tissue Res.* 45(3), 181-189. doi: 10.1080/03008200490514149.

Yamaguchi, M., and Shimizu, N. (1994). Identification of factors mediating the decrease of alkaline phosphatase activity caused by tension-force in periodontal ligament cells. *Gen. Pharmacol.* 25(6), 1229-1235. doi: 10.1016/0306-3623(94)90142-2.

Yamaguchi, M., Shimizu, N., Goseki, T., Shibata, Y., Takiguchi, H., Iwasawa, T., et al. (1994). Effect of different magnitudes of tension force on prostaglandin E2 production by human periodontal ligament cells. *Arch. Oral Biol.* 39(10), 877-884. doi: 10.1016/0003-9969(94)90019-1.

Yamaguchi, M., Shimizu, N., Ozawa, Y., Saito, K., Miura, S., Takiguchi, H., et al. (1997). Effect of tension-force on plasminogen activator activity from human periodontal ligament cells. *J. Periodontal Res.* 32(3), 308-314. doi: 10.1111/j.1600-0765.1997.tb00539.x.

Yamaguchi, M., Shimizu, N., Shibata, Y., and Abiko, Y. (1996). Effects of different magnitudes of tension-force on alkaline phosphatase activity in periodontal ligament cells. *J. Dent. Res.* 75(3), 889-894. doi: 10.1177/00220345960750030501.

Yamaguchi, N., Chiba, M., and Mitani, H. (2002). The induction of c-fos mRNA expression by mechanical stress in human periodontal ligament cells. *Arch. Oral Biol.* 47(6), 465-471. doi: 10.1016/s0003-9969(02)00022-5.

Yamashiro, K., Myokai, F., Hiratsuka, K., Yamamoto, T., Senoo, K., Arai, H., et al. (2007). Oligonucleotide array analysis of cyclic tension-responsive genes in human periodontal ligament fibroblasts. *Int. J. Biochem. Cell Biol.* 39(5), 910-921. doi: 10.1016/j.biocel.2007.01.015.

Yang, S.Y., Kim, J.W., Lee, S.Y., Kang, J.H., Ulziisaikhan, U., Yoo, H.I., et al. (2015). Upregulation of relaxin receptors in the PDL by biophysical force. *Clin. Oral Investig.* 19(3), 657-665. doi: 10.1007/s00784-014-1276-4.

Yang, S.Y., Wei, F.L., Hu, L.H., and Wang, C.L. (2016). PERK-eIF2alpha-ATF4 pathway mediated by endoplasmic reticulum stress response is involved in osteodifferentiation of human periodontal ligament cells under cyclic mechanical force. *Cell. Signal.* 28(8), 880-886. doi: 10.1016/j.cellsig.2016.04.003.

Yang, Y., Wang, B.K., Chang, M.L., Wan, Z.Q., and Han, G.L. (2018). Cyclic Stretch Enhances Osteogenic Differentiation of Human Periodontal Ligament Cells via YAP Activation. *Biomed Res. Int.* 2018, 2174824. doi: 10.1155/2018/2174824.

Yang, Y., Yang, Y., Li, X., Cui, L., Fu, M., Rabie, A.B., et al. (2010). Functional analysis of core binding factor a1 and its relationship with related genes expressed by human periodontal ligament cells exposed to mechanical stress. *Eur. J. Orthod.* 32(6), 698-705. doi: 10.1093/ejo/cjq010.

Yang, Y.Q., Li, X.T., Rabie, A.B., Fu, M.K., and Zhang, D. (2006). Human periodontal ligament cells express osteoblastic phenotypes under intermittent force loading in vitro. *Front. Biosci.* 11, 776-781. doi: 10.2741/1835.

Yoshino, H., Morita, I., Murota, S.I., and Ishikawa, I. (2003). Mechanical stress induces production of angiogenic regulators in cultured human gingival and periodontal ligament fibroblasts. *J. Periodontal Res.* 38(4), 405-410. doi: 10.1034/j.1600-0765.2003.00660.x.

Yu, W., Hu, B., Shi, X., Cao, Z., Ren, M., He, Z., et al. (2018). Nicotine inhibits osteogenic differentiation of human periodontal ligament cells under cyclic tensile stress through canonical Wnt pathway and alpha7 nicotinic acetylcholine receptor. *J. Periodontal Res.* 53(4), 555-564. doi: 10.1111/jre.12545.

Yuda, A., Maeda, H., Fujii, S., Monnouchi, S., Yamamoto, N., Wada, N., et al. (2015). Effect of CTGF/CCN2 on osteo/cementoblastic and fibroblastic differentiation of a human periodontal ligament stem/progenitor cell line. *J. Cell. Physiol.* 230(1), 150-159. doi: 10.1002/jcp.24693.

Zhao, D., Wu, Y., Xu, C., and Zhang, F. (2017). Cyclic-stretch induces apoptosis in human periodontal ligament cells by activation of caspase-5. *Arch. Oral Biol.* 73, 129-135. doi: 10.1016/j.archoralbio.2016.10.009.

Zhao, D., Wu, Y., Zhuang, J., Xu, C., and Zhang, F. (2016). Activation of NLRP1 and NLRP3 inflammasomes contributed to cyclic stretch-induced pyroptosis and release of IL-1beta in human periodontal ligament cells. *Oncotarget* 7(42), 68292-68302. doi: 10.18632/oncotarget.11944.

Zhuang, J., Wang, Y., Qu, F., Wu, Y., Zhao, D., and Xu, C. (2019). Gasdermin-d Played a Critical Role in the Cyclic Stretch-Induced Inflammatory Reaction in Human Periodontal Ligament Cells. *Inflammation* 42(2), 548-558. doi: 10.1007/s10753-018-0912-6.

Ziegler, N., Alonso, A., Steinberg, T., Woodnutt, D., Kohl, A., Mussig, E., et al. (2010). Mechano-transduction in periodontal ligament cells identifies activated states of MAP-kinases p42/44 and p38-stress kinase as a mechanism for MMP-13 expression. *BMC Cell Biol.* 11, 10. doi: 10.1186/1471-2121-11-10.

[a] Entry given as reported in the study.
[b] All official gene symbols come from the HUGO Gene Nomenclature Committee (HGNC; URL: https://www.genenames.org) after checking specificity of primers with Primer-BLAST.
[c] Gender/Sex of donors: “M” – male, “F” – female; Tooth type: “PM” – premolar, “M” – molar; Cell density: given in cells/well if not otherwise mentioned.
[d] Frequencies labeled bold orange were converted to hertz (Hz) according to its definition using the information reported in the study (in brackets)
[e] Force type deduced from the description of the force apparatus given by the authors.
[f] Gene and protein expression: 1. conclusion of change (increase, decrease…) was given according to the defined criteria in Figure 2; 2. different markers to describe the amount of change; † Information derived from figures using Engauge Digitizer; *Folds calculated by measuring the graphs, without using the Engauge Digitizer; No makers: Information derived from figures by description in the articles
