# Supplementary material for: Effect of Tension on Human Periodontal Ligament Cells: Systematic Review and Network Analysis
Source: Front Bioeng Biotechnol. 2021 Aug 27;9:695053. doi: 10.3389/fbioe.2021.695053 (PMC8429507; doi:10.3389/fbioe.2021.695053)
Supplement: Supplementary file 4 [file DataSheet6.pdf]

Supplement 6 Selected publications reporting data on the top 10 most frequently investigated genes, proteins and metabolites

The publications are tabulated according to the direction of regulation reported (“increase of expression”, “decrease of expression”, “no effect of tension force on expression”, and “other changes in expression”).

Designations used in publications	Gene symbol	Articles reporting an increase of expression[1]		Articles reporting a decrease of expression[1]		Articles reporting no effect of tension force on expression[1]		Articles reporting other changes in expression	Total number of citations
		**Gene**	**Protein/Metabolite**	**Gene**	**Protein/Metabolite**	**Gene**	**Protein/Metabolite**		
Runx2<br>RUNX2 / p-RUNX2<br>CBFA1	*RUNX2*	Chang et al. (2017), Fujihara et al. (2010), He et al. (2019), Jiang and Hua (2016), Lee et al. (2015), Li et al. (2014), Li et al. (2015), Liu et al. (2017), Ren et al. (2015), Shen et al. (2014), Tang et al. (2012), Wada et al. (2017), Wang et al. (2019a), Wang et al. (2019b), Wei et al. (2014), Wei et al. (2015), Wu et al. (2019), Yang et al. (2018), Yu et al. (2018)	Jiang and Hua (2016), Li et al. (2014), Li et al. (2015), Shen et al. (2014), Tang et al. (2012), Wang et al. (2019b), Wei et al. (2014), Wu et al. (2019), Yu et al. (2018)	Nokhbehsaim et al. (2010), Nokhbehsaim et al. (2011)				Cho et al. (2010), Li et al. (2013), Sun et al. (2017), Sun et al. (2016), Yang et al. (2010)	**26**
ALP	*ALPP*	Chang et al. (2015), Chen et al. (2014), Fujihara et al. (2010), Jacobs et al. (2013), Jiang and Hua (2016), Konstantonis et al. (2014), Lee et al. (2015), Liu et al. (2017), Monnouchi et al. (2011), Papadopoulou et al. (2019), Shen et al. (2014), Yang et al. (2018)	Chang et al. (2017), Chen et al. (2014), Jiang and Hua (2016), Konstantonis et al. (2014), Matsuda et al. (1998), Nazet et al. (2020), Shen et al. (2014), Wei et al. (2015), Yang et al. (2006)	Yamaguchi et al. (1996)	Chiba and Mitani (2004), Yamaguchi and Shimizu (1994), Yamaguchi et al. (1996)			Nokhbehsaim et al. (2010), Qin and Hua (2016), Yamaguchi et al. (2002), Yang et al. (2010)	***24***
OCN<br>OC<br>Osteocalcin	*BGLAP*	Chang et al. (2015), Chen et al. (2014), Jiang and Hua (2016), Kim et al. (2007), Lee et al. (2015), Ren et al. (2015), Shen et al. (2014), Wang et al. (2019a), Wang et al. (2019b), Wei et al. (2014), Wei et al. (2015), Wu et al. (2019), Yang et al. (2016), Yang et al. (2018)	Chang et al. (2017), Chen et al. (2014), Jiang and Hua (2016), Shen et al. (2014), Wang et al. (2019b), Wei et al. (2014), Yang et al. (2006), Yang et al. (2018)					Jacobs et al. (2013), Qin and Hua (2016)	**18**
IL-1β<br>IL1β	*IL1B*	Lee et al. (2012), Nokhbehsaim et al. (2010), Ritter et al. (2007), Shimizu et al. (1997), Wada et al. (2017)	Abiko et al. (1998), Kaku et al. (2019), Liu et al. (2017), Shimizu et al. (1994), Shimizu et al. (1995), Shimizu et al. (1997), Sun et al. (2016), Sun et al. (2017)	Nokhbehsaim et al. (2012)				Long et al. (2001), Zhao et al. (2016), Zhuang et al. (2019)	***16***

1. Increase = “Increase” or “Increase with Plateau”; Decrease = “Decrease” or “Decrease with Plateau”…
2. The citations in red font appeared more than once. - **(each citation is counted only once)**

Designations used in publications	Gene symbol	Articles reporting an increase of expression[1]		Articles reporting a decrease of expression[1]		Articles reporting no effect of tension force on expression[1]		Articles reporting other changes in expression	Total number of citations
		Gene	Protein/Metabolite	Gene	Protein/Metabolite	Gene	Protein/Metabolite		
COX2	*PTGS2*	Abiko et al. (1998), Agarwal et al. (2003), Jacobs et al. (2014), Jacobs et al. (2018), Nazet et al. (2020), Nokhbehsaim et al. (2010) (20%) , Nokhbehsaim et al. (2012) (20%) , Ohzeki et al. (1999), Shimizu et al. (1998), Suzuki et al. (2014), Wada et al. (2017), Wang et al. (2011)	Long et al. (2002), Nogueira et al. (2014)			Nokhbehsaim et al. (2012) (3%)		Nokhbehsaim et al. (2010) (3%)	**14**
OPG (Cytokine)	*TNFRSF11B*	Jacobs et al. (2013), Jacobs et al. (2015), Kanzaki et al. (2006), Kanzaki et al. (2019), Lee et al. (2015), Li et al. (2015), Liao and Hua (2013), Liu et al. (2017), Monnouchi et al. (2011), Spencer and Lallier (2009), Tsuji et al. (2004)	Jacobs et al. (2013), Jacobs et al. (2015), Kanzaki et al. (2006), Kanzaki et al. (2019), Li et al. (2015), Liao and Hua (2013), Tsuji et al. (2004)	Nogueira et al. (2014), Yang et al. (2006)	Nogueira et al. (2014), Yang et al. (2006)			Yang et al. (2010)	**14**
RANKL (Cytokine)	*TNFSF11*	Kanzaki et al. (2006), Kanzaki et al. (2019), Lee et al. (2015), Li et al. (2015), Liao and Hua (2013), Liu et al. (2017), Yang et al. (2010)	Li et al. (2015), Kaku et al. (2019)	Nogueira et al. (2014), Spencer and Lallier (2009)	Liao and Hua (2013), Nogueira et al. (2014)	Tsuji et al. (2004)		Jacobs et al. (2013), Jacobs et al. (2015), Monnouchi et al. (2011)	**14**
COL1A1<br>COLIα1<br>Collagen type-I (COL-1)<br>Colα1 (collagen α1)<br>COL1<br>COL-I	*COL1A1*	Chen et al. (2015), He et al. (2004), Lee et al. (2015), Takano et al. (2009), Wang et al. (2019b), Yang et al. (2018), Yu et al. (2018)	He et al. (2004), Qin and Hua (2016), Takano et al. (2009), Wang et al. (2019b), Yu et al. (2018)	Nemoto et al. (2010), Sun et al. (2017)	Sun et al. (2016), Sun et al. (2017)			Jacobs et al. (2013)<br>Yamaguchi et al. (2002)	**13**
PGE_2	PGE_2		Abiko et al. (1998), Agarwal et al. (2003), Jacobs et al. (2014), Long et al. (2002), Ngan et al. (1990), Nogueira et al. (2014), Ohzeki et al. (1999), Shimizu et al. (1995), Shimizu et al. (1998), Suzuki et al. (2014), Yamaguchi et al. (1994)				Jacobs et al. (2018)		**12**
OSX<br>SP7<br>Osx	*SP7*	Li et al. (2014), Li et al. (2015) Ren et al. (2015), Tang et al. (2012), Wang et al. (2019a), Wang et al. (2019b), Yang et al. (2018), Yu et al. (2018)	Chang et al. (2017), Li et al. (2014), Li et al. (2015), Tang et al. (2012), Wang et al. (2019b), Yu et al. (2018)	Li et al. (2013)				Li et al. (2013) (protein)	**10**

1. Increase = “Increase” or “Increase with Plateau”; Decrease = “Decrease” or “Decrease with Plateau”…
2. The citations in red font appeared more than once. - **(each citation is counted only once)**

References

Abiko, Y., Shimizu, N., Yamaguchi, M., Suzuki, H., and Takiguchi, H. (1998). Effect of aging on functional changes of periodontal tissue cells. *Ann. Periodontol.* 3(1), 350-369. doi: 10.1902/annals.1998.3.1.350.

Agarwal, S., Long, P., Seyedain, A., Piesco, N., Shree, A., and Gassner, R. (2003). A central role for the nuclear factor-κB pathway in anti-inflammatory and proinflammatory actions of mechanical strain. *FASEB J.* 17(8), 899-901. doi: 10.1096/fj.02-0901fje.

Chang, M., Lin, H., Fu, H., Wang, B., Han, G., and Fan, M. (2017). MicroRNA-195-5p regulates osteogenic differentiation of periodontal ligament cells under mechanical loading. *J. Cell. Physiol.* 232(12), 3762-3774. doi: 10.1002/jcp.25856.

Chang, M., Lin, H., Luo, M., Wang, J., and Han, G. (2015). Integrated miRNA and mRNA expression profiling of tension force-induced bone formation in periodontal ligament cells. *In Vitro Cell. Dev. Biol. Anim.* 51(8), 797-807. doi: 10.1007/s11626-015-9892-0.

Chen, Y., Mohammed, A., Oubaidin, M., Evans, C.A., Zhou, X., Luan, X., et al. (2015). Cyclic stretch and compression forces alter microRNA-29 expression of human periodontal ligament cells. *Gene* 566(1), 13-17. doi: 10.1016/j.gene.2015.03.055.

Chen, Y.J., Shie, M.Y., Hung, C.J., Wu, B.C., Liu, S.L., Huang, T.H., et al. (2014). Activation of focal adhesion kinase induces extracellular signal-regulated kinase-mediated osteogenesis in tensile force-subjected periodontal ligament fibroblasts but not in osteoblasts. *J. Bone Miner. Metab.* 32(6), 671-682. doi: 10.1007/s00774-013-0549-3.

Chiba, M., and Mitani, H. (2004). Cytoskeletal changes and the system of regulation of alkaline phosphatase activity in human periodontal ligament cells induced by mechanical stress. *Cell Biochem. Funct.* 22(4), 249-256. doi: 10.1002/cbf.1097.

Cho, J.H., Lee, S.K., Lee, J.W., and Kim, E.C. (2010). The role of heme oxygenase-1 in mechanical stress- and lipopolysaccharide-induced osteogenic differentiation in human periodontal ligament cells. *Angle Orthod.* 80(4), 552-559. doi: 10.2319/091509-520.1.

Fujihara, C., Yamada, S., Ozaki, N., Takeshita, N., Kawaki, H., Takano-Yamamoto, T., et al. (2010). Role of mechanical stress-induced glutamate signaling-associated molecules in cytodifferentiation of periodontal ligament cells. *J. Biol. Chem.* 285(36), 28286-28297. doi: 10.1074/jbc.M109.097303.

He, Y., Macarak, E.J., Korostoff, J.M., and Howard, P.S. (2004). Compression and tension: differential effects on matrix accumulation by periodontal ligament fibroblasts in vitro. *Connect Tissue Res.* 45(1), 28-39. doi: 10.1080/03008200490278124.

He, Y., Xu, H., Xiang, Z., Yu, H., Xu, L., Guo, Y., et al. (2019). YAP regulates periodontal ligament cell differentiation into myofibroblast interacted with RhoA/ROCK pathway. *J. Cell. Physiol.* 234(4), 5086-5096. doi: 10.1002/jcp.27312.

Jacobs, C., Grimm, S., Ziebart, T., Walter, C., and Wehrbein, H. (2013). Osteogenic differentiation of periodontal fibroblasts is dependent on the strength of mechanical strain. *Arch. Oral Biol.* 58(7), 896-904. doi: 10.1016/j.archoralbio.2013.01.009.

Jacobs, C., Schramm, S., Dirks, I., Walter, C., Pabst, A., Meila, D., et al. (2018). Mechanical loading increases pro-inflammatory effects of nitrogen-containing bisphosphonate in human periodontal fibroblasts. *Clin. Oral Investig.* 22(2), 901-907. doi: 10.1007/s00784-017-2168-1.

Jacobs, C., Walter, C., Ziebart, T., Dirks, I., Schramm, S., Grimm, S., et al. (2015). Mechanical loading influences the effects of bisphosphonates on human periodontal ligament fibroblasts. *Clin. Oral Investig.* 19(3), 699-708. doi: 10.1007/s00784-014-1284-4.

Jacobs, C., Walter, C., Ziebart, T., Grimm, S., Meila, D., Krieger, E., et al. (2014). Induction of IL-6 and MMP-8 in human periodontal fibroblasts by static tensile strain. *Clin. Oral Investig.* 18(3), 901-908. doi: 10.1007/s00784-013-1032-1.

Jiang, Z., and Hua, Y. (2016). Hydrogen sulfide promotes osteogenic differentiation of human periodontal ligament cells via p38-MAPK signaling pathway under proper tension stimulation. *Arch. Oral Biol.* 72, 8-13. doi: 10.1016/j.archoralbio.2016.08.008.

Kaku, M., Yamamoto, T., Yashima, Y., Izumino, J., Kagawa, H., Ikeda, K., et al. (2019). Acetaminophen reduces apical root resorption during orthodontic tooth movement in rats. *Arch. Oral Biol.* 102, 83-92. doi: 10.1016/j.archoralbio.2019.04.002.

Kanzaki, H., Chiba, M., Sato, A., Miyagawa, A., Arai, K., Nukatsuka, S., et al. (2006). Cyclical tensile force on periodontal ligament cells inhibits osteoclastogenesis through OPG induction. *J. Dent. Res.* 85(5), 457-462. doi: 10.1177/154405910608500512.

Kanzaki, H., Wada, S., Yamaguchi, Y., Katsumata, Y., Itohiya, K., Fukaya, S., et al. (2019). Compression and tension variably alter Osteoprotegerin expression via miR-3198 in periodontal ligament cells. *BMC Mol. Cell Biol.* 20(1), 6. doi: 10.1186/s12860-019-0187-2.

Kim, H.J., Choi, Y.S., Jeong, M.J., Kim, B.O., Lim, S.H., Kim, D.K., et al. (2007). Expression of UNCL during development of periodontal tissue and response of periodontal ligament fibroblasts to mechanical stress in vivo and in vitro. *Cell Tissue Res.* 327(1), 25-31. doi: 10.1007/s00441-006-0304-3.

Konstantonis, D., Papadopoulou, A., Makou, M., Eliades, T., Basdra, E., and Kletsas, D. (2014). The role of cellular senescence on the cyclic stretching-mediated activation of MAPK and ALP expression and activity in human periodontal ligament fibroblasts. *Exp. Gerontol.* 57, 175-180. doi: 10.1016/j.exger.2014.05.010.

Lee, S.I., Park, K.H., Kim, S.J., Kang, Y.G., Lee, Y.M., and Kim, E.C. (2012). Mechanical stress-activated immune response genes via Sirtuin 1 expression in human periodontal ligament cells. *Clin. Exp. Immunol.* 168(1), 113-124. doi: 10.1111/j.1365-2249.2011.04549.x.

Lee, S.Y., Yoo, H.I., and Kim, S.H. (2015). CCR5-CCL Axis in PDL during Orthodontic Biophysical Force Application. *J. Dent. Res.* 94(12), 1715-1723. doi: 10.1177/0022034515603926.

Li, L., Han, M., Li, S., Wang, L., and Xu, Y. (2013). Cyclic tensile stress during physiological occlusal force enhances osteogenic differentiation of human periodontal ligament cells via ERK1/2-Elk1 MAPK pathway. *DNA Cell Biol.* 32(9), 488-497. doi: 10.1089/dna.2013.2070.

Li, L., Han, M.X., Li, S., Xu, Y., and Wang, L. (2014). Hypoxia regulates the proliferation and osteogenic differentiation of human periodontal ligament cells under cyclic tensile stress via mitogen-activated protein kinase pathways. *J. Periodontol.* 85(3), 498-508. doi: 10.1902/jop.2013.130048.

Li, S., Zhang, H., Li, S., Yang, Y., Huo, B., and Zhang, D. (2015). Connexin 43 and ERK regulate tension-induced signal transduction in human periodontal ligament fibroblasts. *J. Orthop. Res.* 33(7), 1008-1014. doi: 10.1002/jor.22830.

Liao, C., and Hua, Y. (2013). Effect of hydrogen sulphide on the expression of osteoprotegerin and receptor activator of NF-kappaB ligand in human periodontal ligament cells induced by tension-force stimulation. *Arch. Oral Biol.* 58(12), 1784-1790. doi: 10.1016/j.archoralbio.2013.08.004.

Liu, J., Li, Q., Liu, S., Gao, J., Qin, W., Song, Y., et al. (2017). Periodontal Ligament Stem Cells in the Periodontitis Microenvironment Are Sensitive to Static Mechanical Strain. *Stem Cells Int.* 2017, 1380851. doi: 10.1155/2017/1380851.

Long, P., Hu, J., Piesco, N., Buckley, M., and Agarwal, S. (2001). Low magnitude of tensile strain inhibits IL-1beta-dependent induction of pro-inflammatory cytokines and induces synthesis of IL-10 in human periodontal ligament cells in vitro. *J. Dent. Res.* 80(5), 1416-1420. doi: 10.1177/00220345010800050601.

Long, P., Liu, F., Piesco, N.P., Kapur, R., and Agarwal, S. (2002). Signaling by mechanical strain involves transcriptional regulation of proinflammatory genes in human periodontal ligament cells in vitro. *Bone* 30(4), 547-552. doi: 10.1016/s8756-3282(02)00673-7.

Matsuda, N., Yokoyama, K., Takeshita, S., and Watanabe, M. (1998). Role of epidermal growth factor and its receptor in mechanical stress-induced differentiation of human periodontal ligament cells in vitro. *Arch. Oral Biol.* 43(12), 987-997. doi: 10.1016/s0003-9969(98)00079-x.

Monnouchi, S., Maeda, H., Fujii, S., Tomokiyo, A., Kono, K., and Akamine, A. (2011). The roles of angiotensin II in stretched periodontal ligament cells. *J. Dent. Res.* 90(2), 181-185. doi: 10.1177/0022034510382118.

Nazet, U., Schröder, A., Spanier, G., Wolf, M., Proff, P., and Kirschneck, C. (2020). Simplified method for applying static isotropic tensile strain in cell culture experiments with identification of valid RT-qPCR reference genes for PDL fibroblasts. *Eur. J. Orthod.* 42(4), 359-370. doi: 10.1093/ejo/cjz052.

Nemoto, T., Kajiya, H., Tsuzuki, T., Takahashi, Y., and Okabe, K. (2010). Differential induction of collagens by mechanical stress in human periodontal ligament cells. *Arch. Oral Biol.* 55(12), 981-987. doi: 10.1016/j.archoralbio.2010.08.004.

Ngan, P., Saito, S., Saito, M., Lanese, R., Shanfeld, J., and Davidovitch, Z. (1990). The interactive effects of mechanical stress and interleukin-1 beta on prostaglandin E and cyclic AMP production in human periodontal ligament fibroblasts in vitro: comparison with cloned osteoblastic cells of mouse (MC3T3-E1). *Arch. Oral Biol.* 35(9), 717-725. doi: 10.1016/0003-9969(90)90094-Q.

Nogueira, A.V., Nokhbehsaim, M., Eick, S., Bourauel, C., Jäger, A., Jepsen, S., et al. (2014). Biomechanical loading modulates proinflammatory and bone resorptive mediators in bacterial-stimulated PDL cells. *Mediators Inflamm.* 2014, 425421. doi: 10.1155/2014/425421.

Nokhbehsaim, M., Deschner, B., Bourauel, C., Reimann, S., Winter, J., Rath, B., et al. (2011). Interactions of enamel matrix derivative and biomechanical loading in periodontal regenerative healing. *J. Periodontol.* 82(12), 1725-1734. doi: 10.1902/jop.2011.100678.

Nokhbehsaim, M., Deschner, B., Winter, J., Bourauel, C., Jäger, A., Jepsen, S., et al. (2012). Anti-inflammatory effects of EMD in the presence of biomechanical loading and interleukin-1β in vitro. *Clin. Oral Investig.* 16(1), 275-283. doi: 10.1007/s00784-010-0505-8.

Nokhbehsaim, M., Deschner, B., Winter, J., Reimann, S., Bourauel, C., Jepsen, S., et al. (2010). Contribution of orthodontic load to inflammation-mediated periodontal destruction. *J. Orofac. Orthop.* 71(6), 390-402. doi: 10.1007/s00056-010-1031-7.

Ohzeki, K., Yamaguchi, M., Shimizu, N., and Abiko, Y. (1999). Effect of cellular aging on the induction of cyclooxygenase-2 by mechanical stress in human periodontal ligament cells. *Mech. Ageing Dev.* 108(2), 151-163. doi: 10.1016/s0047-6374(99)00006-8.

Papadopoulou, A., Todaro, A., Eliades, T., and Kletsas, D. (2019). Effect of hyperglycaemic conditions on the response of human periodontal ligament fibroblasts to mechanical stretching. *Eur. J. Orthod.* doi: 10.1093/ejo/cjz051.

Qin, J., and Hua, Y. (2016). Effects of hydrogen sulfide on the expression of alkaline phosphatase, osteocalcin and collagen type I in human periodontal ligament cells induced by tension force stimulation. *Mol. Med. Rep.* 14(4), 3871-3877. doi: 10.3892/mmr.2016.5680.

Ren, D., Wei, F., Hu, L., Yang, S., Wang, C., and Yuan, X. (2015). Phosphorylation of Runx2, induced by cyclic mechanical tension via ERK1/2 pathway, contributes to osteodifferentiation of human periodontal ligament fibroblasts. *J. Cell. Physiol.* 230(10), 2426-2436. doi: 10.1002/jcp.24972.

Ritter, N., Mussig, E., Steinberg, T., Kohl, A., Komposch, G., and Tomakidi, P. (2007). Elevated expression of genes assigned to NF-kappaB and apoptotic pathways in human periodontal ligament fibroblasts following mechanical stretch. *Cell Tissue Res.* 328(3), 537-548. doi: 10.1007/s00441-007-0382-x.

Shen, T., Qiu, L., Chang, H., Yang, Y., Jian, C., Xiong, J., et al. (2014). Cyclic tension promotes osteogenic differentiation in human periodontal ligament stem cells. *Int. J. Clin. Exp. Pathol.* 7(11), 7872-7880.

Shimizu, N., Goseki, T., Yamaguchi, M., Iwasawa, T., Takiguchi, H., and Abiko, Y. (1997). In vitro cellular aging stimulates interleukin-1 beta production in stretched human periodontal-ligament-derived cells. *J. Dent. Res.* 76(7), 1367-1375. doi: 10.1177/00220345970760070601.

Shimizu, N., Ozawa, Y., Yamaguchi, M., Goseki, T., Ohzeki, K., and Abiko, Y. (1998). Induction of COX-2 expression by mechanical tension force in human periodontal ligament cells. *J. Periodontol.* 69(6), 670-677. doi: 10.1902/jop.1998.69.6.670.

Shimizu, N., Yamaguchi, M., Goseki, T., Ozawa, Y., Saito, K., Takiguchi, H., et al. (1994). Cyclic-tension force stimulates interleukin-1 beta production by human periodontal ligament cells. *J. Periodontal Res.* 29(5), 328-333. doi: 10.1111/j.1600-0765.1994.tb01230.x.

Shimizu, N., Yamaguchi, M., Goseki, T., Shibata, Y., Takiguchi, H., Iwasawa, T., et al. (1995). Inhibition of prostaglandin E2 and interleukin 1-beta production by low-power laser irradiation in stretched human periodontal ligament cells. *J. Dent. Res.* 74(7), 1382-1388. doi: 10.1177/00220345950740071001.

Spencer, A.Y., and Lallier, T.E. (2009). Mechanical tension alters semaphorin expression in the periodontium. *J. Periodontol.* 80(10), 1665-1673. doi: 10.1902/jop.2009.090212.

Sun, C., Chen, L., Shi, X., Cao, Z., Hu, B., Yu, W., et al. (2016). Combined effects of proinflammatory cytokines and intermittent cyclic mechanical strain in inhibiting osteogenicity in human periodontal ligament cells. *Cell Biol. Int.* 40(9), 999-1007. doi: 10.1002/cbin.10641.

Sun, C., Liu, F., Cen, S., Chen, L., Wang, Y., Sun, H., et al. (2017). Tensile strength suppresses the osteogenesis of periodontal ligament cells in inflammatory microenvironments. *Mol. Med. Rep.* 16(1), 666-672. doi: 10.3892/mmr.2017.6644.

Suzuki, R., Nemoto, E., and Shimauchi, H. (2014). Cyclic tensile force up-regulates BMP-2 expression through MAP kinase and COX-2/PGE2 signaling pathways in human periodontal ligament cells. *Exp. Cell Res.* 323(1), 232-241. doi: 10.1016/j.yexcr.2014.02.013.

Takano, M., Yamaguchi, M., Nakajima, R., Fujita, S., Kojima, T., and Kasai, K. (2009). Effects of relaxin on collagen type I released by stretched human periodontal ligament cells. *Orthod. Craniofac. Res.* 12(4), 282-288. doi: 10.1111/j.1601-6343.2009.01463.x.

Tang, N., Zhao, Z., Zhang, L., Yu, Q., Li, J., Xu, Z., et al. (2012). Up-regulated osteogenic transcription factors during early response of human periodontal ligament stem cells to cyclic tensile strain. *Arch. Med. Sci.* 8(3), 422-430. doi: 10.5114/aoms.2012.28810.

Tsuji, K., Uno, K., Zhang, G.X., and Tamura, M. (2004). Periodontal ligament cells under intermittent tensile stress regulate mRNA expression of osteoprotegerin and tissue inhibitor of matrix metalloprotease-1 and -2. *J. Bone Miner. Metab.* 22(2), 94-103. doi: 10.1007/s00774-003-0456-0.

Wada, S., Kanzaki, H., Narimiya, T., and Nakamura, Y. (2017). Novel device for application of continuous mechanical tensile strain to mammalian cells. *Biol. Open* 6(4), 518-524. doi: 10.1242/bio.023671.

Wang, H., Feng, C., Jin, Y., Tan, W., and Wei, F. (2019a). Identification and characterization of circular RNAs involved in mechanical force-induced periodontal ligament stem cells. *J. Cell. Physiol.* 234(7), 10166-10177. doi: 10.1002/jcp.27686.

Wang, Y., Hu, B., Hu, R., Tong, X., Zhang, M., Xu, C., et al. (2019b). TAZ contributes to osteogenic differentiation of periodontal ligament cells under tensile stress. *J. Periodontal Res.* doi: 10.1111/jre.12698.

Wang, Y., Li, Y., Fan, X., Zhang, Y., Wu, J., and Zhao, Z. (2011). Early proliferation alteration and differential gene expression in human periodontal ligament cells subjected to cyclic tensile stress. *Arch. Oral Biol.* 56(2), 177-186. doi: 10.1016/j.archoralbio.2010.09.009.

Wei, F., Liu, D., Feng, C., Zhang, F., Yang, S., Hu, Y., et al. (2015). microRNA-21 mediates stretch-induced osteogenic differentiation in human periodontal ligament stem cells. *Stem Cells Dev.* 24(3), 312-319. doi: 10.1089/scd.2014.0191.

Wei, F.L., Wang, J.H., Ding, G., Yang, S.Y., Li, Y., Hu, Y.J., et al. (2014). Mechanical force-induced specific MicroRNA expression in human periodontal ligament stem cells. *Cells Tissues Organs* 199(5-6), 353-363. doi: 10.1159/000369613.

Wu, Y., Ou, Y., Liao, C., Liang, S., and Wang, Y. (2019). High-throughput sequencing analysis of the expression profile of microRNAs and target genes in mechanical force-induced osteoblastic/cementoblastic differentiation of human periodontal ligament cells. *Am. J. Transl. Res.* 11(6), 3398-3411.

Yamaguchi, M., and Shimizu, N. (1994). Identification of factors mediating the decrease of alkaline phosphatase activity caused by tension-force in periodontal ligament cells. *Gen. Pharmacol.* 25(6), 1229-1235. doi: 10.1016/0306-3623(94)90142-2.

Yamaguchi, M., Shimizu, N., Goseki, T., Shibata, Y., Takiguchi, H., Iwasawa, T., et al. (1994). Effect of different magnitudes of tension force on prostaglandin E2 production by human periodontal ligament cells. *Arch. Oral Biol.* 39(10), 877-884. doi: 10.1016/0003-9969(94)90019-1.

Yamaguchi, M., Shimizu, N., Shibata, Y., and Abiko, Y. (1996). Effects of different magnitudes of tension-force on alkaline phosphatase activity in periodontal ligament cells. *J. Dent. Res.* 75(3), 889-894. doi: 10.1177/00220345960750030501.

Yamaguchi, N., Chiba, M., and Mitani, H. (2002). The induction of c-fos mRNA expression by mechanical stress in human periodontal ligament cells. *Arch. Oral Biol.* 47(6), 465-471. doi: 10.1016/s0003-9969(02)00022-5.

Yang, S.Y., Wei, F.L., Hu, L.H., and Wang, C.L. (2016). PERK-eIF2alpha-ATF4 pathway mediated by endoplasmic reticulum stress response is involved in osteodifferentiation of human periodontal ligament cells under cyclic mechanical force. *Cell. Signal.* 28(8), 880-886. doi: 10.1016/j.cellsig.2016.04.003.

Yang, Y., Wang, B.K., Chang, M.L., Wan, Z.Q., and Han, G.L. (2018). Cyclic Stretch Enhances Osteogenic Differentiation of Human Periodontal Ligament Cells via YAP Activation. *Biomed Res. Int.* 2018, 2174824. doi: 10.1155/2018/2174824.

Yang, Y., Yang, Y., Li, X., Cui, L., Fu, M., Rabie, A.B., et al. (2010). Functional analysis of core binding factor a1 and its relationship with related genes expressed by human periodontal ligament cells exposed to mechanical stress. *Eur. J. Orthod.* 32(6), 698-705. doi: 10.1093/ejo/cjq010.

Yang, Y.Q., Li, X.T., Rabie, A.B., Fu, M.K., and Zhang, D. (2006). Human periodontal ligament cells express osteoblastic phenotypes under intermittent force loading in vitro. *Front. Biosci.* 11, 776-781. doi: 10.2741/1835.

Yu, W., Hu, B., Shi, X., Cao, Z., Ren, M., He, Z., et al. (2018). Nicotine inhibits osteogenic differentiation of human periodontal ligament cells under cyclic tensile stress through canonical Wnt pathway and alpha7 nicotinic acetylcholine receptor. *J. Periodontal Res.* 53(4), 555-564. doi: 10.1111/jre.12545.

Zhao, D., Wu, Y., Zhuang, J., Xu, C., and Zhang, F. (2016). Activation of NLRP1 and NLRP3 inflammasomes contributed to cyclic stretch-induced pyroptosis and release of IL-1beta in human periodontal ligament cells. *Oncotarget* 7(42), 68292-68302. doi: 10.18632/oncotarget.11944.

Zhuang, J., Wang, Y., Qu, F., Wu, Y., Zhao, D., and Xu, C. (2019). Gasdermin-d Played a Critical Role in the Cyclic Stretch-Induced Inflammatory Reaction in Human Periodontal Ligament Cells. *Inflammation* 42(2), 548-558. doi: 10.1007/s10753-018-0912-6.
