# Supplementary material for: Effect of Tension on Human Periodontal Ligament Cells: Systematic Review and Network Analysis
Source: Front Bioeng Biotechnol. 2021 Aug 27;9:695053. doi: 10.3389/fbioe.2021.695053 (PMC8429507; doi:10.3389/fbioe.2021.695053)
Supplement: Supplementary file 5 [file DataSheet3.PDF]

Supplement 3: Criteria for Risk of Bias assessment

Included are the criteria (and their definitions) to assess the reporting quality of *in vitro* studies ("Reporting Risk of Bias") and the methodlogical quality of *in vitro* studies ("Methodological Risk of Bias") and the data sheets that were applied for the actual assessment.

Content

CRITERIA FOR RISK OF BIAS 2

REPORTING QUALITY OF *IN VITRO* STUDIES 2
METHODOLOGICAL QUALITY OF *IN VITRO* STUDIES 3
REFERENCES 6

DATA SHEETS FOR „RISK OF BIAS" ASSESSMENT 7

REPORTING QUALITY OF *IN VITRO* STUDIES 7
METHODOLOGICAL QUALITY OF *IN VITRO* STUDIES 8

Criteria for Risk of Bias

Reporting quality of *in vitro* studies

This table was compiled from different sources. The main structure derived from supplementary Table S4 published by Vasant et al (2018), that is based on Samuel et al. (2016).

Criterion #	Variable/Sub variable	Where should it be found?	Definition for "low risk of bias" (LoB)
1	Description of scientific background	→ Introduction	LoB: Scientific background is described.
2	Description objective	Research question – What I'm interested in? Hypotheses to be tested.<br>→ In the last paragraph of the introduction and/or the Abstract	LoB: All objectives (primary and secondary) clearly reported. ("Aim of the study was…", "We hypnotized …", etc.)
3	Justification for model	→ Abstract, Introduction and M&M	LoB: Reason for choosing cell type(s) and the type of force, its magnitude and its duration are given.<br>Optional: Reasons for choosing analytes and assays used.
4	Study design description	→ Abstract; last paragraph of the Introduction; M&M	LoB: The materials and methods are described in a way, that the experimental procedures can be replicated if the samples are given.
5	Defined experimental outcomes	→ Experimental procedures outlined in M&M and outcomes presented in results correspond	LoB: Results as presented in the Results section and the experimental procedures outlined in M&M correspond.
6	Ethical statement	→ M&M: Cell culture	LoB: Ethical statement is provided.<br>n.a.: not applicable for commercial vendors of primary cells (e.g. Lonza, Promocell, etc.) or cell lines from official sources (RIKEN, ATCC, ECACC, etc.)
7	Cell maintenance condition	→ M&M: Cell culture	LoB: Minimum required are source of cells (primary cells and cell lines), their isolation method (primary cells), culture conditions (both), passage used (primary cells) and seeding density (both), confluency level (both).
8	Description of measurement precision and variability	"*Statistics should be fully reported in the manuscript/article, including the statistical test used, exact value of N and the definitions. value of N and the definitions of center and dispersion and the precision measures (e.g., mean, median, SD, SEM, confidence intervals).*" (Biophysical Journal's Guidelines for the Reproducibility of Biophysics Research)<br>→ M&M: Statistics; Results	LoB: Measurement precision and variability are described.
9	Statistical analysis		LoB: All statistical procedures for data analysis are provided; all statistical results derive from statistical procedures described in M&M.
10	Results description	→ Results	LoB: All results outlined in "Objective" and generated by experimental procedures described M&M are given.

Abbreviations:

LoB Low risk of bias
M&M Materials and Methods

Levels:

"+" Low risk of bias (LoB)
"-" High risk of bias (HoB)
"?" Incomplete/unclear risk of bias
n. a. Not applicable

Methodological quality of *in vitro* studies

This table was compiled from different sources. The main structure derived from supplementary Table S3 published by Vansant et al. (2018) that is based on Samuel et al. (2016). Additionally, table 1 ("Glossary key terms") and further definitions listed in table 2, both from Samuel et al. (2016), were used to prepare the following table.

Criterion #	Variable/Sub variable	Definition of variable or sub variable	Definition for "low risk of bias" (LoB)
	Selection bias	Samuel et al (2016): "*Selection bias: Systematic differences in the comparison groups.*"<br><br>OHAT RoBT, p. 5: "*Selection bias refers to systematic differences between baseline characteristics of the groups that are compared (Higgins and Green 2011).*"	
1*	Selection bias / Baseline characteristics similarity/appropriate control group selection	OHAT RoBT, p. 9: "*Comparison group appropriateness refers to having similar baseline characteristics of factors related to the outcome measures of interest between groups aside from the exposures (and outcomes for case- control studies).*"	LoB: *Control and treated groups are similar at the start of the study (e.g. cell type, passage, cell density [cells/well], confluency).* => Appropriate/controlled exposure
2	Selection bias / Allocation concealment	OHAT RoBT, p. 7: "*Allocation concealment prior to assigning the exposure level or treatment group (along with randomization in question #1) helps to assure that treatment is not given selectively based on potential differences in human subjects or non-human experimental animals.*"<br><br>Samuel et al (2016): "*A process that it used to prevent selection bias. The person allocating subjects to experimental arms is unaware of which arm the subjects are being allocated **until the moment of assignment**. This prevents researchers from (unconsciously or otherwise) influencing the allocation of subjects.*"	LoB: The experimental subjects (i.e. cell culture plates) are all prepared at the same time without allocating them to specific experimental arms (no definition in advance of the experiment). Directly before an experiment, plates are randomly chosen and allocated to the experimental arms.
3	Selection bias / Randomization	OHAT RoBT, p. 5: "*Randomization of exposure or sequence generation (along with allocation concealment in question #2) helps to assure that treatment is not given selectively based on potential differences in human subjects or non-human experimental animals (e.g., randomization by animal body weight avoids potential selection bias introduced by assigning all of the smallest animals to the high-dose exposure group).*"	n. a.
4	Performance bias / Blinding of researchers	OHAT RoBT, p. 15: "*Performance bias refers to systematic differences in the care provided to human participants or experimental animals by study groups. Examples include contamination of the control group with the exposure or intervention, unbalanced provision of additional interventions or co-interventions, difference in co-interventions, inadequate blinding of providers and participants in human studies (Viswanathan et al. 2012), and inadequate blinding of research personnel to the animal's study group (Sena et al. 2007).*"<br><br>OHAT RoBT, p. 16: "*Blinding requires that research personnel do not know which administered dose or exposure level the human subject or animal is being given (i.e., study group). Human studies also require blinding of the human subjects when possible.*"	n. a.
5	Detection bias / Blinding of outcome assessors	Samuel et al (2016): "*Detection bias: Systematic differences in the outcome assessment between groups.*"<br><br>OHAT RoBT, p. 22: *"Detection bias refers to systematic differences between experimental and control groups with regards to how outcomes and exposures are assessed (Higgins and Green 2011) and also considers validity and reliability of methods used to assess outcomes and exposures (Viswanathan et al. 2012)."*<br><br>OHAT RoBT, p. 25: *"Detection bias can be minimized by using valid and reliable methods to assess the outcome applied consistently across groups (i.e., under the same method and time-frame). Objectivity of the outcome assessment and the need for blinding are two sides of the same issue. Blinding requires that outcome assessors do not know the study group or exposure level of the human subject or animal when the outcome was assessed."*	n. a.

Criterion #	Variable/Sub variable	Definition of variable or sub variable	Definition for "low risk of bias" (LoB)
6	Attrition bias<br>/ Complete outcome data	Samuel et al (2016): "*Systematic differences in excluding study units between groups*"<br><br>OHAT RoBT, p. 19: *"Attrition or exclusion bias: systematic differences in the loss or exclusion from analyses of participants or animals.* […] *Incomplete outcome data includes loss due to attrition (nonresponse, dropout, or loss of follow-up) or exclusion from analyses."*	LoB: Accounting for all included study units.<br><br>HoB: Reports on incomplete outcome data including loss due to attrition or exclusion from analyses.
7	Reporting bias<br>/ Selective outcome data	Samuel et al (2016): "*Reporting bias: Systematic omission of results in the study documentation/ publication*." And: "*Selective outcome reporting: The reporting of only selected results, not all results*."<br><br>OHAT RoBT, p. 30: *"Selective reporting bias refers to selective inclusion of outcomes in the publication of the study on the basis of the results (Hutton and Williamson 2000, Higgins and Green 2011).* […] *Selective reporting is present if pre-specified outcomes are not reported or incompletely reported.* […] *Selective reporting bias can be assessed by comparing the "methods" and "results" section of the paper, and by considering outcomes measured in the context of knowledge in the field."*	LoB: The outcome data from all experiments as given in M&M is reported and accounted for. All results given in the Results section must derive from materials and methods reported in M&M. All uninterpretable or intermediate test results and withdrawals are explained including lost samples; e.g. ELISA measurements below detection limit.
8	Confounding bias<br>/ Account for confounding variables	Samuel et al (2016): "*Systematic differences in factors potentially influencing the results between groups. […] Is very context depending. In an animal study of endocrine disruption, bedding material potentially containing phytoestrogens should be the same for all groups."*<br><br>OHAT RoBT, p. 11: "*Confounding variables or confounders include any factor that is: 1) associated with the exposure, 2) an independent risk factor for a given outcome, and 3) unequally distributed between study groups (Gerstman 2013). The potential confounder cannot be an intermediate effect on the causal pathway between exposure and the outcome (Gerstman 2013, Sterne et al. 2014). Appropriate methods to account for these differences would include multivariable analysis, stratification, matching of cases and controls, or other approaches."*<br>see also OHAT RoBT, p. 32!<br><br>OHAT RoBT, p. 13: Low risk of bias can be assumed, if *"There is direct evidence that appropriate adjustments or explicit considerations were made for primary covariates and confounders in the final analyses through the use of statistical models to reduce research-specific bias including standardization, matching, adjustment in multivariate model, stratification, propensity scoring, or other methods that were appropriately justified."*	LoB: Confounding variables were identified/named in connection with:<br>• exposure (e.g. uneven force distribution),<br>• test procedures (e.g. vehicle controls),<br>• cell culture (e.g. age and gender of donors, and passage numbers of primary cells used for experiment).<br>Appropriate adjustments or explicit considerations were made in the final analyses using statistical methods or other methods that were appropriately justified and discussed or discussed only in an appropriate section in the Discussion part.
9	Appropriate statistical methods<br>/ Sample size determination	Samuel et al (2016): "*Appropriateness of statistical methods of experimental design and data analysis has to be demonstrated/justified.*"<br><br>OHAT RoBT, p. 31: "*One of the common statistical issues identified has been reporting of statistical tests that require normally distributed data (e.g., t-test or ANOVA) without reporting that the homogeneity of variance was tested or confirmed. It is recommended that experts with some knowledge of statistical methods used in the literature participate in drafting the risk-of-bias criteria for identifying inappropriate statistical methods when a review protocol is developed. Even with early expert consultation and planning, statistical methods questions may arise when the actual studies are assessed. Additional consultation and modifications to the statistical methods risk-of-bias criteria may be necessary. When changes are made, they should be documented along with the date on which modifications were made and the logic for the changes.*"	LoB: Sample size calculation is given.
10	Appropriate statistical methods<br>/ Statistical analysis		LoB: Appropriate statistical analysis and their justification are given.<br>• Why was that specific test chosen?<br>• Preliminaries for statistical procedures are tested – e.g. normal distribution – and the tests were chosen accordingly.

Criterion #	Variable/Sub variable	Definition of variable or sub variable	Definition for "low risk of bias" (LoB)
11*	Appropriate/controlled exposure (incl. characterization)	Samuel et al (2016): "*It needs to be ensured that all subjects are treated/exposed in the same way, e.g., by controlling the food consumption per animal in a feeding study.*"<br><br>OHAT RoBT, p. 22-23: "*Detection bias refers to systematic differences between experimental and control groups with regards to how outcomes and exposures are assessed (Higgins and Green 2011) and also considers validity and reliability of methods used to assess outcomes and exposures (Viswanathan et al. 2012).* [...] *For controlled exposure studies (i.e., experimental human or animal studies), the use of reliable methods to measure exposure depends primarily on ensuring the purity and stability of the treatment compound.*"	LoB: All data on the exposure characteristics are reported for both experimental and control groups. In cell culture:<br>• same cell type,<br>• cultivated identically,<br>• same seeding density or same confluency,<br>• same passage numbers.
12*	Optimal time window used	Samuel et al (2016): "*This refers to the age and status (e.g., pregnancy or disease status) of the animals. In a developmental toxicity study, for example, the exposure should take place during the most appropriate gestation days. In cell culture experiments, the cells should be exposed at their optimal developmental state, e.g., at confluency, or within certain cell passage numbers, for which the stability of the karyotype is guaranteed.*"<br><br>OHAT RoBT, p. 2: "*Was the exposure in the appropriate biological window to affect the outcome? This is considered under indirectness. Was the outcome assessed at an adequate amount of time after the exposure for the development of the outcome? This is considered under indirectness. Does the timing of exposure or outcome assessment impact the consistency of results? If the appropriate biological window is unclear for an outcome of interest, differences in timing of exposure or outcome assessment could be used to stratify results when considering unexplained inconsistency.* [...] *Does the duration of the experiment lasts long enough to cause the biological response?*"	LoB: In cell culture experiments, the same cell type is used for experimental and control(s) condition, cultivated identically, seeded at the same densities/confluency and same passage number.<br>For force application, force type, force duration and force magnitude are proved to be of biological relevance.<br>Proved e.g. by presenting a dose-response curve or by reference to previous publications where a dose-response curve or similar was published.
13	Statement conflict of interest/funding source	Samuel et al (2016): "*Conflicts or funding by bodies with vested interests may result in (un-)conscious biases during the entire study, from planning to publication.*"	LoB: Statement conflict of interest and funding source are given.
14*	Test substance/treatment details	Samuel et al (2016): "*The test substance identity should be known, including possibly interfering impurities. Treatment details should be known, in order to assess issues such as optimal time window used.*"	LoB: All chemicals, kits or tools/apparatuses used are named with their manufacture, purity and their specificity (e.g. order/article number) and preparation for experimental application is given (e.g. dilution and diluent, etc.); PCR primers sequence, etc.
15	Test organism/system	Samuel et al (2016): "*The animal type/strain or the cell system needs to be stated, e.g. using different cell batches may introduce bias.*"	LoB: Primary cells isolated from donors of the same age range and sex. In cell culture experiments, the same cell type is used for experimental and control(s) condition, cultivated identically, seeded at the same densities/confluency and same passage number.

Abbreviations:

LoB	Low risk of bias
HoB	High risk of bias
M&M	Materials and Methods

Levels:

"+"	Low risk of bias (LoB)
"-"	High risk of bias (HoB)
"?"	Incomplete/unclear risk of bias
n. a.	Not applicable

References

Vansant et al (2018). Expression of biological mediators during orthodontic tooth movement: A systematic review. Arch Oral Biol 95:170-186.

Samuel et al (2016). Guidance on assessing the methodological and reporting quality of toxicologically relevant studies: A scoping review. Environment International 92-93: 630-646.

OHAT RoBT – OHAT Risk of Bias Rating Tool for Human and Animal Studies (January 2015). URL : https://ntp.niehs.nih.gov/ntp/ohat/pubs/riskofbiastool_508.pdf (assessed: 2019-02-11); National Toxicology Program (2015). Handbook for Conducting a Literature-Based Health Assessment Using OHAT Approach for Systematic Review and Evidence Integration. URL: https://ntp.niehs.nih.gov/ntp/ohat/pubs/handbookjan2015_508.pdf (assessed: 2019-02-11)

On mechanistic studies: National Research Council (2014). Review of EPA's Integrated Risk Information System (IRIS) process. Washington, DC: The National Academies Press. URL: https://doi.org/10.17226/18764 ; https://nap.edu/18764 (assessed: 2019-02-11)

Biophysical Journal's Guidelines for the Reproducibility of Biophysics Research (April 2017) (URL: http://www.cell.com/pb/assets/raw/journals/society/biophysj/PDFs/reproducibility-guidelines.pdf) as referenced from National Institutes of Health's Principles and Guidelines for Reporting Preclinical Research (http://www.nih.gov/about/reporting-preclinical-research.htm) (assessed: 2019-02-11)

Data sheets for „Risk of Bias“ Assessment

Reporting quality of *in vitro* studies

Reporting quality of *in vitro* studies

**Reference**
**Description of scientific background**
**Description objective**
**Justification for model**
**Study design description**
**Defined experimental outcomes**
**Ethical statement**
**Cell maintenance condition**
**Description of measurement precision and variability**
**Statistical analysis**
**Results description**

Levels:

“+”	Low risk of bias (LoB)
“-“	High risk of bias (HoB)
“?”	Incomplete/unclear risk of bias
n. a.	Not applicable

Methodological quality of *in vitro* studies

Methodological quality of *in vitro* studies

**Reference**
**Selection bias**
**Selection bias / Baseline characteristics similarity/appropriate control group selection**
**Selection bias / Allocation concealment**
**Selection bias / Randomization**
**Performance bias / Blinding of researchers**
**Detection bias / Blinding of outcome assessors**
**Attrition bias / Complete outcome data**
**Reporting bias / Selective outcome data**
**Confounding bias / Account for confounding variables**
**Appropriate statistical methods / Sample size determination**
**Appropriate statistical methods / Statistical analysis**
**Appropriate/controlled exposure (incl. characterization)**
**Optimal time window used**
**Statement conflict of interest/funding source**
**Test substance/treatment details**
**Test organism/system**

Levels:

“+” Low risk of bias (LoB)
“-“ High risk of bias (HoB)
“?” Incomplete/unclear risk of bias
n. a. Not applicable
