# Supplementary material for: Effect of Tension on Human Periodontal Ligament Cells: Systematic Review and Network Analysis
Source: Front Bioeng Biotechnol. 2021 Aug 27;9:695053. doi: 10.3389/fbioe.2021.695053 (PMC8429507; doi:10.3389/fbioe.2021.695053)
Supplement: Supplementary file 6 [file DataSheet1.PDF]

Supplement 1: Reason for Exclusion

After full text reading, articles not fulfilling the eligibility criteria were excluded. In line with the quantitative report given in the PRISMA workflow (Figure 3A) the reasoning is reported here with references.

Table S1: Reasons for exclusion after full text reading

Total number of excluded publications: n = 45

Reason for exclusion (N)	Study
i. In vivo loading (2)	Anastasi et al. (2008); Xu et al. (2017)
ii. Force application *in vitro* on extracted teeth only (1)	Atkinson and Ralph (1977)
iii. Review (4)	Pavasant and Yongchaitrakul (2011); Yamaguchi and Kasai (2005); Yang et al. (2015); Li et al. (2019a)
iv. No quantitative information on gene or protein expression (7)	Basdra (1997); Norton et al. (1995); Norton et al. (1990); Pender and McCulloch (1991); Kletsas et al. (1998); Zhong et al. (2008); Wan et al. (2019)
v. Another type of force applied (10)	Wongkhantee et al. (2008); Wolf et al. (2013), Saminathan et al. (2015); Marciniak et al. (2019); Liu et al. (2017); Kaku et al. (2016); Huang et al. (2013); Feng et al. (2017); Berendsen et al. (2009); Li et al. (2019b)
vi. 3D model (2)	Von den Hoff (2003), (Ku et al., 2009)
vii. Results not related to hPDL (1)	Rosselli-Murai et al. (2013)
viii. No information on force type (1)	Wang et al. (2016)
ix. Co-culture (1)	Xu et al. (2014)
x. Only the apparatus (1)	Andersen and Norton (1991)
xi. Other cell types or not of human origin (14)	Duarte et al. (1999); Bellows et al. (1982); Chen et al. (2013); Glogauer et al. (1995); Zhao et al. (2008); Takimoto et al. (2015); Pavlin and Gluhak-Heinrich (2001); Loesberg et al. (2005); Lew et al. (1999); Gadhari et al. (2013); Fu et al. (2016); Duncan et al. (1984); Andrade et al. (2009); Carano and Siciliani (1996)
xii. Not directly related to mechanical force (conditioned medium) (1)	Wang et al. (2019)

References

Anastasi, G., Cordasco, G., Matarese, G., Rizzo, G., Nucera, R., Mazza, M., et al. (2008). An immunohistochemical, histological, and electron-microscopic study of the human periodontal ligament during orthodontic treatment. *Int. J. Mol. Med.* 21(5), 545-554.

Andersen, K.L., and Norton, L.A. (1991). A device for the application of known simulated orthodontic forces to human cells in vitro. *J. Biomech.* 24(7), 649-654.

Andrade, I., Jr., Taddei, S.R., Garlet, G.P., Garlet, T.P., Teixeira, A.L., Silva, T.A., et al. (2009). CCR5 down-regulates osteoclast function in orthodontic tooth movement. *J. Dent. Res.* 88(11), 1037-1041. doi: 10.1177/0022034509346230.

Atkinson, H.F., and Ralph, W.J. (1977). In vitro strength of the human periodontal ligament. *J. Dent. Res.* 56(1), 48-52. doi: 10.1177/00220345770560011001.

Basdra, E.K. (1997). Biological reactions to orthodontic tooth movement. *J. Orofac. Orthop.* 58(1), 2-15.

Bellows, C.G., Melcher, A.H., and Aubin, J.E. (1982). Association between tension and orientation of periodontal ligament fibroblasts and exogenous collagen fibres in collagen gels in vitro. *J. Cell Sci.* 58, 125-138.

Berendsen, A.D., Smit, T.H., Walboomers, X.F., Everts, V., Jansen, J.A., and Bronckers, A.L. (2009). Three-dimensional loading model for periodontal ligament regeneration in vitro. *Tissue engineering. Part C, Methods* 15(4), 561-570. doi: 10.1089/ten.TEC.2008.0336.

Carano, A., and Siciliani, G. (1996). Effects of continuous and intermittent forces on human fibroblasts in vitro. *Eur. J. Orthod.* 18(1), 19-26.

Chen, Y.J., Jeng, J.H., Chang, H.H., Huang, M.Y., Tsai, F.F., and Yao, C.C. (2013). Differential regulation of collagen, lysyl oxidase and MMP-2 in human periodontal ligament cells by low- and high-level mechanical stretching. *J. Periodontal Res.* 48(4), 466-474. doi: 10.1111/jre.12028.

Duarte, W.R., Mikuni-Takagaki, Y., Kawase, T., Limura, T., Oida, S., Ohya, K., et al. (1999). Effects of mechanical stress on the mRNA expression of S100A4 and cytoskeletal components by periodontal ligament cells. *Journal of medical and dental sciences* 46(3), 117-122.

Duncan, G.W., Yen, E.H., Pritchard, E.T., and Suga, D.M. (1984). Collagen and prostaglandin synthesis in force-stressed periodontal ligament in vitro. *J. Dent. Res.* 63(5), 665-669. doi: 10.1177/00220345840630051201.

Feng, L., Zhang, Y., Kou, X., Yang, R., Liu, D., Wang, X., et al. (2017). Cadherin-11 modulates cell morphology and collagen synthesis in periodontal ligament cells under mechanical stress. *Angle Orthod.* 87(2), 193-199. doi: 10.2319/020716-107.1.

Fu, H.D., Wang, B.K., Wan, Z.Q., Lin, H., Chang, M.L., and Han, G.L. (2016). Wnt5a mediated canonical Wnt signaling pathway activation in orthodontic tooth movement: possible role in the tension force-induced bone formation. *Journal of molecular histology* 47(5), 455-466. doi: 10.1007/s10735-016-9687-y.

Gadhari, N., Charnley, M., Marelli, M., Brugger, J., and Chiquet, M. (2013). Cell shape-dependent early responses of fibroblasts to cyclic strain. *Biochim. Biophys. Acta* 1833(12), 3415-3425. doi: 10.1016/j.bbamcr.2013.10.012.

Glogauer, M., Ferrier, J., and McCulloch, C.A. (1995). Magnetic fields applied to collagen-coated ferric oxide beads induce stretch-activated Ca2+ flux in fibroblasts. *The American journal of physiology* 269(5 Pt 1), C1093-1104. doi: 10.1152/ajpcell.1995.269.5.C1093.

Huang, T.H., Liu, S.L., Chen, C.L., Shie, M.Y., and Kao, C.T. (2013). Low-level laser effects on simulated orthodontic tension side periodontal ligament cells. *Photomedicine and laser surgery* 31(2), 72-77. doi: 10.1089/pho.2012.3359.

Kaku, M., Rosales Rocabado, J.M., Kitami, M., Ida, T., Akiba, Y., Yamauchi, M., et al. (2016). Mechanical Loading Stimulates Expression of Collagen Cross-Linking Associated Enzymes in Periodontal Ligament. *J. Cell. Physiol.* 231(4), 926-933. doi: 10.1002/jcp.25184.

Kletsas, D., Basdra, E.K., and Papavassiliou, A.G. (1998). Mechanical stress induces DNA synthesis in PDL fibroblasts by a mechanism unrelated to autocrine growth factor action. *FEBS letters* 430(3), 358-362.

Ku, S.J., Chang, Y.I., Chae, C.H., Kim, S.G., Park, Y.W., Jung, Y.K., et al. (2009). Static tensional forces increase osteogenic gene expression in three-dimensional periodontal ligament cell culture. *BMB Rep.* 42(7), 427-432. doi: 10.5483/bmbrep.2009.42.7.427.

Lew, A.M., Glogauer, M., and McUlloch, C.A. (1999). Specific inhibition of skeletal alpha-actin gene transcription by applied mechanical forces through integrins and actin. *The Biochemical journal* 341 (Pt 3), 647-653.

Li, M., Zhang, C., and Yang, Y. (2019a). Effects of mechanical forces on osteogenesis and osteoclastogenesis in human periodontal ligament fibroblasts: A systematic review of in vitro studies. *Bone Joint Res* 8(1), 19-31. doi: 10.1302/2046-3758.81.Bjr-2018-0060.R1.

Li, Q., Han, G., Liu, D., and Zhou, Y. (2019b). Force-induced decline of TEA domain family member 1 contributes to osteoclastogenesis via regulation of Osteoprotegerin. *Arch. Oral Biol.* 100, 23-32. doi: 10.1016/j.archoralbio.2019.01.020.

Liu, F., Wen, F., He, D., Liu, D., Yang, R., Wang, X., et al. (2017). Force-Induced H2S by PDLSCs Modifies Osteoclastic Activity during Tooth Movement. *J. Dent. Res.* 96(6), 694-702. doi: 10.1177/0022034517690388.

Loesberg, W.A., Walboomers, X.F., van Loon, J.J., and Jansen, J.A. (2005). The effect of combined cyclic mechanical stretching and microgrooved surface topography on the behavior of fibroblasts. *Journal of biomedical materials research. Part A* 75(3), 723-732. doi: 10.1002/jbm.a.30480.

Marciniak, J., Lossdorfer, S., Kirschneck, C., Deschner, J., Jager, A., and Wolf, M. (2019). Heat shock protein 70 dampens the inflammatory response of human PDL cells to mechanical loading in vitro. *J. Periodontal Res.* doi: 10.1111/jre.12648.

Norton, L.A., Andersen, K.L., Arenholt-Bindslev, D., Andersen, L., and Melsen, B. (1995). A methodical study of shape changes in human oral cells perturbed by a simulated orthodontic strain in vitro. *Arch. Oral Biol.* 40(9), 863-872.

Norton, L.A., Andersen, K.L., Melsen, B., Bindslev, D.A., and Celis, J.E. (1990). Buccal mucosa fibroblasts and periodontal ligament cells perturbed by tensile stimuli in vitro. *Scandinavian journal of dental research* 98(1), 36-46.
Pavasant, P., and Yongchaitrakul, T. (2011). Role of mechanical stress on the function of periodontal ligament cells. *Periodontol. 2000* 56(1), 154-165. doi: 10.1111/j.1600-0757.2010.00374.x.
Pavlin, D., and Gluhak-Heinrich, J. (2001). Effect of mechanical loading on periodontal cells. *Critical reviews in oral biology and medicine : an official publication of the American Association of Oral Biologists* 12(5), 414-424.
Pender, N., and McCulloch, C.A. (1991). Quantitation of actin polymerization in two human fibroblast sub-types responding to mechanical stretching. *J. Cell Sci.* 100 (Pt 1), 187-193.
Rosselli-Murai, L.K., Almeida, L.O., Zagni, C., Galindo-Moreno, P., Padial-Molina, M., Volk, S.L., et al. (2013). Periostin responds to mechanical stress and tension by activating the MTOR signaling pathway. *PloS One* 8(12), e83580. doi: 10.1371/journal.pone.0083580.
Saminathan, A., Sriram, G., Vinoth, J.K., Cao, T., and Meikle, M.C. (2015). Engineering the periodontal ligament in hyaluronan-gelatin-type I collagen constructs: upregulation of apoptosis and alterations in gene expression by cyclic compressive strain. *Tissue engineering. Part A* 21(3-4), 518-529. doi: 10.1089/ten.TEA.2014.0221.
Takimoto, A., Kawatsu, M., Yoshimoto, Y., Kawamoto, T., Seiryu, M., Takano-Yamamoto, T., et al. (2015). Scleraxis and osterix antagonistically regulate tensile force-responsive remodeling of the periodontal ligament and alveolar bone. *Development (Cambridge, England)* 142(4), 787-796. doi: 10.1242/dev.116228.
Von den Hoff, J.W. (2003). Effects of mechanical tension on matrix degradation by human periodontal ligament cells cultured in collagen gels. *J. Periodontal Res.* 38(5), 449-457.
Wan, W., He, C., Du, C., Wang, Y., Wu, S., Wang, T., et al. (2019). Effect of ILK on small-molecule metabolism of human periodontal ligament fibroblasts with mechanical stretching. *J. Periodontal Res.* doi: 10.1111/jre.12706.
Wang, T., Li, G., Chen, J., Lin, Z., Qin, H., and Ji, J. (2016). Three-dimensional stress In vitro promotes the proliferation and differentiation of periodontal ligament stem cells implanted by bioactive glass. *Cellular and molecular biology (Noisy-le-Grand, France)* 62(10), 62-67.
Wang, Z., Maruyama, K., Sakisaka, Y., Suzuki, S., Tada, H., Suto, M., et al. (2019). Cyclic Stretch Force Induces Periodontal Ligament Cells to Secrete Exosomes That Suppress IL-1beta Production Through the Inhibition of the NF-kappaB Signaling Pathway in Macrophages. *Frontiers in immunology* 10, 1310. doi: 10.3389/fimmu.2019.01310.
Wolf, M., Lossdorfer, S., Craveiro, R., Gotz, W., and Jager, A. (2013). Regulation of macrophage migration and activity by high-mobility group box 1 protein released from periodontal ligament cells during orthodontically induced periodontal repair: an in vitro and in vivo experimental study. *J. Orofac. Orthop.* 74(5), 420-434. doi: 10.1007/s00056-013-0167-7.
Wongkhantee, S., Yongchaitrakul, T., and Pavasant, P. (2008). Mechanical stress induces osteopontin via ATP/P2Y1 in periodontal cells. *J. Dent. Res.* 87(6), 564-568. doi: 10.1177/154405910808700601.
Xu, H., Han, X., Meng, Y., Gao, L., Guo, Y., Jing, Y., et al. (2014). Favorable effect of myofibroblasts on collagen synthesis and osteocalcin production in the periodontal ligament. *Am. J. Orthod. Dentofacial Orthop.* 145(4), 469-479. doi: 10.1016/j.ajodo.2013.12.019.
Xu, Y., Shen, J., Muhammed, F.K., Zheng, B., Zhang, Y., and Liu, Y. (2017). Effect of orthodontic force on the expression of PI3K, Akt, and P70S6 K in the human periodontal ligament during orthodontic loading. *Cell Biochem. Funct.* 35(7), 372-377. doi: 10.1002/cbf.3284.
Yamaguchi, M., and Kasai, K. (2005). Inflammation in periodontal tissues in response to mechanical forces. *Arch. Immunol. Ther. Exp. (Warsz.)* 53(5), 388-398.
Yang, L., Yang, Y., Wang, S., Li, Y., and Zhao, Z. (2015). In vitro mechanical loading models for periodontal ligament cells: from two-dimensional to three-dimensional models. *Arch. Oral Biol.* 60(3), 416-424. doi: 10.1016/j.archoralbio.2014.11.012.
Zhao, Z., Fan, Y., Bai, D., Wang, J., and Li, Y. (2008). The adaptive response of periodontal ligament to orthodontic force loading - a combined biomechanical and biological study. *Clinical biomechanics (Bristol, Avon)* 23 Suppl 1, S59-66. doi: 10.1016/j.clinbiomech.2007.10.016.
Zhong, W., Xu, C., Zhang, F., Jiang, X., Zhang, X., and Ye, D. (2008). Cyclic stretching force-induced early apoptosis in human periodontal ligament cells. *Oral diseases* 14(3), 270-276. doi: 10.1111/j.1601-0825.2007.01375.x.
