# Supplementary material for: Effect of Tension on Human Periodontal Ligament Cells: Systematic Review and Network Analysis
Source: Front Bioeng Biotechnol. 2021 Aug 27;9:695053. doi: 10.3389/fbioe.2021.695053 (PMC8429507; doi:10.3389/fbioe.2021.695053)
Supplement: Supplementary file 7 [file DataSheet5.pdf]

# Supplement 5:

# Summary on force application.

Herein, the studies included were summarized in terms of force apparatus used, force duration and force magnitude for both dynamic/static and equibiaxial/uniaxial tension.

## Contents

Table S5.1 Force apparatuses used to apply dynamic equibiaxial tension 2
Table S5.2 Force apparatuses used to apply dynamic uniaxial tension 3
Table S5.3 Force apparatuses used to apply static equibiaxial tension 3
Table S5.4 Force apparatuses used to apply static uniaxial tension 3
Table S5.5 Maximal force magnitude and force duration applied using dynamic equibiaxial tension 4
Table S5.6 Maximal force magnitude and force duration applied using dynamic uniaxial tension 4
Table S5.7 Maximal force magnitude and force duration applied using static equibiaxial tension 5
Table S5.8 Maximal force magnitude and force duration applied using static uniaxial tension 5
References 6

## Table S5.1 Force apparatuses used to apply dynamic equibiaxial tension

| Type of apparatus | Publications | Number | Subtotal |
|---|---|---|---|
| Flexcell FX-5000 Tension Unit<br>Flexcell Tension Plus System FX-5000T<br>Flexercell FX-4000 Strain Unit<br>Flexcell FX-4000T<br>Flexcell FX 3000 | Chang et al. (2015), Jiang and Hua (2016), Lee et al. (2015), Li et al. (2013), Li et al. (2014), Liu et al. (2012), Liu et al. (2017), Padial-Molina et al. (2013), Pan et al. (2014), Ren et al. (2015), Shen et al. (2014), Wang et al. (2013), Wang et al. (2019a), Wang et al. (2019b), Wei et al. (2014), Wei et al. (2015), Wu et al. (2015), Wu et al. (2019a), Xu et al. (2017), Yang et al. (2015), Yang et al. (2016), Yang et al. (2018), Yu et al. (2018), Zhao et al. (2016), Zhuang et al. (2019) | 25 | 53 |
| Flexercell Strain Unit | Abiko et al. (1998), Agarwal et al. (2003), Chang et al. (2017), Chiba and Mitani (2004), Doi et al. (2003), Kanzaki et al. (2006), Kikuiri et al. (2000), Long et al. (2002), Long et al. (2001), Miura et al. (2000), Ohzeki et al. (1999), Ozawa et al. (1997), Shimizu et al. (1998), Shimizu et al. (1997), Shimizu et al. (1995), Shimizu et al. (1994), Tsuji et al. (2004), Yamaguchi et al. (1996), Yamaguchi and Shimizu (1994), Yamaguchi et al. (1994), Yamaguchi et al. (2002), Yamashiro et al. (2007), Yoshino et al. (2003), Kanzaki et al. (2019) | 24 | |
| Flexercell Strain Unit Model FX-2000 | Kim et al. (2007), Matsuda et al. (1998b), Matsuda et al. (1998a), Kaku et al. (2019) | 4 | |
| "*Cell Strain Unit*" (CSU) (silicone rubber membrane in disk) | Hao et al. (2009), Ma et al. (2015), Wu et al. (2017), Wu et al. (2016), Xu et al. (2012), Xu et al. (2011), Zhao et al. (2017), Wu et al. (2019b) | 8 | 10 |
| Circularly clamped compliant membrane with spherical cap and vacuum (Tecoflex membrane) | Howard et al. (1998) | 1 | |
| Plastic culture cylinder with elastic silicone membrane and movable plate | He et al. (2004) | 1 | |
| CESTRA cell strain device (based on Bioflex® plates) | Deschner et al. (2012), Nogueira et al. (2014a), Nogueira et al. (2014b), Nokhbehsaim et al. (2012), Nokhbehsaim et al. (2011b), Nokhbehsaim et al. (2011a), Nokhbehsaim et al. (2010), Memmert et al. (2020) | 8 | 9 |
| "*Cell Extender*" (Bioflex-based) | Wada et al. (2017) | 1 | |
| | **Total number** | | **72** |

## Table S5.2 Force apparatuses used to apply dynamic uniaxial tension

| Type of apparatus | Publications | Number | Subtotal |
|---|---|---|---|
| STREX STB-140 | Arima et al. (2019), Goto et al. (2011), Nemoto et al. (2010), Monnouchi et al. (2015), Monnouchi et al. (2011), Nakashima et al. (2009), Suzuki et al. (2014), Tsuruga et al. (2012), Tsuruga et al. (2009), Yuda et al. (2015) | 10 | 10 |
| Six station stretching apparatus (silicone dishes and moving clamp) | Konstantonis et al. (2014), Papadopoulou et al. (2017), Papadopoulou et al. (2019) | 3 | 10 |
| Uniaxial stretch apparatus (Chulalongkorn University, silicone membrane) | Tantilertanant et al. (2019a), Tantilertanant et al. (2019b) | 2 | |
| "*A new model*" (silicone membrane and motor) | Yang et al. (2010), Yang et al. (2006) | 2 | |
| Scholertec NS-350 (Scholertec, silicone membrane) | Fujihara et al. (2010) | 1 | |
| "*Custom-made tensile device*" poly dimethyl siloxane (PDMS) gel and motor | Li et al. (2015) | 1 | |
| "*custom-built bioreactor system and linear actuator*" | Pelaez et al. (2017) | 1 | |
| Flexcell FX-4000 strain unit FX-5000T Flexcell Tension Plus unit | Chen et al. (2015), Cho et al. (2010), Lee et al. (2012), Pinkerton et al. (2008), Saminathan et al. (2012), Sun et al. (2016), Sun et al. (2017), Wescott et al. (2007) | 8 | 8 |
| Four-point bending system | He et al. (2019), Meng et al. (2010), Tang et al. (2012), Wang et al. (2011), Wang et al. (2018), Xu et al. (2015) | 6 | 6 |
| | | **Total number** | **34** |

## Table S5.3 Force apparatuses used to apply static equibiaxial tension

| Type of apparatus | Publications | Number | Subtotal |
|---|---|---|---|
| Flexercell Strain Unit | Bolcato-Bellemin et al. (2000), Yamaguchi et al. (1997) | 2 | 11 |
| Flexcell FX-5000 Tension System<br>Flexercell Strain Unit FX5000-T<br>FX-4000 Tension Plus System<br>Flexercell Strain Unit FX 3000 | Huelter-Hassler et al. (2017), Hülter-Hassler et al. (2017), Jacobs et al. (2013), Jacobs et al. (2014), Jacobs et al. (2015), Jacobs et al. (2018), Kook and Lee (2012), Liao and Hua (2013), Symmank et al. (2019) | 9 | |
| Petriperm dish and template | Basdra et al. (1996), Basdra et al. (1995), Diercke et al. (2011), Kletsas et al. (2002), Molina et al. (2001), Ngan et al. (1990), Peverali et al. (2001), Ritter et al. (2007), Spencer and Lallier (2009), Yamaguchi et al. (2004) | 10 | 10 |
| "*Cell Extender*" (Bioflex-based) | Narimiya et al. (2017), Wada et al. (2017) | 2 | 7 |
| CESTRA cell strain device (Bioflex-based) | Memmert et al. (2019), Memmert et al. (2020) | 2 | |
| "*loading platform with cylindrical posts*" (University of Bonn, Bioflex-based) | Rath-Deschner et al. (2009), Wolf et al. (2014) | 2 | |
| custom-made spherical cap silicone stamps Bioflex-based) | Nazet et al. (2020) | 1 | |
| Lumox culture dishes | Steinberg et al. (2011), Ziegler et al. (2010) | 2 | 2 |
| "*a tension incubator*" | Chen et al. (2014) | 1 | 1 |
| | | **Total number** | **31** |

## Table S5.4 Force apparatuses used to apply static uniaxial tension

| Type of apparatus | Publications | Number |
|---|---|---|
| "*in-house designed device*" (silicone dishes and moving clamp) | Papadopoulou et al. (2017) | 1 |
| STREX system | Takano et al. (2009) | 1 |
| | **Total number** | **2** |

Table S5.5 Maximal force magnitude and force duration applied using dynamic equibiaxial tension depending on the frequency of force application

| Frequency | Publications | Number |
|---|---|---|
| 0.005 Hz | Agarwal et al. (2003) (48h; 3%, 6%, 8%, 15%), Long et al. (2002) (48h; 6%), Long et al. (2001) (48h; 6%) | 3 |
| 0.05 Hz | Deschner et al. (2012) (24h; 3%), Nogueira et al. (2014a) (3d; 3%, 20%), Nogueira et al. (2014b) (3d; 3%, 20%), Nokhbehsaim et al. (2010) (6d; 3%, 20%), Nokhbehsaim et al. (2011a) (6d; 3%), Nokhbehsaim et al. (2011b) (6d; 3%, 20%), Nokhbehsaim et al. (2012) (6d; 3%, 20%) | 7 |
| 0.1 Hz | Abiko et al. (1998) (5d; 18%), Chang et al. (2015) (72h; 12%), Chang et al. (2017) (72h; 12%), Hao et al. (2009) (48h; 1%, 10%, 20%), Jiang and Hua (2016) (48h; 5%), Kikuiri et al. (2000) (12h; 18%), Kim et al. (2007) (6d; 9%), Lee et al. (2015) (48h; 12%), Liu et al. (2012) (24h; 12%), Liu et al. (2017) (12h; 6%, 8%, 10%, 12%, 14%), Ma et al. (2015) (24h; 10%), Matsuda et al. (1998a) (1h; 9%), Matsuda et al. (1998b) (6d; 9%,18%), Memmert et al. (2020) (24h; 3%), Miura et al. (2000) (5d; 9%, 18%), Ohzeki et al. (1999) (5d; 9%, 18%), Ozawa et al. (1997) (5d; 18%), Padial-Molina et al. (2013) (7d; 14%), Pan et al. (2014) (24h; 10%), Shen et al. (2014) (24h; 12%), Shimizu et al. (1994) (5d: 9%, 18%), Shimizu et al. (1995) (5d; 18%), Shimizu et al. (1997) (5d; 9%, 18%), Shimizu et al. (1998) (5d; 18%), Wang et al. (2013) (24h; 20%), Wang et al. (2019b) (48h; 12%), Wu et al. (2015) (24h; 10%), Wu et al. (2016) (24h; 20%), Wu et al. (2017) (24h; 1%, 10%, 20%), Wu et al. (2019a) (24h; 10%), Wu et al. (2019b) (24h; 20%), Xu et al. (2011) (24h; 20%), Xu et al. (2012) (24h; 1%, 10%, 20%), Yamaguchi and Shimizu (1994) (3d; 24%), Yamaguchi et al. (1994) (5d; 18%), Yamaguchi et al. (1996) (5d; 24%), Yamashiro et al. (2007) (16h; 18%), Yang et al. (2015) (48h; 12%), Yang et al. (2018) (72h;10%), Zhao et al. (2016) (24h; 20%), Zhao et al. (2017) (24h; 10%, 20%), Zhuang et al. (2019) (24h; 20%) | 42 |
| 0.17 Hz (1/6Hz) | Tsuji et al. (2004) (48h; 20%) | 1 |
| 0.2 Hz | Yoshino et al. (2003) (48h; 7%, 14%, 21%) | 1 |
| 0.5 Hz | Chiba and Mitani (2004) (5d; 15%), Doi et al. (2003) (48h; 7.2 kPa, 15.4 kPa), He et al. (2004) (24h; 10%), Howard et al. (1998) (24h; 5%, 10%), Kaku et al. (2019) (48h; 12%), Kanzaki et al. (2006) (72h; 15%), Kanzaki et al. (2019) (24h; 15%), Li et al. (2013) (48h; 10%), Li et al. (2014) (48h; 10%), Ren et al. (2015) (24h; 10%), Wada et al. (2017) (n.g; 15%), Xu et al. (2017) (48h; 10%), Yamaguchi et al. (2002) (6h; 15%), Yang et al. (2016) (24h; 10%), Yu et al. (2018) (72h; 12%) | 15 |
| 1 Hz | Wang et al. (2019a) (12h; 10%), Wei et al. (2014) (12h; 10%), Wei et al. (2015) (48h; 10%) | 3 |
| | **Total number** | **72** |

Table S5.6 Maximal force magnitude and force duration applied using dynamic uniaxial tension depending on the frequency of force application

| Frequency | Publications | Number |
|---|---|---|
| 0.005 Hz | Li et al. (2015) (24h; 5%), Yang et al. (2010) (24h; 12%) | 2 |
| 0.01 Hz | Nakashima et al. (2009) (7d; 5%), Pinkerton et al. (2008) (24h; 12%), Saminathan et al. (2012) (24h; 12%), Wescott et al. (2007) (24h; 12%) | 4 |
| 1/60 Hz (0.017 Hz) | Goto et al. (2011) (7d; 5%), Nemoto et al. (2010) (7d; 5%), Suzuki et al. (2014) (24h; 3%, 5%, 10%), Tsuruga et al. (2009) (7d; 5%), Tsuruga et al. (2012) (7d; 5%) | 5 |
| 0.05 Hz | Yang et al. (2006) (24h; 310-320 grams force) | 1 |
| 0.1 Hz | Chen et al. (2015) (24h; 12%) | 1 |
| 0.2 Hz | Cho et al. (2010) (48h; 3%, 6%, 12%, 15%), Lee et al. (2012) (48h; 33%, 6%, 12%,15%) | 2 |
| 0.5 Hz | Arima et al. (2019) (24h; 10%), Fujihara et al. (2010) (48h; 10%), He et al. (2019) (3h; 0.2%), Meng et al. (2010) (12h; 0.4%), Pelaez et al. (2017) (2h; 5%), Sun et al. (2016) (5d; 12%), Sun et al. (2017) (48h; 12%), Tang et al. (2012) (24h; 0.3%), Wang et al. (2011) (2h; 0.5%), Wang et al. (2018) (6h; 0.4%), Xu et al. (2015) (12h; 0.2%, 0.4%) | 11 |
| 1 Hz | Konstantonis et al. (2014) (12h; 8%), Monnouchi et al. (2011) (1h; 8%, 12%), Monnouchi et al. (2015) (1h; 8%), Papadopoulou et al. (2017) (3h; 8%), Papadopoulou et al. (2019) (18h; 8%), Tantilertanant et al. (2019a) (48h; 10%), Tantilertanant et al. (2019b) (6h; 10%), Yuda et al. (2015) (3h; 8%) | 8 |
| | **Total number** | **34** |

Table S5.7 Maximal force magnitude and force duration applied using static equibiaxial tension

| Magnitude | Publications | Number |
|---|---|---|
| 0.28 % | Ngan et al. (1990) (1h), Yamaguchi et al. (2004) (12h) | 2 |
| 0.95 % | Ngan et al. (1990) (1h), Yamaguchi et al. (2004) (12h) | 2 |
| 1 % | Jacobs et al. (2013) (12h), Jacobs et al. (2014) (12h) | 2 |
| 1.09 % | Ngan et al. (1990) (1h), Yamaguchi et al. (2004) (12h) | 2 |
| 1.5 % | Kook and Lee (2012) (1h), Liao and Hua (2013) (2h) | 2 |
| 1.72 % | Ngan et al. (1990) (1h), Yamaguchi et al. (2004) (12h) | 2 |
| -100 kPa | Chen et al. (2014) (15d) | 1 |
| 20 kPa | Bolcato-Bellemin et al. (2000) (12h) | 1 |
| 2.5 % | Basdra et al. (1995) (1h), Basdra et al. (1996) (12h), Diercke et al. (2011) (72h), Huelter-Hassler et al. (2017) (24h), Kletsas et al. (2002) (24h), Molina et al. (2001) (72h), Peverali et al. (2001) (0.5h), Ritter et al. (2007) (6h), Steinberg et al. (2011) (24h), Yamaguchi et al. (2004) (24h), Ziegler et al. (2010) (6h) | 11 |
| 3 % | Jacobs et al. (2018) (12h), Kook and Lee (2012) (1h), Rath-Deschner et al. (2009) (24h), Memmert et al. (2019) (24h), Memmert et al. (2020) (24h) | 5 |
| 5 % | Jacobs et al. (2015) (12h), Jacobs et al. (2014) (12h), Jacobs et al. (2013) (12h), Kook and Lee (2012) (1h), Symmank et al. (2019) (12h) | 5 |
| 7 % | Nazet et al. (2020) (48h) | 1 |
| 9 % | Yamaguchi et al. (1997) (5d) | 1 |
| 10 % | Jacobs et al. (2013) (12h), Jacobs et al. (2014) (12h), Jacobs et al. (2015) (12h), Kook and Lee (2012) (1h), Nazet et al. (2020) (48h), Spencer and Lallier (2009) (12h) | 6 |
| 15 % | Narimiya et al. (2017) (24h), Wada et al. (2017) (24h) | 2 |
| 16 % | Nazet et al. (2020) (48h) | 1 |
| 18 % | Yamaguchi et al. (1997) (5d) | 1 |
| 20 % | Memmert et al. (2019) (24h), Memmert et al. (2020) (24h), Rath-Deschner et al. (2009) (24h), Wolf et al. (2014) (8h) | 4 |
| 35 % | Nazet et al. (2020) (72h) | 1 |
| | **Total number** | **52** |

Table S5.8 Maximal force magnitude and force duration applied using static uniaxial tension

| Magnitude | Publications | Number |
|---|---|---|
| 5 % | Takano et al. (2009) (12h) | 1 |
| 8 % | Papadopoulou et al. (2017) (3h) | 1 |
| 10 % | Takano et al. (2009) (12h) | 1 |
| | **Total number** | **3** |

Dynamic equibiaxial tension (72): longest duration and mainly used magnitude

Dynamic uniaxial tension (34): longest duration and mainly used magnitude

Static equibiaxial tension (52): Magnitude and corresponding longest duration

Static uniaxial tension (3): Magnitude and corresponding longest duration

# References


Abiko, Y., Shimizu, N., Yamaguchi, M., Suzuki, H., and Takiguchi, H. (1998). Effect of aging on functional changes of periodontal tissue cells. *Ann. Periodontol.* 3(1), 350-369. doi: 10.1902/annals.1998.3.1.350.

Agarwal, S., Long, P., Seyedain, A., Piesco, N., Shree, A., and Gassner, R. (2003). A central role for the nuclear factor-κB pathway in anti-inflammatory and proinflammatory actions of mechanical strain. *FASEB J.* 17(8), 899-901. doi: 10.1096/fj.02-0901fje.

Arima, M., Hasegawa, D., Yoshida, S., Mitarai, H., Tomokiyo, A., Hamano, S., et al. (2019). R-spondin 2 promotes osteoblastic differentiation of immature human periodontal ligament cells through the Wnt/beta-catenin signaling pathway. *J. Periodontal Res.* 54(2), 143-153. doi: 10.1111/jre.12611.

Basdra, E.K., Kohl, A., and Komposch, G. (1996). Mechanical stretching of periodontal ligament fibroblasts--a study on cytoskeletal involvement. *J. Orofac. Orthop.* 57(1), 24-30. doi: 10.1007/BF02189045.

Basdra, E.K., Papavassiliou, A.G., and Huber, L.A. (1995). Rab and rho GTPases are involved in specific response of periodontal ligament fibroblasts to mechanical stretching. *Biochim. Biophys. Acta* 1268(2), 209-213. doi: 10.1016/0167-4889(95)00090-f.

Bolcato-Bellemin, A.L., Elkaim, R., Abehsera, A., Fausser, J.L., Haikel, Y., and Tenenbaum, H. (2000). Expression of mRNAs encoding for alpha and beta integrin subunits, MMPs, and TIMPs in stretched human periodontal ligament and gingival fibroblasts. *J. Dent. Res.* 79(9), 1712-1716. doi: 10.1177/00220345000790091201.

Chang, M., Lin, H., Fu, H., Wang, B., Han, G., and Fan, M. (2017). MicroRNA-195-5p regulates osteogenic differentiation of periodontal ligament cells under mechanical loading. *J. Cell. Physiol.* 232(12), 3762-3774. doi: 10.1002/jcp.25856.

Chang, M., Lin, H., Luo, M., Wang, J., and Han, G. (2015). Integrated miRNA and mRNA expression profiling of tension force-induced bone formation in periodontal ligament cells. *In Vitro Cell. Dev. Biol. Anim.* 51(8), 797-807. doi: 10.1007/s11626-015-9892-0.

Chen, Y., Mohammed, A., Oubaidin, M., Evans, C.A., Zhou, X., Luan, X., et al. (2015). Cyclic stretch and compression forces alter microRNA-29 expression of human periodontal ligament cells. *Gene* 566(1), 13-17. doi: 10.1016/j.gene.2015.03.055.

Chen, Y.J., Shie, M.Y., Hung, C.J., Wu, B.C., Liu, S.L., Huang, T.H., et al. (2014). Activation of focal adhesion kinase induces extracellular signal-regulated kinase-mediated osteogenesis in tensile force-subjected periodontal ligament fibroblasts but not in osteoblasts. *J. Bone Miner. Metab.* 32(6), 671-682. doi: 10.1007/s00774-013-0549-3.

Chiba, M., and Mitani, H. (2004). Cytoskeletal changes and the system of regulation of alkaline phosphatase activity in human periodontal ligament cells induced by mechanical stress. *Cell Biochem. Funct.* 22(4), 249-256. doi: 10.1002/cbf.1097.

Cho, J.H., Lee, S.K., Lee, J.W., and Kim, E.C. (2010). The role of heme oxygenase-1 in mechanical stress- and lipopolysaccharide-induced osteogenic differentiation in human periodontal ligament cells. *Angle Orthod.* 80(4), 552-559. doi: 10.2319/091509-520.1.

Deschner, B., Rath, B., Jager, A., Deschner, J., Denecke, B., Memmert, S., et al. (2012). Gene analysis of signal transduction factors and transcription factors in periodontal ligament cells following application of dynamic strain. *J. Orofac. Orthop.* 73(6), 486-495, 497. doi: 10.1007/s00056-012-0104-1.

Diercke, K., Kohl, A., Lux, C.J., and Erber, R. (2011). Strain-dependent up-regulation of ephrin-B2 protein in periodontal ligament fibroblasts contributes to osteogenesis during tooth movement. *J. Biol. Chem.* 286(43), 37651-37664. doi: 10.1074/jbc.M110.166900.

Doi, T., Ohno, S., Tanimoto, K., Honda, K., Tanaka, N., Ohno-Nakahara, M., et al. (2003). Mechanical stimuli enhances the expression of RGD-CAP/betaig-h3 in the periodontal ligament. *Arch. Oral Biol.* 48(8), 573-579. doi: 10.1016/s0003-9969(03)00103-1.

Fujihara, C., Yamada, S., Ozaki, N., Takeshita, N., Kawaki, H., Takano-Yamamoto, T., et al. (2010). Role of mechanical stress-induced glutamate signaling-associated molecules in cytodifferentiation of periodontal ligament cells. *J. Biol. Chem.* 285(36), 28286-28297. doi: 10.1074/jbc.M109.097303.

Goto, K.T., Kajiya, H., Nemoto, T., Tsutsumi, T., Tsuzuki, T., Sato, H., et al. (2011). Hyperocclusion stimulates osteoclastogenesis via CCL2 expression. *J. Dent. Res.* 90(6), 793-798. doi: 10.1177/0022034511400742.

Hao, Y., Xu, C., Sun, S.Y., and Zhang, F.Q. (2009). Cyclic stretching force induces apoptosis in human periodontal ligament cells via caspase-9. *Arch. Oral Biol.* 54(9), 864-870. doi: 10.1016/j.archoralbio.2009.05.012.

He, Y., Macarak, E.J., Korostoff, J.M., and Howard, P.S. (2004). Compression and tension: differential effects on matrix accumulation by periodontal ligament fibroblasts in vitro. *Connect Tissue Res.* 45(1), 28-39. doi: 10.1080/03008200490278124.

He, Y., Xu, H., Xiang, Z., Yu, H., Xu, L., Guo, Y., et al. (2019). YAP regulates periodontal ligament cell differentiation into myofibroblast interacted with RhoA/ROCK pathway. *J. Cell. Physiol.* 234(4), 5086-5096. doi: 10.1002/jcp.27312.

Howard, P.S., Kucich, U., Taliwal, R., and Korostoff, J.M. (1998). Mechanical forces alter extracellular matrix synthesis by human periodontal ligament fibroblasts. *J. Periodontal Res.* 33(8), 500-508. doi: 10.1111/j.1600-0765.1998.tb02350.x.

Huelter-Hassler, D., Tomakidi, P., Steinberg, T., and Jung, B.A. (2017). Orthodontic strain affects the Hippo-pathway effector YAP concomitant with proliferation in human periodontal ligament fibroblasts. *Eur. J. Orthod.* 39(3), 251-257. doi: 10.1093/ejo/cjx012.

Hülter-Hassler, D., Wein, M., Schulz, S.D., Proksch, S., Steinberg, T., Jung, B.A., et al. (2017). Biomechanical strain-induced modulation of proliferation coincides with an ERK1/2-independent nuclear YAP localization. *Exp. Cell Res.* 361(1), 93-100. doi: 10.1016/j.yexcr.2017.10.006.

Jacobs, C., Grimm, S., Ziebart, T., Walter, C., and Wehrbein, H. (2013). Osteogenic differentiation of periodontal fibroblasts is dependent on the strength of mechanical strain. *Arch. Oral Biol.* 58(7), 896-904. doi: 10.1016/j.archoralbio.2013.01.009.

Jacobs, C., Schramm, S., Dirks, I., Walter, C., Pabst, A., Meila, D., et al. (2018). Mechanical loading increases pro-inflammatory effects of nitrogen-containing bisphosphonate in human periodontal fibroblasts. *Clin. Oral Investig.* 22(2), 901-907. doi: 10.1007/s00784-017-2168-1.

Jacobs, C., Walter, C., Ziebart, T., Dirks, I., Schramm, S., Grimm, S., et al. (2015). Mechanical loading influences the effects of bisphosphonates on human periodontal ligament fibroblasts. *Clin. Oral Investig.* 19(3), 699-708. doi: 10.1007/s00784-014-1284-4.

Jacobs, C., Walter, C., Ziebart, T., Grimm, S., Meila, D., Krieger, E., et al. (2014). Induction of IL-6 and MMP-8 in human periodontal fibroblasts by static tensile strain. *Clin. Oral Investig.* 18(3), 901-908. doi: 10.1007/s00784-013-1032-1.

Jiang, Z., and Hua, Y. (2016). Hydrogen sulfide promotes osteogenic differentiation of human periodontal ligament cells via p38-MAPK signaling pathway under proper tension stimulation. *Arch. Oral Biol.* 72, 8-13. doi: 10.1016/j.archoralbio.2016.08.008.

Kaku, M., Yamamoto, T., Yashima, Y., Izumino, J., Kagawa, H., Ikeda, K., et al. (2019). Acetaminophen reduces apical root resorption during orthodontic tooth movement in rats. *Arch. Oral Biol.* 102, 83-92. doi: 10.1016/j.archoralbio.2019.04.002.

Kanzaki, H., Chiba, M., Sato, A., Miyagawa, A., Arai, K., Nukatsuka, S., et al. (2006). Cyclical tensile force on periodontal ligament cells inhibits osteoclastogenesis through OPG induction. *J. Dent. Res.* 85(5), 457-462. doi: 10.1177/154405910608500512.

Kanzaki, H., Wada, S., Yamaguchi, Y., Katsumata, Y., Itohiya, K., Fukaya, S., et al. (2019). Compression and tension variably alter Osteoprotegerin expression via miR-3198 in periodontal ligament cells. *BMC Mol. Cell Biol.* 20(1), 6. doi: 10.1186/s12860-019-0187-2.

Kikuiri, T., Hasegawa, T., Yoshimura, Y., Shirakawa, T., and Oguchi, H. (2000). Cyclic tension force activates nitric oxide production in

cultured human periodontal ligament cells. *J. Periodontol.* 71(4), 533-539. doi: 10.1902/jop.2000.71.4.533.
Kim, H.J., Choi, Y.S., Jeong, M.J., Kim, B.O., Lim, S.H., Kim, D.K., et al. (2007). Expression of UNCL during development of periodontal tissue and response of periodontal ligament fibroblasts to mechanical stress in vivo and in vitro. *Cell Tissue Res.* 327(1), 25-31. doi: 10.1007/s00441-006-0304-3.
Kletsas, D., Basdra, E.K., and Papavassiliou, A.G. (2002). Effect of protein kinase inhibitors on the stretch-elicited c-Fos and c-Jun up-regulation in human PDL osteoblast-like cells. *J. Cell. Physiol.* 190(3), 313-321. doi: 10.1002/jcp.10052.
Konstantonis, D., Papadopoulou, A., Makou, M., Eliades, T., Basdra, E., and Kletsas, D. (2014). The role of cellular senescence on the cyclic stretching-mediated activation of MAPK and ALP expression and activity in human periodontal ligament fibroblasts. *Exp. Gerontol.* 57, 175-180. doi: 10.1016/j.exger.2014.05.010.
Kook, S.H., and Lee, J.C. (2012). Tensile force inhibits the proliferation of human periodontal ligament fibroblasts through Ras-p38 MAPK up-regulation. *J. Cell. Physiol.* 227(3), 1098-1106. doi: 10.1002/jcp.22829.
Lee, S.I., Park, K.H., Kim, S.J., Kang, Y.G., Lee, Y.M., and Kim, E.C. (2012). Mechanical stress-activated immune response genes via Sirtuin 1 expression in human periodontal ligament cells. *Clin. Exp. Immunol.* 168(1), 113-124. doi: 10.1111/j.1365-2249.2011.04549.x.
Lee, S.Y., Yoo, H.I., and Kim, S.H. (2015). CCR5-CCL Axis in PDL during Orthodontic Biophysical Force Application. *J. Dent. Res.* 94(12), 1715-1723. doi: 10.1177/0022034515603926.
Li, L., Han, M., Li, S., Wang, L., and Xu, Y. (2013). Cyclic tensile stress during physiological occlusal force enhances osteogenic differentiation of human periodontal ligament cells via ERK1/2-Elk1 MAPK pathway. *DNA Cell Biol.* 32(9), 488-497. doi: 10.1089/dna.2013.2070.
Li, L., Han, M.X., Li, S., Xu, Y., and Wang, L. (2014). Hypoxia regulates the proliferation and osteogenic differentiation of human periodontal ligament cells under cyclic tensile stress via mitogen-activated protein kinase pathways. *J. Periodontol.* 85(3), 498-508. doi: 10.1902/jop.2013.130048.
Li, S., Zhang, H., Li, S., Yang, Y., Huo, B., and Zhang, D. (2015). Connexin 43 and ERK regulate tension-induced signal transduction in human periodontal ligament fibroblasts. *J. Orthop. Res.* 33(7), 1008-1014. doi: 10.1002/jor.22830.
Liao, C., and Hua, Y. (2013). Effect of hydrogen sulphide on the expression of osteoprotegerin and receptor activator of NF-kappaB ligand in human periodontal ligament cells induced by tension-force stimulation. *Arch. Oral Biol.* 58(12), 1784-1790. doi: 10.1016/j.archoralbio.2013.08.004.
Liu, J., Li, Q., Liu, S., Gao, J., Qin, W., Song, Y., et al. (2017). Periodontal Ligament Stem Cells in the Periodontitis Microenvironment Are Sensitive to Static Mechanical Strain. *Stem Cells Int.* 2017, 1380851. doi: 10.1155/2017/1380851.
Liu, M., Dai, J., Lin, Y., Yang, L., Dong, H., Li, Y., et al. (2012). Effect of the cyclic stretch on the expression of osteogenesis genes in human periodontal ligament cells. *Gene* 491(2), 187-193. doi: 10.1016/j.gene.2011.09.031.
Long, P., Hu, J., Piesco, N., Buckley, M., and Agarwal, S. (2001). Low magnitude of tensile strain inhibits IL-1beta-dependent induction of pro-inflammatory cytokines and induces synthesis of IL-10 in human periodontal ligament cells in vitro. *J. Dent. Res.* 80(5), 1416-1420. doi: 10.1177/00220345010800050601.
Long, P., Liu, F., Piesco, N.P., Kapur, R., and Agarwal, S. (2002). Signaling by mechanical strain involves transcriptional regulation of proinflammatory genes in human periodontal ligament cells in vitro. *Bone* 30(4), 547-552. doi: 10.1016/s8756-3282(02)00673-7.
Ma, J., Zhao, D., Wu, Y., Xu, C., and Zhang, F. (2015). Cyclic stretch induced gene expression of extracellular matrix and adhesion molecules in human periodontal ligament cells. *Arch. Oral Biol.* 60(3), 447-455. doi: 10.1016/j.archoralbio.2014.11.019.
Matsuda, N., Morita, N., Matsuda, K., and Watanabe, M. (1998a). Proliferation and differentiation of human osteoblastic cells associated with differential activation of MAP kinases in response to epidermal growth factor, hypoxia, and mechanical stress in vitro. *Biochem. Biophys. Res. Commun.* 249(2), 350-354. doi: 10.1006/bbrc.1998.9151.
Matsuda, N., Yokoyama, K., Takeshita, S., and Watanabe, M. (1998b). Role of epidermal growth factor and its receptor in mechanical stress-induced differentiation of human periodontal ligament cells in vitro. *Arch. Oral Biol.* 43(12), 987-997. doi: 10.1016/s0003-9969(98)00079-x.
Memmert, S., Damanaki, A., Weykopf, B., Rath-Deschner, B., Nokhbehsaim, M., Gotz, W., et al. (2019). Autophagy in periodontal ligament fibroblasts under biomechanical loading. *Cell Tissue Res.* doi: 10.1007/s00441-019-03063-1.
Memmert, S., Nogueira, A.V.B., Damanaki, A., Nokhbehsaim, M., Rath-Deschner, B., Götz, W., et al. (2020). Regulation of the autophagy-marker Sequestosome 1 in periodontal cells and tissues by biomechanical loading. *J. Orofac. Orthop.* 81(1), 10-21. doi: 10.1007/s00056-019-00197-3.
Meng, Y., Han, X., Huang, L., Bai, D., Yu, H., He, Y., et al. (2010). Orthodontic mechanical tension effects on the myofibroblast expression of alpha-smooth muscle actin. *Angle Orthod.* 80(5), 912-918. doi: 10.2319/101609-578.1.
Miura, S., Yamaguchi, M., Shimizu, N., and Abiko, Y. (2000). Mechanical stress enhances expression and production of plasminogen activator in aging human periodontal ligament cells. *Mech. Ageing Dev.* 112(3), 217-231. doi: 10.1016/s0047-6374(99)00095-0.
Molina, T., Kabsch, K., Alonso, A., Kohl, A., Komposch, G., and Tomakidi, P. (2001). Topographic changes of focal adhesion components and modulation of p125FAK activation in stretched human periodontal ligament fibroblasts. *J. Dent. Res.* 80(11), 1984-1989. doi: 10.1177/00220345010800110701.
Monnouchi, S., Maeda, H., Fujii, S., Tomokiyo, A., Kono, K., and Akamine, A. (2011). The roles of angiotensin II in stretched periodontal ligament cells. *J. Dent. Res.* 90(2), 181-185. doi: 10.1177/0022034510382118.
Monnouchi, S., Maeda, H., Yuda, A., Hamano, S., Wada, N., Tomokiyo, A., et al. (2015). Mechanical induction of interleukin-11 regulates osteoblastic/cementoblastic differentiation of human periodontal ligament stem/progenitor cells. *J. Periodontal Res.* 50(2), 231-239. doi: 10.1111/jre.12200.
Nakashima, K., Tsuruga, E., Hisanaga, Y., Ishikawa, H., and Sawa, Y. (2009). Stretching stimulates fibulin-5 expression and controls microfibril bundles in human periodontal ligament cells. *J. Periodontal Res.* 44(5), 622-627. doi: 10.1111/j.1600-0765.2008.01170.x.
Narimiya, T., Wada, S., Kanzaki, H., Ishikawa, M., Tsuge, A., Yamaguchi, Y., et al. (2017). Orthodontic tensile strain induces angiogenesis via type IV collagen degradation by matrix metalloproteinase-12. *J. Periodontal Res.* 52(5), 842-852. doi: 10.1111/jre.12453.
Nazet, U., Schröder, A., Spanier, G., Wolf, M., Proff, P., and Kirschneck, C. (2020). Simplified method for applying static isotropic tensile strain in cell culture experiments with identification of valid RT-qPCR reference genes for PDL fibroblasts. *Eur. J. Orthod.* 42(4), 359-370. doi: 10.1093/ejo/cjz052.
Nemoto, T., Kajiya, H., Tsuzuki, T., Takahashi, Y., and Okabe, K. (2010). Differential induction of collagens by mechanical stress in human periodontal ligament cells. *Arch. Oral Biol.* 55(12), 981-987. doi: 10.1016/j.archoralbio.2010.08.004.
Ngan, P., Saito, S., Saito, M., Lanese, R., Shanfeld, J., and Davidovitch, Z. (1990). The interactive effects of mechanical stress and interleukin-1 beta on prostaglandin E and cyclic AMP production in human periodontal ligament fibroblasts in vitro: comparison with cloned osteoblastic cells of mouse (MC3T3-E1). *Arch. Oral Biol.* 35(9), 717-725. doi: 10.1016/0003-9969(90)90094-Q.

Nogueira, A.V., Nokhbehsaim, M., Eick, S., Bourauel, C., Jäger, A., Jepsen, S., et al. (2014a). Regulation of visfatin by microbial and biomechanical signals in PDL cells. *Clin. Oral Investig.* 18(1), 171-178. doi: 10.1007/s00784-013-0935-1.

Nogueira, A.V., Nokhbehsaim, M., Eick, S., Bourauel, C., Jäger, A., Jepsen, S., et al. (2014b). Biomechanical loading modulates proinflammatory and bone resorptive mediators in bacterial-stimulated PDL cells. *Mediators Inflamm.* 2014, 425421. doi: 10.1155/2014/425421.

Nokhbehsaim, M., Deschner, B., Bourauel, C., Reimann, S., Winter, J., Rath, B., et al. (2011a). Interactions of enamel matrix derivative and biomechanical loading in periodontal regenerative healing. *J. Periodontol.* 82(12), 1725-1734. doi: 10.1902/jop.2011.100678.

Nokhbehsaim, M., Deschner, B., Winter, J., Bourauel, C., Jäger, A., Jepsen, S., et al. (2012). Anti-inflammatory effects of EMD in the presence of biomechanical loading and interleukin-1β in vitro. *Clin. Oral Investig.* 16(1), 275-283. doi: 10.1007/s00784-010-0505-8.

Nokhbehsaim, M., Deschner, B., Winter, J., Bourauel, C., Rath, B., Jager, A., et al. (2011b). Interactions of regenerative, inflammatory and biomechanical signals on bone morphogenetic protein-2 in periodontal ligament cells. *J. Periodontal Res.* 46(3), 374-381. doi: 10.1111/j.1600-0765.2011.01357.x.

Nokhbehsaim, M., Deschner, B., Winter, J., Reimann, S., Bourauel, C., Jepsen, S., et al. (2010). Contribution of orthodontic load to inflammation-mediated periodontal destruction. *J. Orofac. Orthop.* 71(6), 390-402. doi: 10.1007/s00056-010-1031-7.

Ohzeki, K., Yamaguchi, M., Shimizu, N., and Abiko, Y. (1999). Effect of cellular aging on the induction of cyclooxygenase-2 by mechanical stress in human periodontal ligament cells. *Mech. Ageing Dev.* 108(2), 151-163. doi: 10.1016/s0047-6374(99)00006-8.

Ozawa, Y., Shimizu, N., and Abiko, Y. (1997). Low-energy diode laser irradiation reduced plasminogen activator activity in human periodontal ligament cells. *Lasers Surg. Med.* 21(5), 456-463. doi: 10.1002/(sici)1096-9101(1997)21:5<456::aid-lsm7>3.0.co;2-p.

Padial-Molina, M., Volk, S.L., Rodriguez, J.C., Marchesan, J.T., Galindo-Moreno, P., and Rios, H.F. (2013). Tumor necrosis factor-alpha and Porphyromonas gingivalis lipopolysaccharides decrease periostin in human periodontal ligament fibroblasts. *J. Periodontol.* 84(5), 694-703. doi: 10.1902/jop.2012.120078.

Pan, J., Wang, T., Wang, L., Chen, W., and Song, M. (2014). Cyclic strain-induced cytoskeletal rearrangement of human periodontal ligament cells via the Rho signaling pathway. *PloS One* 9(3), e91580. doi: 10.1371/journal.pone.0091580.

Papadopoulou, A., Iliadi, A., Eliades, T., and Kletsas, D. (2017). Early responses of human periodontal ligament fibroblasts to cyclic and static mechanical stretching. *Eur. J. Orthod.* 39(3), 258-263. doi: 10.1093/ejo/cjw075.

Papadopoulou, A., Todaro, A., Eliades, T., and Kletsas, D. (2019). Effect of hyperglycaemic conditions on the response of human periodontal ligament fibroblasts to mechanical stretching. *Eur. J. Orthod.* doi: 10.1093/ejo/cjz051.

Pelaez, D., Acosta Torres, Z., Ng, T.K., Choy, K.W., Pang, C.P., and Cheung, H.S. (2017). Cardiomyogenesis of periodontal ligament-derived stem cells by dynamic tensile strain. *Cell Tissue Res.* 367(2), 229-241. doi: 10.1007/s00441-016-2503-x.

Peverali, F.A., Basdra, E.K., and Papavassiliou, A.G. (2001). Stretch-mediated activation of selective MAPK subtypes and potentiation of AP-1 binding in human osteoblastic cells. *Mol. Med.* 7(1), 68-78.

Pinkerton, M.N., Wescott, D.C., Gaffey, B.J., Beggs, K.T., Milne, T.J., and Meikle, M.C. (2008). Cultured human periodontal ligament cells constitutively express multiple osteotropic cytokines and growth factors, several of which are responsive to mechanical deformation. *J. Periodontal Res.* 43(3), 343-351. doi: 10.1111/j.1600-0765.2007.01040.x.

Rath-Deschner, B., Deschner, J., Reimann, S., Jager, A., and Gotz, W. (2009). Regulatory effects of biomechanical strain on the insulin-like growth factor system in human periodontal cells. *J. Biomech.* 42(15), 2584-2589. doi: 10.1016/j.jbiomech.2009.07.013.

Ren, D., Wei, F., Hu, L., Yang, S., Wang, C., and Yuan, X. (2015). Phosphorylation of Runx2, induced by cyclic mechanical tension via ERK1/2 pathway, contributes to osteodifferentiation of human periodontal ligament fibroblasts. *J. Cell. Physiol.* 230(10), 2426-2436. doi: 10.1002/jcp.24972.

Ritter, N., Mussig, E., Steinberg, T., Kohl, A., Komposch, G., and Tomakidi, P. (2007). Elevated expression of genes assigned to NF-kappaB and apoptotic pathways in human periodontal ligament fibroblasts following mechanical stretch. *Cell Tissue Res.* 328(3), 537-548. doi: 10.1007/s00441-007-0382-x.

Saminathan, A., Vinoth, K.J., Wescott, D.C., Pinkerton, M.N., Milne, T.J., Cao, T., et al. (2012). The effect of cyclic mechanical strain on the expression of adhesion-related genes by periodontal ligament cells in two-dimensional culture. *J. Periodontal Res.* 47(2), 212-221. doi: 10.1111/j.1600-0765.2011.01423.x.

Shen, T., Qiu, L., Chang, H., Yang, Y., Jian, C., Xiong, J., et al. (2014). Cyclic tension promotes osteogenic differentiation in human periodontal ligament stem cells. *Int. J. Clin. Exp. Pathol.* 7(11), 7872-7880.

Shimizu, N., Goseki, T., Yamaguchi, M., Iwasawa, T., Takiguchi, H., and Abiko, Y. (1997). In vitro cellular aging stimulates interleukin-1 beta production in stretched human periodontal-ligament-derived cells. *J. Dent. Res.* 76(7), 1367-1375. doi: 10.1177/00220345970760070601.

Shimizu, N., Ozawa, Y., Yamaguchi, M., Goseki, T., Ohzeki, K., and Abiko, Y. (1998). Induction of COX-2 expression by mechanical tension force in human periodontal ligament cells. *J. Periodontol.* 69(6), 670-677. doi: 10.1902/jop.1998.69.6.670.

Shimizu, N., Yamaguchi, M., Goseki, T., Ozawa, Y., Saito, K., Takiguchi, H., et al. (1994). Cyclic-tension force stimulates interleukin-1 beta production by human periodontal ligament cells. *J. Periodontal Res.* 29(5), 328-333. doi: 10.1111/j.1600-0765.1994.tb01230.x.

Shimizu, N., Yamaguchi, M., Goseki, T., Shibata, Y., Takiguchi, H., Iwasawa, T., et al. (1995). Inhibition of prostaglandin E2 and interleukin 1-beta production by low-power laser irradiation in stretched human periodontal ligament cells. *J. Dent. Res.* 74(7), 1382-1388. doi: 10.1177/00220345950740071001.

Spencer, A.Y., and Lallier, T.E. (2009). Mechanical tension alters semaphorin expression in the periodontium. *J. Periodontol.* 80(10), 1665-1673. doi: 10.1902/jop.2009.090212.

Steinberg, T., Ziegler, N., Alonso, A., Kohl, A., Mussig, E., Proksch, S., et al. (2011). Strain response in fibroblasts indicates a possible role of the Ca(2+)-dependent nuclear transcription factor NM1 in RNA synthesis. *Cell Calcium* 49(4), 259-271. doi: 10.1016/j.ceca.2011.03.001.

Sun, C., Chen, L., Shi, X., Cao, Z., Hu, B., Yu, W., et al. (2016). Combined effects of proinflammatory cytokines and intermittent cyclic mechanical strain in inhibiting osteogenicity in human periodontal ligament cells. *Cell Biol. Int.* 40(9), 999-1007. doi: 10.1002/cbin.10641.

Sun, C., Liu, F., Cen, S., Chen, L., Wang, Y., Sun, H., et al. (2017). Tensile strength suppresses the osteogenesis of periodontal ligament cells in inflammatory microenvironments. *Mol. Med. Rep.* 16(1), 666-672. doi: 10.3892/mmr.2017.6644.

Suzuki, R., Nemoto, E., and Shimauchi, H. (2014). Cyclic tensile force up-regulates BMP-2 expression through MAP kinase and COX-2/PGE2 signaling pathways in human periodontal ligament cells. *Exp. Cell Res.* 323(1), 232-241. doi: 10.1016/j.yexcr.2014.02.013.

Symmank, J., Zimmermann, S., Goldschmitt, J., Schiegnitz, E., Wolf, M., Wehrbein, H., et al. (2019). Mechanically-induced GDF15 Secretion by Periodontal Ligament Fibroblasts Regulates Osteogenic Transcription. *Sci. Rep.* 9(1), 11516. doi:

10.1038/s41598-019-47639-x.
Takano, M., Yamaguchi, M., Nakajima, R., Fujita, S., Kojima, T., and Kasai, K. (2009). Effects of relaxin on collagen type I released by stretched human periodontal ligament cells. *Orthod. Craniofac. Res.* 12(4), 282-288. doi: 10.1111/j.1601-6343.2009.01463.x.
Tang, N., Zhao, Z., Zhang, L., Yu, Q., Li, J., Xu, Z., et al. (2012). Up-regulated osteogenic transcription factors during early response of human periodontal ligament stem cells to cyclic tensile strain. *Arch. Med. Sci.* 8(3), 422-430. doi: 10.5114/aoms.2012.28810.
Tantilertanant, Y., Niyompanich, J., Everts, V., Supaphol, P., Pavasant, P., and Sanchavanakit, N. (2019a). Cyclic tensile force stimulates BMP9 synthesis and in vitro mineralization by human periodontal ligament cells. *J. Cell. Physiol.* 234(4), 4528-4539. doi: 10.1002/jcp.27257.
Tantilertanant, Y., Niyompanich, J., Everts, V., Supaphol, P., Pavasant, P., and Sanchavanakit, N. (2019b). Cyclic tensile force-upregulated IL6 increases MMP3 expression by human periodontal ligament cells. *Arch. Oral Biol.* 107, 104495. doi: 10.1016/j.archoralbio.2019.104495.
Tsuji, K., Uno, K., Zhang, G.X., and Tamura, M. (2004). Periodontal ligament cells under intermittent tensile stress regulate mRNA expression of osteoprotegerin and tissue inhibitor of matrix metalloprotease-1 and -2. *J. Bone Miner. Metab.* 22(2), 94-103. doi: 10.1007/s00774-003-0456-0.
Tsuruga, E., Nakashima, K., Ishikawa, H., Yajima, T., and Sawa, Y. (2009). Stretching modulates oxytalan fibers in human periodontal ligament cells. *J. Periodontal Res.* 44(2), 170-174. doi: 10.1111/j.1600-0765.2008.01099.x.
Tsuruga, E., Oka, K., Hatakeyama, Y., Isokawa, K., and Sawa, Y. (2012). Latent transforming growth factor-beta binding protein 2 negatively regulates coalescence of oxytalan fibers induced by stretching stress. *Connect Tissue Res.* 53(6), 521-527. doi: 10.3109/03008207.2012.702816.
Wada, S., Kanzaki, H., Narimiya, T., and Nakamura, Y. (2017). Novel device for application of continuous mechanical tensile strain to mammalian cells. *Biol. Open* 6(4), 518-524. doi: 10.1242/bio.023671.
Wang, H., Feng, C., Jin, Y., Tan, W., and Wei, F. (2019a). Identification and characterization of circular RNAs involved in mechanical force-induced periodontal ligament stem cells. *J. Cell. Physiol.* 234(7), 10166-10177. doi: 10.1002/jcp.27686.
Wang, L., Pan, J., Wang, T., Song, M., and Chen, W. (2013). Pathological cyclic strain-induced apoptosis in human periodontal ligament cells through the RhoGDIalpha/caspase-3/PARP pathway. *PloS One* 8(10), e75973. doi: 10.1371/journal.pone.0075973.
Wang, Y., Hu, B., Hu, R., Tong, X., Zhang, M., Xu, C., et al. (2019b). TAZ contributes to osteogenic differentiation of periodontal ligament cells under tensile stress. *J. Periodontal Res.* doi: 10.1111/jre.12698.
Wang, Y., Li, Y., Fan, X., Zhang, Y., Wu, J., and Zhao, Z. (2011). Early proliferation alteration and differential gene expression in human periodontal ligament cells subjected to cyclic tensile stress. *Arch. Oral Biol.* 56(2), 177-186. doi: 10.1016/j.archoralbio.2010.09.009.
Wang, Y.F., Zuo, Z.H., Luo, P., Pang, F.S., and Hu, J.T. (2018). The effect of cyclic tensile force on the actin cytoskeleton organization and morphology of human periodontal ligament cells. *Biochem. Biophys. Res. Commun.* 506(4), 950-955. doi: 10.1016/j.bbrc.2018.10.163.
Wei, F., Liu, D., Feng, C., Zhang, F., Yang, S., Hu, Y., et al. (2015). microRNA-21 mediates stretch-induced osteogenic differentiation in human periodontal ligament stem cells. *Stem Cells Dev.* 24(3), 312-319. doi: 10.1089/scd.2014.0191.
Wei, F.L., Wang, J.H., Ding, G., Yang, S.Y., Li, Y., Hu, Y.J., et al. (2014). Mechanical force-induced specific MicroRNA expression in human periodontal ligament stem cells. *Cells Tissues Organs* 199(5-6), 353-363. doi: 10.1159/000369613.
Wescott, D.C., Pinkerton, M.N., Gaffey, B.J., Beggs, K.T., Milne, T.J., and Meikle, M.C. (2007). Osteogenic gene expression by human periodontal ligament cells under cyclic tension. *J. Dent. Res.* 86(12), 1212-1216. doi: 10.1177/154405910708601214.
Wolf, M., Lossdorfer, S., Kupper, K., and Jager, A. (2014). Regulation of high mobility group box protein 1 expression following mechanical loading by orthodontic forces in vitro and in vivo. *Eur. J. Orthod.* 36(6), 624-631. doi: 10.1093/ejo/cjt037.
Wu, J., Song, M., Li, T., Zhu, Z., and Pan, J. (2015). The Rho-mDia1 signaling pathway is required for cyclic strain-induced cytoskeletal rearrangement of human periodontal ligament cells. *Exp. Cell Res.* 337(1), 28-36. doi: 10.1016/j.yexcr.2015.07.016.
Wu, Y., Ou, Y., Liao, C., Liang, S., and Wang, Y. (2019a). High-throughput sequencing analysis of the expression profile of microRNAs and target genes in mechanical force-induced osteoblastic/cementoblastic differentiation of human periodontal ligament cells. *Am. J. Transl. Res.* 11(6), 3398-3411.
Wu, Y., Zhao, D., Zhuang, J., Zhang, F., and Xu, C. (2016). Caspase-8 and Caspase-9 Functioned Differently at Different Stages of the Cyclic Stretch-Induced Apoptosis in Human Periodontal Ligament Cells. *PloS One* 11(12), e0168268. doi: 10.1371/journal.pone.0168268.
Wu, Y., Zhuang, J., Zhao, D., and Xu, C. (2019b). Interaction between caspase-3 and caspase-5 in the stretch-induced programmed cell death in the human periodontal ligament cells. *J. Cell. Physiol.* 234(8), 13571-13581. doi: 10.1002/jcp.28035.
Wu, Y., Zhuang, J., Zhao, D., Zhang, F., Ma, J., and Xu, C. (2017). Cyclic stretch-induced the cytoskeleton rearrangement and gene expression of cytoskeletal regulators in human periodontal ligament cells. *Acta Odontol. Scand.* 75(7), 507-516. doi: 10.1080/00016357.2017.1347823.
Xu, C., Fan, Z., Shan, W., Hao, Y., Ma, J., Huang, Q., et al. (2012). Cyclic stretch influenced expression of membrane connexin 43 in human periodontal ligament cell. *Arch. Oral Biol.* 57(12), 1602-1608. doi: 10.1016/j.archoralbio.2012.07.002.
Xu, C., Hao, Y., Wei, B., Ma, J., Li, J., Huang, Q., et al. (2011). Apoptotic gene expression by human periodontal ligament cells following cyclic stretch. *J. Periodontal Res.* 46(6), 742-748. doi: 10.1111/j.1600-0765.2011.01397.x.
Xu, H., Bai, D., Ruest, L.B., Feng, J.Q., Guo, Y.W., Tian, Y., et al. (2015). Expression analysis of alpha-smooth muscle actin and tenascin-C in the periodontal ligament under orthodontic loading or in vitro culture. *Int. J. Oral Sci.* 7(4), 232-241. doi: 10.1038/ijos.2015.26.
Xu, H.Y., Nie, E.M., Deng, G., Lai, L.Z., Sun, F.Y., Tian, H., et al. (2017). Periostin is essential for periodontal ligament remodeling during orthodontic treatment. *Mol. Med. Rep.* 15(4), 1800-1806. doi: 10.3892/mmr.2017.6200.
Yamaguchi, M., Ozawa, Y., Nogimura, A., Aihara, N., Kojima, T., Hirayama, Y., et al. (2004). Cathepsins B and L increased during response of periodontal ligament cells to mechanical stress in vitro. *Connect Tissue Res.* 45(3), 181-189. doi: 10.1080/03008200490514149.
Yamaguchi, M., and Shimizu, N. (1994). Identification of factors mediating the decrease of alkaline phosphatase activity caused by tension-force in periodontal ligament cells. *Gen. Pharmacol.* 25(6), 1229-1235. doi: 10.1016/0306-3623(94)90142-2.
Yamaguchi, M., Shimizu, N., Goseki, T., Shibata, Y., Takiguchi, H., Iwasawa, T., et al. (1994). Effect of different magnitudes of tension force on prostaglandin E2 production by human periodontal ligament cells. *Arch. Oral Biol.* 39(10), 877-884. doi: 10.1016/0003-9969(94)90019-1.
Yamaguchi, M., Shimizu, N., Ozawa, Y., Saito, K., Miura, S., Takiguchi, H., et al. (1997). Effect of tension-force on plasminogen activator activity from human periodontal ligament cells. *J. Periodontal Res.* 32(3), 308-314. doi: 10.1111/j.1600-0765.1997.tb00539.x.

Yamaguchi, M., Shimizu, N., Shibata, Y., and Abiko, Y. (1996). Effects of different magnitudes of tension-force on alkaline phosphatase activity in periodontal ligament cells. *J. Dent. Res.* 75(3), 889-894. doi: 10.1177/00220345960750030501.

Yamaguchi, N., Chiba, M., and Mitani, H. (2002). The induction of c-fos mRNA expression by mechanical stress in human periodontal ligament cells. *Arch. Oral Biol.* 47(6), 465-471. doi: 10.1016/s0003-9969(02)00022-5.

Yamashiro, K., Myokai, F., Hiratsuka, K., Yamamoto, T., Senoo, K., Arai, H., et al. (2007). Oligonucleotide array analysis of cyclic tension-responsive genes in human periodontal ligament fibroblasts. *Int. J. Biochem. Cell Biol.* 39(5), 910-921. doi: 10.1016/j.biocel.2007.01.015.

Yang, S.Y., Kim, J.W., Lee, S.Y., Kang, J.H., Ulziisaikhan, U., Yoo, H.I., et al. (2015). Upregulation of relaxin receptors in the PDL by biophysical force. *Clin. Oral Investig.* 19(3), 657-665. doi: 10.1007/s00784-014-1276-4.

Yang, S.Y., Wei, F.L., Hu, L.H., and Wang, C.L. (2016). PERK-eIF2alpha-ATF4 pathway mediated by endoplasmic reticulum stress response is involved in osteodifferentiation of human periodontal ligament cells under cyclic mechanical force. *Cell. Signal.* 28(8), 880-886. doi: 10.1016/j.cellsig.2016.04.003.

Yang, Y., Wang, B.K., Chang, M.L., Wan, Z.Q., and Han, G.L. (2018). Cyclic Stretch Enhances Osteogenic Differentiation of Human Periodontal Ligament Cells via YAP Activation. *Biomed Res. Int.* 2018, 2174824. doi: 10.1155/2018/2174824.

Yang, Y., Yang, Y., Li, X., Cui, L., Fu, M., Rabie, A.B., et al. (2010). Functional analysis of core binding factor a1 and its relationship with related genes expressed by human periodontal ligament cells exposed to mechanical stress. *Eur. J. Orthod.* 32(6), 698-705. doi: 10.1093/ejo/cjq010.

Yang, Y.Q., Li, X.T., Rabie, A.B., Fu, M.K., and Zhang, D. (2006). Human periodontal ligament cells express osteoblastic phenotypes under intermittent force loading in vitro. *Front. Biosci.* 11, 776-781. doi: 10.2741/1835.

Yoshino, H., Morita, I., Murota, S.I., and Ishikawa, I. (2003). Mechanical stress induces production of angiogenic regulators in cultured human gingival and periodontal ligament fibroblasts. *J. Periodontal Res.* 38(4), 405-410. doi: 10.1034/j.1600-0765.2003.00660.x.

Yu, W., Hu, B., Shi, X., Cao, Z., Ren, M., He, Z., et al. (2018). Nicotine inhibits osteogenic differentiation of human periodontal ligament cells under cyclic tensile stress through canonical Wnt pathway and alpha7 nicotinic acetylcholine receptor. *J. Periodontal Res.* 53(4), 555-564. doi: 10.1111/jre.12545.

Yuda, A., Maeda, H., Fujii, S., Monnouchi, S., Yamamoto, N., Wada, N., et al. (2015). Effect of CTGF/CCN2 on osteo/cementoblastic and fibroblastic differentiation of a human periodontal ligament stem/progenitor cell line. *J. Cell. Physiol.* 230(1), 150-159. doi: 10.1002/jcp.24693.

Zhao, D., Wu, Y., Xu, C., and Zhang, F. (2017). Cyclic-stretch induces apoptosis in human periodontal ligament cells by activation of caspase-5. *Arch. Oral Biol.* 73, 129-135. doi: 10.1016/j.archoralbio.2016.10.009.

Zhao, D., Wu, Y., Zhuang, J., Xu, C., and Zhang, F. (2016). Activation of NLRP1 and NLRP3 inflammasomes contributed to cyclic stretch-induced pyroptosis and release of IL-1beta in human periodontal ligament cells. *Oncotarget* 7(42), 68292-68302. doi: 10.18632/oncotarget.11944.

Zhuang, J., Wang, Y., Qu, F., Wu, Y., Zhao, D., and Xu, C. (2019). Gasdermin-d Played a Critical Role in the Cyclic Stretch-Induced Inflammatory Reaction in Human Periodontal Ligament Cells. *Inflammation* 42(2), 548-558. doi: 10.1007/s10753-018-0912-6.

Ziegler, N., Alonso, A., Steinberg, T., Woodnutt, D., Kohl, A., Mussig, E., et al. (2010). Mechano-transduction in periodontal ligament cells identifies activated states of MAP-kinases p42/44 and p38-stress kinase as a mechanism for MMP-13 expression. *BMC Cell Biol.* 11, 10. doi: 10.1186/1471-2121-11-10.
