# Supplementary material for: Effect of Tension on Human Periodontal Ligament Cells: Systematic Review and Network Analysis
Source: Front Bioeng Biotechnol. 2021 Aug 27;9:695053. doi: 10.3389/fbioe.2021.695053 (PMC8429507; doi:10.3389/fbioe.2021.695053)
Supplement: Supplementary file 8 [file DataSheet8.pdf]

Supplement 8: Gene List Analysis

Summary of the pathway analysis applied using “dynamic” and “static” differential expressed gene (DEG) lists (Figures 3 and 5, Table 4). Pathway analysis was done quering the “GeneOntology/Biological Process” and GeneAnalytics’ “SuperPath” databases using the complete DEG lists (Table 4, Figures 5A and 5B) and the nodes included in the individual subnetworks (Figures 5C and 5D). To increase specificity, results from both databases were filtered according to the proportion of query genes in relation to the number of background genes of the specific database entry and a cut-off of ≥0.05 (i.e. 5 %) was applied.

Contents

Gene list – Dynamic tension DEGs *2*

Table S8.1 Enriched GeneOntology/Biological Process terms 2

Table S8.2 Enriched GeneAnalytics’ SuperPathways 3

Gene list – Static tension DEGs *5*

Table S8.3 Enriched GeneOntology Biological Process terms 5

Table S8.4 Enriched GeneAnalytics’ SuperPathways 6

Gene list – Dynamic tension DEGs

Table S8.1 Enriched GeneOntology/Biological Process terms

Ratio: Number of genes in gene set divided by the total number of background genes in the specific GeneOntology/Biological Process term. A ratio cut-off ≥ 0.05 was applied. The top 10 most significant enriched terms (i.e. those with the highest $-\log_{10}$(FDR) values) were selected.

Gene list	GO Term	Ratio	$-\log_{10}$(FDR)
Complete	GO.0009719: response to endogenous stimulus	0.05	36.16
	GO.0071495: cellular response to endogenous stimulus	0.05	30.14
	GO.0070848: response to growth factor	0.09	30.14
	GO.0001503: ossification	0.15	29.10
	GO.0071363: cellular response to growth factor stimulus	0.09	28.91
	GO.0051270: regulation of cellular component movement	0.06	27.80
	GO.0030334: regulation of cell migration	0.06	27.77
	GO.0072359: circulatory system development	0.06	27.54
	GO.2000145: regulation of cell motility	0.06	27.54
	GO.0045597: positive regulation of cell differentiation	0.06	27.36
Cluster #1	GO.0048661: positive regulation of smooth muscle cell proliferation	0.09	8.97
	GO.0007196: adenylate cyclase-inhibiting G protein-coupled glutamate receptor signaling pathway	0.56	8.97
	GO.1904705: regulation of vascular smooth muscle cell proliferation	0.12	8.38
	GO.0050729: positive regulation of inflammatory response	0.06	8.13
	GO.0032642: regulation of chemokine production	0.08	7.64
	GO.1904707: positive regulation of vascular smooth muscle cell proliferation	0.16	7.52
	GO.0007193: adenylate cyclase-inhibiting G protein-coupled receptor signaling pathway	0.07	7.44
	GO.0032722: positive regulation of chemokine production	0.09	6.61
	GO.0051966: regulation of synaptic transmission, glutamatergic	0.08	6.47
	GO.0031663: lipopolysaccharide-mediated signaling pathway	0.13	5.81
Cluster #2	GO.0070848: response to growth factor	0.05	23.23
	GO.0071363: cellular response to growth factor stimulus	0.05	22.47
	GO.0051216: cartilage development	0.12	19.23
	GO.0061448: connective tissue development	0.09	18.94
	GO.0003007: heart morphogenesis	0.08	17.64
	GO.0050678: regulation of epithelial cell proliferation	0.06	17.06
	GO.0048514: blood vessel morphogenesis	0.05	16.87
	GO.0055024: regulation of cardiac muscle tissue development	0.18	16.68
	GO.0060393: regulation of pathway-restricted SMAD protein phosphorylation	0.19	15.67
	GO.0003206: cardiac chamber morphogenesis	0.11	15.47
Cluster #3	GO.0060079: excitatory postsynaptic potential	0.09	14.10
	GO.0035235: ionotropic glutamate receptor signaling pathway	0.24	14.10
	GO.0098976: excitatory chemical synaptic transmission	0.38	6.97
	GO.0001964: startle response	0.11	5.79
	GO.0097553: calcium ion transmembrane import into cytosol	0.05	4.87
	GO.0060134: prepulse inhibition	0.14	3.82
	GO.1903539: protein localization to postsynaptic membrane	0.13	3.77
	GO.0035249: synaptic transmission, glutamatergic	0.09	3.51
	GO.0007616: long-term memory	0.06	3.20
Cluster #4	GO.0001649: osteoblast differentiation	0.10	12.50
	GO.0002076: osteoblast development	0.22	6.80
	GO.0031214: biomineral tissue development	0.06	6.56
	GO.0045124: regulation of bone resorption	0.08	4.09
	GO.0045669: positive regulation of osteoblast differentiation	0.05	3.60
	GO.0060346: bone trabecula formation	0.25	3.37
	GO.0060389: pathway-restricted SMAD protein phosphorylation	0.15	3.11
	GO.0034505: tooth mineralization	0.14	3.08
	GO.0042487: regulation of odontogenesis of dentin-containing tooth	0.14	3.08
	GO.0002063: chondrocyte development	0.09	2.77
Cluster #5	GO.0050718: positive regulation of interleukin-1 beta secretion	0.16	7.23

Gene list	GO Term	Ratio	$-log_{10}(FDR)$
	GO.0051402: neuron apoptotic process	0.05	2.96
Cluster 6	GO.0030199: collagen fibril organization	0.08	3.68
	GO.0044319: wound healing, spreading of cells	0.08	2.57
	GO.0042310: vasoconstriction	0.06	2.46
	GO.0045777: positive regulation of blood pressure	0.05	2.33

Table S8.2 Enriched GeneAnalytics' SuperPathways

Ratio: Number of genes in gene set divided by the total number of background genes in the specific SuperPath. The top 10 most significant enriched terms (i.e. those with the highest Score) were selected.

Gene list	SuperPath Name	Ratio	Score
Complete	ERK Signaling	0.05	115.97
	Akt Signaling	0.06	96.02
	PAK Pathway	0.06	95.94
	PEDF Induced Signaling	0.06	91.03
	TGF-Beta Pathway	0.06	84.03
	Interleukin-4 and 13 Signaling	0.18	70.16
	Lung Fibrosis	0.27	70.05
	Integrin Pathway	0.06	66.46
	Hippo Signaling Pathway	0.14	66.29
	Pathways in Cancer	0.06	65.92
Cluster #1	Rheumatoid Arthritis	0.08	37.32
	GPCRs, Class C Metabotropic Glutamate, Pheromone	0.31	37.04
	Th1 Differentiation Pathway	0.08	32.22
	IL-10 Pathway	0.14	31.41
	Taste Transduction	0.07	30.90
	T Cell Receptor Signaling Pathway	0.04	30.56
	Phospholipase D Signaling Pathway	0.04	30.40
	Transcription_Role of VDR in Regulation of Genes Involved in Osteoporosis	0.11	29.60
	Interleukin-10 Signaling	0.10	28.99
	Malaria	0.10	28.85
Cluster #2	ERK Signaling	0.03	81.42
	PAK Pathway	0.04	77.43
	PEDF Induced Signaling	0.04	74.29
	Akt Signaling	0.04	72.97
	TGF-Beta Pathway	0.04	70.06
	Interleukin-4 and 13 Signaling	0.13	66.96
	Pathways in Cancer	0.04	63.44
	Cardiomyocyte Differentiation Through BMP Receptors	0.45	62.49
	Human Embryonic Stem Cell Pluripotency	0.09	61.85
	TGF-beta Signaling Pathway (KEGG)	0.14	58.94
Cluster #3	Nicotine Addiction	0.18	63.13
	Amyotrophic Lateral Sclerosis (ALS)	0.10	47.79
	Amphetamine Addiction	0.07	44.99
	Circadian Entrainment	0.03	44.11
	CAMP Signaling Pathway	0.03	36.72
	Dopamine-DARPP32 Feedback Onto CAMP Pathway	0.03	36.29
	SALM Protein Interactions at The Synapses	0.19	34.67
	Post NMDA Receptor Activation Events	0.10	30.81
	Neurophysiological Process Glutamate Regulation of Dopamine D1A Receptor Signaling	0.06	27.81
	Protein-protein Interactions at Synapses	0.06	27.57
Cluster #4	Development_Hedgehog and PTH Signaling Pathways in Bone and Cartilage Development	0.13	34.33
	Transcription_Role of VDR in Regulation of Genes Involved in Osteoporosis	0.11	33.30
	Osteoblast Signaling	0.29	32.17
	Interleukin-11 Signaling Pathway	0.07	18.16
	FGF Signaling Pathway	0.06	17.88
	Regulation of Retinoblastoma Protein	0.05	17.12

Gene list	SuperPath Name	Ratio	Score
	TGF-beta Receptor Signaling (WikiPathways)	0.05	16.97
	Endochondral Ossification	0.05	16.49
	TGF-beta Signaling Pathways	0.03	14.82
	Parathyroid Hormone Synthesis, Secretion and Action	0.03	14.39
Cluster #5	NOD-like Receptor Signaling Pathway	0.03	41.05
	Nucleotide-binding Oligomerization Domain (NOD) Pathway	0.10	32.03
	Nucleotide-binding Domain, Leucine Rich Repeat Containing Receptor (NLR) Signaling Pathways	0.08	31.12
	Toll-like Receptor Signaling Pathway	0.01	26.29
	Apoptosis and Autophagy	0.02	23.41
	Necroptosis	0.02	16.71
	Shigellosis	0.01	15.09
	Innate Immune System	0.00	13.99
	Measles	0.01	13.68
Cluster #6	Integrin Pathway	0.02	39.05
	Phospholipase-C Pathway	0.02	34.17
	Focal Adhesion	0.02	33.83
	Degradation of The Extracellular Matrix	0.02	33.35
	Cytoskeleton Remodeling Regulation of Actin Cytoskeleton By Rho GTPases	0.03	30.98
	ERK Signaling	0.01	29.68
	Smooth Muscle Contraction	0.11	27.75
	MiRNA Targets in ECM and Membrane Receptors	0.09	26.73
	Actin Nucleation By ARP-WASP Complex	0.01	20.13
	Cell Adhesion_Endothelial Cell Contacts By Non-junctional Mechanisms	0.08	19.29

Gene list – Static tension DEGs

Table S8.3 Enriched GeneOntology Biological Process terms

Ratio: Number of genes in gene set divided by the total number of background genes in the specific GeneOntology/Biological Process term. A ratio cut-off ≥ 0.05 was applied. The top 10 most significant enriched terms (i.e. those with the highest -log_{10}(FDR) values) were selected.

Network	GO Term	Ratio	-log_{10}(FDR)
Complete	GO.0001503: ossification	0.06	10.08
	GO.0009612: response to mechanical stimulus	0.05	8.97
	GO.0031960: response to corticosteroid	0.06	8.91
	GO.0071260: cellular response to mechanical stimulus	0.10	8.40
	GO.0007565: female pregnancy	0.06	8.32
	GO.0062013: positive regulation of small molecule metabolic process	0.07	8.21
	GO.0051384: response to glucocorticoid	0.07	7.99
	GO.0097191: extrinsic apoptotic signaling pathway	0.09	7.88
	GO.0008625: extrinsic apoptotic signaling pathway via death domain receptors	0.19	7.54
	GO.0048660: regulation of smooth muscle cell proliferation	0.06	6.99
Cluster #1	GO.0008625: extrinsic apoptotic signaling pathway via death domain receptors	0.16	7.78
	GO.0097191: extrinsic apoptotic signaling pathway	0.06	7.66
	GO.2000134: negative regulation of G1/S transition of mitotic cell cycle	0.05	6.14
	GO.0031571: mitotic G1 DNA damage checkpoint	0.06	5.08
	GO.2000811: negative regulation of anoikis	0.17	4.91
	GO.0048661: positive regulation of smooth muscle cell proliferation	0.05	4.89
	GO.0001836: release of cytochrome c from mitochondria	0.14	4.74
	GO.0043567: regulation of insulin-like growth factor receptor signaling pathway	0.13	4.69
	GO.1900740: positive regulation of protein insertion into mitochondrial membrane involved in apoptotic signaling pathway	0.12	4.60
	GO.1902895: positive regulation of pri-miRNA transcription by RNA polymerase II	0.12	4.60
Cluster #2	GO.0022617: extracellular matrix disassembly	0.09	6.26
	GO.0051043: regulation of membrane protein ectodomain proteolysis	0.13	4.23
	GO.0002675: positive regulation of acute inflammatory response	0.12	4.18
	GO.0030574: collagen catabolic process	0.08	3.92
	GO.0070498: interleukin-1-mediated signaling pathway	0.06	3.68
	GO.0032963: collagen metabolic process	0.06	3.64
	GO.0051155: positive regulation of striated muscle cell differentiation	0.05	3.59
	GO.1905049: negative regulation of metallopeptidase activity	0.33	3.54
	GO.0031622: positive regulation of fever generation	0.29	3.47
	GO.0060558: regulation of calcidiol 1-monooxygenase activity	0.29	3.47
Cluster #3	GO.0042487: regulation of odontogenesis of dentin-containing tooth	0.14	3.48
	GO.0002076: osteoblast development	0.11	3.44
	GO.0071295: cellular response to vitamin	0.09	3.35
	GO.0001958: endochondral ossification	0.08	3.29
	GO.0045124: regulation of bone resorption	0.05	3.09
Cluster #4	GO.0006501: C-terminal protein lipidation	0.18	3.92
	GO.0044804: autophagy of nucleus	0.12	3.64
Cluster #5	GO.0045725: positive regulation of glycogen biosynthetic process	0.12	3.68
	GO.0046628: positive regulation of insulin receptor signaling pathway	0.13	3.68
	GO.0048009: insulin-like growth factor receptor signaling pathway	0.14	3.68
	GO.0014065: phosphatidylinositol 3-kinase signaling	0.06	3.54
Cluster #6	GO.0050919: negative chemotaxis	0.09	4.83
	GO.1990138: neuron projection extension	0.05	4.46
	GO.0071526: semaphorin-plexin signaling pathway	0.08	3.38
	GO.0048675: axon extension	0.06	3.19

Table S8.4 Enriched GeneAnalytics' SuperPathways

Ratio: Number of genes in gene set divided by the total number of background genes in specific SuperPath. The top 10 most significant enriched terms (i.e. those with the highest Score) were selected.

Gene list	SuperPath Name	Ratio	Score
Complete	Senescence and Autophagy in Cancer	0.15	76.37
	Transcription_Role of VDR in Regulation of Genes Involved in Osteoporosis	0.27	66.96
	ERK Signaling	0.02	64.47
	Apoptosis Modulation and Signaling	0.07	62.14
	Cell Adhesion_ECM Remodeling	0.20	61.73
	Pathways in Cancer	0.04	61.42
	Photodynamic Therapy-induced NF-kB Survival Signaling	0.23	59.03
	Akt Signaling	0.03	58.38
	Interleukin-4 and 13 Signaling	0.11	56.77
	Apoptotic Pathways in Synovial Fibroblasts	0.03	53.00
Cluster #2	Photodynamic Therapy-induced AP-1 Survival Signaling.	0.16	53.00
	TNFR1 Pathway	0.04	44.37
	DREAM Repression and Dynorphin Expression	0.04	43.47
	Direct P53 Effectors	0.06	43.04
	Toll-like Receptor Signaling Pathway	0.03	42.49
	Apoptosis Modulation and Signaling	0.04	42.00
	Pathways in Cancer	0.02	37.74
	Interleukin-4 and 13 Signaling	0.06	37.67
	Human Cytomegalovirus Infection	0.03	37.28
	Endometrial Cancer	0.03	36.40
Cluster #2	Photodynamic Therapy-induced NF-kB Survival Signaling	0.13	39.60
	Cell Adhesion_ECM Remodeling	0.10	37.53
	GPCR Pathway	0.01	36.02
	HIF-1 Signaling Pathway	0.06	32.53
	Interleukin-4 and 13 Signaling	0.05	32.15
	NF-KappaB Family Pathway	0.03	31.61
	Akt Signaling	0.01	31.17
	PAK Pathway	0.01	31.15
	Matrix Metalloproteinases	0.09	30.74
	Cytokine Signaling in Immune System	0.01	29.80
Cluster #3	Transcription_Role of VDR in Regulation of Genes Involved in Osteoporosis	0.09	35.40
	Osteoblast Signaling	0.21	29.60
	Development_Hedgehog and PTH Signaling Pathways in Bone and Cartilage Development	0.08	25.17
	Interleukin-11 Signaling Pathway	0.05	15.18
	FGF Signaling Pathway	0.04	14.99
	Notch-mediated HES/HEY Network	0.04	14.93
	Regulation of Retinoblastoma Protein	0.04	14.49
Cluster #4	Autophagy Pathway	0.05	31.79
	Autophagy - Animal	0.03	28.97
	Cellular Senescence (REACTOME)	0.01	22.08
	Senescence and Autophagy in Cancer	0.03	20.84
Cluster #5	Immune Response Function of MEF2 in T Lymphocytes	0.03	22.92
	Development IGF-1 Receptor Signaling	0.02	21.82
	EGFR Transactivation By Gastrin	0.12	18.92
	NFAT and Cardiac Hypertrophy	0.01	17.98
	IL-2 Pathway	0.01	17.95
	IGF1 Pathway	0.07	17.48
	Factors and Pathways Affecting Insulin-like Growth Factor (IGF1)-Akt Signaling	0.06	17.19
	P70S6K Signaling	0.01	17.18
	Integrins in Angiogenesis	0.04	16.05
	PI3K-Akt Signaling Pathway	0.01	15.92
Cluster #6	Axon Guidance	0.03	34.21
	Semaphorin Interactions	0.04	20.53
